# Supplementary material for: Cyclin D3 restricts SARS‐CoV‐2 envelope incorporation into virions and interferes with viral spread
Source: EMBO J. 2022 Oct 10;41(22):e111653. doi: 10.15252/embj.2022111653 (PMC9539236; doi:10.15252/embj.2022111653)
Supplement: Supplementary file 1 — Appendix [file EMBJ-41-e111653-s001.pdf]

Cyclin D3 restricts SARS-CoV-2 Envelope incorporation into virions and interferes with viral spread

Ravi K. Gupta[1,2,3], Petra Mlcochova[1,2,*]
1 Cambridge Institute of Therapeutic Immunology & Infectious Disease (CITIID), Cambridge, UK.
2 Department of Medicine, University of Cambridge, Cambridge, UK.
3 Africa Health Research Institute, Durban, South Africa.
* Corresponding author

APPENDIX MATERIAL

Table of Contents

Appendix Figure S1. ***SARS-CoV-2 infection relocalises cyclin D3 from the nucleus in HeLa ACE2 cells.*** *2*
Appendix Figure S2. ***Effect of proteasome inhibition on SARS-CoV-2 infection*** *.* *3*
Appendix Figure S3. ***Depletion of cyclins by siRNA and genome replication*** *.* *4*
Appendix Figure S4. ***D-cyclin knock-down arrests cell cycle in uninfected cells*** *.* *5*
Appendix Figure S5. ***Cyclin D3 degradation is independent from syncytium formation*** *.* *6*

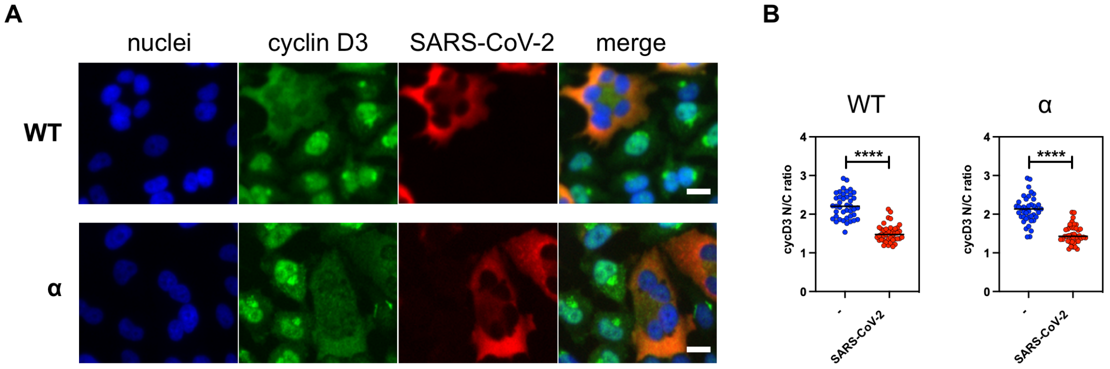

Appendix Figure S1. **SARS-CoV-2 infection relocalises cyclin D3 from the nucleus in HeLa ACE2 cells.**

(A) HeLa cells expressing ACE2 were infected with WT and α variants and 24h later fixed and stained for SARS-CoV-2 nucleocapsid and cyclin D3. Scale bar: 20μm.

(B) Quantification of ratio between nuclear and cytoplasm (N/C) staining of cyclin D3. Statistical analysis was performed using two-sided unpaired Student's t-tests; ***$p < 0.001$.

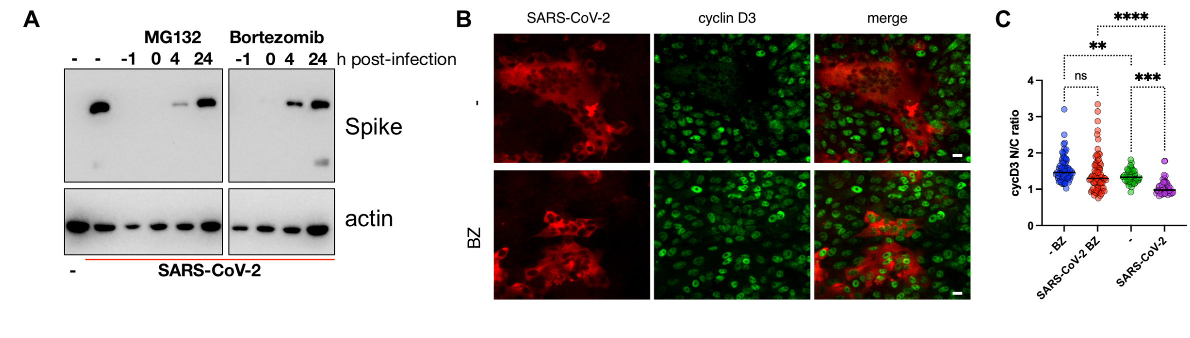

Appendix Figure S2. **Effect of proteasome inhibition on SARS-CoV-2 infection.**

(A) Proteasome inhibitors MG-132(1µM), Bortezomib (BZ, 1µM) were added to A549 AT2 cells 1h before infection (-1), at infection (0), 4h (4) and 24h (24) post-infection. Cells were infected with Delta SARS-CoV-2 variant and lysed 28h post-infection, viral protein Spike as measure of infection was detected by western blot.

(B) VERO AT2 cells were infected with Delta SARS-CoV-2 variant. Proteasome inhibitor Bortezomib (BZ, 1µM) was added to cells 8h later. Cells were fixed and stained 24h post-addition of inhibitor. Scale bar: 20µm.

(C) Quantification of D3 cyclin relocalization from nucleus after infection. Uninfected (-) and SARS-CoV-2 infected cells were identified by negative/positive nucleocapsid staining. Ratio between nuclear and cytoplasm (N/C ratio) staining intensity of cyclins was measured using ImageJ and Harmony (PerkinElmer). At least 50 cells have been counted. Ordinary two way ANOVA; ns, non-significant; ****$p < 0.0001$; ***$p < 0.001$; **$p < 0.01$.

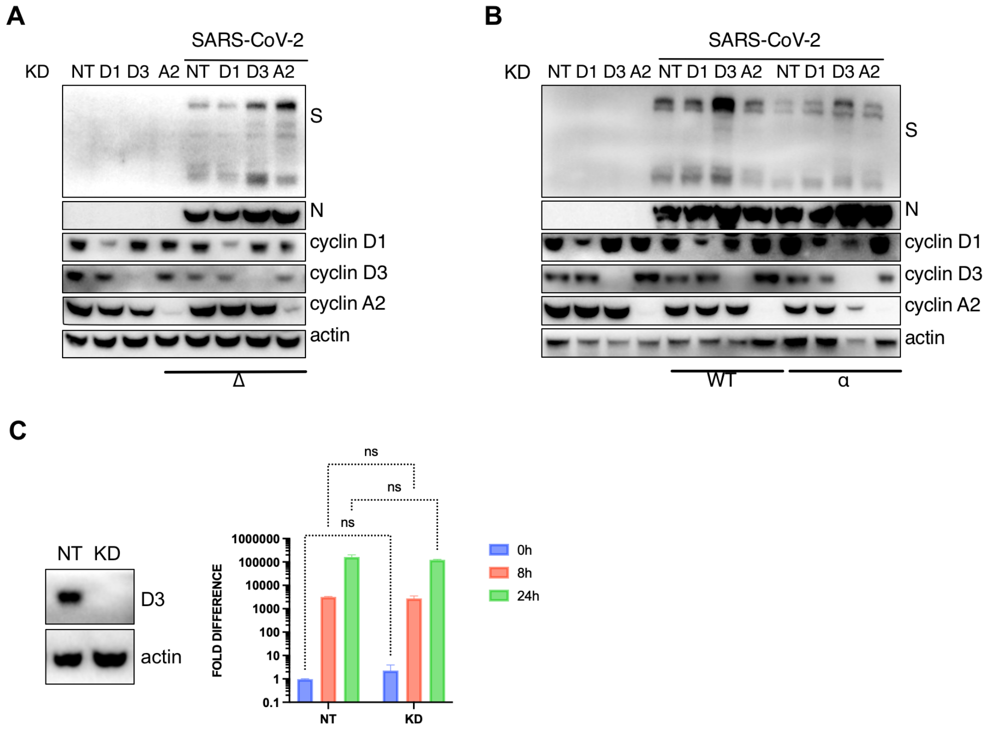

Appendix Figure S3. **Depletion of cyclins by siRNA and genome replication.**

(A,B) D and A-cyclins were depleted using siRNA. A549 AT2 Cells were infected 18h later with Delta (Δ), Alpha (α) or wild type (WT) SARS-CoV-2 variants at MOI 0.001, 0.1, 0.1 respectively. Cells were washed 4h post-infection and new media added. Cells were collected 48h later, viral proteins and cyclins detected using western blotting.

(C) **Cyclin D3 does not affect genome replication.** Cyclin D3 was depleted from VERO AT2 cells using siRNA, cells were infected 24h later and cells were collected at 8h (1 round of replication) and 24h (multiple rounds) post-infection. cDNA was prepared from isolated RNA and used in qPCR to detect nucleocapsid transcripts. n = 2 biological replicates; ordinary two way ANOVA with Dunnett's multiple comparisons test: ns, non-significant. Bars indicate mean with SD. NT, non-target siRNA. KD, knock-down.

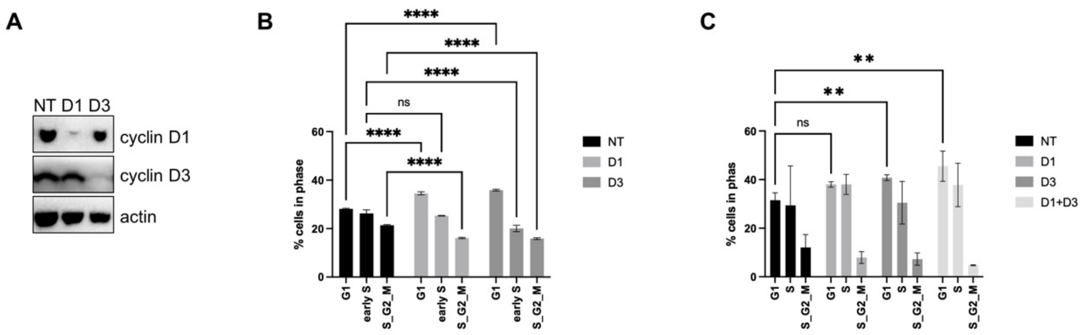

Appendix Figure S4. **D-cyclin knock-down arrests cell cycle in uninfected cells.**

(A-C) Cyclins D1, D3 were depleted by siRNA. Cells were transduced with Fucci VSV-G pseudotype virus 18h later and flow cytometry used to identify cell cycle phases 24h later.

(A) Western blot of cell lysates from A549 AT2 cells depleted for various cyclins.

(B) Flow cytometry analysis of cell cycle comparing cyclin D1, D3 knockdown to NT (non-target siRNA) in A549 AT2 cells. n = 3 biological replicates; ordinary two way ANOVA with Dunnett's multiple comparisons test: ns, non-significant; $^{****}p < 0.0001$; $^{***}p < 0.001$; $^{*}p < 0.1$. Bars indicate mean with SD.

(C) Flow cytometry analysis of cell cycle comparing cyclin D1, D3, combined D1+D3 knockdown to NT (non-target siRNA) in VERO AT2 cells. n = 3 biological replicates; ordinary two way ANOVA with Dunnett's multiple comparisons test: ns, non-significant; $^{**}p < 0.01$; $^{*}p < 0.1$. Bars indicate mean with SD.

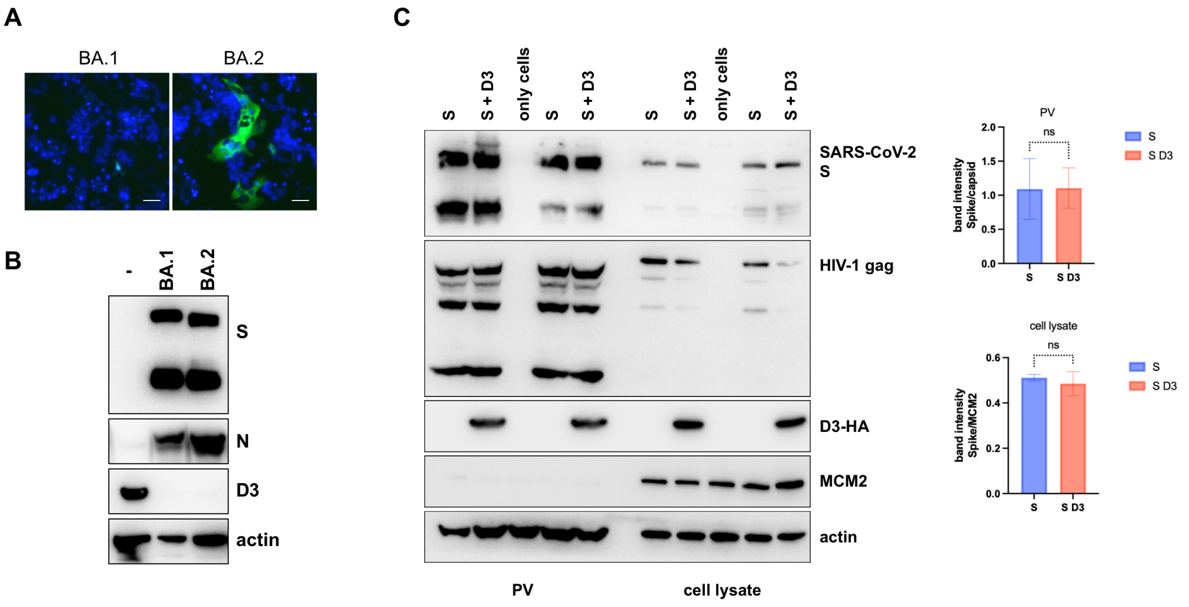

Appendix Figure S5. **Cyclin D3 degradation is independent from syncytium formation. Cyclin D3 does not change Spike incorporation into pseudotyped viral particles.**

(A,B) **Cyclin D3 degradation is independent from syncytium formation.**

(A) GFP split system was used to visualise syncytia formation after expression of Omicron Spike protein from lineages BA.1 and BA.2. Scale bar: 20µm.

(B) VERO AT2 cell were infected with BA.1 and BA.2 at MOI 0.1. Cells were collected 48h later and subjected to western blot. NP, nucleocapsid.

(C) **Cyclin D3 does not change Spike incorporation into pseudotyped viral particles.** SARS-CoV-2 Spike containing HIV-1 gag-pol based pseudotyped particles (PV) were generated in the presence or absence of cyclin D3. PV were purified and subjected to western blotting. Graphs represent quantification of immunoblot. n=2 biological replicates. Statistical analysis was performed using two-sided unpaired Student's t-tests; ns, non-significant. S, spike.
