# Supplementary material for: Cyclin D3 restricts SARS‐CoV‐2 envelope incorporation into virions and interferes with viral spread
Source: EMBO J. 2022 Oct 10;41(22):e111653. doi: 10.15252/embj.2022111653 (PMC9539236; doi:10.15252/embj.2022111653)
Supplement: Supplementary file 2 — Expanded View Figures PDF [file EMBJ-41-e111653-s009.pdf]

Expanded View Figures

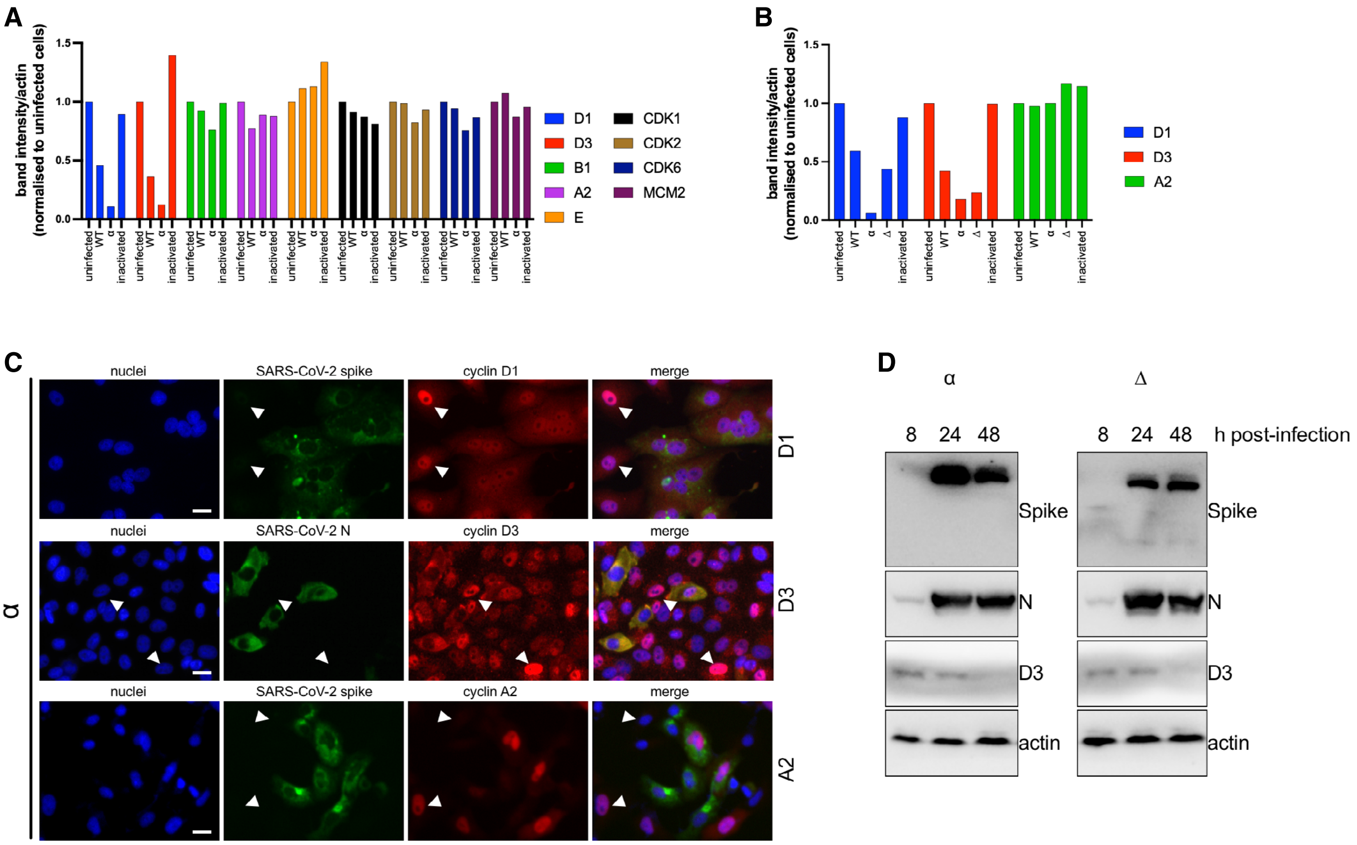

Figure EV1. Densitometry data from cell lysates from SARS-CoV-2-infected cells.

A, B ImageJ was used to record densitometry of bands from Fig 1A and D. All band densities were normalised to actin and further normalised to uninfected cells (=1). (A) VERO AT2 cell line. (B) A549 AT2 cell line.

C VERO AT2 cells were infected with Alpha (α) SARS-CoV-2 variant. Cells were fixed 24 h post-infection and stained for viral proteins and cyclins. Arrowheads highlight uninfected cells and cyclin D/A nuclear localisation. Arrowheads: Nuclear cyclin staining in uninfected cells. Scale bars: 20 μm.

D Calu3 cells were infected with Alpha (α) and Delta (Δ) SARS-CoV-2 variants at MOI 0.1. Cells were lysed 8, 24 and 48 h post-infection and viral proteins and cyclin D3 expression were analysed by western blot. N, nucleocapsid.

Source data are available online for this figure.

 © 2022 The Authors

Figure EV2. SARS-CoV-2 infection of VERO AT2 arrests cells in S and G2/M phases.

A Fluorescence ubiquitination cell cycle indicator (Fucci) cell cycle sensor is a two-colour (red and green) indicator. Red: RFP-Cdt1 protein is expressed in G1 phase. GFP-geminin protein is expressed in S, G2 and M phases. Both proteins are expressed in the early S phase (both red and green colours).

B Cell cycle analysis can be performed using flow cytometry. G0/neg population of cells cannot be analysed as it comprises of cells that are in G0 phase and/or were not transduced by Fucci-containing lentiviral particles.

C Automated microscope platform and ImageJ and/or Harmony imaging software (PerkinElmer) analysis can be used to study cell cycle changes. Scale bar: 20 μm.

D–F (D) VERO AT2 cells were transduced with Fucci-containing lentiviral particles for 18 h and infected with SARS-CoV-2 WT in the absence (−) or presence of Chloroquine (CQ), Remdesivir (RVD) or infected with heat-inactivated virus for additional 24 h. The percentage of infected cells was determined by staining of SARS-CoV-2 nucleocapsid (NP) in infected cells using flow cytometry. (E) Western blot for SARS-CoV-2 spike protein as a measure of infection in cells. (F) Analysis of cell cycle phases. $n = 3$ biological replicates; Statistical analysis was performed using two-sided unpaired Student's t-tests; ns, non-significant; ***$P < 0.001$; **$P < 0.01$. Bars indicate mean with SD.

G, H VERO AT2 cells were transduced with Fucci-containing lentiviral particles for 18 h and infected with SARS-CoV-2 WT at different MOI for 24 h. (G) Cells were lysed and used for Western blot. (H) Cells and their cell cycle status were analysed using Flow cytometry. $n = 3$ biological replicates; one-way ANOVA with Dunnett's multiple comparisons test: ***$P < 0.001$; **$P < 0.01$. Bars indicate the mean with SD.

I, J A549 AT2 cells were transduced with Fucci-containing lentiviral particles for 18 h and infected with SARS-CoV-2 variants for an additional 24 h. Comparison of uninfected cell populations from truly uninfected cells (not exposed to virus, uninfected) and cells exposed to SARS-CoV-2 but uninfected (nucleocapsid negative, uninfected (INF)) or infected (nucleocapsid positive). (I) Example of gating strategy for cell cycle analysis. (J) Quantification of cell cycle arrest in early S phase after exposure of SARS-CoV-2 variants. α, Alpha; Δ, Delta; WT, Wuhan. $n = 3$ biological replicates; two-way ANOVA test: ns, non-significant; ****$P < 0.0001$; ***$P < 0.001$; **$P < 0.01$. Bars indicate the mean with SD.

Source data are available online for this figure.

© 2022 The Authors

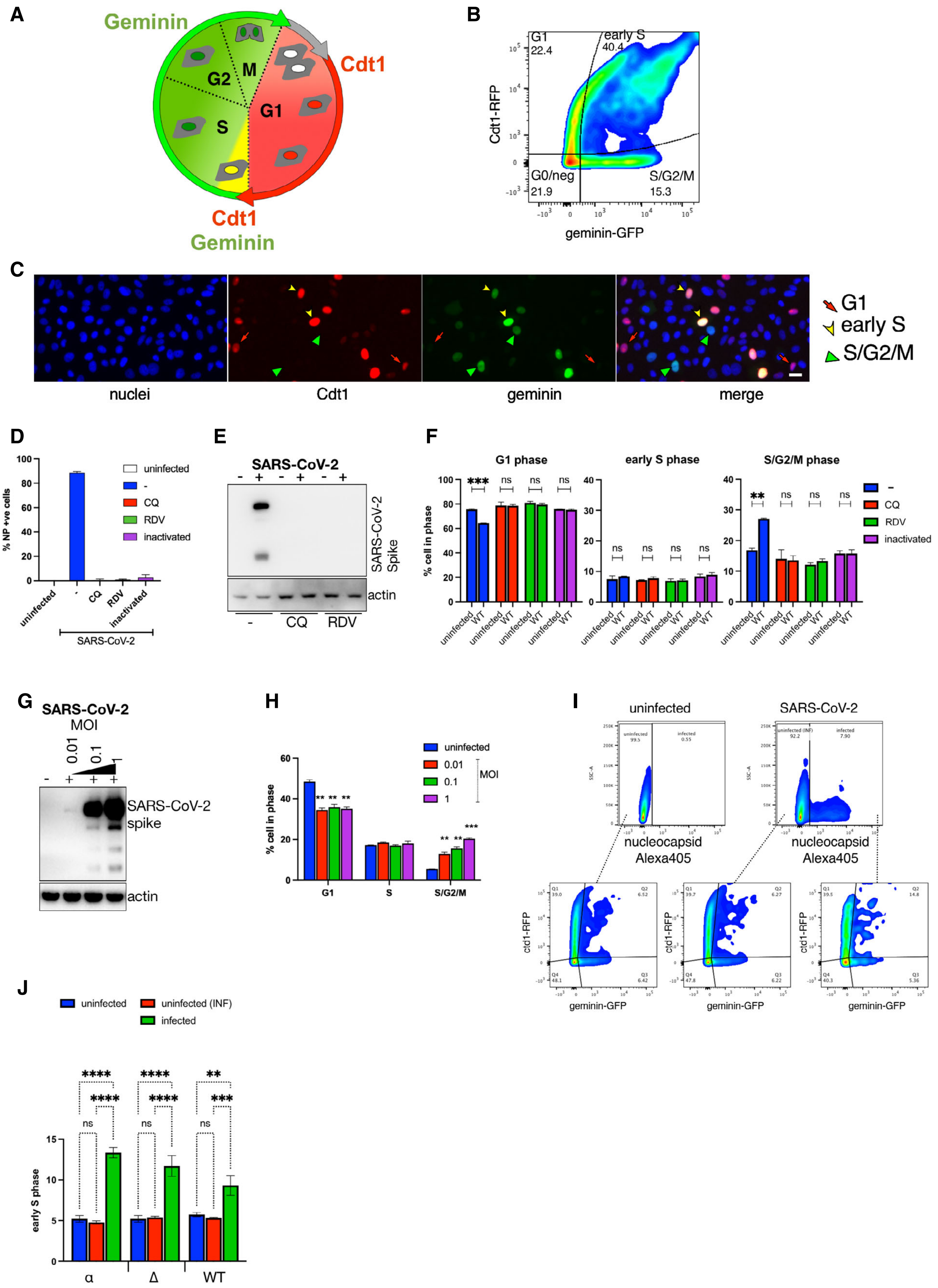

Figure EV2.

 © 2022 The Authors

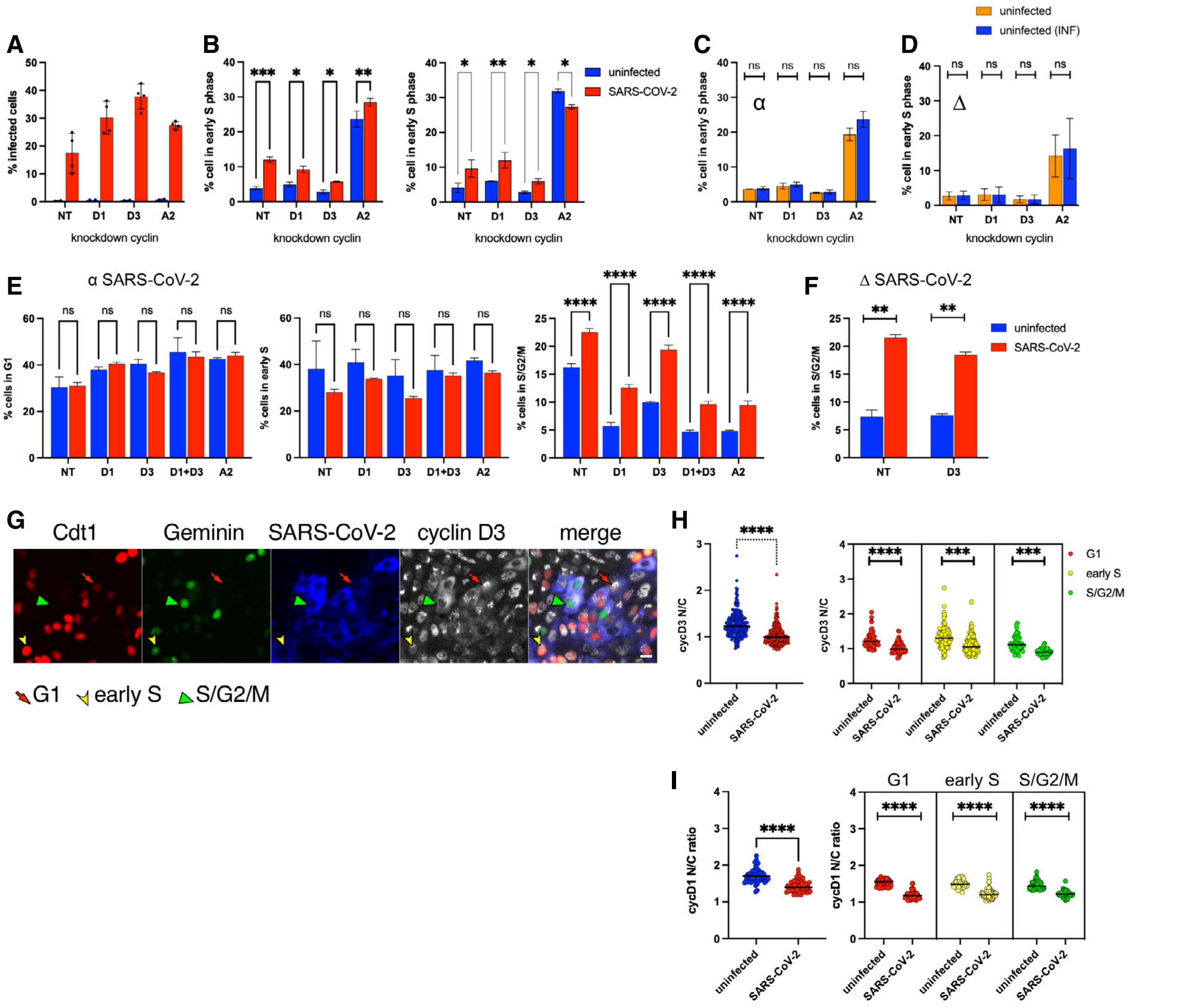

Figure EV3. SARS-CoV-2-mediated depletion of D-cyclins is cell cycle arrest independent.

A–D A549 AT2 cells were depleted for D and A2 cyclins and 18 h later infected with Alpha variant SARS-CoV-2 for 24 h. Cells were fixed, stained for SARS-CoV-2 nucleocapsid and analysed for infection and Fucci cell cycle sensor. (A) Percentage of infected cells in cells depleted for cyclins. $n = 2$ biological replicates. Bars indicate the mean with SD. (B) Flow cytometry analysis of early S cell cycle phase comparing cyclin D1, D3 and A2 knockdown to NT (non-target siRNA) in two independent experiments in duplicates. Statistical analysis was performed using two-sided unpaired Student's *t*-tests; ns, non-significant; ***$P < 0.001$; **$P < 0.01$; *$P < 0.1$. Bars indicate the mean with SD. (C, D) Comparison of uninfected cell populations from truly uninfected cells (not exposed to virus and uninfected) and cells exposed to SARS-CoV-2 but uninfected (nucleocapsid negative and uninfected (INF)). A549 AT2 cells were transduced with Fucci-containing lentiviral particles for 18 h and infected with (C) Alpha and (D) Delta SARS-CoV-2 variants for an additional 24 h. $n = 3$ biological replicates. Statistical analysis was performed using two-sided unpaired Student's *t*-test: ns, non-significant. Bars indicate the mean with SD.

E–I VERO AT2 cells were transduced with VSV-G pseudotyped Fucci-containing lentiviral particles and 18 h later infected with Alpha SARS-CoV-2. Cells were fixed and stained for SARS-CoV-2 nucleocapsid, D-cyclins and analysed for infection and Fucci cell cycle sensor 24 h later. Statistical analysis was performed using two-sided unpaired Student's *t*-tests; ns, non-significant. Bars indicate the mean with SD. (E) VERO AT2 cells infected with Alpha variant. Flow cytometry analysis of cell cycle comparing cyclin D1, D3 and combined D1 + D3 knockdown to NT (non-target siRNA) in uninfected and SARS-CoV-2-infected cells. The plot is an example of three biological replicates in technical duplicates. Statistical analysis was performed using two-sided unpaired Student's *t*-test: ns, non-significant; ****$P < 0.0001$. Bars indicate the mean with SD. (F) VERO AT2 cells infected with Delta variant. Flow cytometry analysis of S/G2/M cell cycle phase comparing cyclin D3 knockdown to NT (non-target siRNA). $n = 3$ biological replicates. Statistical analysis was performed using two-sided unpaired Student's *t*-test: ns, non-significant; **$P < 0.01$. Bars indicate the mean with SD. (G) Example of acquisition using the automated microscopic platform. Cells are identified for infection, cell cycle (Red/arrow = G1phase; Green/arrowhead = S/G2/M; Red+Green/arrowhead = early S) and expression of cyclin D3. Scale bar: 20 μm. (H, I) Quantification of cyclin D3 relocalisation from the nucleus to cytoplasm and correlation with cell cycle phases using ImageJ and Harmony (PerkinElmer). (H) Cyclin D3. (I) Cyclin D1. At least 50–200 cells were analysed in each condition. Bars indicate the mean with SD. Statistical analysis was performed using two-sided unpaired Student's *t*-test: ****$P < 0.0001$; ***$P < 0.001$.

Source data are available online for this figure.

© 2022 The Authors

Figure EV4. Cyclin D3 associates with E and M proteins.

A Western blot of cell lysates from cell lines used in this study, detecting endogenous expression of cyclin D1 and D3.

B 293T cells were contransfected with HA-cyclin D3 and SARS-CoV-2 spike, or Strep-tagged E, M, N or nsp9. Whole-cell lysates show expression levels of protein input into immunoprecipitation.

C–E Immunoprecipitation was performed using (C) mouse anti-cyclin D3 antibody, (D) anti-HA antibody and (E) anti-Strep beads. The immunoprecipitates were blotted and stained with anti-Strep, anti-HA and anti-spike antibodies. *Non-specific band. N, nucleocapsid; E, envelope; M, membrane; S, spike.

Source data are available online for this figure.

 © 2022 The Authors

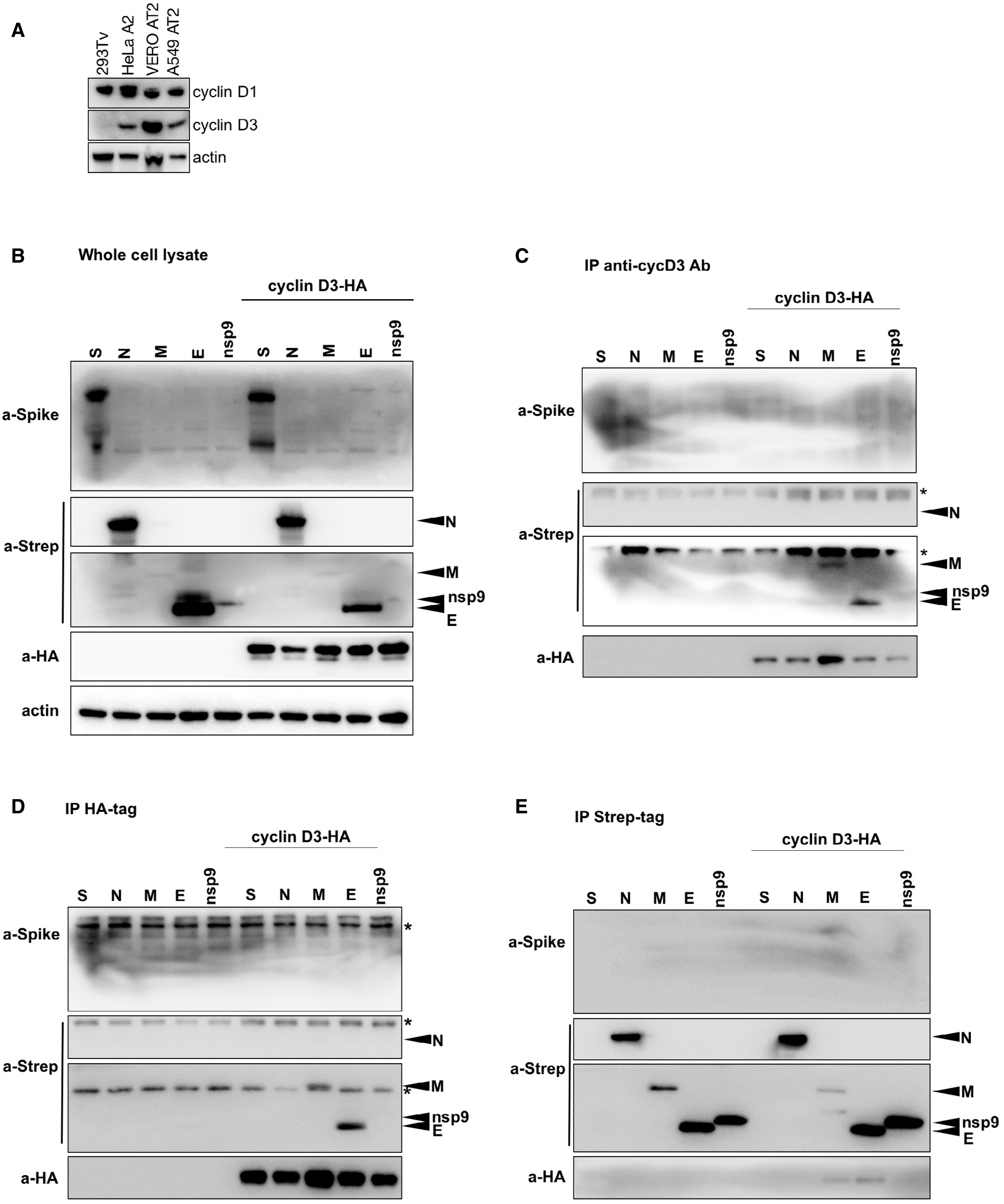
A
293Tv
HeLa A2
VERO AT2
A549 AT2
cyclin D1
cyclin D3
actin
B
Whole cell lysate
cyclin D3-HA
S N M E nsp9 S N M E nsp9
a-Spike
a-Strep
N
M
nsp9
E
a-HA
actin
C
IP anti-cycD3 Ab
cyclin D3-HA
S N M E nsp9 S N M E nsp9
a-Spike
a-Strep
*
N
*
M
nsp9
E
a-HA
D
IP HA-tag
cyclin D3-HA
S N M E nsp9 S N M E nsp9
a-Spike
*
a-Strep
*
N
M
*
nsp9
E
a-HA
E
IP Strep-tag
cyclin D3-HA
S N M E nsp9 S N M E nsp9
a-Spike
a-Strep
N
M
nsp9
E
a-HA

Figure EV4.

Figure EV5. Envelope and membrane proteins are not responsible for cyclin D degradation.

293T cells were cotransfected with SARS-CoV-2 genes (tagged with Strep-Tag) and cyclin D3.

A Cell lysates were collected 24 h post-transfection and subjected to western blotting.

B Cyclin D3 expression/degradation was quantified as cyclin D3 band intensity and normalised to actin. $n = 5$ biological replicates; ordinary one-way ANOVA with Dunnett's multiple comparisons test: ***$P < 0.001$; *$P < 0.1$. Bars indicate the mean with SD.

C, D Examples of two independent experiments demonstrating experimental variation. EV, empty vector; GFP, Strep-tagged GFP expressed.

Source data are available online for this figure.

© 2022 The Authors

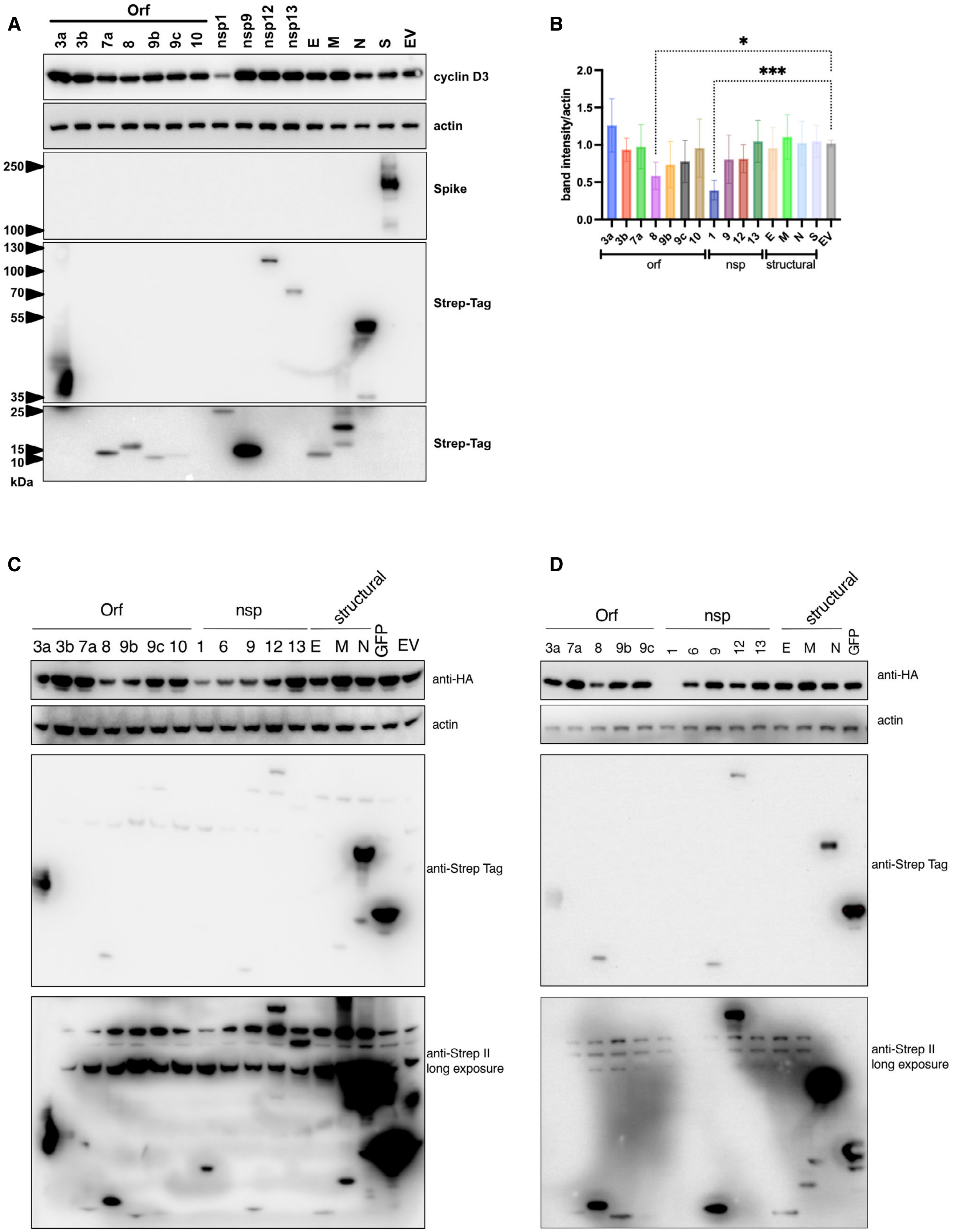
A
Orf
3a
3b
7a
8
9b
9c
10
nsp1
nsp9
nsp12
nsp13
E
M
N
S
EV
cyclin D3
actin
250
100
Spike
130
100
70
55
35
Strep-Tag
25
15
10
kDa
Strep-Tag
B
band intensity/actin
2.0
1.5
1.0
0.5
0.0
*
***
3a 3b 7a 8 9b 9c 10 1 9 12 13 E M N S EV
orf
nsp
structural
C
Orf
nsp
structural
3a 3b 7a 8 9b 9c 10 1 6 9 12 13 E M N GFP EV
anti-HA
actin
anti-Strep Tag
anti-Strep II
long exposure
D
Orf
nsp
structural
3a 7a 8 9b 9c 1 6 9 12 13 E M N GFP
anti-HA
actin
anti-Strep Tag
anti-Strep II
long exposure

Figure EV5.
