# Supplementary material for: Cyclin D3 restricts SARS‐CoV‐2 envelope incorporation into virions and interferes with viral spread
Source: EMBO J. 2022 Oct 10;41(22):e111653. doi: 10.15252/embj.2022111653 (PMC9539236; doi:10.15252/embj.2022111653)

## EV10

### EV10A

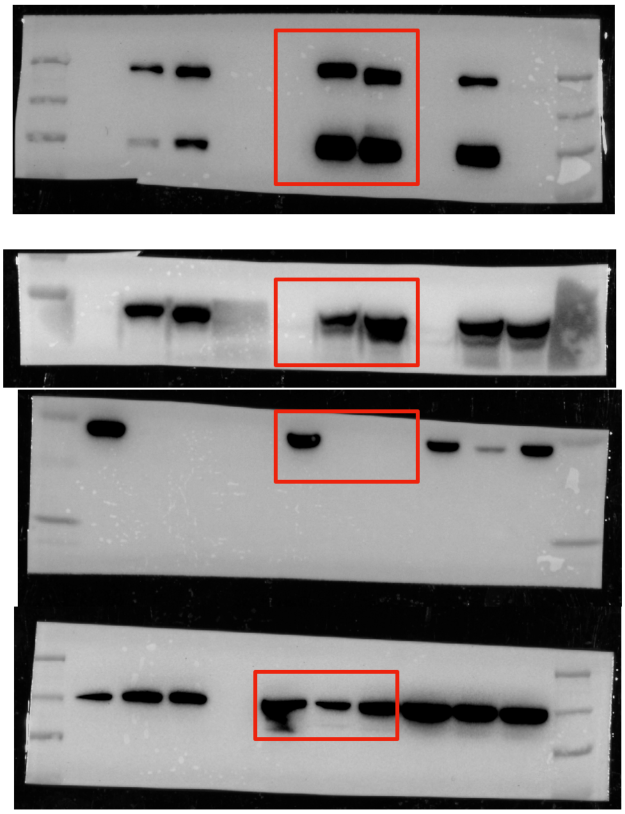


### EV10B

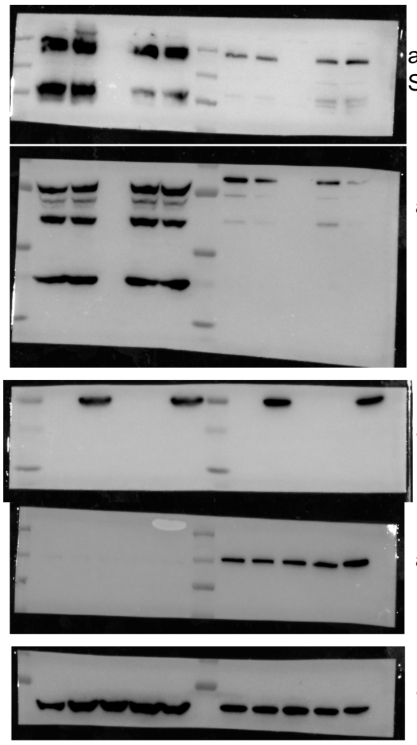

Supplement: Supplementary file 3 — Source Data for Expanded View and Appendix [file EMBJ-41-e111653-s005.zip › appendix/EMBOJ-2022-111653R-Figure_Appendix_S5_Source_Data-sd.pdf]
