# Supplementary material for: Cyclin D3 restricts SARS‐CoV‐2 envelope incorporation into virions and interferes with viral spread
Source: EMBO J. 2022 Oct 10;41(22):e111653. doi: 10.15252/embj.2022111653 (PMC9539236; doi:10.15252/embj.2022111653)
Supplement: Supplementary file 3 — Source Data for Expanded View and Appendix [file EMBJ-41-e111653-s005.zip › fig EV5/panel A C D/EMBOJ-2022-111653R-Figure_EV5_Source_Data-sd.pdf]

EV 5

EV5A

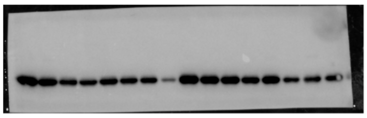

anti-HA (cyclin D3)

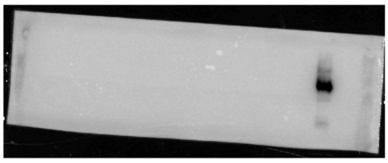

anti-Spike

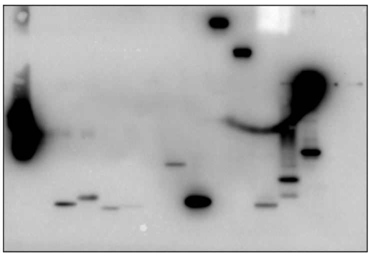

anti-Strep Tag (SARS-CoV-2 proteins)

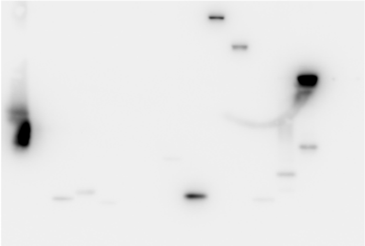

anti-Strep Tag (SARS-CoV-2 proteins)

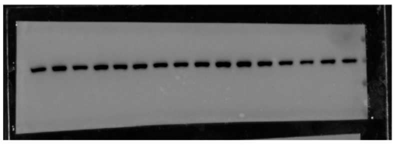

anti-actin

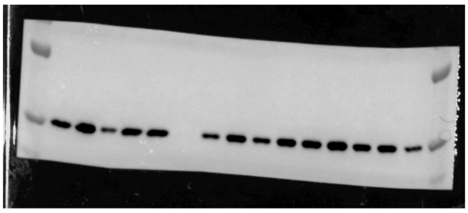

EV5C

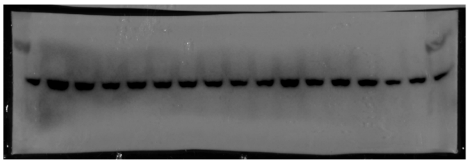

anti-actin

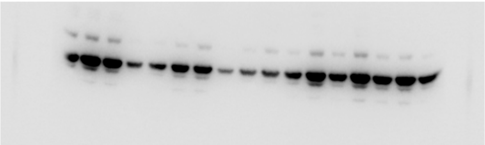

cyclin D3 (anti-HA)

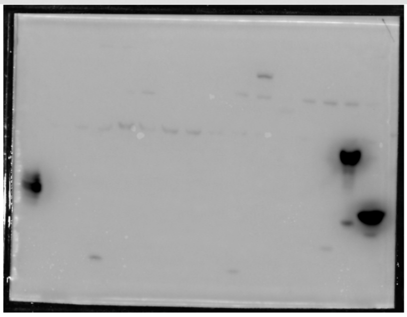

anti-strep tag

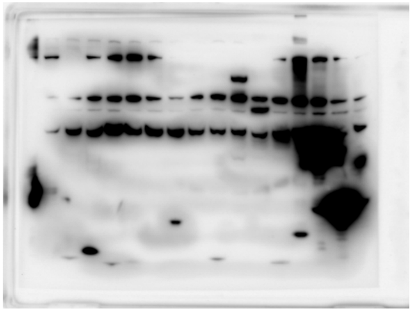

anti-strep tag longer exposure

EV5D

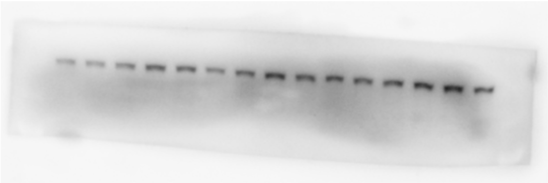

anti-actin

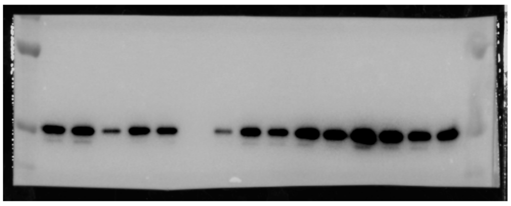

anti-HA (cyclin D3)

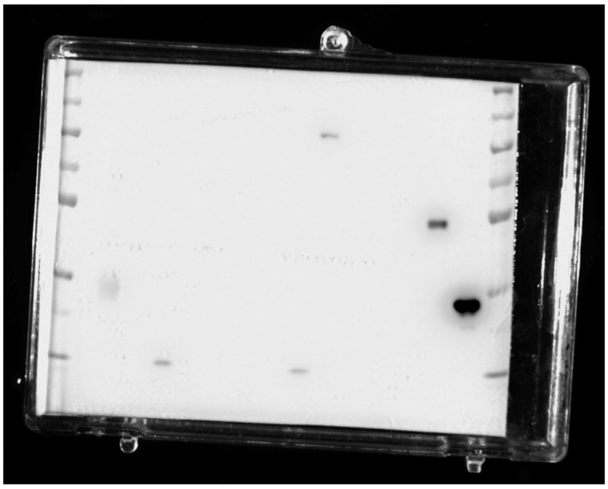

anti-Strep Tag (SARS-CoV-2 proteins)
