# Supplementary material for: Cyclin D3 restricts SARS‐CoV‐2 envelope incorporation into virions and interferes with viral spread
Source: EMBO J. 2022 Oct 10;41(22):e111653. doi: 10.15252/embj.2022111653 (PMC9539236; doi:10.15252/embj.2022111653)
Supplement: Supplementary file 3 — Source Data for Expanded View and Appendix [file EMBJ-41-e111653-s005.zip › fig EV5/panel A C D/replicates.pdf]

EV11 quantification

EXPERIMENT 1

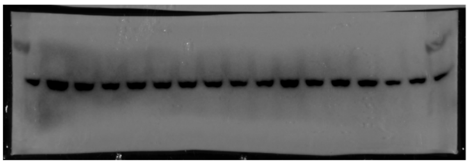

anti-actin

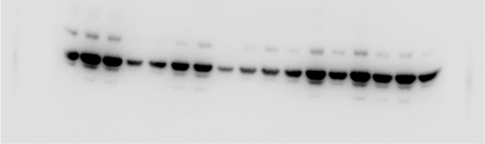

cyclin D3 (anti-HA)

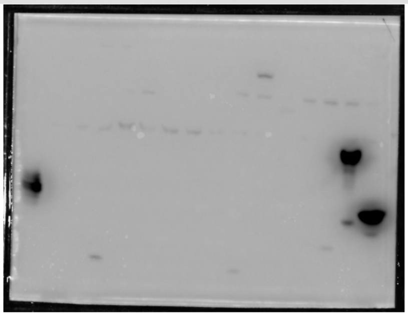

anti-strep tag

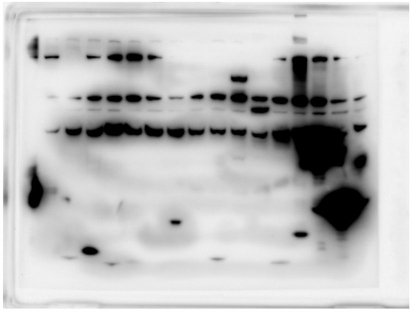

anti-strep tag longer exposure

EXPERIMENT 3

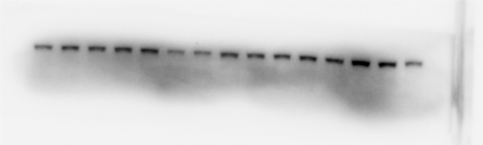

anti-actin

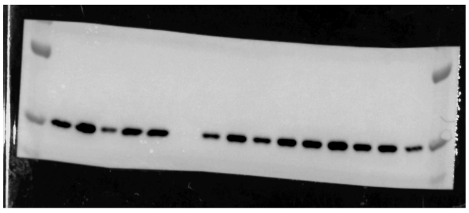

cyclin D3 (anti-HA)

EXPERIMENT 2

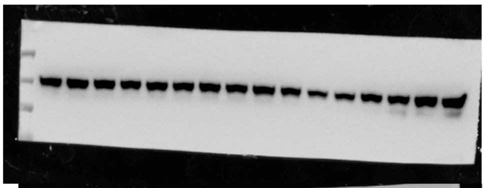

anti-actin

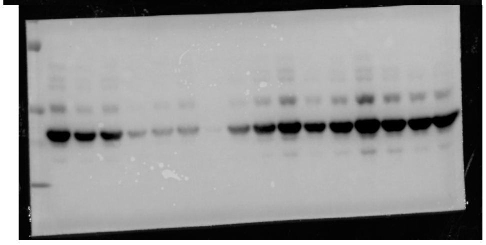

cyclin D3 (anti-HA)

EXPERIMENT 4,5

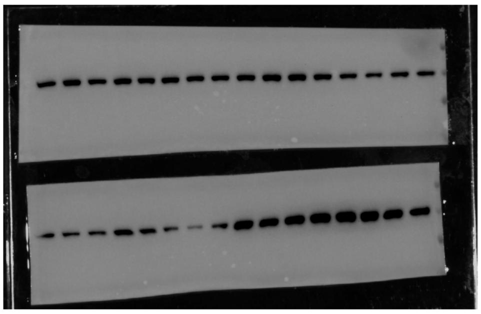

anti-actin

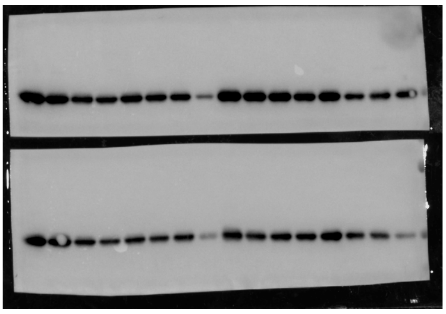

cyclin D3 (anti-HA)

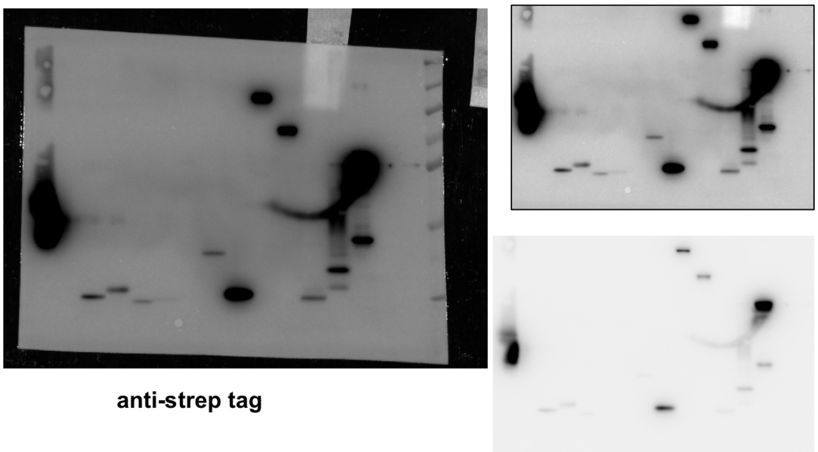

anti-strep tag

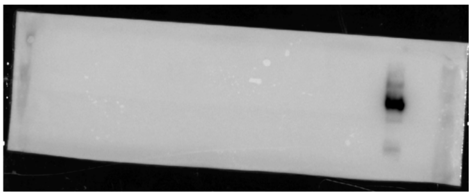

anti-spike
