# Supplementary figures and images for: Cyclin D3 restricts SARS‐CoV‐2 envelope incorporation into virions and interferes with viral spread
Source: EMBO J. 2022 Oct 10;41(22):e111653. doi: 10.15252/embj.2022111653 (PMC9539236; doi:10.15252/embj.2022111653)

EV 3A

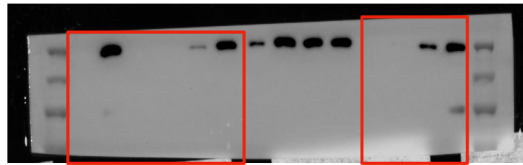

anti-Spike

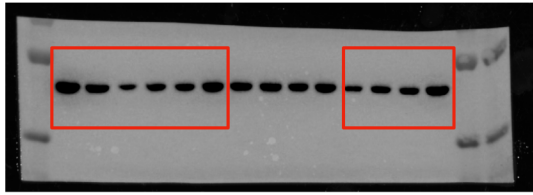

anti-actin

Supplement: Supplementary file 3 — Source Data for Expanded View and Appendix [file EMBJ-41-e111653-s005.zip › appendix/EMBOJ-2022-111653R-Figure_Appendix_S2_Source_Data-sd.pdf]

EV 7A

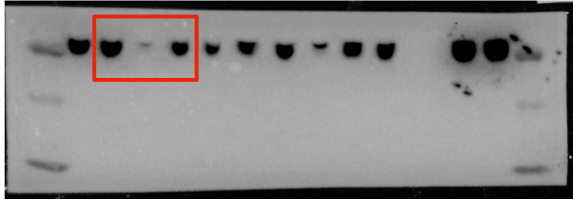

anti-cyclin D1

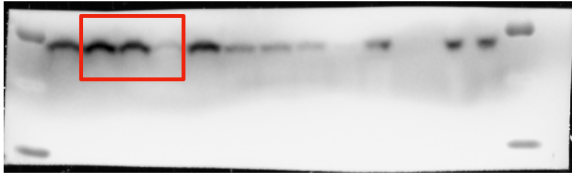

anti-cyclin D3

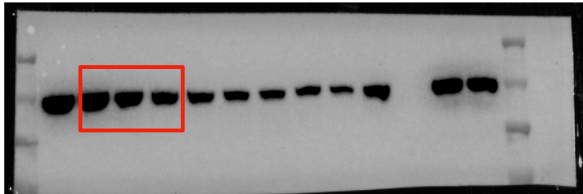

anti-actin

Supplement: Supplementary file 3 — Source Data for Expanded View and Appendix [file EMBJ-41-e111653-s005.zip › appendix/EMBOJ-2022-111653R-Figure_Appendix_S4_Source_Data-sd.pdf]

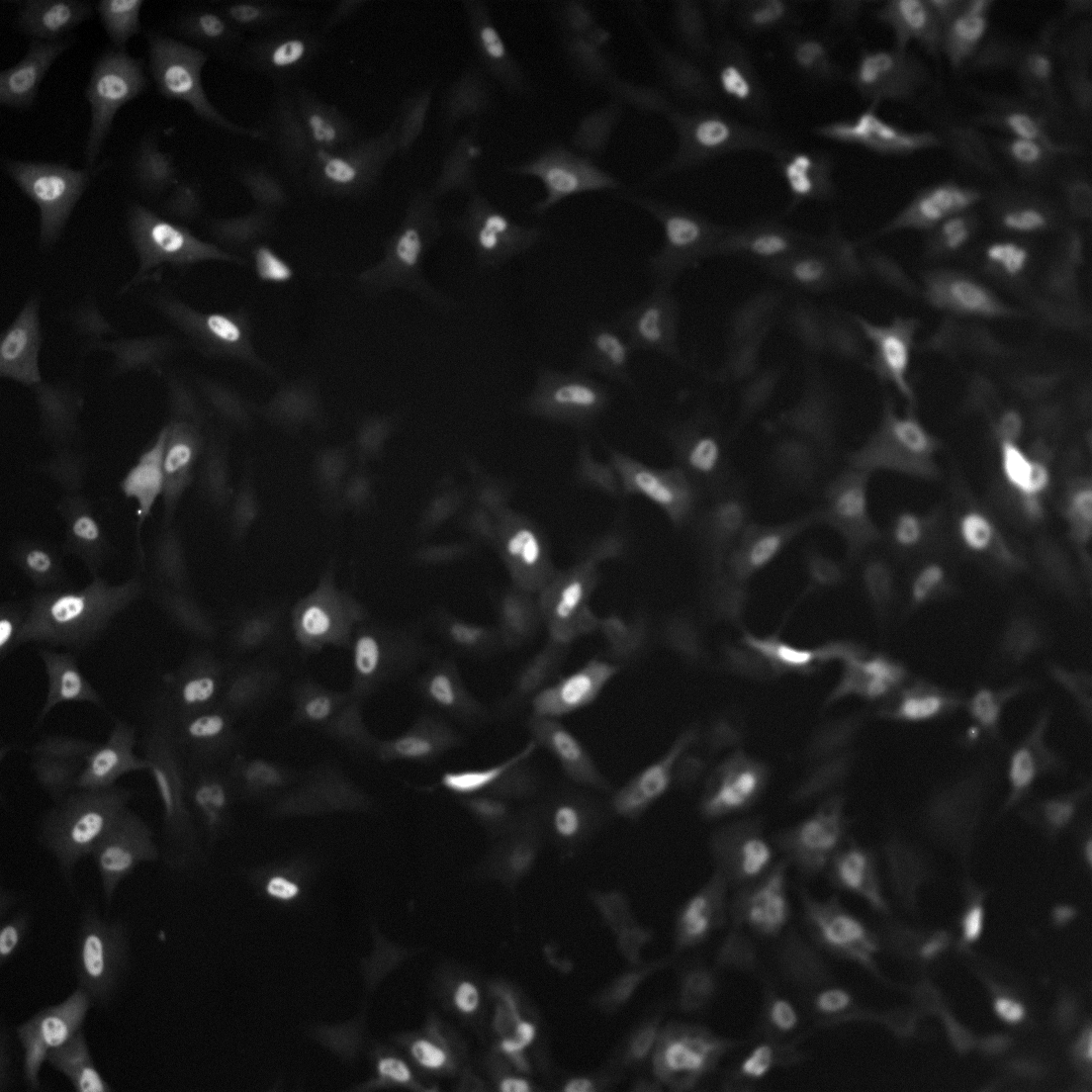

Supplement: Supplementary file 3 — Source Data for Expanded View and Appendix [file EMBJ-41-e111653-s005.zip › fig EV1/panel C/cyclin A2/cyclin A2.TIFF]

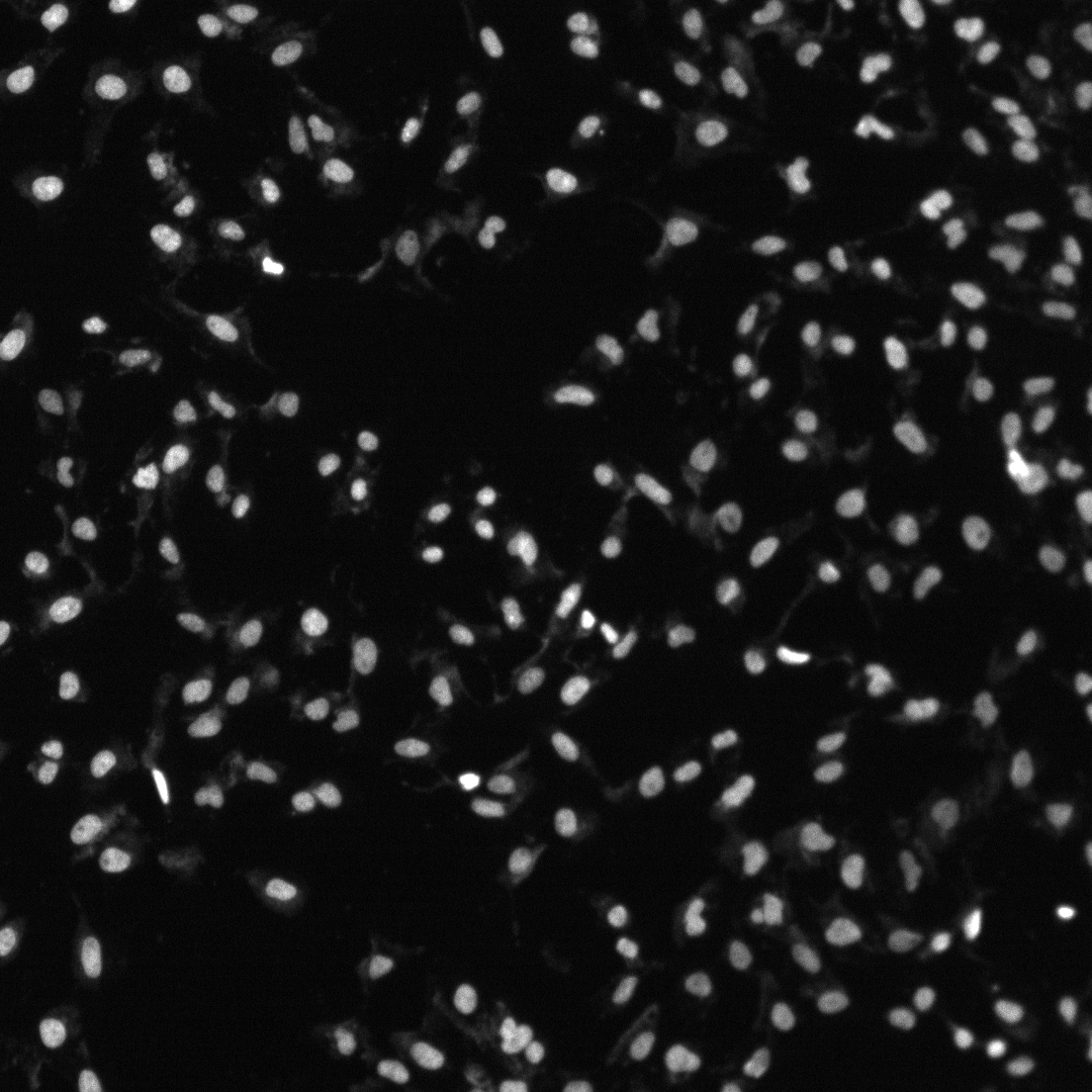

Supplement: Supplementary file 3 — Source Data for Expanded View and Appendix [file EMBJ-41-e111653-s005.zip › fig EV1/panel C/cyclin A2/nuclei.TIFF]

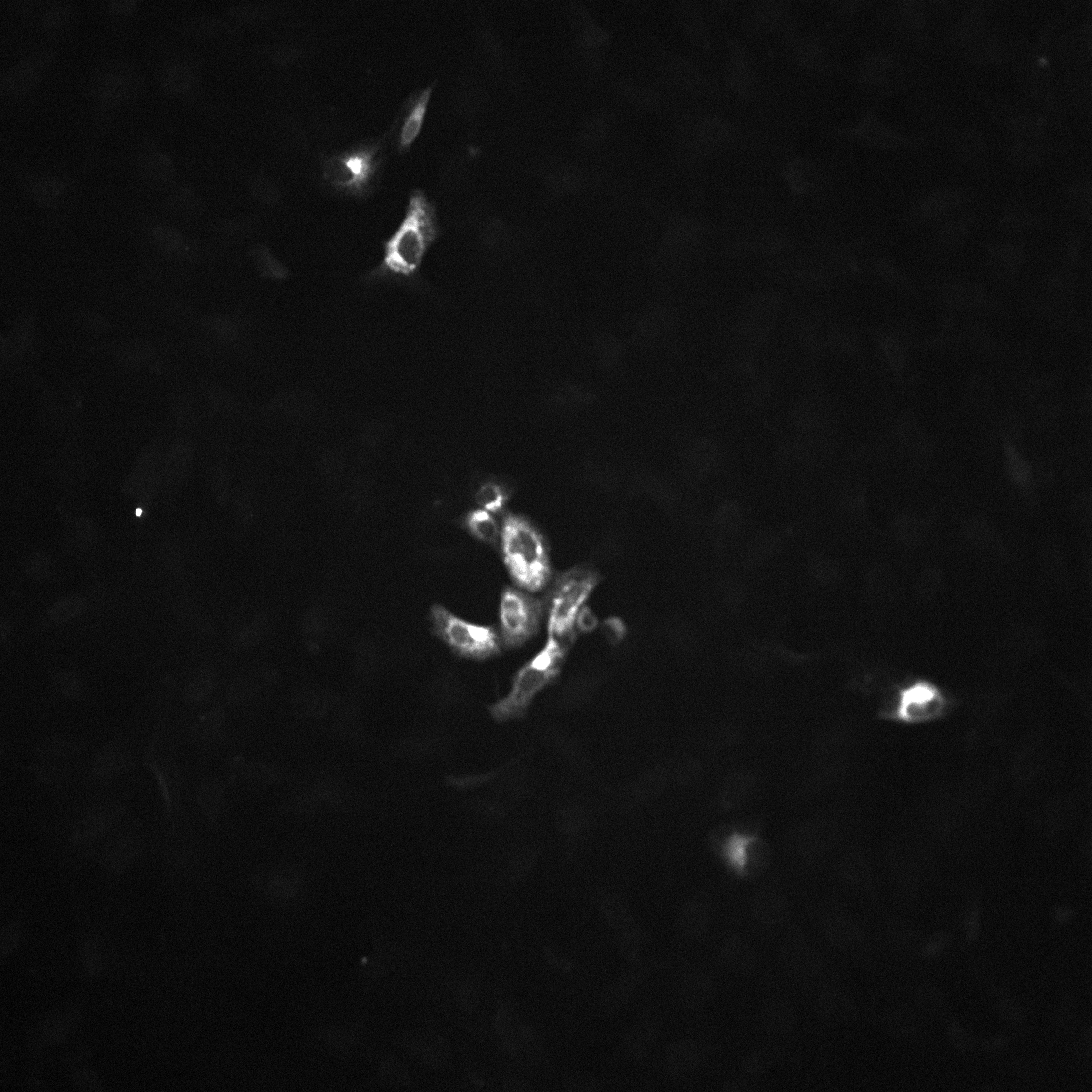

Supplement: Supplementary file 3 — Source Data for Expanded View and Appendix [file EMBJ-41-e111653-s005.zip › fig EV1/panel C/cyclin A2/SARS Spike.TIFF]

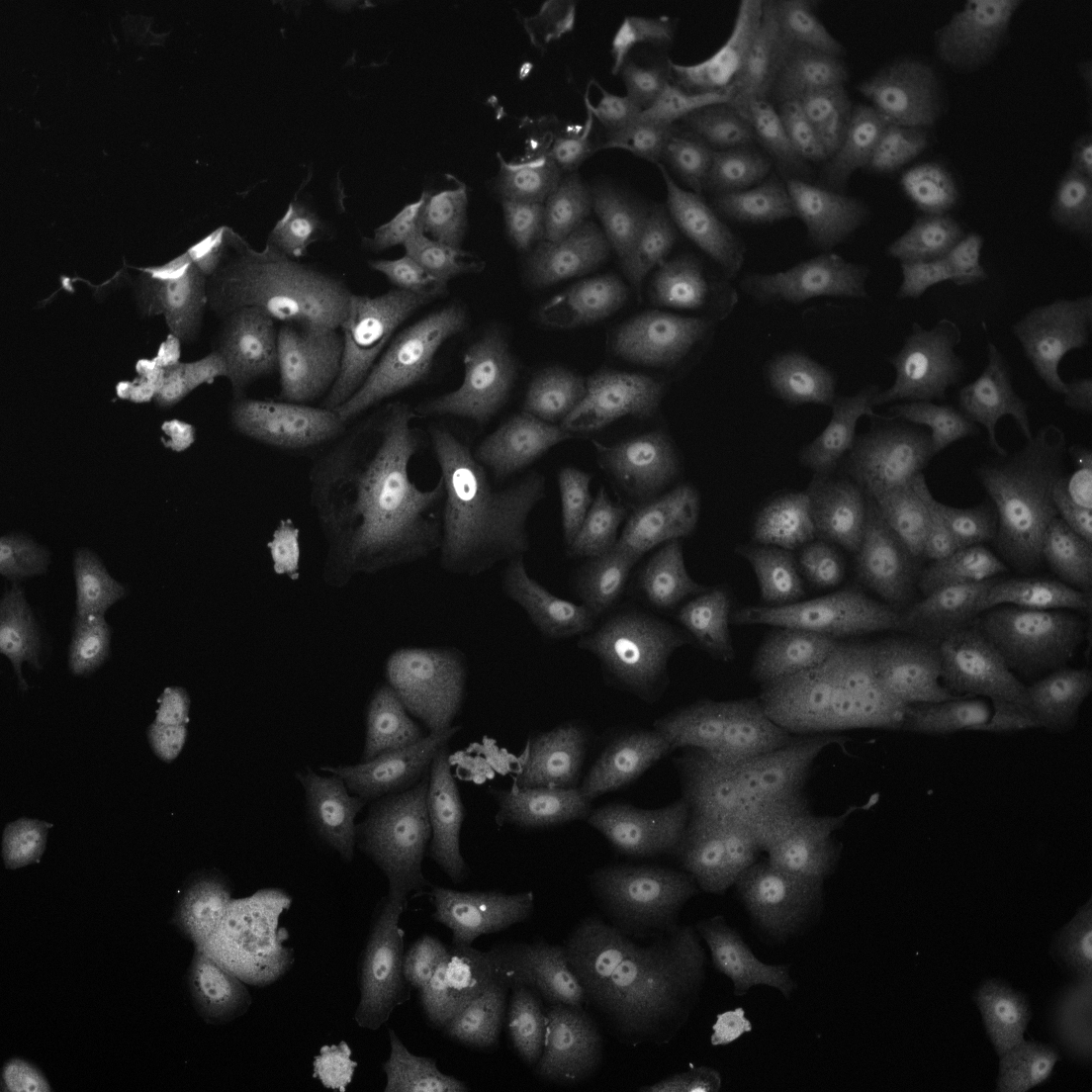

Supplement: Supplementary file 3 — Source Data for Expanded View and Appendix [file EMBJ-41-e111653-s005.zip › fig EV1/panel C/cyclin D1/cyclin D1.TIFF]

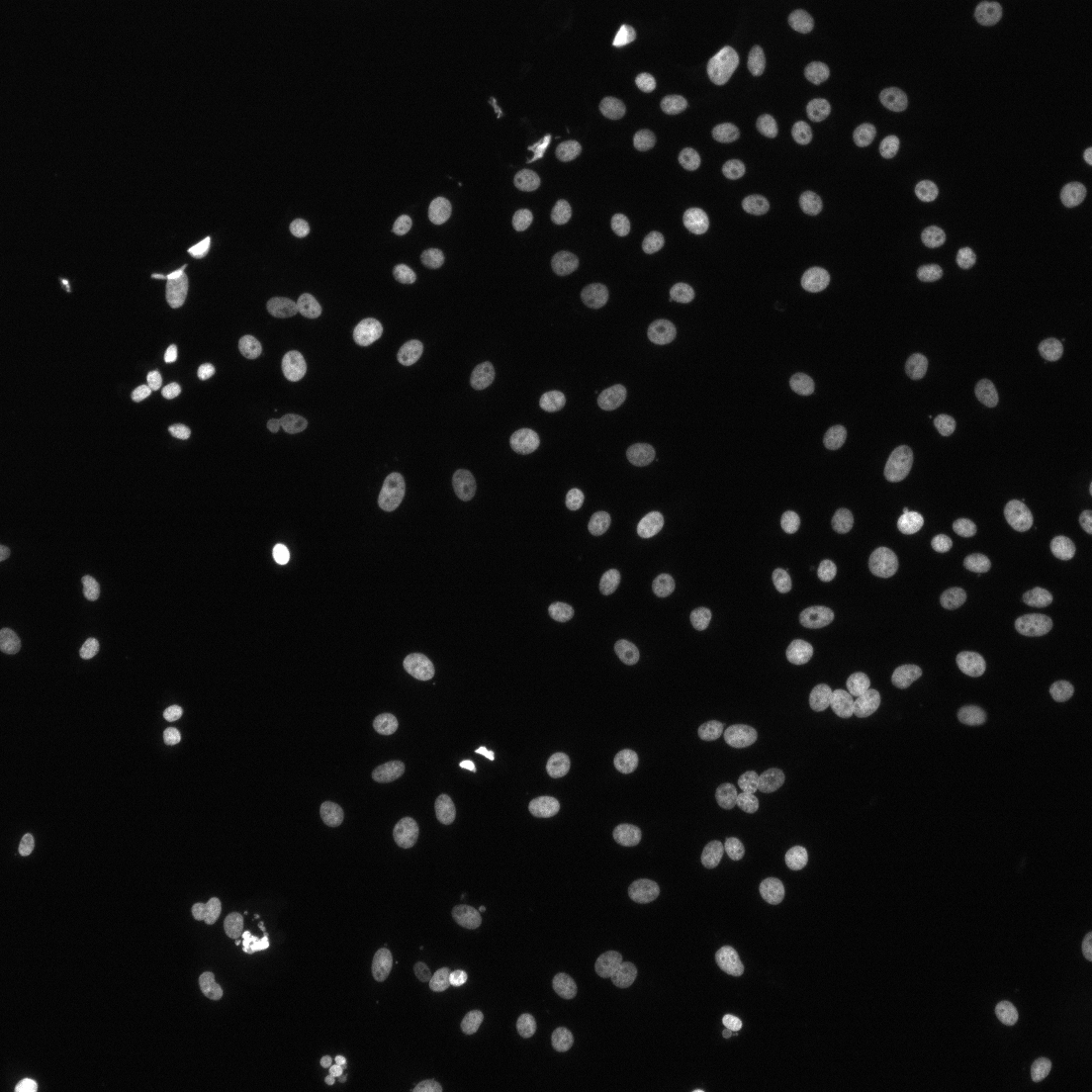

Supplement: Supplementary file 3 — Source Data for Expanded View and Appendix [file EMBJ-41-e111653-s005.zip › fig EV1/panel C/cyclin D1/nuclei.TIFF]

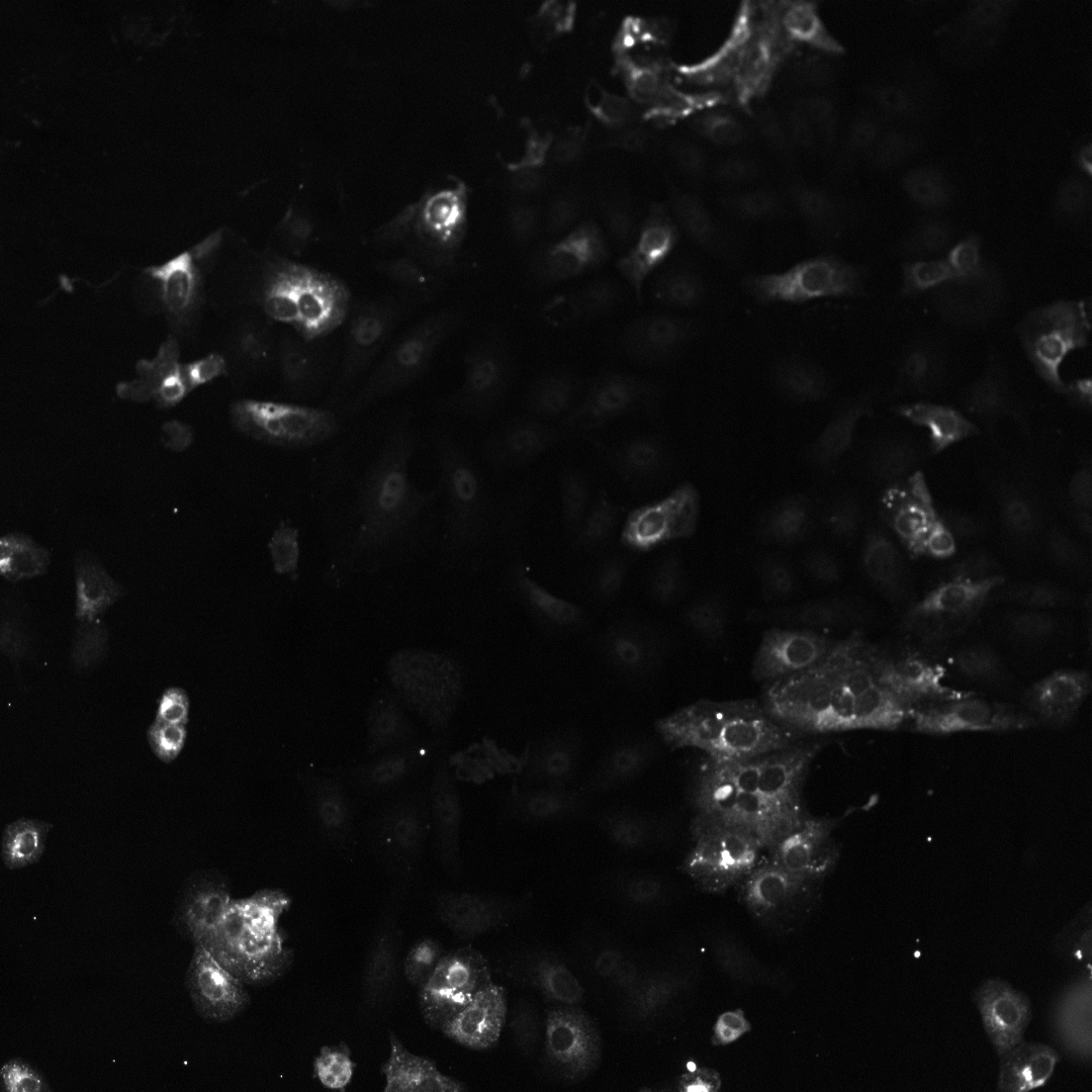

Supplement: Supplementary file 3 — Source Data for Expanded View and Appendix [file EMBJ-41-e111653-s005.zip › fig EV1/panel C/cyclin D1/SARS Spike.TIFF]

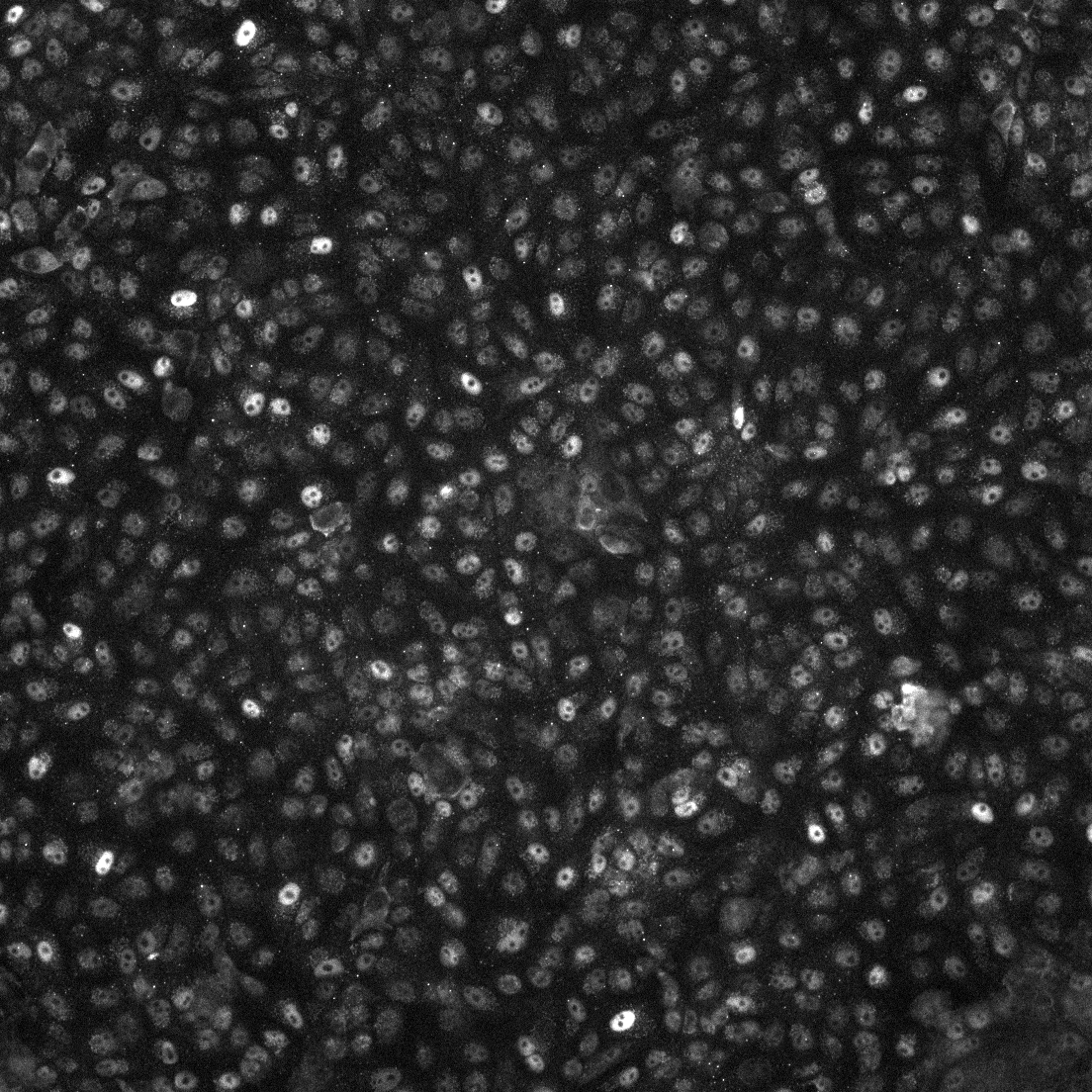

Supplement: Supplementary file 3 — Source Data for Expanded View and Appendix [file EMBJ-41-e111653-s005.zip › fig EV1/panel C/cyclin D3/cyclin D3.TIFF]

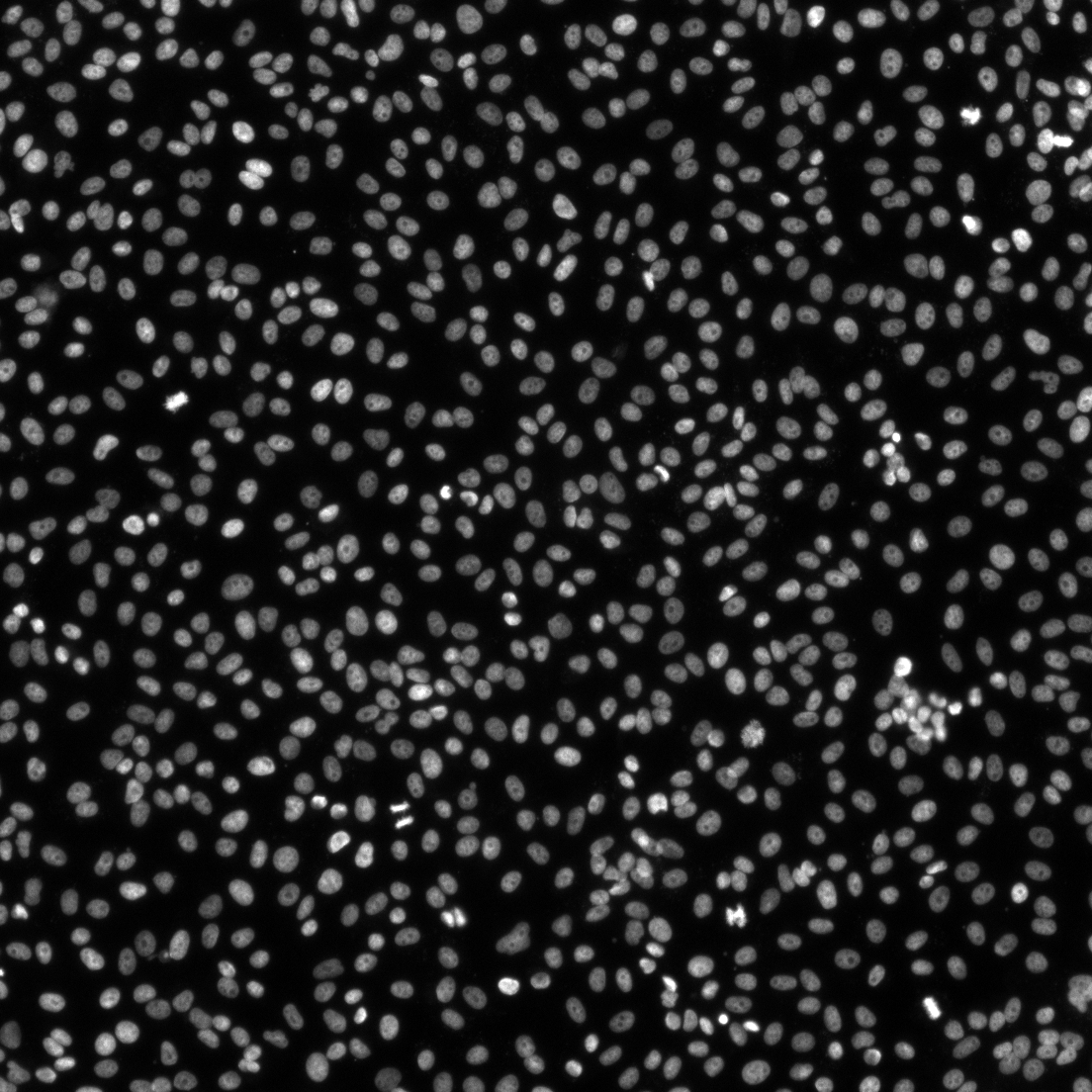

Supplement: Supplementary file 3 — Source Data for Expanded View and Appendix [file EMBJ-41-e111653-s005.zip › fig EV1/panel C/cyclin D3/nuclei.TIFF]

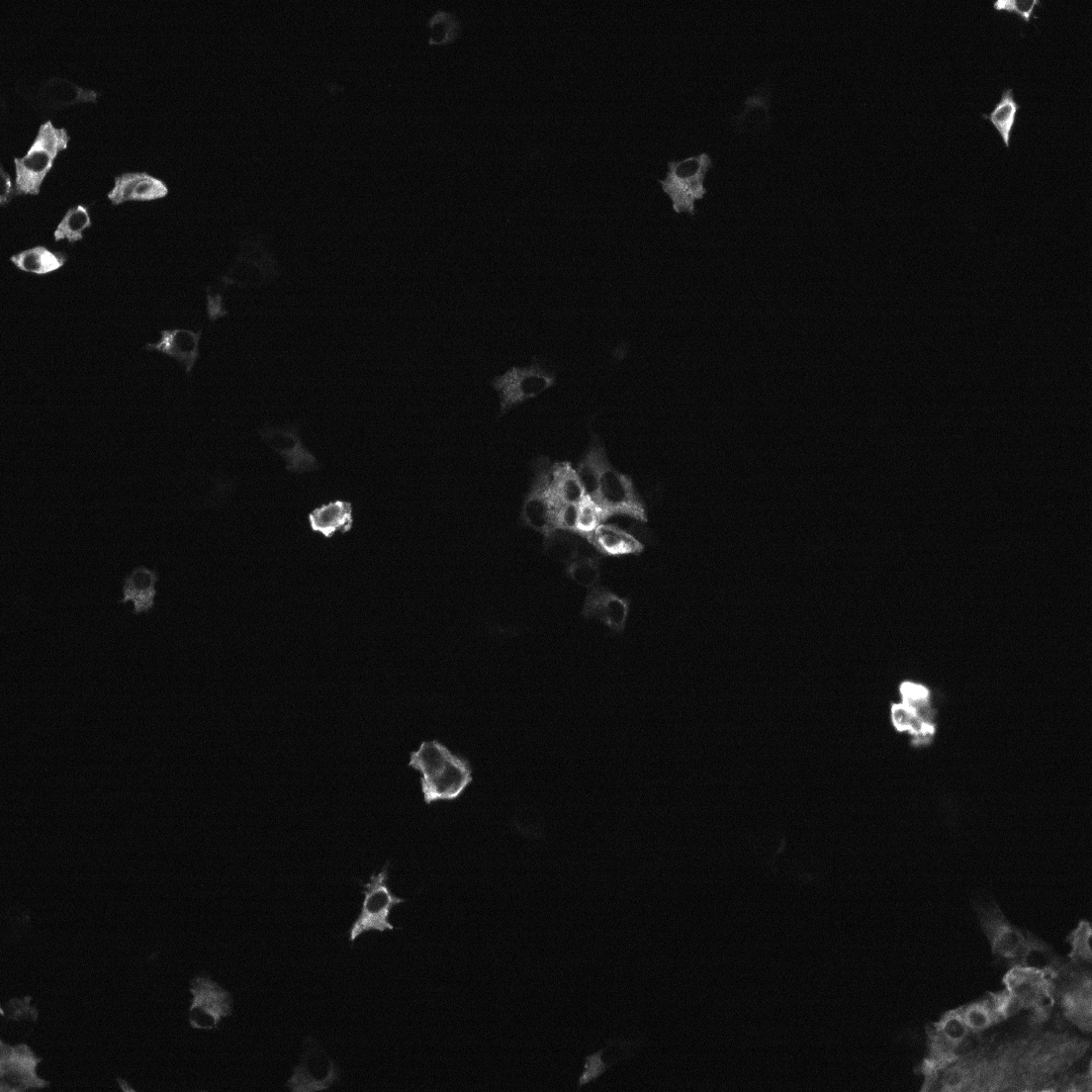

Supplement: Supplementary file 3 — Source Data for Expanded View and Appendix [file EMBJ-41-e111653-s005.zip › fig EV1/panel C/cyclin D3/SARS N.TIFF]

EV1D

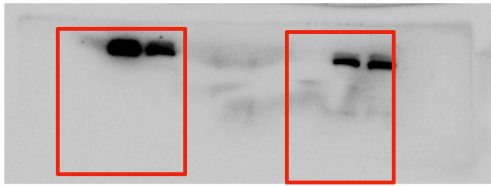

anti-Spike

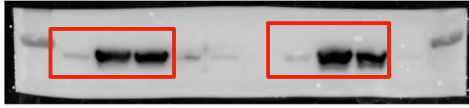

anti-nucleocapsid

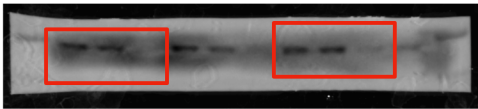

anti-cyclin D3

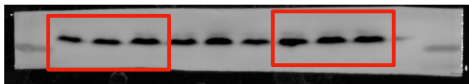

anti-actin

Supplement: Supplementary file 3 — Source Data for Expanded View and Appendix [file EMBJ-41-e111653-s005.zip › fig EV1/panel D/EMBOJ-2022-111653R-Figure_EV1_Source_Data-sd.pdf]

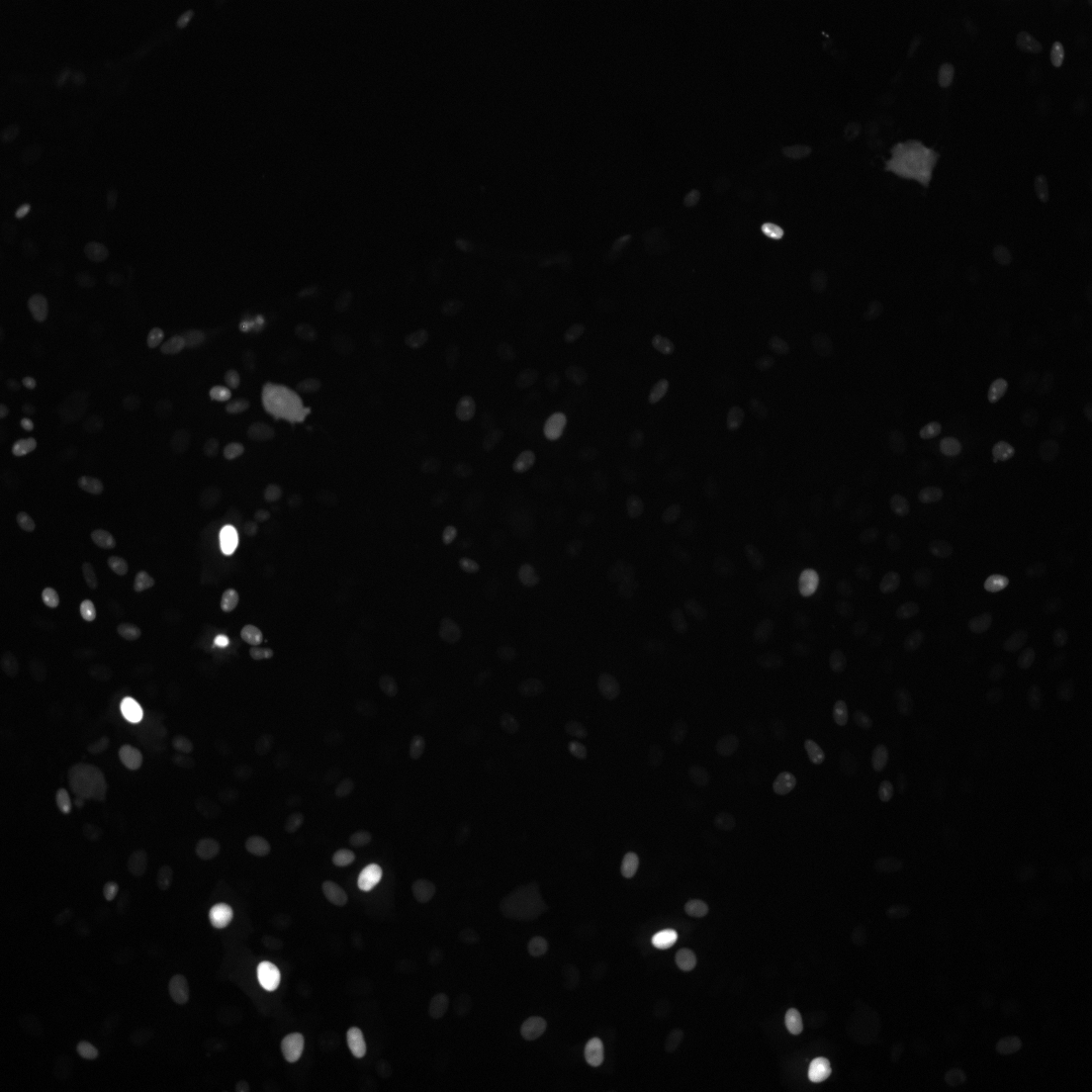

Supplement: Supplementary file 3 — Source Data for Expanded View and Appendix [file EMBJ-41-e111653-s005.zip › fig EV2/panel C/cdt1.TIFF]

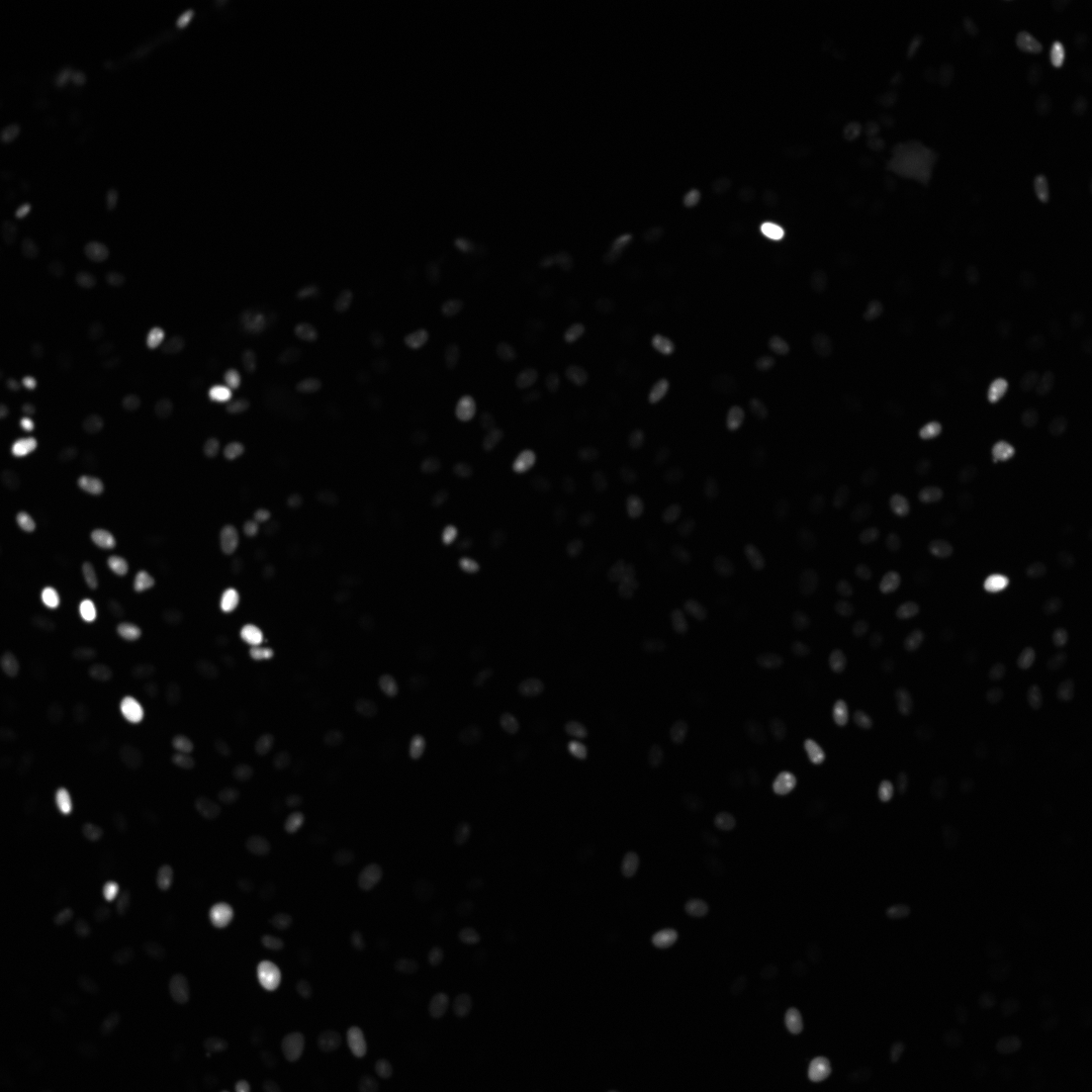

Supplement: Supplementary file 3 — Source Data for Expanded View and Appendix [file EMBJ-41-e111653-s005.zip › fig EV2/panel C/geminin.TIFF]

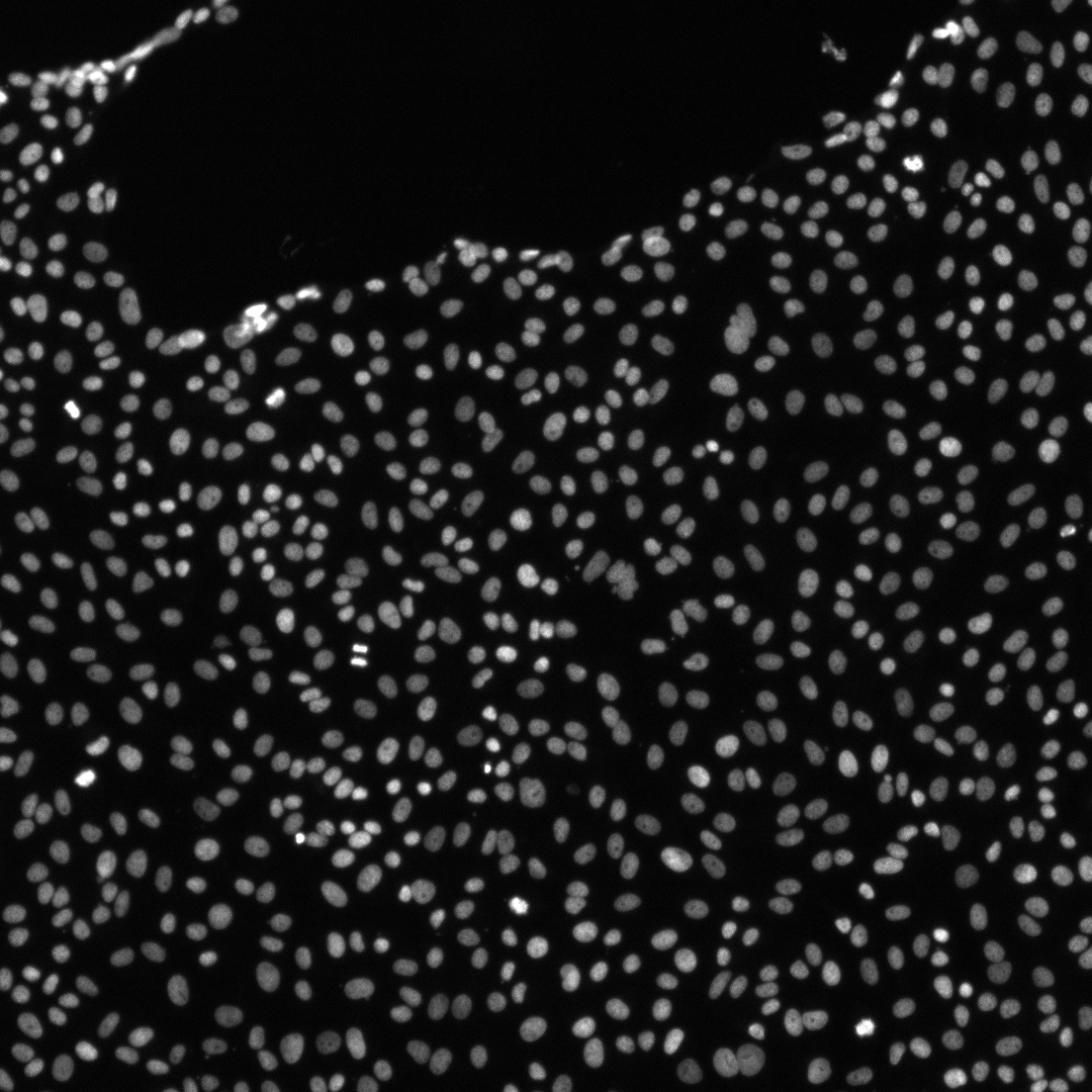

Supplement: Supplementary file 3 — Source Data for Expanded View and Appendix [file EMBJ-41-e111653-s005.zip › fig EV2/panel C/nuclei.TIFF]

EV 2

EV 2B

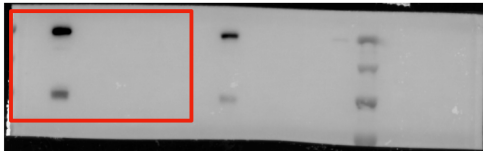

anti-Spike

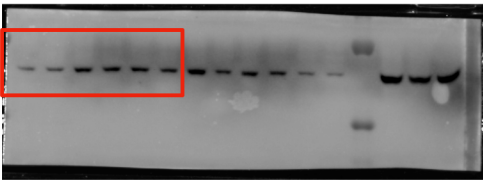

anti-actin

EV 2D

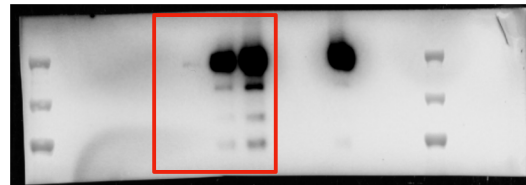

anti-Spike

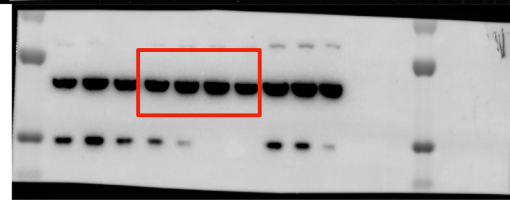

anti-actin

Supplement: Supplementary file 3 — Source Data for Expanded View and Appendix [file EMBJ-41-e111653-s005.zip › fig EV2/panel E G/EMBOJ-2022-111653R-Figure_EV2_Source_Data-sd.pdf]

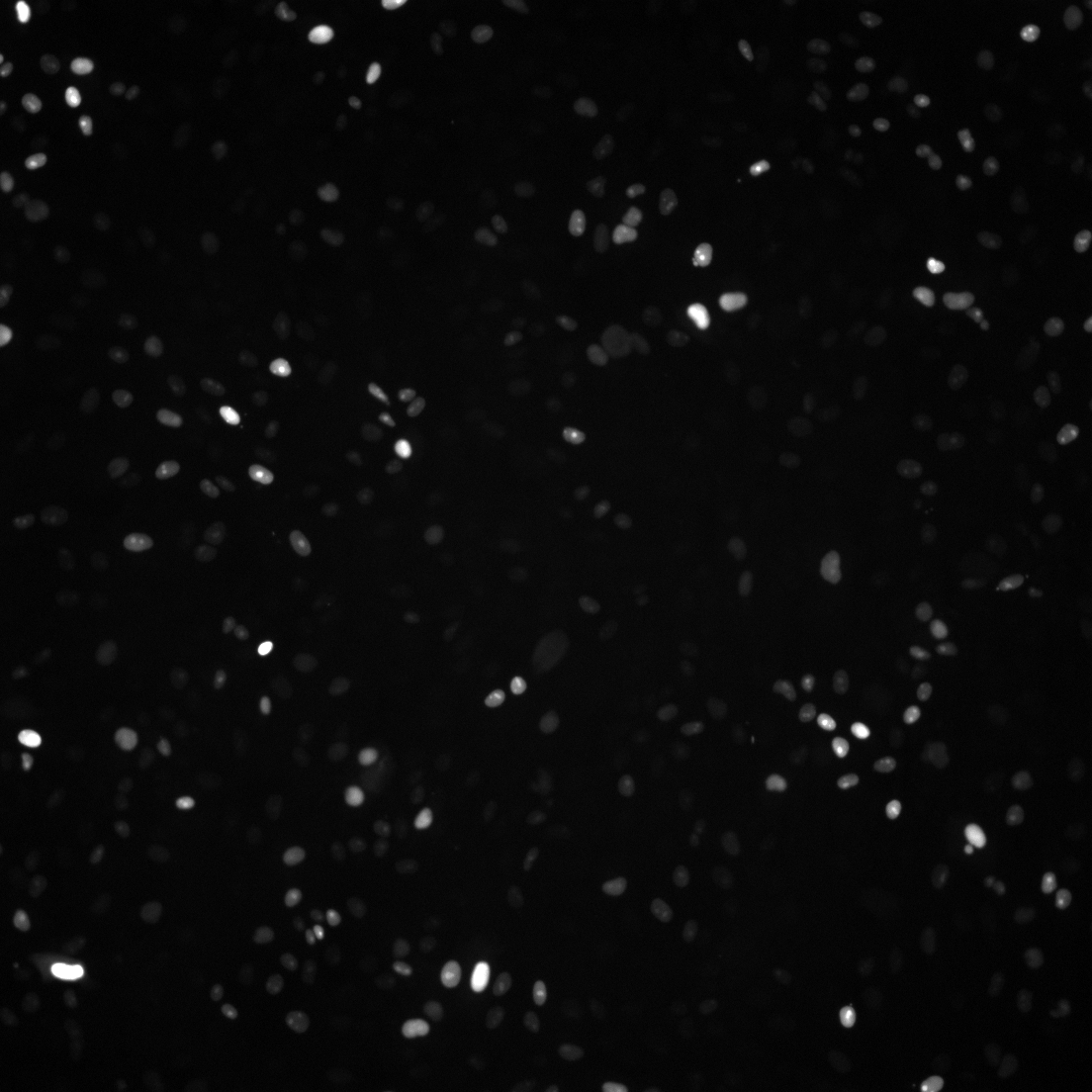

Supplement: Supplementary file 3 — Source Data for Expanded View and Appendix [file EMBJ-41-e111653-s005.zip › fig EV3/panel G/Cdt1.TIFF]

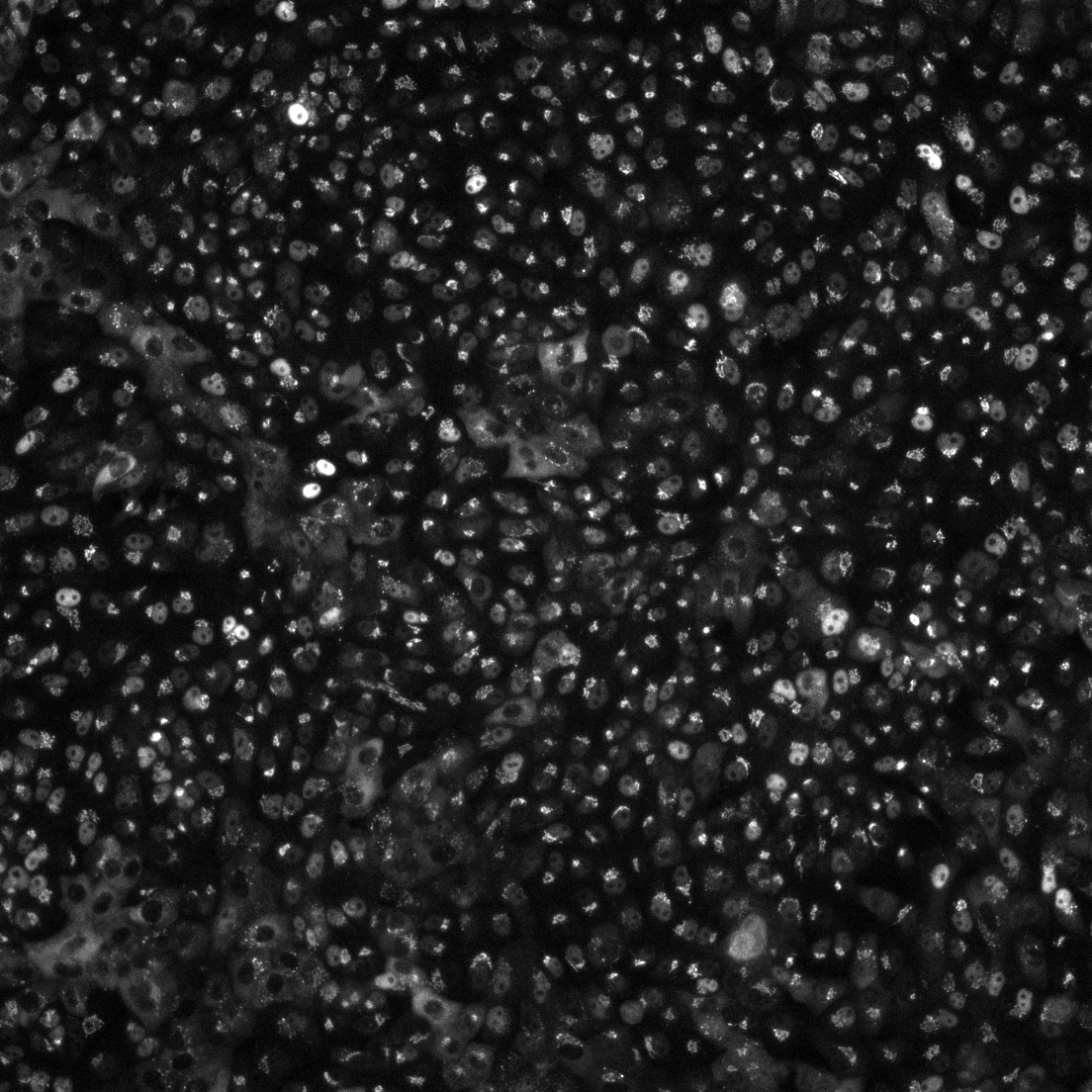

Supplement: Supplementary file 3 — Source Data for Expanded View and Appendix [file EMBJ-41-e111653-s005.zip › fig EV3/panel G/cyclin D3.TIFF]

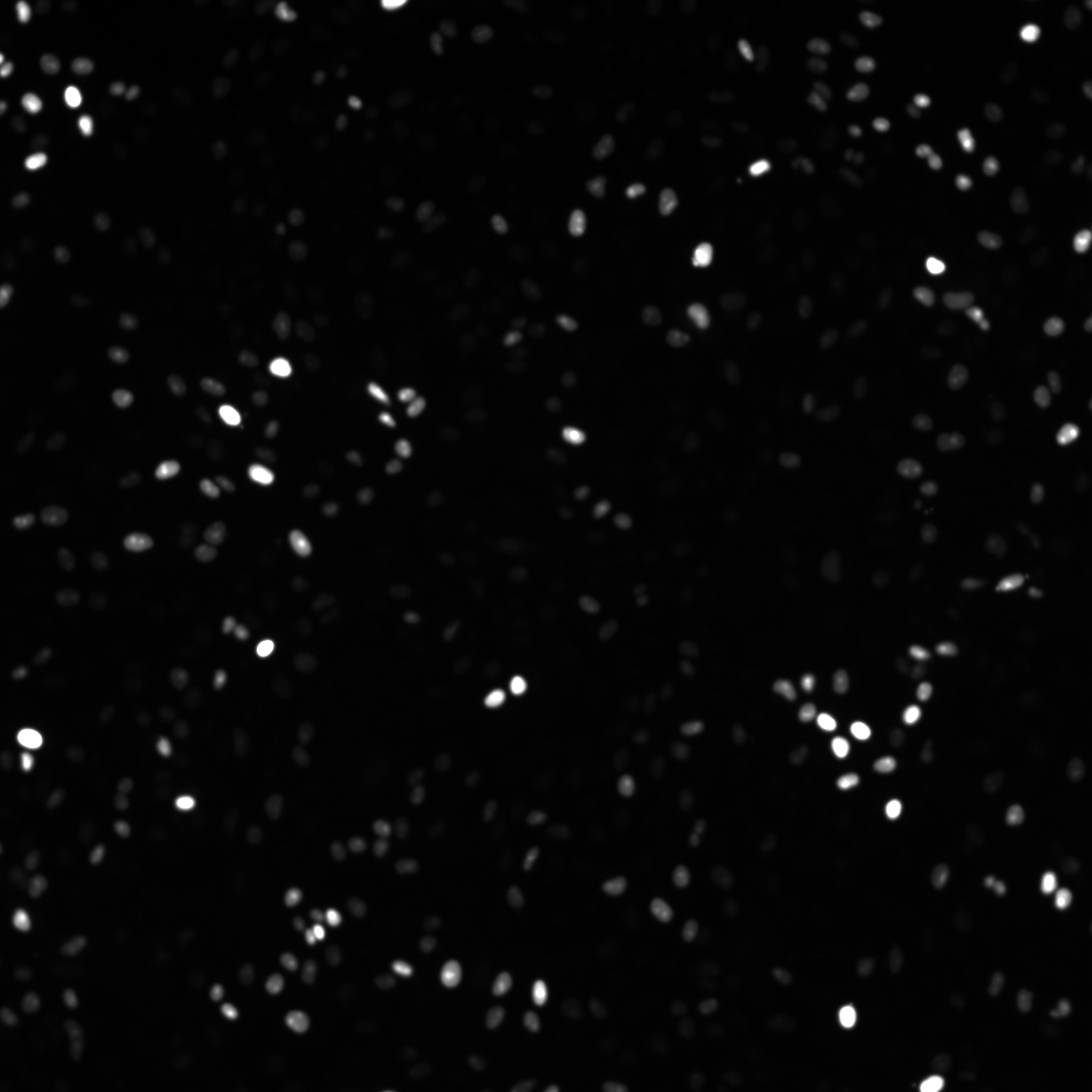

Supplement: Supplementary file 3 — Source Data for Expanded View and Appendix [file EMBJ-41-e111653-s005.zip › fig EV3/panel G/geminin.TIFF]

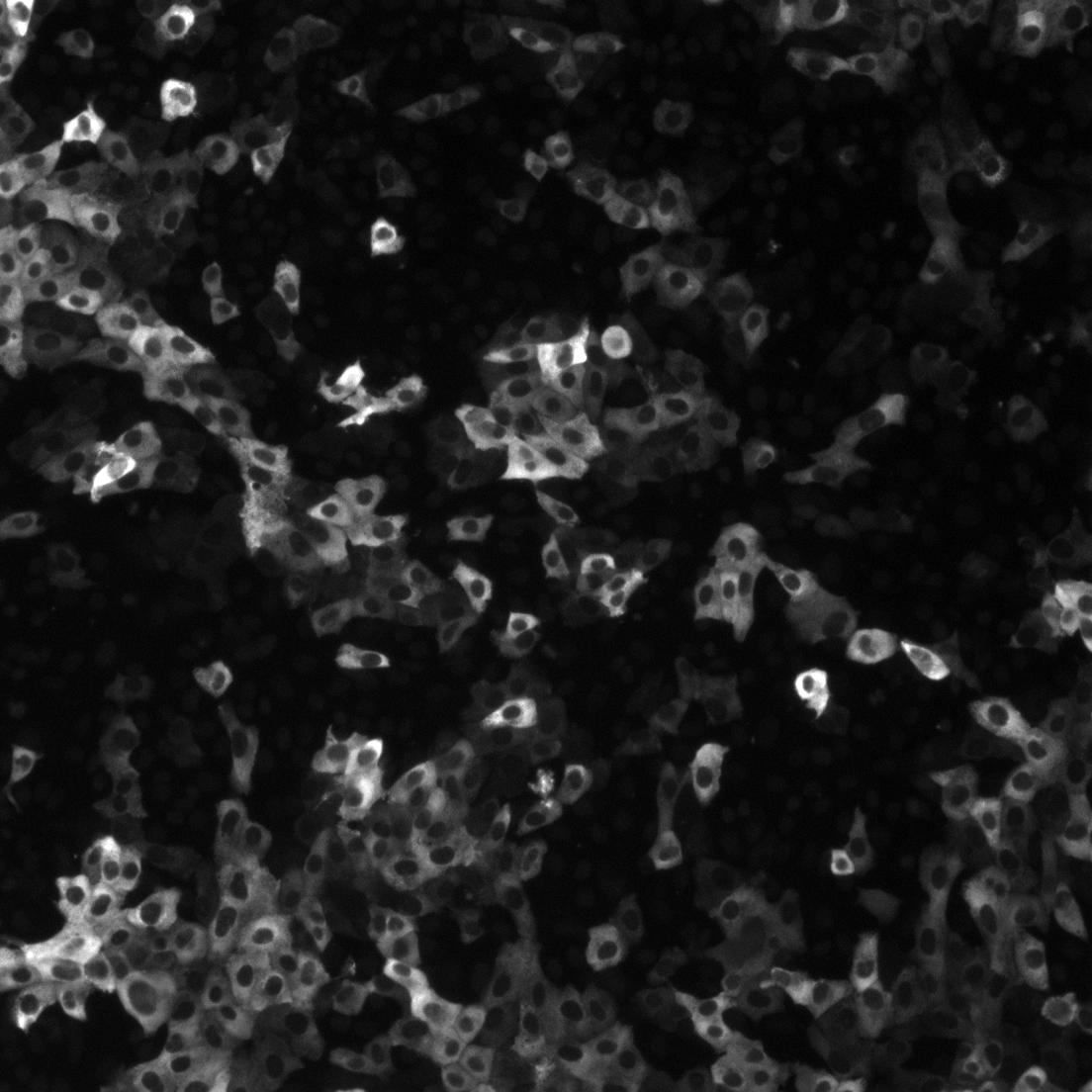

Supplement: Supplementary file 3 — Source Data for Expanded View and Appendix [file EMBJ-41-e111653-s005.zip › fig EV3/panel G/SARS N.TIFF]

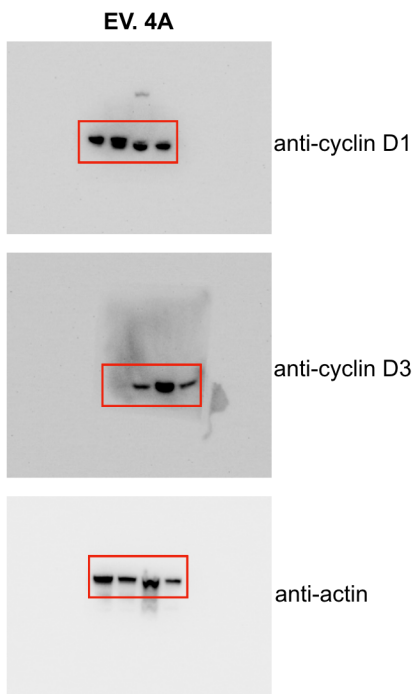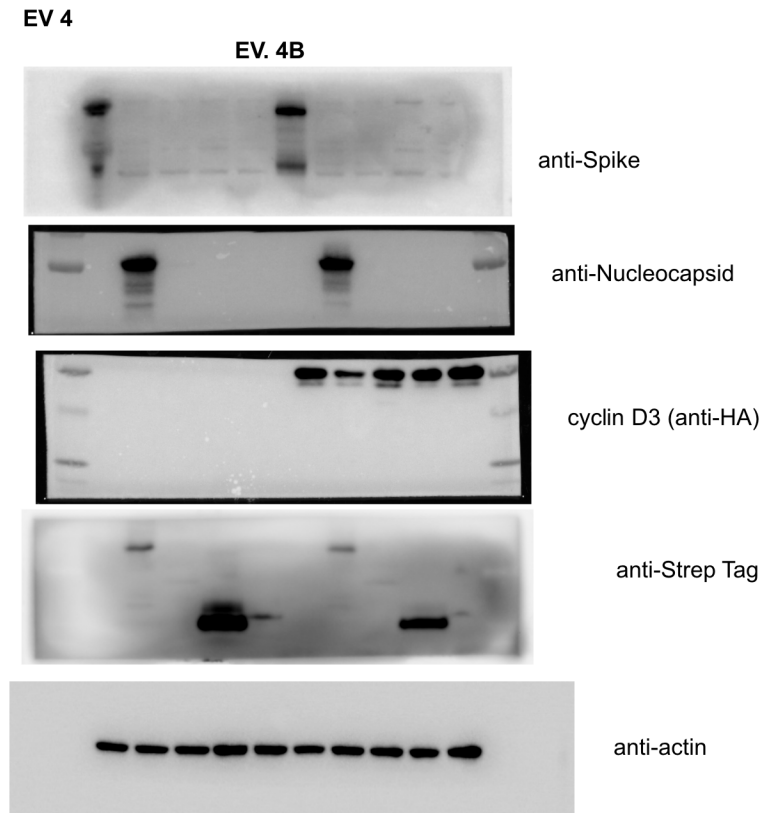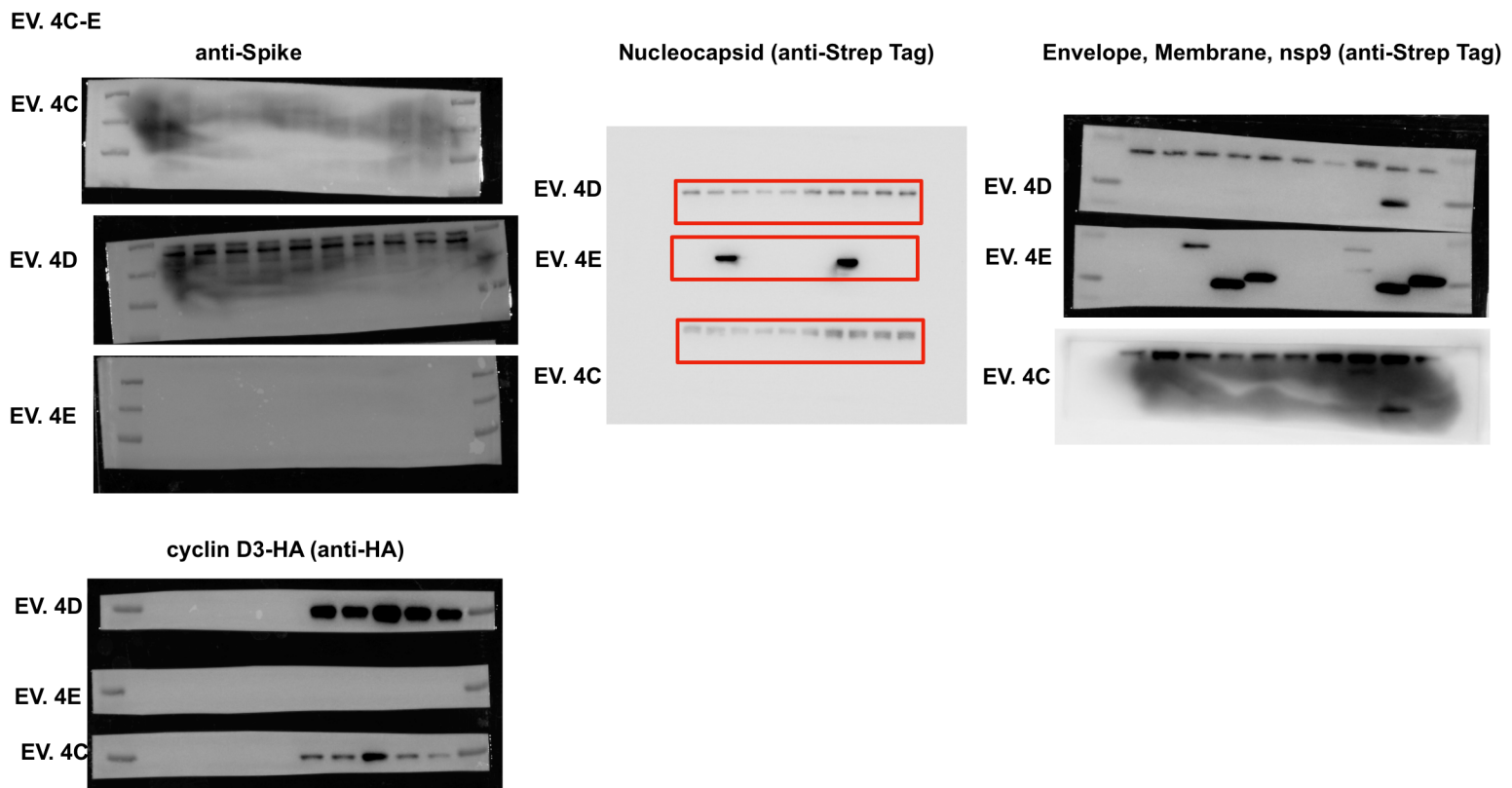

Supplement: Supplementary file 3 — Source Data for Expanded View and Appendix [file EMBJ-41-e111653-s005.zip › fig EV4/panel A B C E/EMBOJ-2022-111653R-Figure_EV4_Source_Data-sd.pdf]

Figure 1

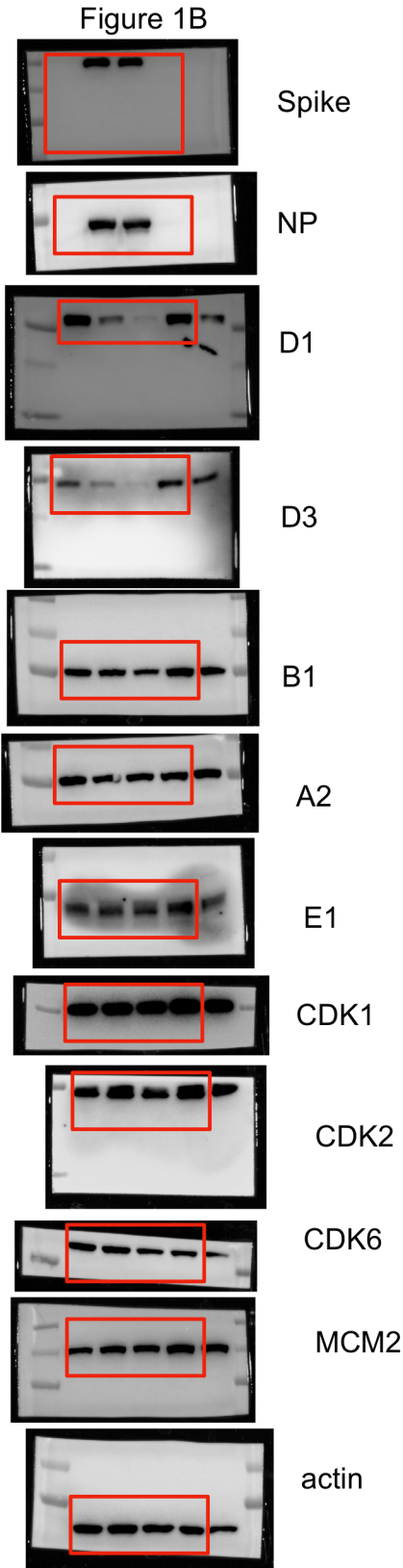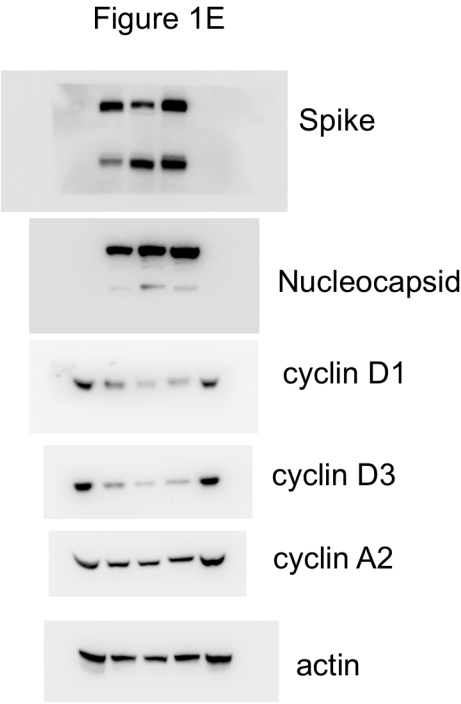

Supplement: Supplementary file 4 — Source Data for Figure 1 [file EMBJ-41-e111653-s006.zip › panel B E/EMBOJ-2022-111653R-Figure_1_Source_Data-sd.pdf]

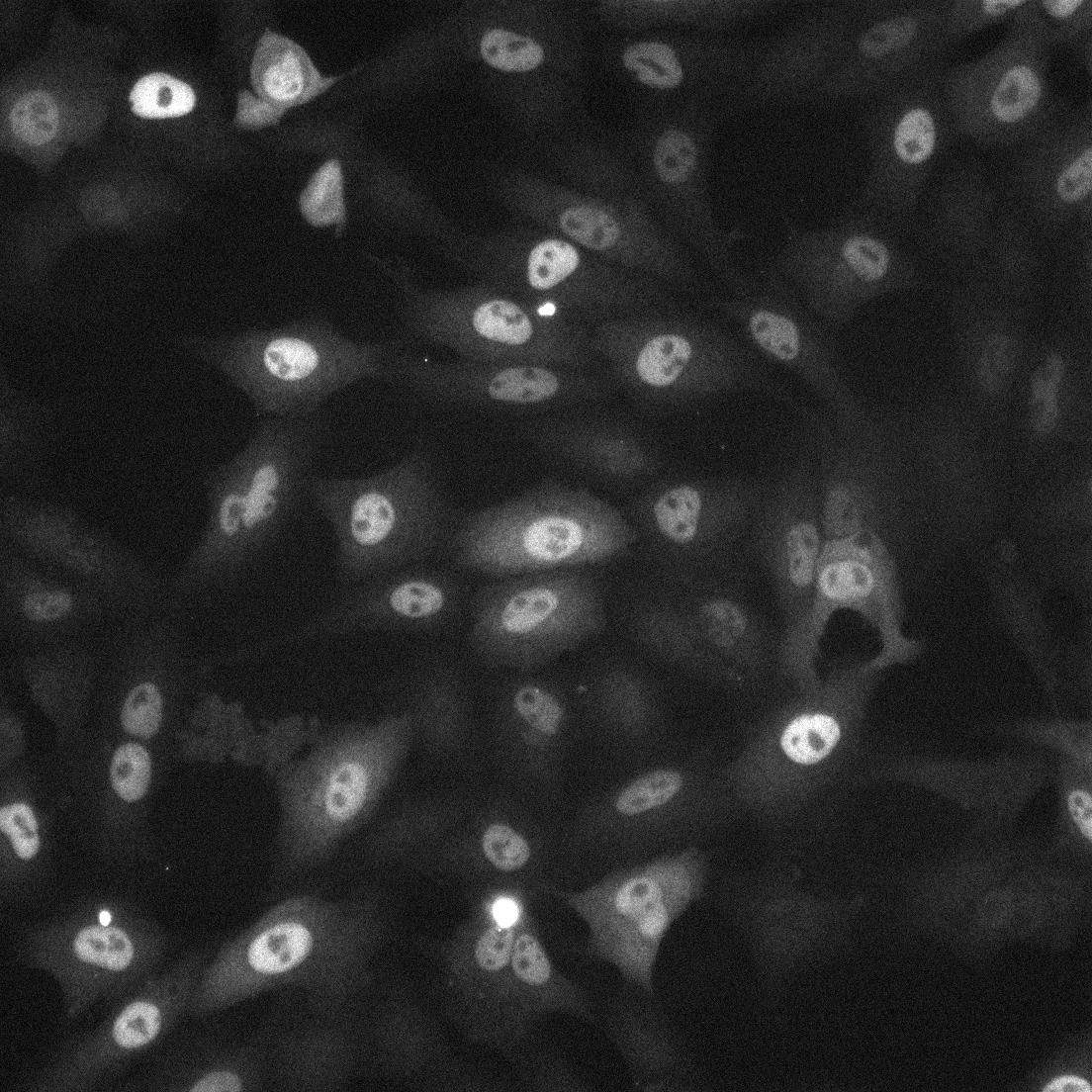

Supplement: Supplementary file 4 — Source Data for Figure 1 [file EMBJ-41-e111653-s006.zip › panel C/cyclin A2/cyclin A2.TIFF]

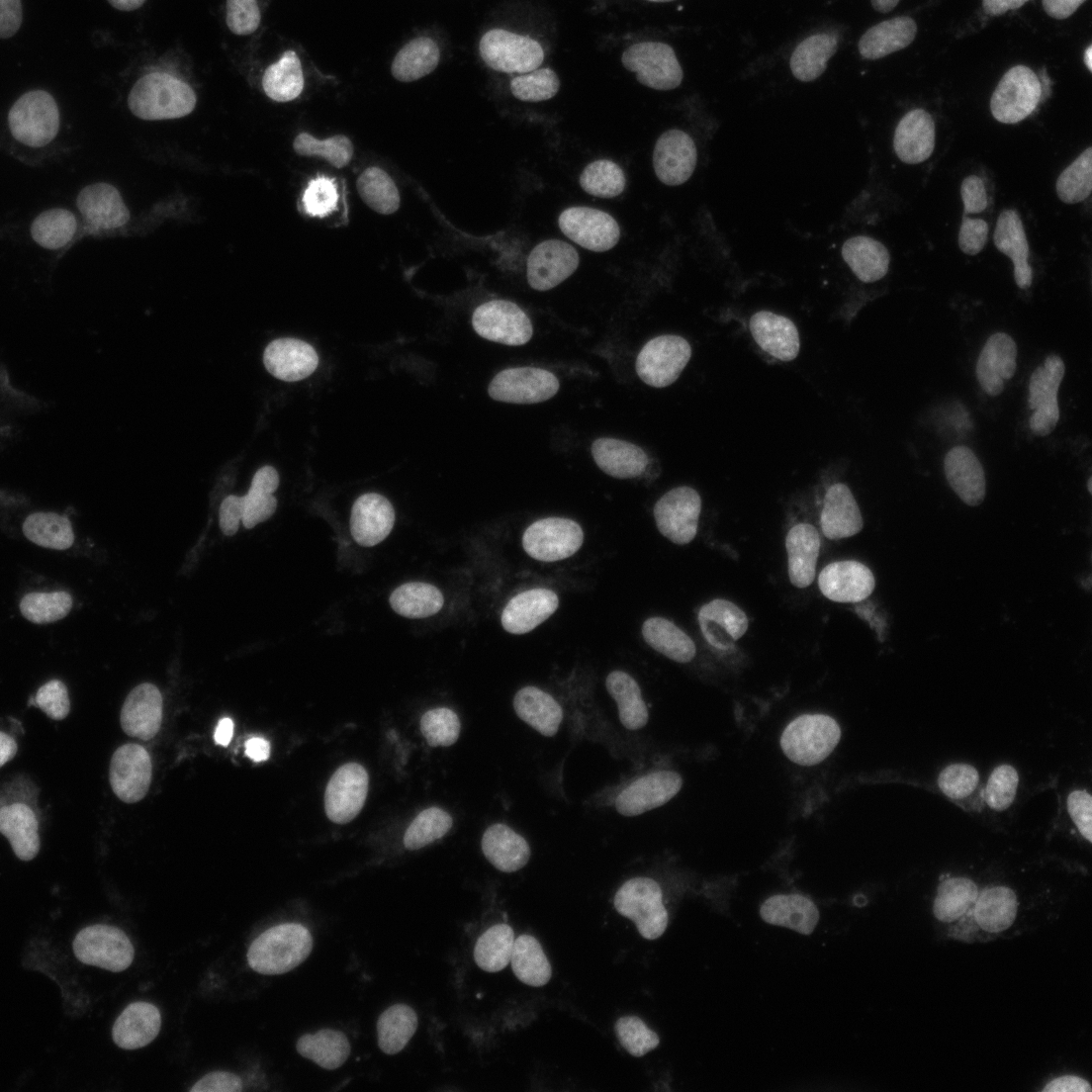

Supplement: Supplementary file 4 — Source Data for Figure 1 [file EMBJ-41-e111653-s006.zip › panel C/cyclin A2/nuclei.TIFF]

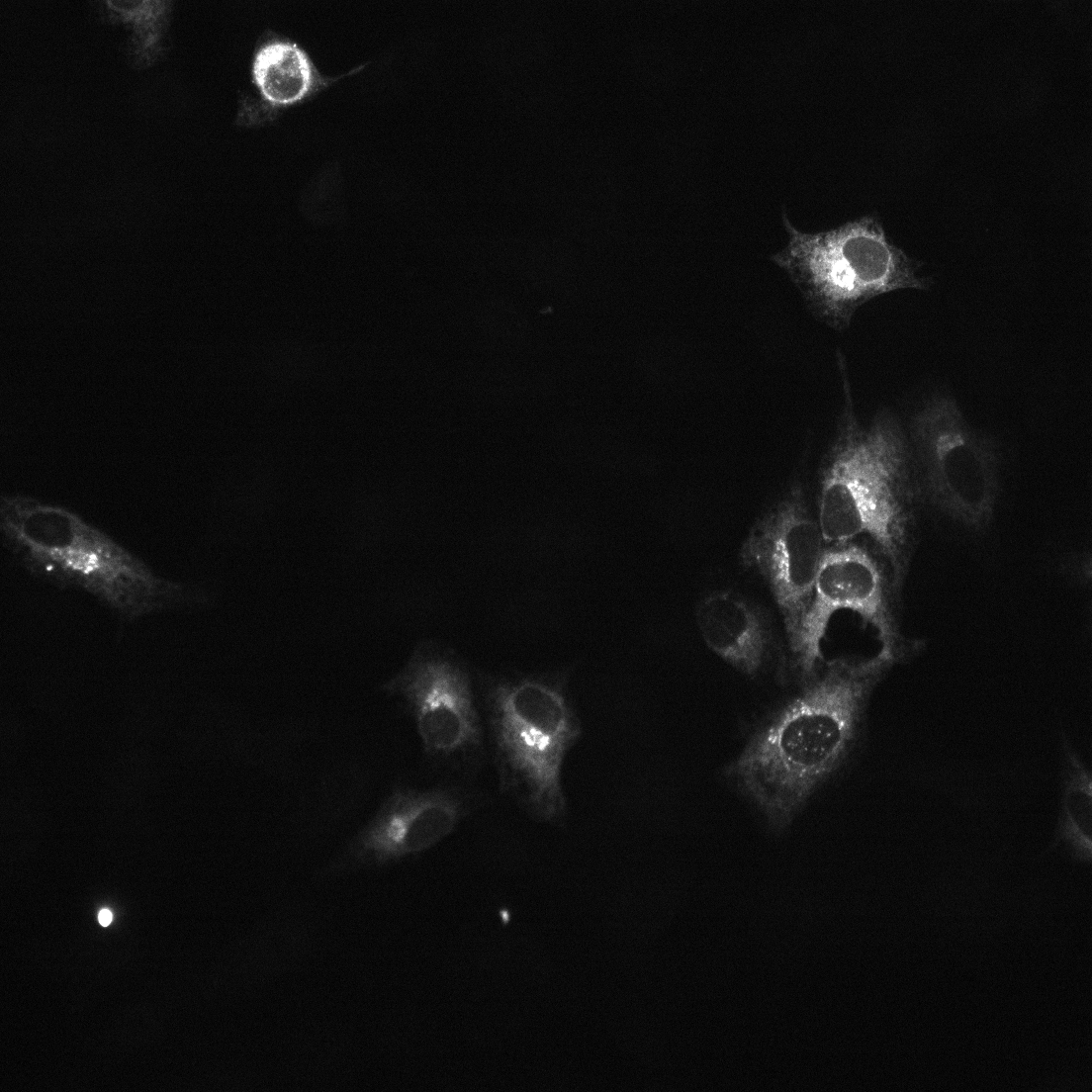

Supplement: Supplementary file 4 — Source Data for Figure 1 [file EMBJ-41-e111653-s006.zip › panel C/cyclin A2/SARS Spike.TIFF]

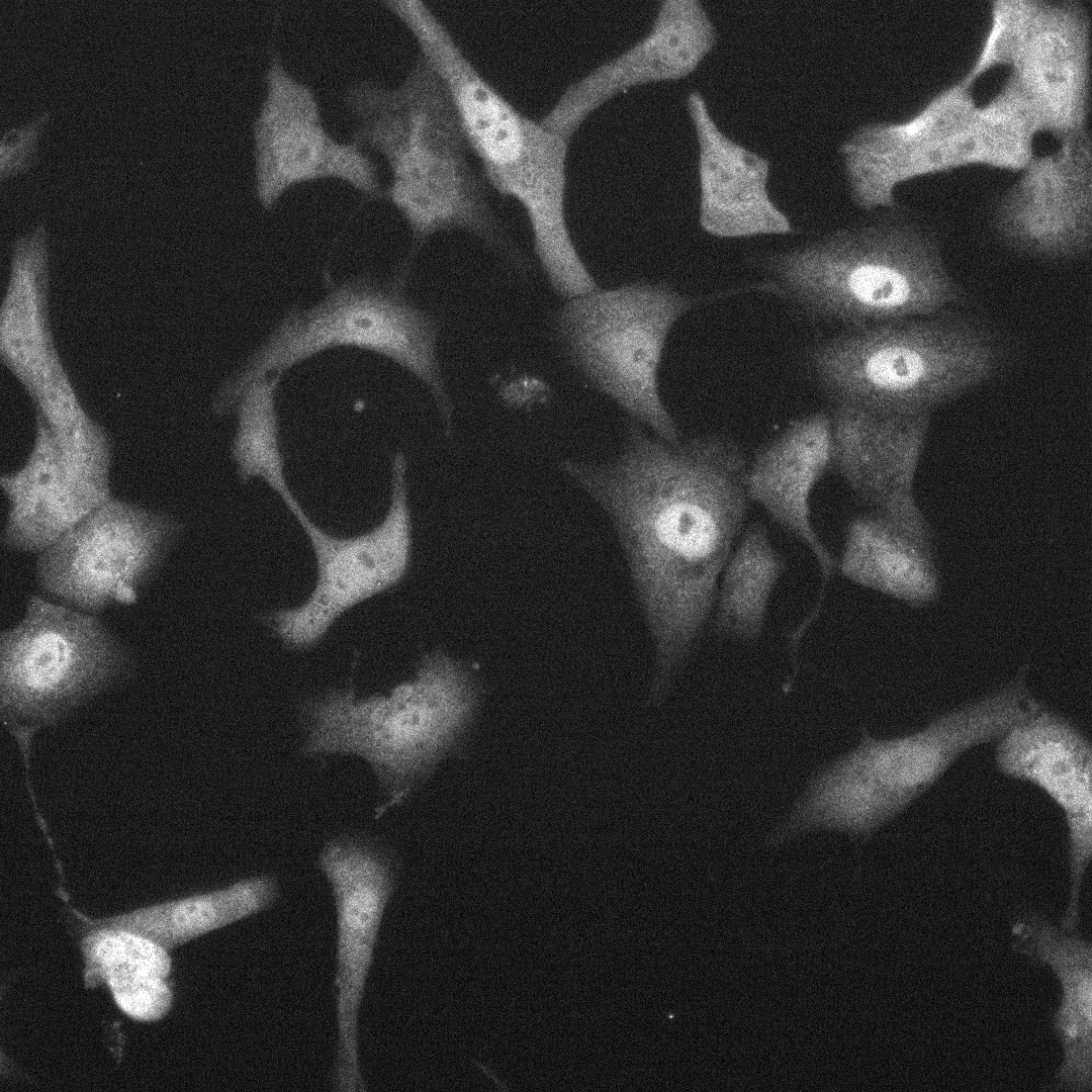

Supplement: Supplementary file 4 — Source Data for Figure 1 [file EMBJ-41-e111653-s006.zip › panel C/cyclin D1/cyclin D1.TIFF]

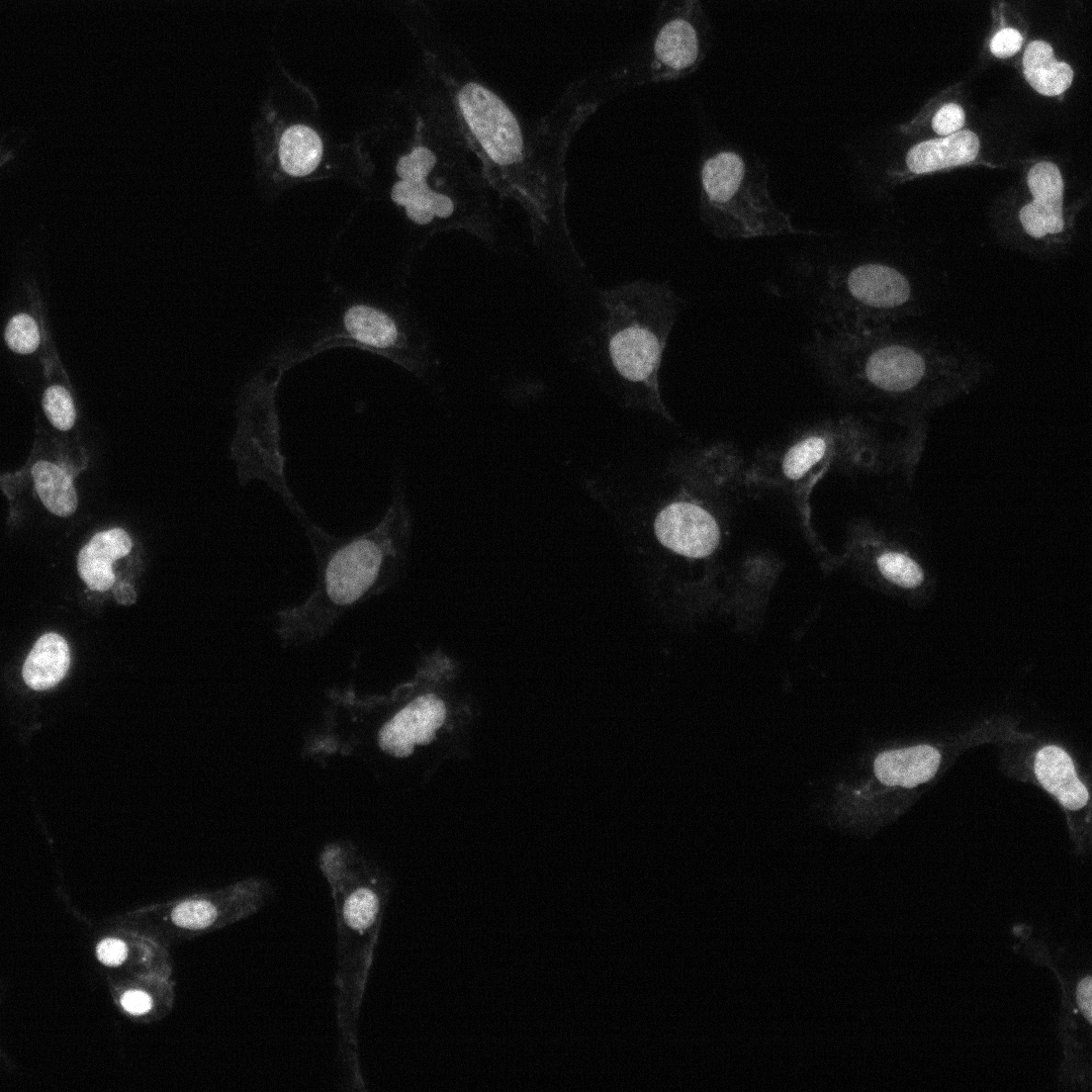

Supplement: Supplementary file 4 — Source Data for Figure 1 [file EMBJ-41-e111653-s006.zip › panel C/cyclin D1/nuclei.TIFF]

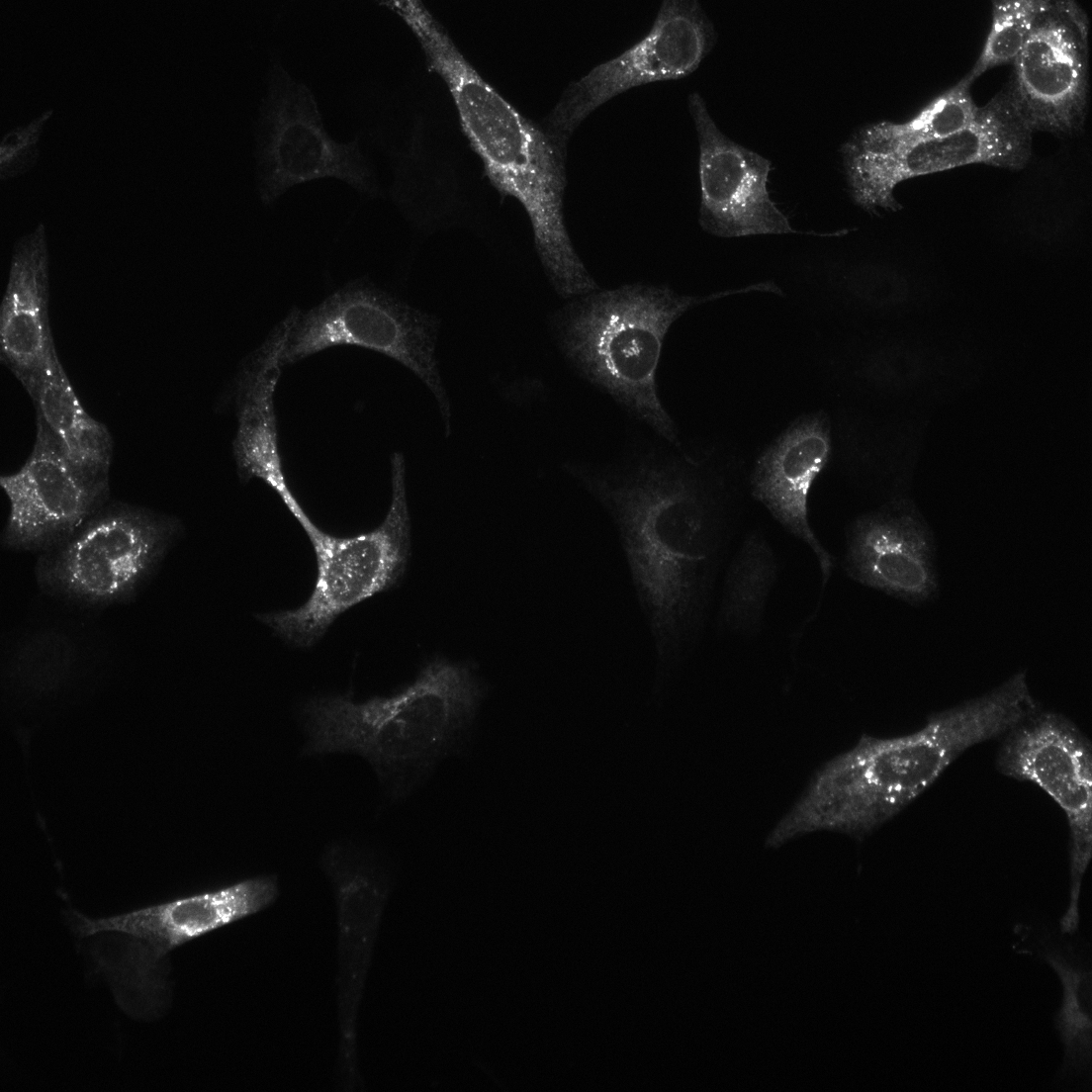

Supplement: Supplementary file 4 — Source Data for Figure 1 [file EMBJ-41-e111653-s006.zip › panel C/cyclin D1/SARS Spike.TIFF]

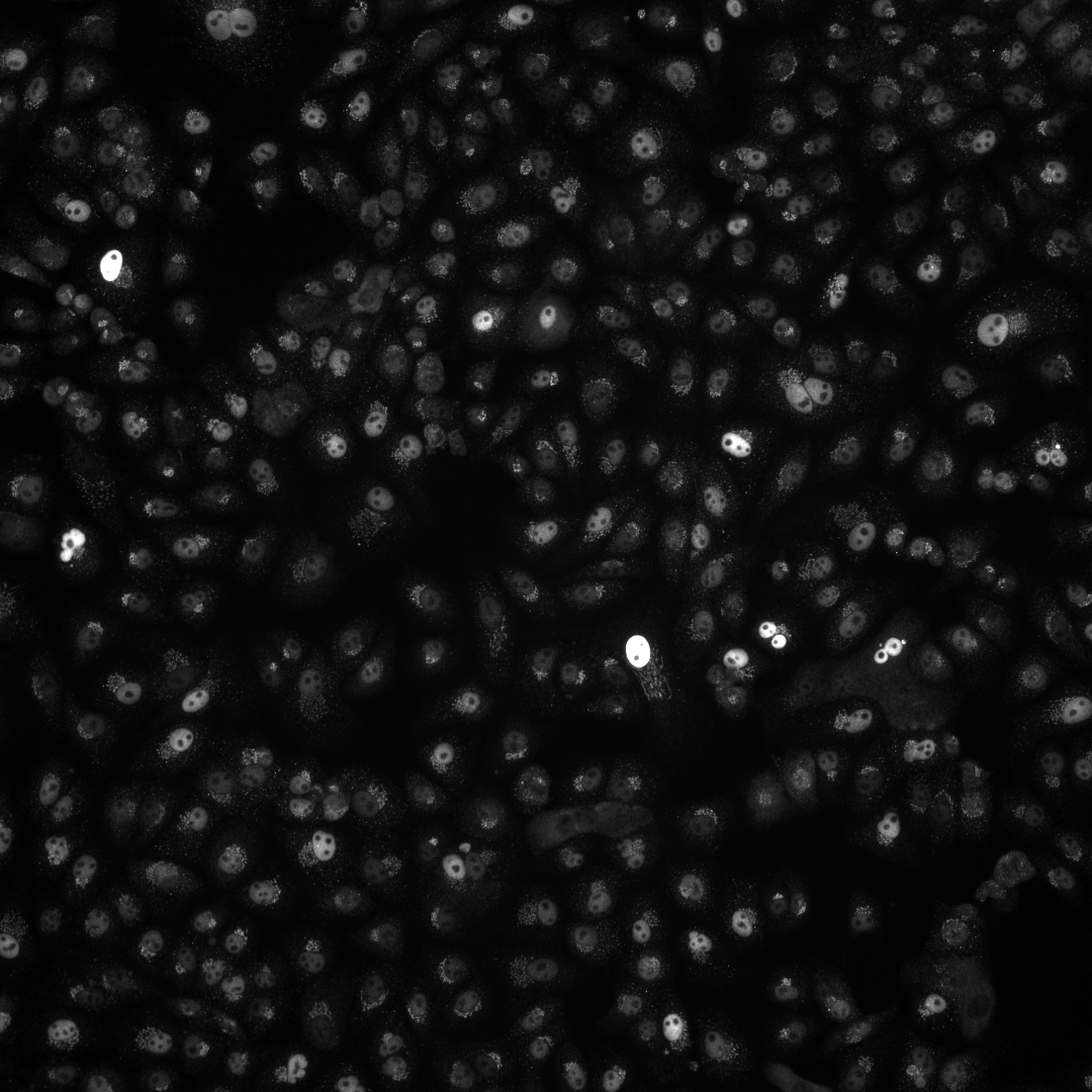

Supplement: Supplementary file 4 — Source Data for Figure 1 [file EMBJ-41-e111653-s006.zip › panel C/cyclin D3/cyclin D3.TIFF]

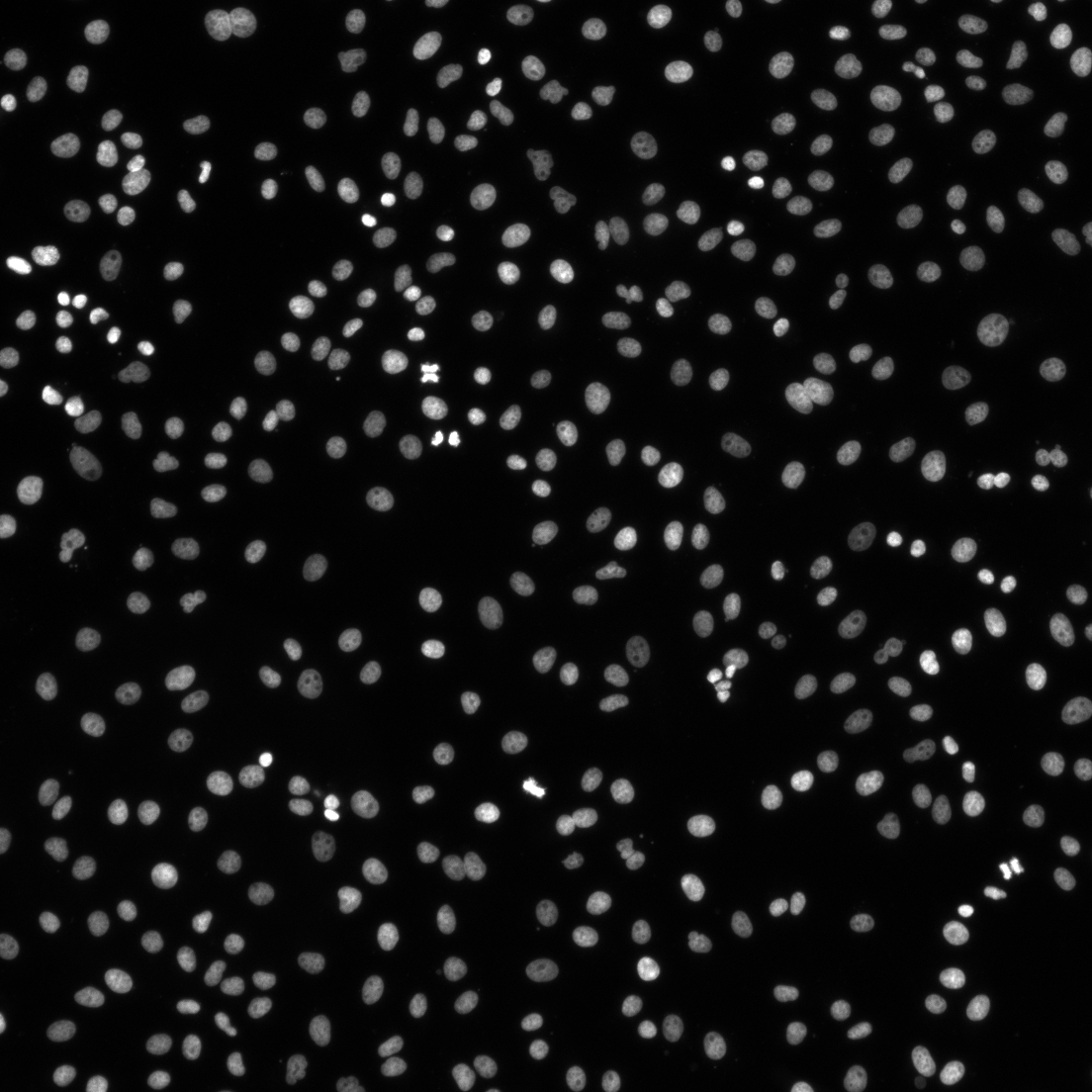

Supplement: Supplementary file 4 — Source Data for Figure 1 [file EMBJ-41-e111653-s006.zip › panel C/cyclin D3/nuclei.TIFF]

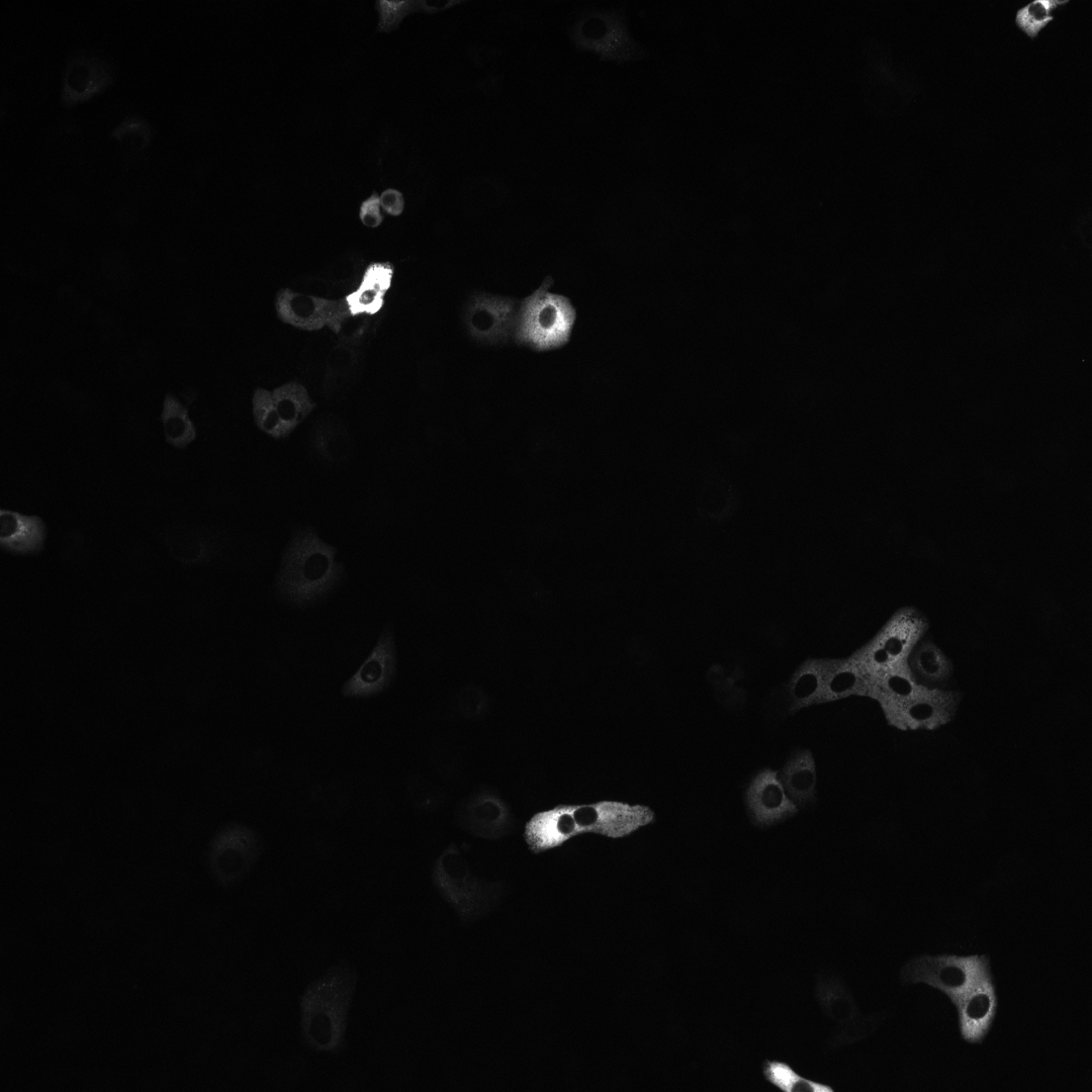

Supplement: Supplementary file 4 — Source Data for Figure 1 [file EMBJ-41-e111653-s006.zip › panel C/cyclin D3/SARS N.TIFF]

Figure 2

Figure 2A

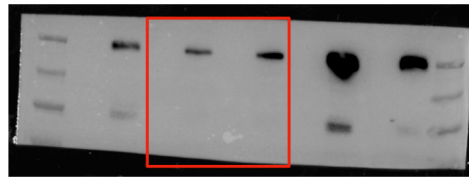

anti-Spike

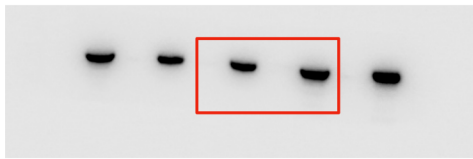

anti-nucleocapsid

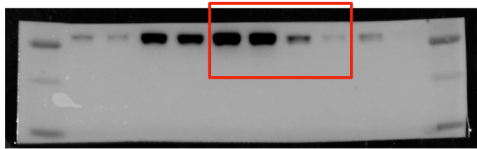

anti-cyclin D1

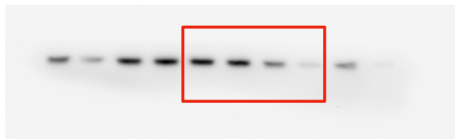

anti-cyclin D3

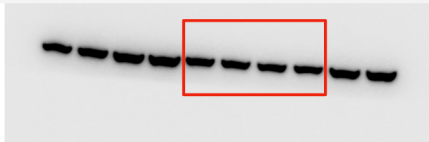

anti-actin

Supplement: Supplementary file 5 — Source Data for Figure 2 [file EMBJ-41-e111653-s004.zip › panel A/EMBOJ-2022-111653R-Figure_2_Source_Data-sd.pdf]

Figure 2 blot quantification

### EXPERIMENT 1

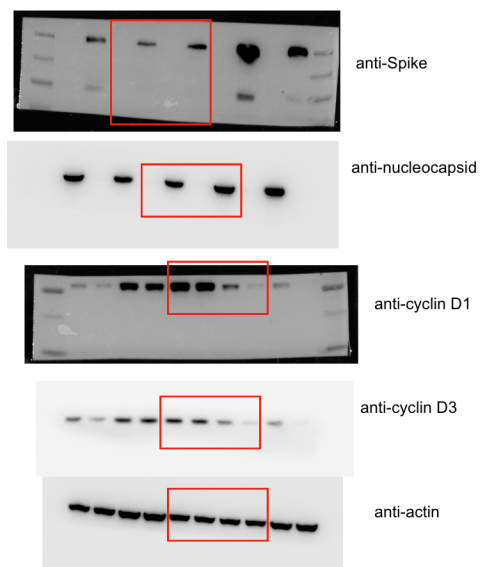

### EXPERIMENT 2

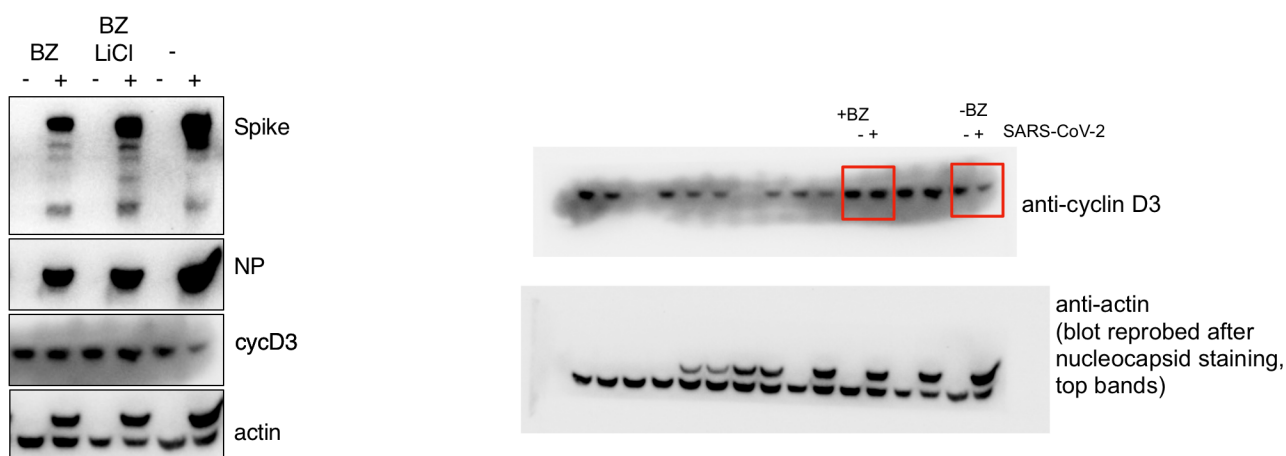

### EXPERIMENT 3

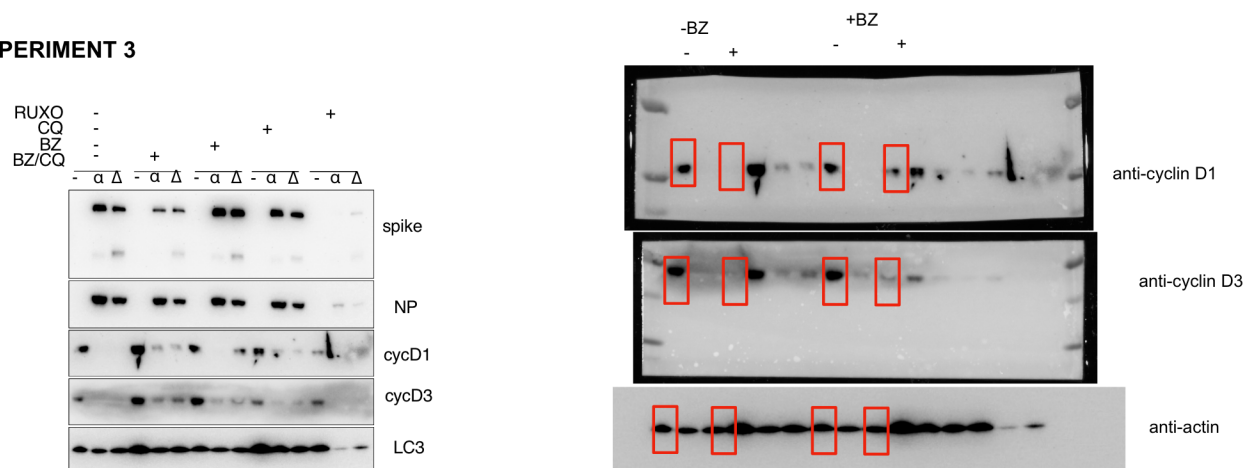

Supplement: Supplementary file 5 — Source Data for Figure 2 [file EMBJ-41-e111653-s004.zip › panel A/replicates.pdf]

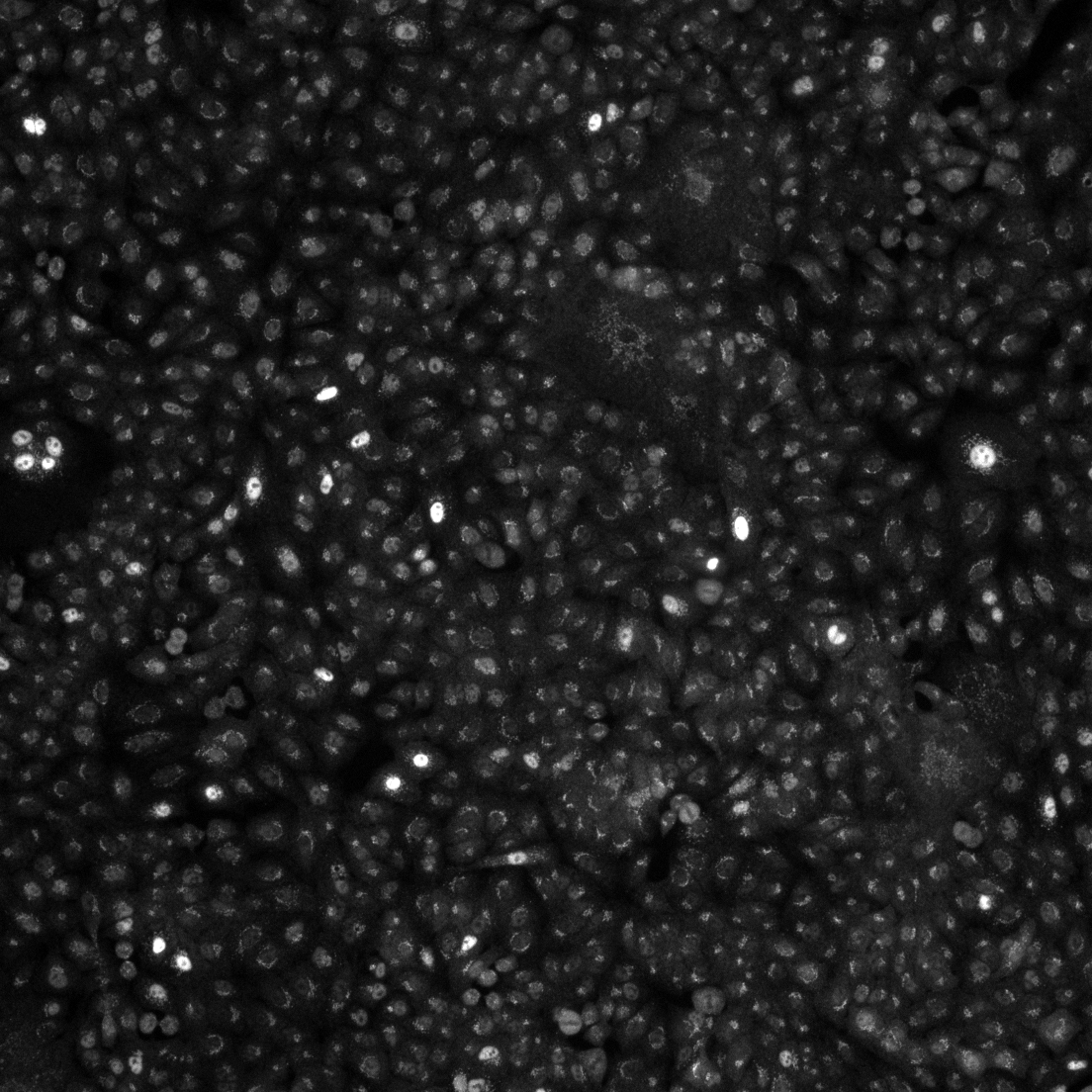

Supplement: Supplementary file 5 — Source Data for Figure 2 [file EMBJ-41-e111653-s004.zip › panel C/Infected -/cyclin D3.TIFF]

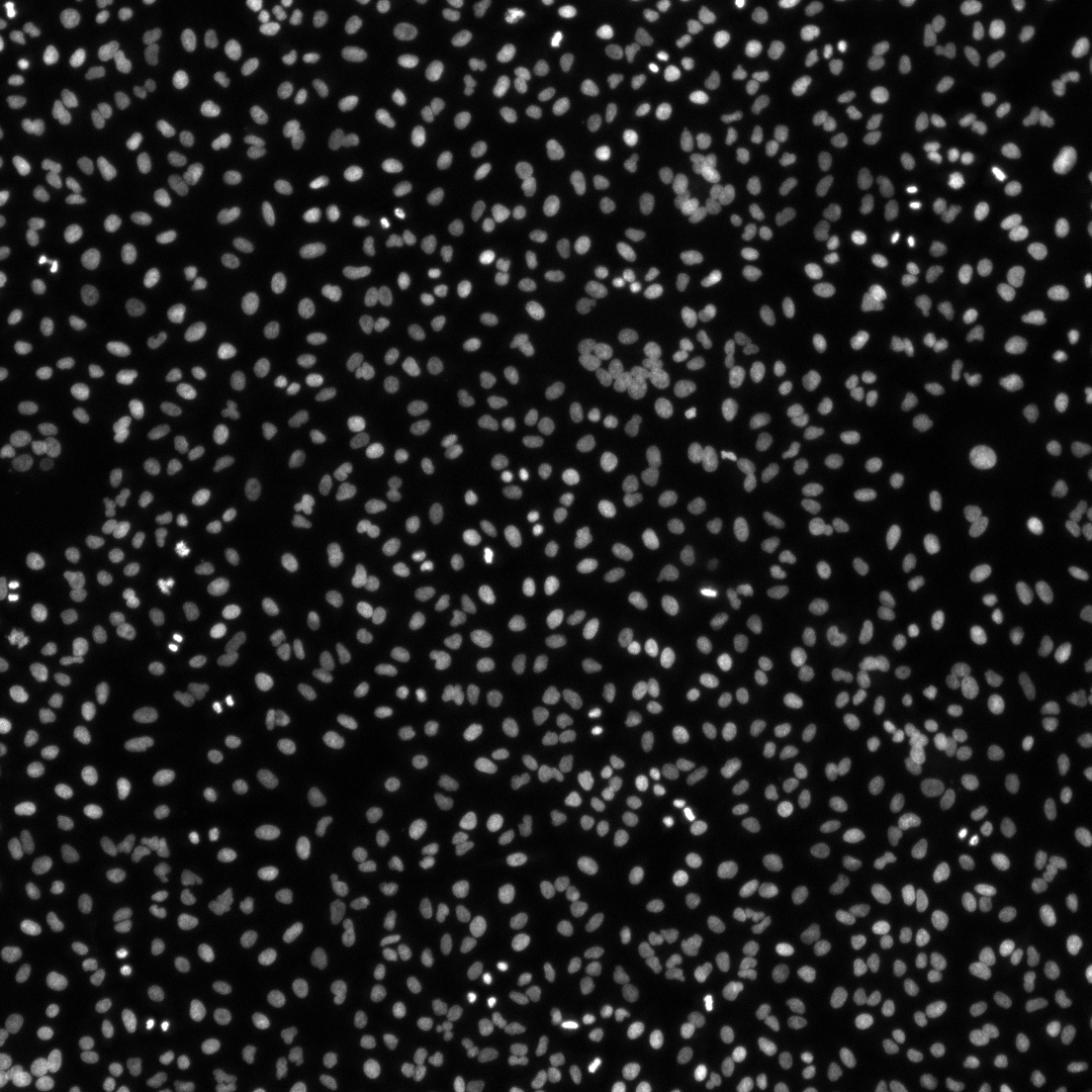

Supplement: Supplementary file 5 — Source Data for Figure 2 [file EMBJ-41-e111653-s004.zip › panel C/Infected -/nuclei.TIFF]

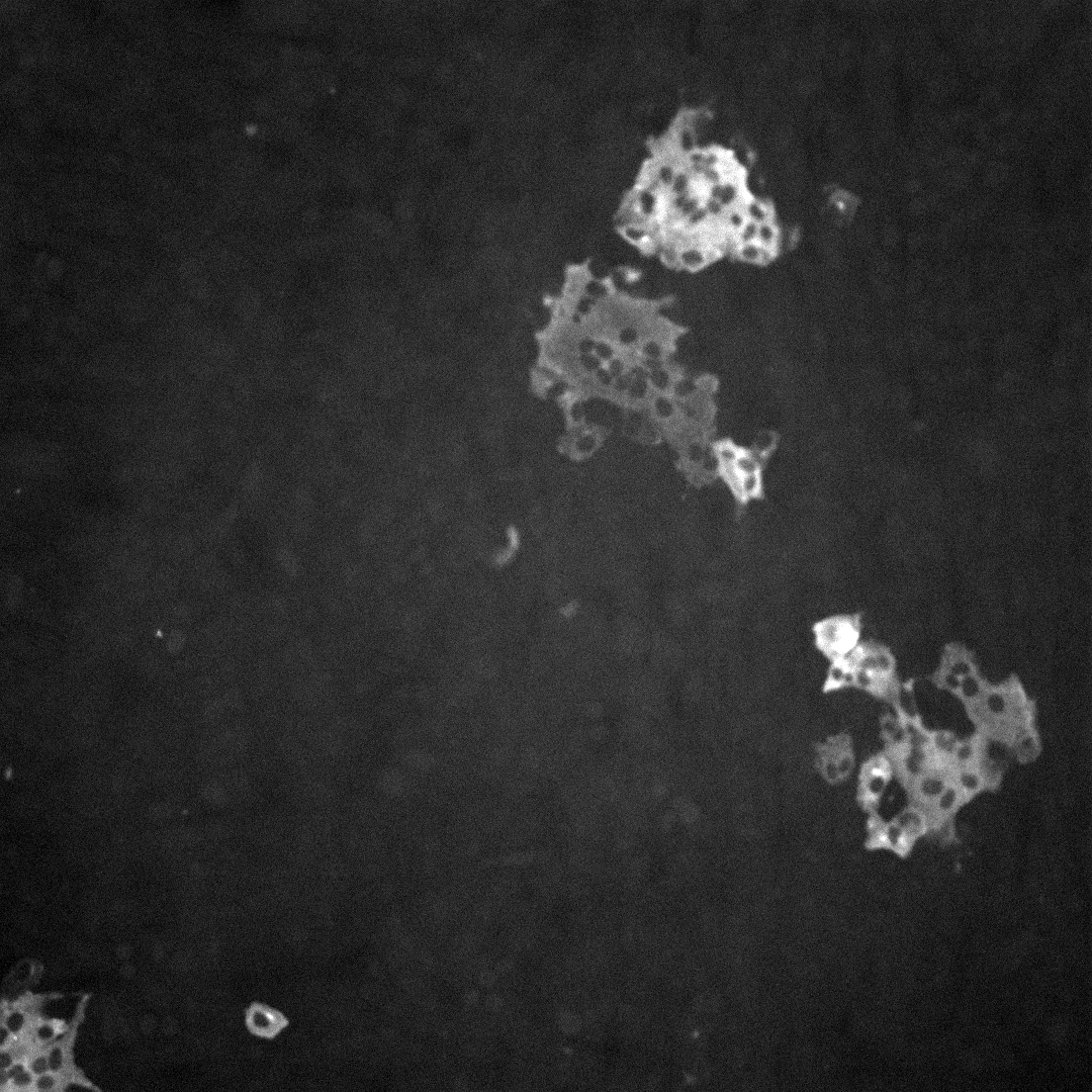

Supplement: Supplementary file 5 — Source Data for Figure 2 [file EMBJ-41-e111653-s004.zip › panel C/Infected -/SARS N.TIFF]

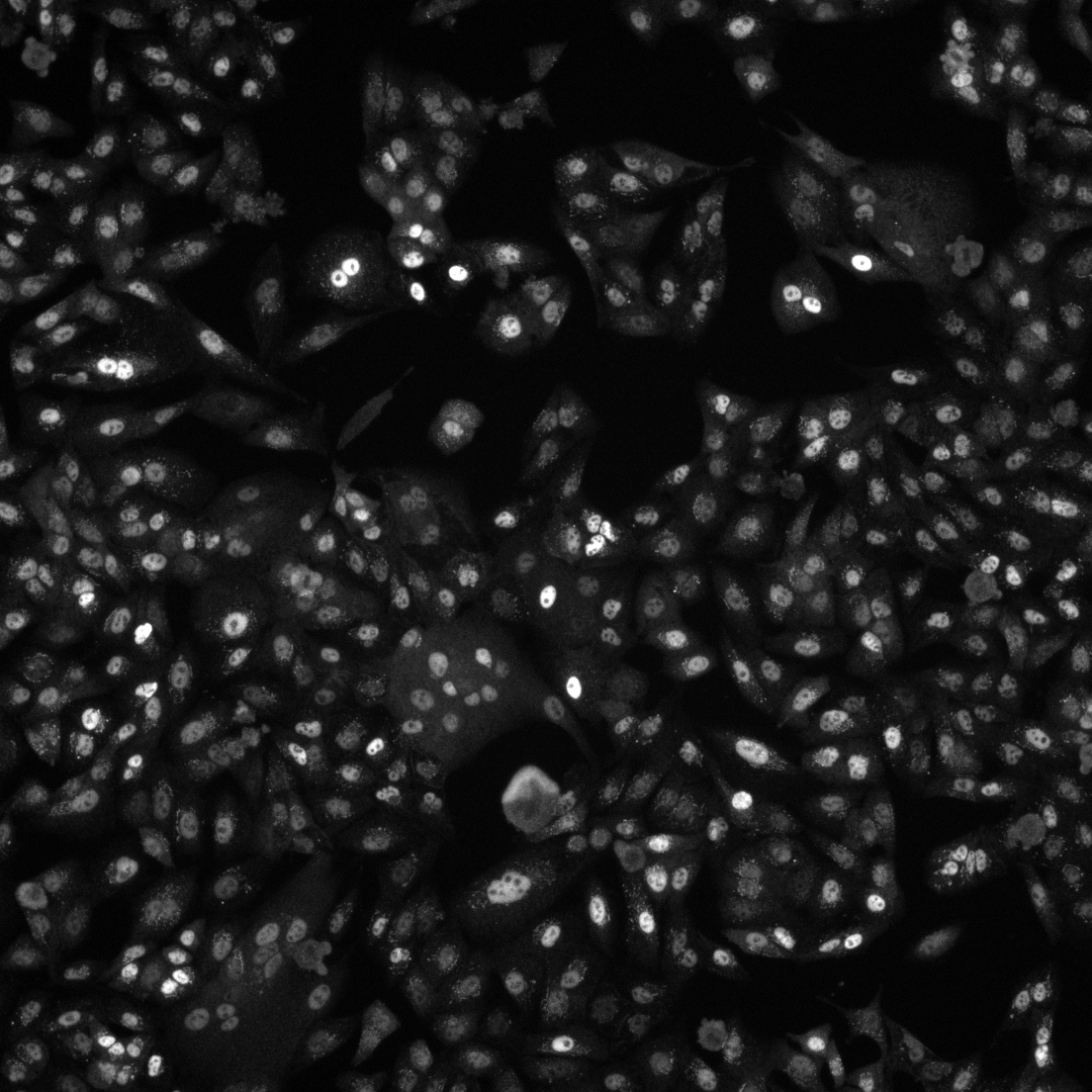

Supplement: Supplementary file 5 — Source Data for Figure 2 [file EMBJ-41-e111653-s004.zip › panel C/Infected BZ/cyclin D3.TIFF]

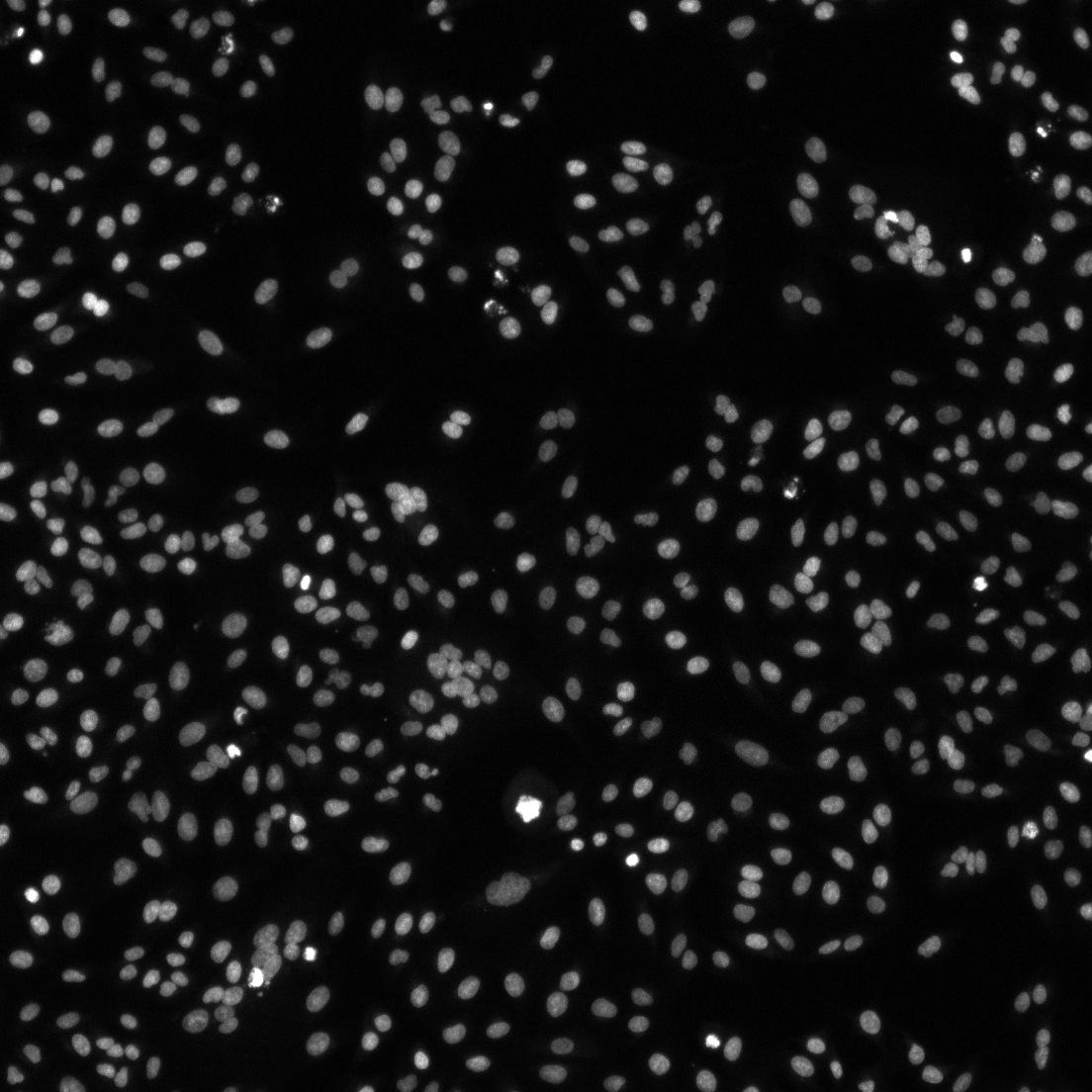

Supplement: Supplementary file 5 — Source Data for Figure 2 [file EMBJ-41-e111653-s004.zip › panel C/Infected BZ/nuclei.TIFF]

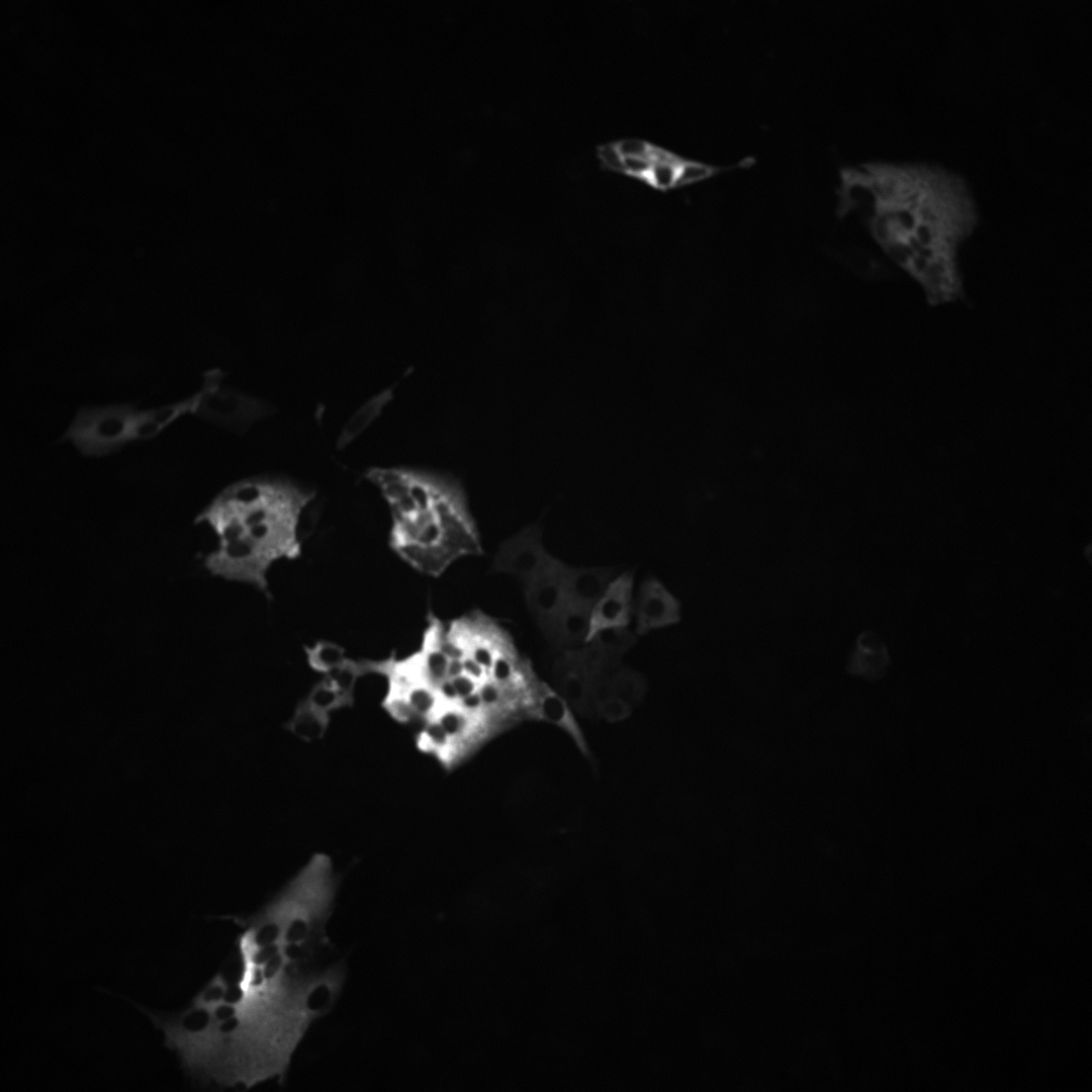

Supplement: Supplementary file 5 — Source Data for Figure 2 [file EMBJ-41-e111653-s004.zip › panel C/Infected BZ/SARS N.TIFF]

Figure 3

Figure 3A

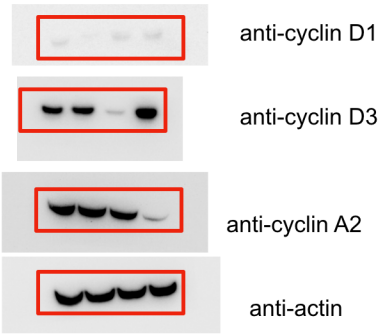

Figure 3C

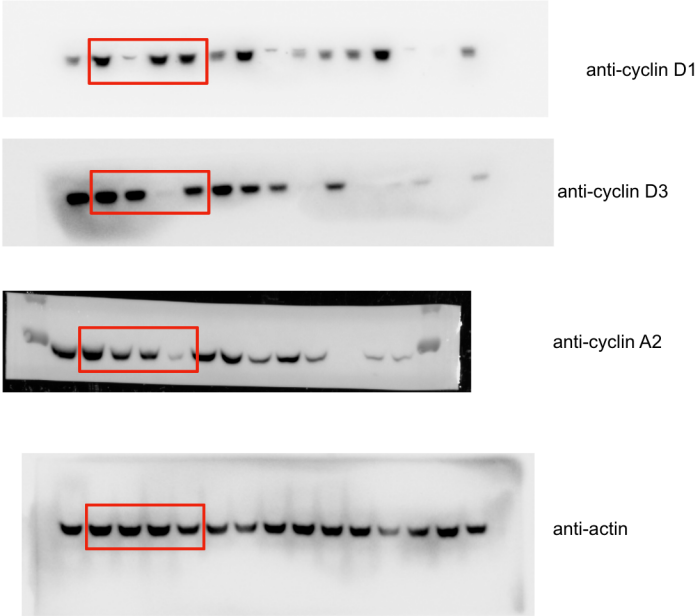

Figure 3E

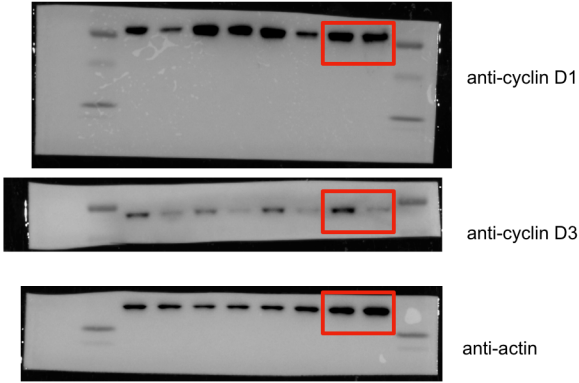

Supplement: Supplementary file 6 — Source Data for Figure 3 [file EMBJ-41-e111653-s002.zip › panel A C E/EMBOJ-2022-111653R-Figure_3_Source_Data-sd.pdf]

Figure 5

Figure 5A

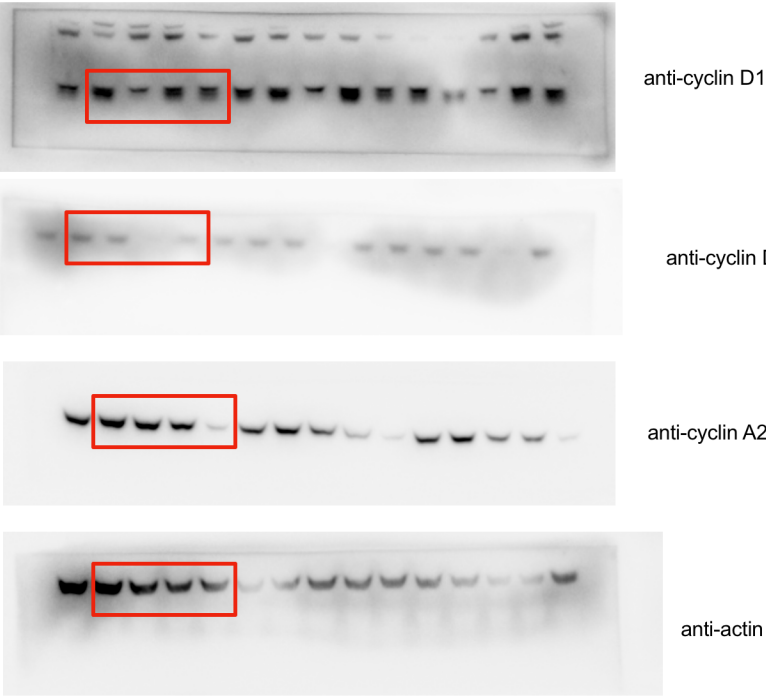

Figure 5E

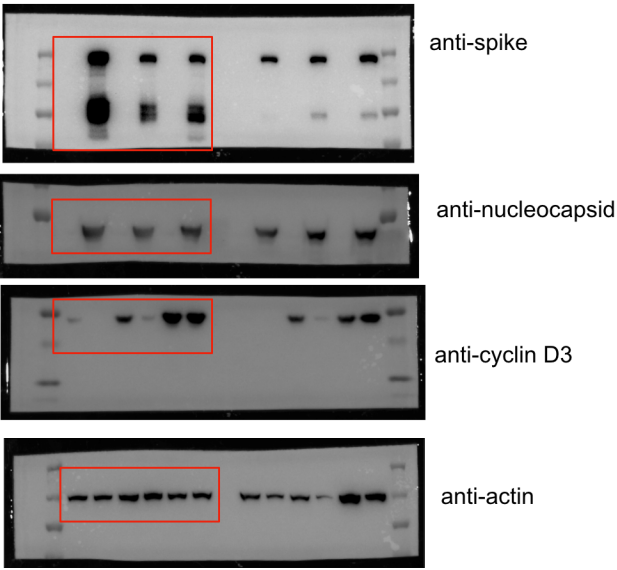

Supplement: Supplementary file 7 — Source Data for Figure 5 [file EMBJ-41-e111653-s010.zip › panel A E/EMBOJ-2022-111653R-Figure_5_Source_Data-sd.pdf]

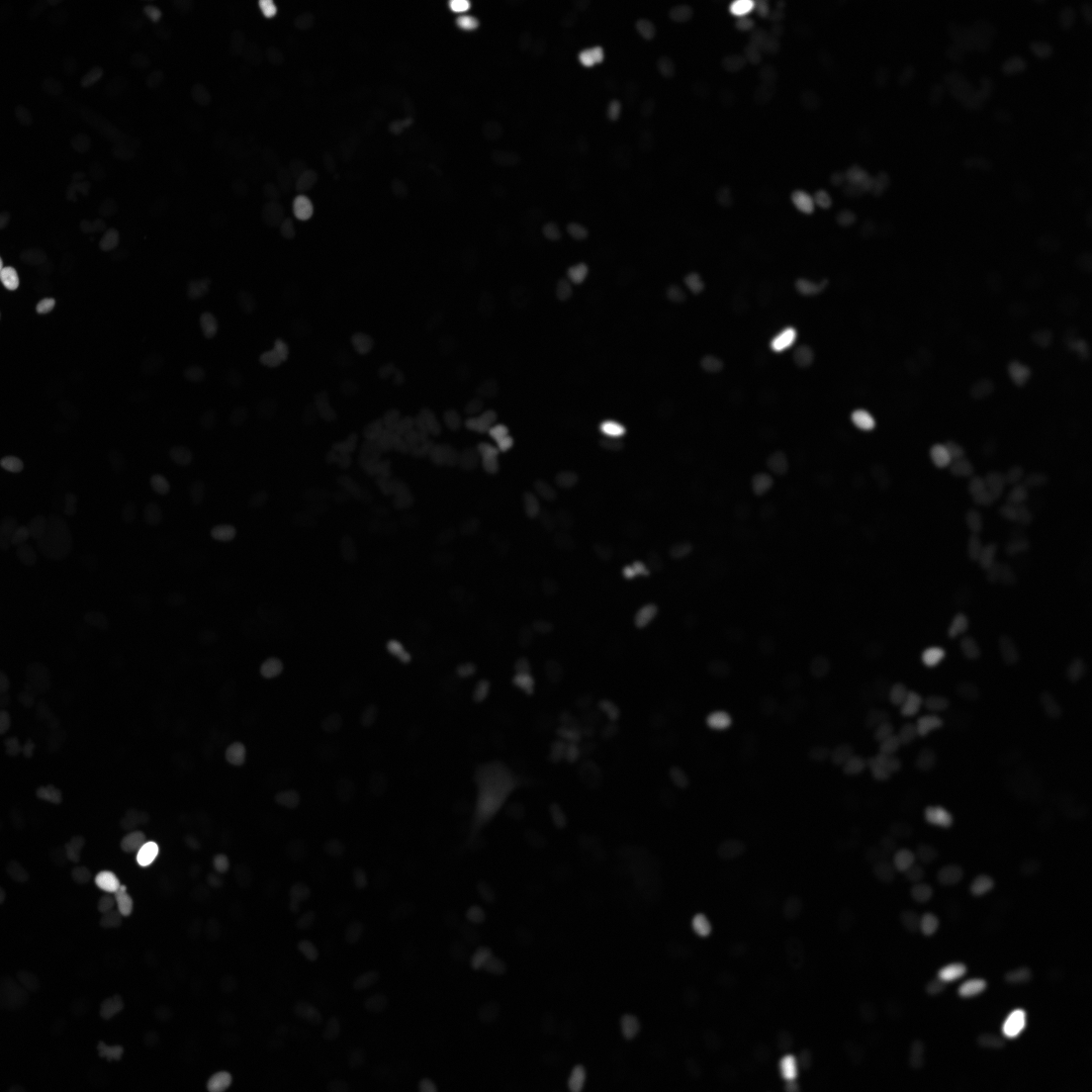

Supplement: Supplementary file 7 — Source Data for Figure 5 [file EMBJ-41-e111653-s010.zip › panel F/Cdt1.TIFF]

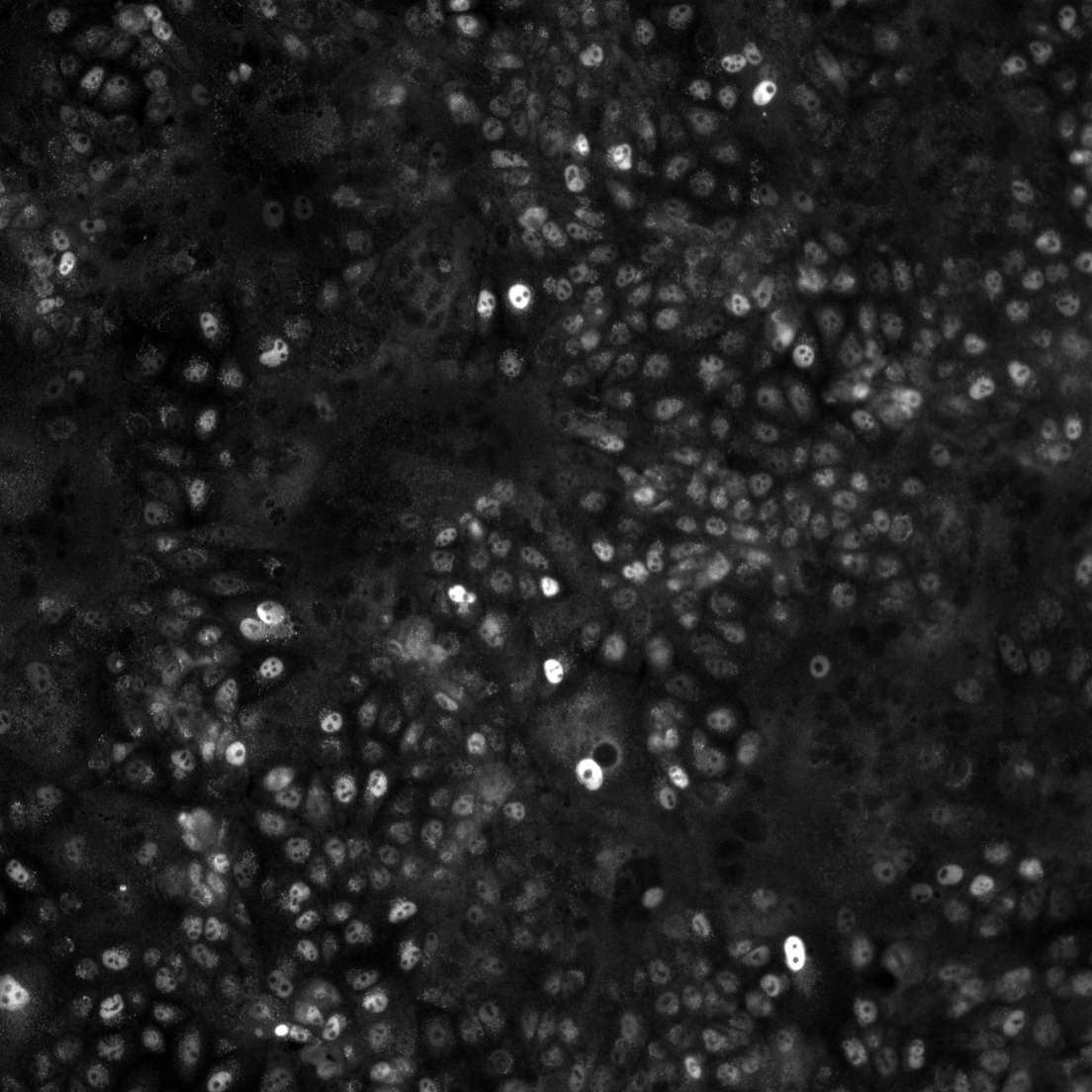

Supplement: Supplementary file 7 — Source Data for Figure 5 [file EMBJ-41-e111653-s010.zip › panel F/cyclin D3.TIFF]

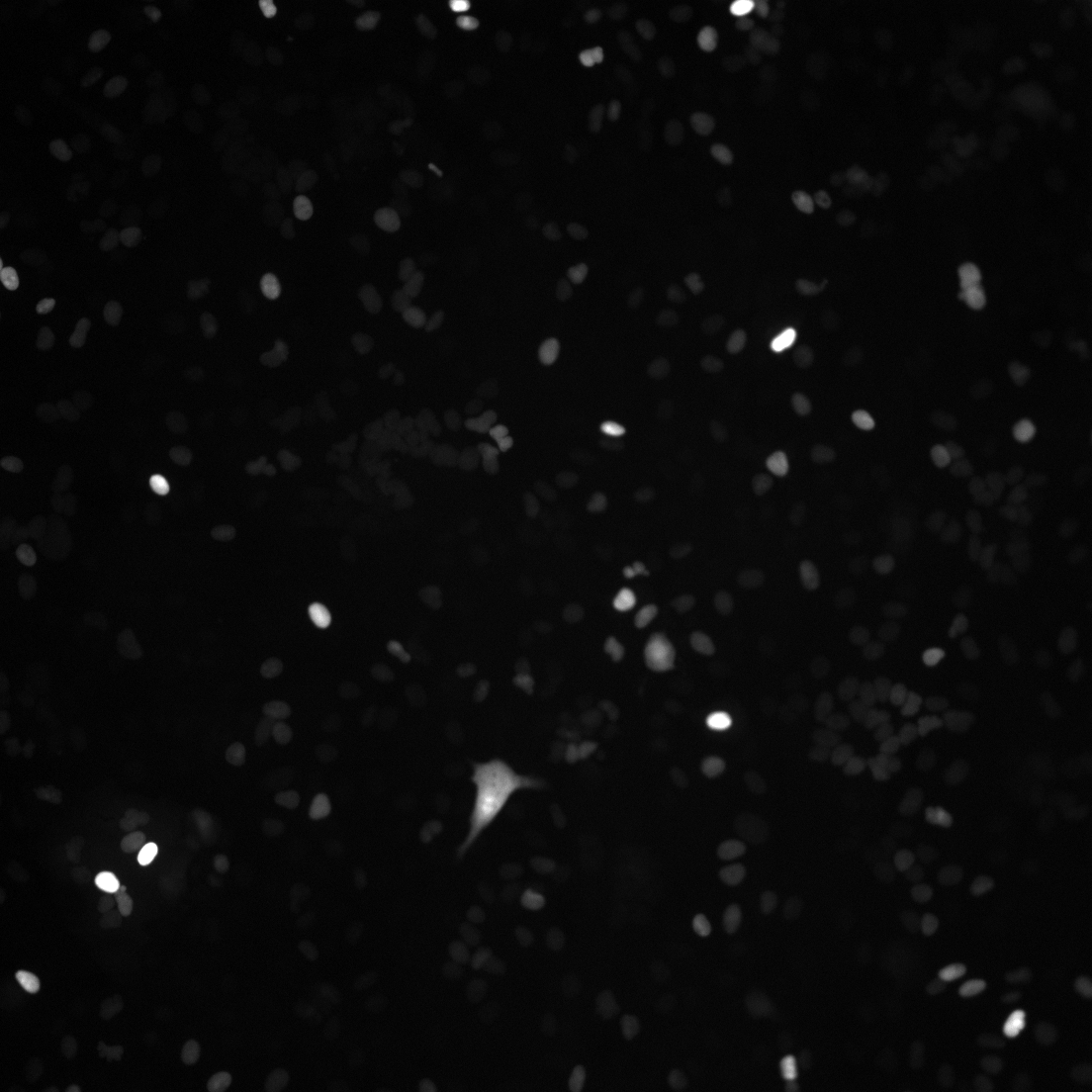

Supplement: Supplementary file 7 — Source Data for Figure 5 [file EMBJ-41-e111653-s010.zip › panel F/Geminin.TIFF]

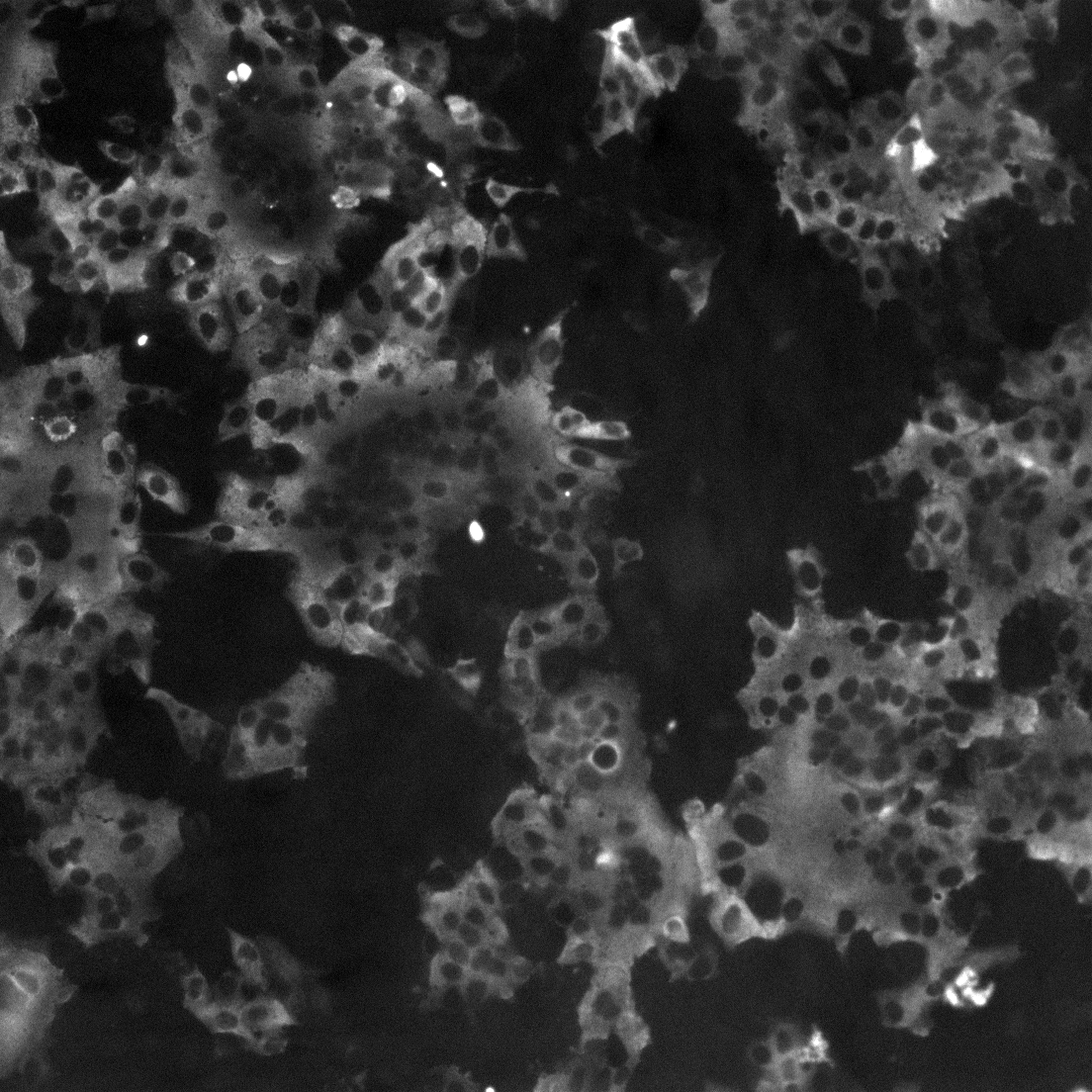

Supplement: Supplementary file 7 — Source Data for Figure 5 [file EMBJ-41-e111653-s010.zip › panel F/SARS N.TIFF]

Figure 6

Figure 6A

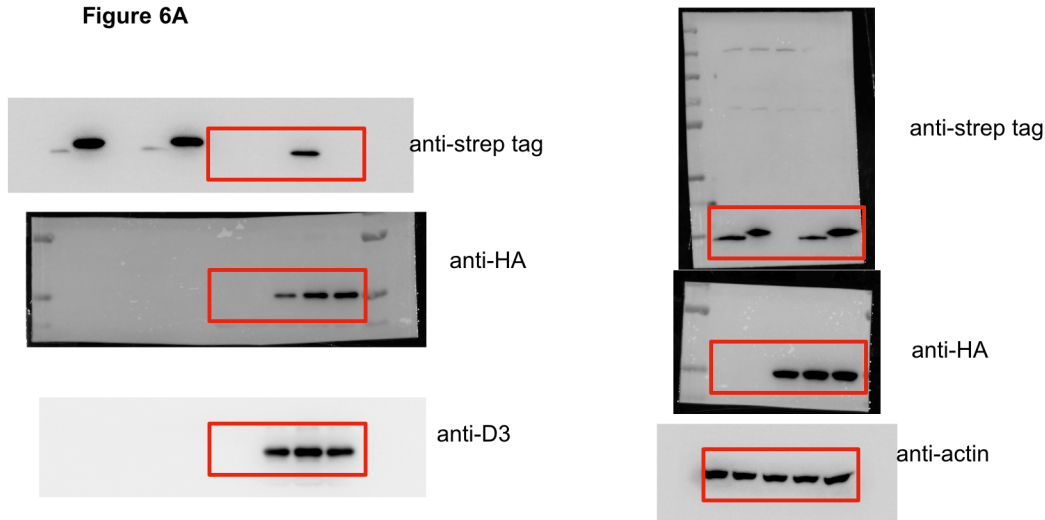

Figure 6B

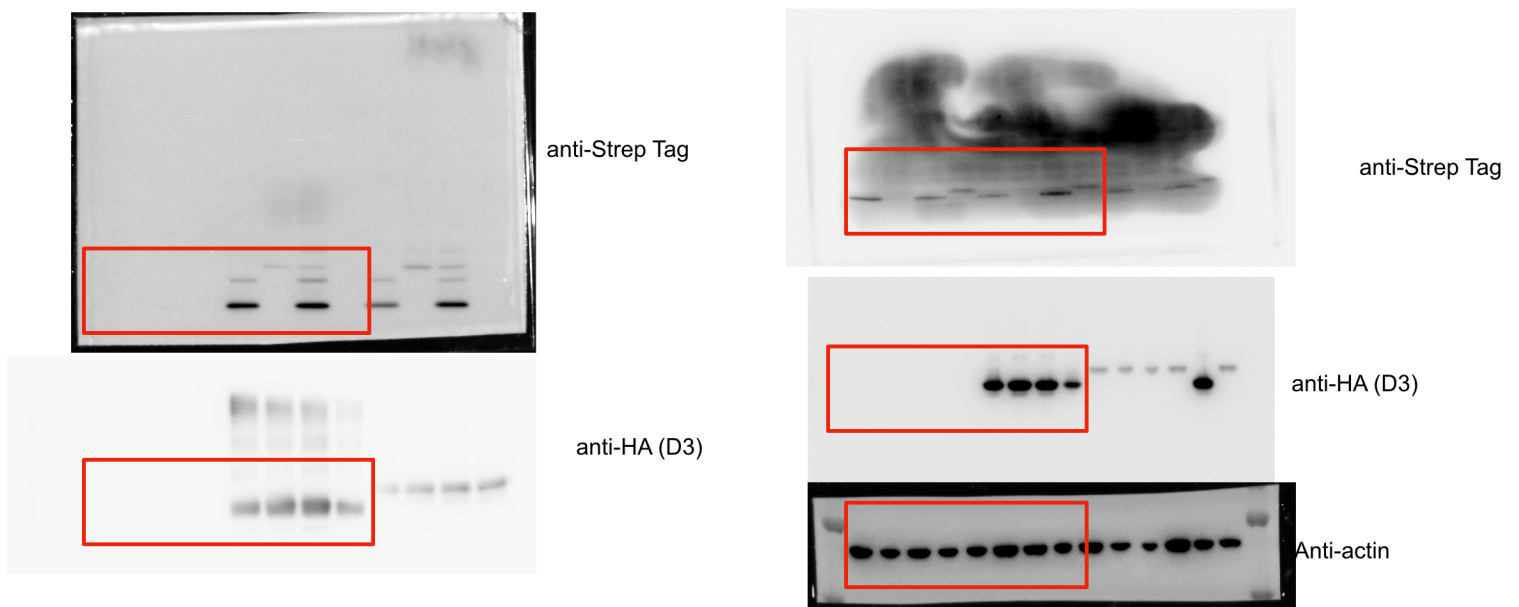

Figure 6I

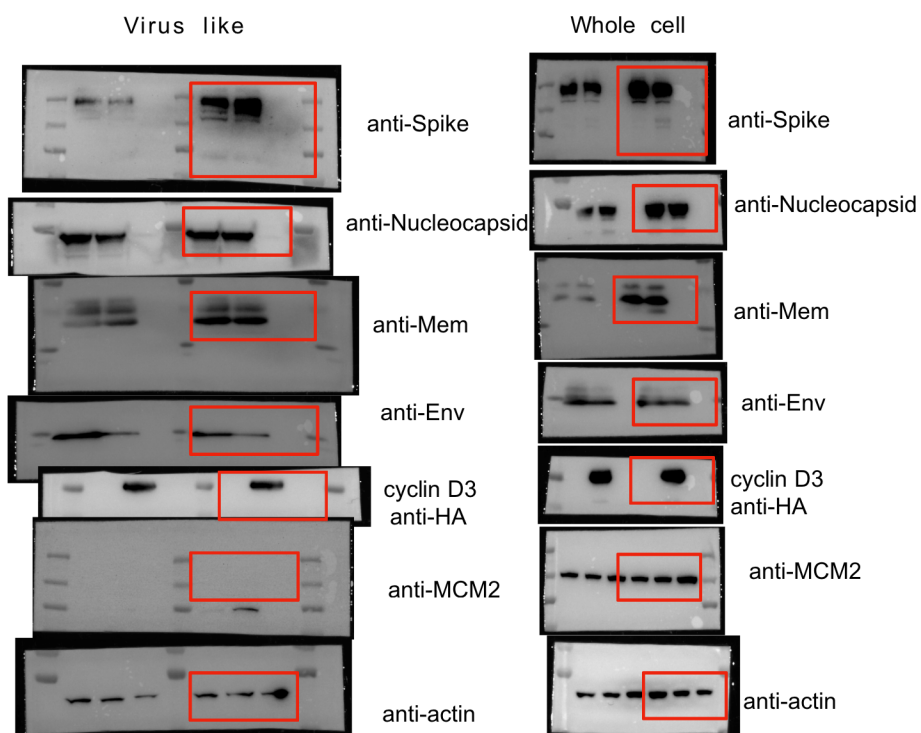

Supplement: Supplementary file 8 — Source Data for Figure 6 [file EMBJ-41-e111653-s003.zip › panel A B I/EMBOJ-2022-111653R-Figure_6_Source_Data-sd.pdf]

Figure 6 blot quantification

EXPERIMENT 1

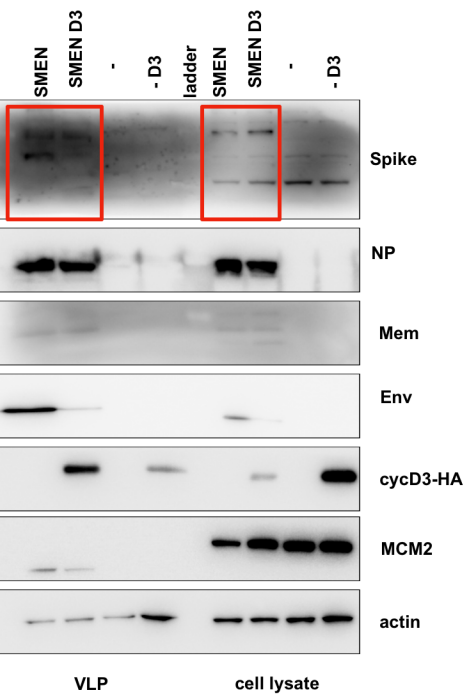

EXPERIMENT 2

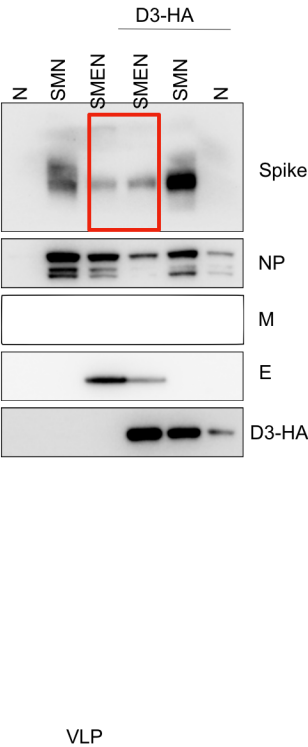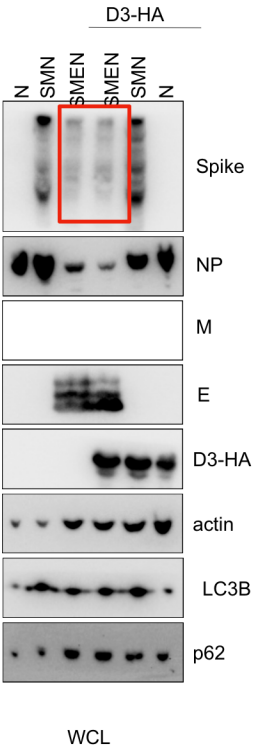

EXPERIMENT 3,4

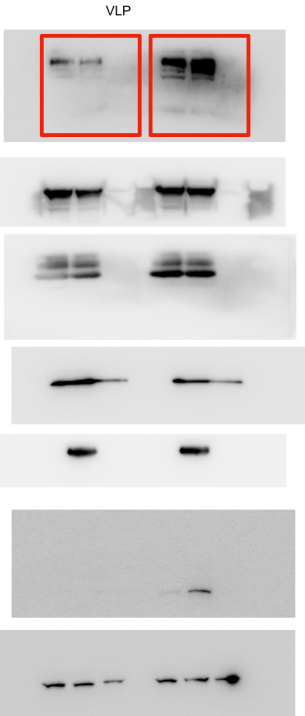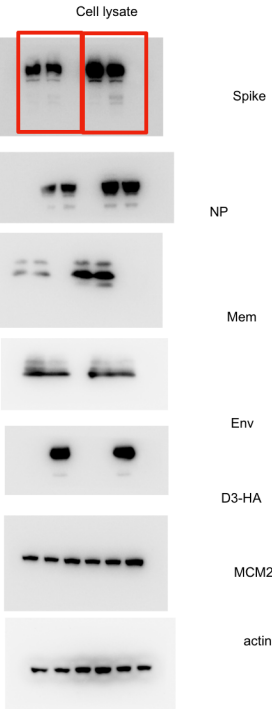

Supplement: Supplementary file 8 — Source Data for Figure 6 [file EMBJ-41-e111653-s003.zip › panel A B I/replicates.pdf]

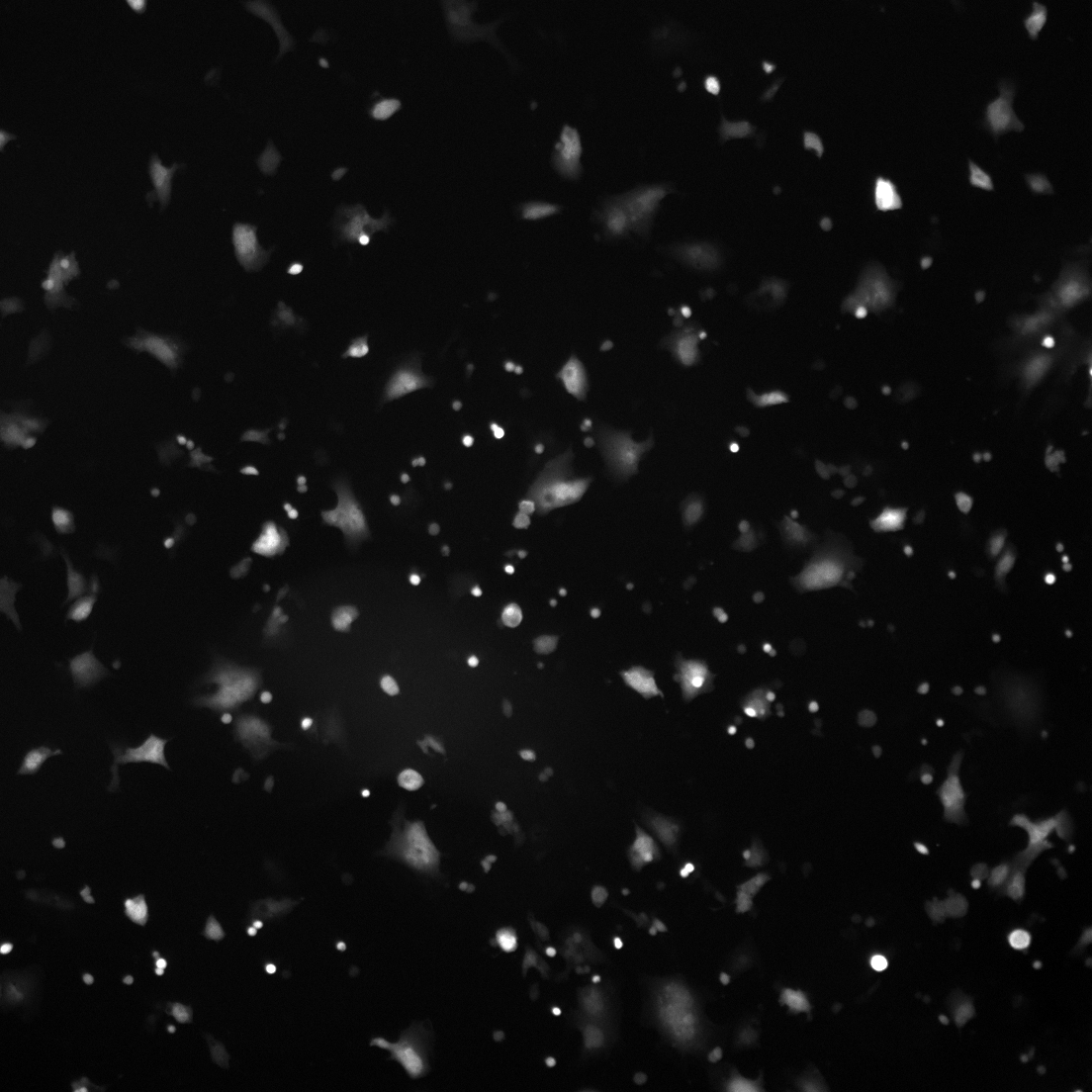

Supplement: Supplementary file 8 — Source Data for Figure 6 [file EMBJ-41-e111653-s003.zip › panel C/SE/GFP.TIFF]

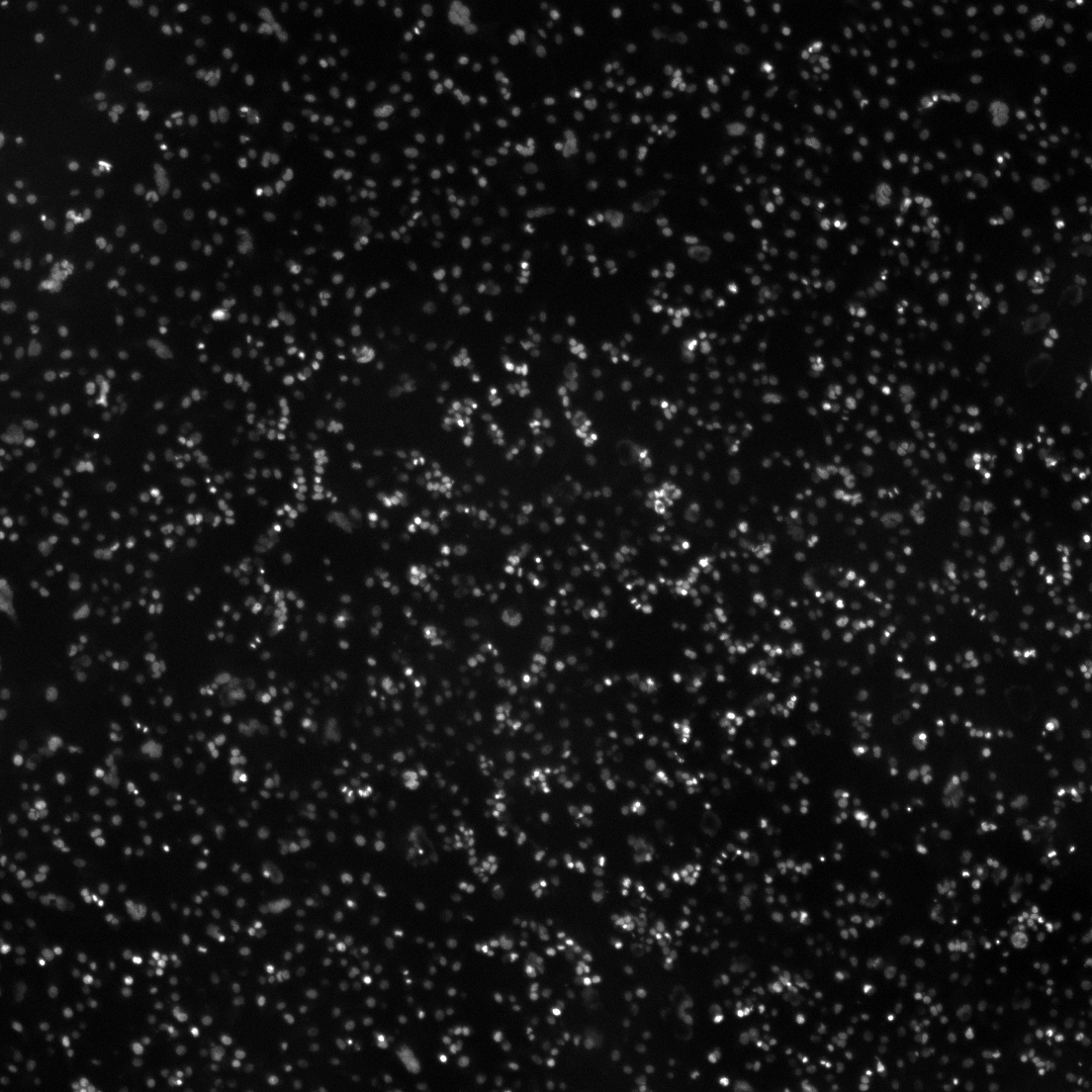

Supplement: Supplementary file 8 — Source Data for Figure 6 [file EMBJ-41-e111653-s003.zip › panel C/SE/nuclei.TIFF]

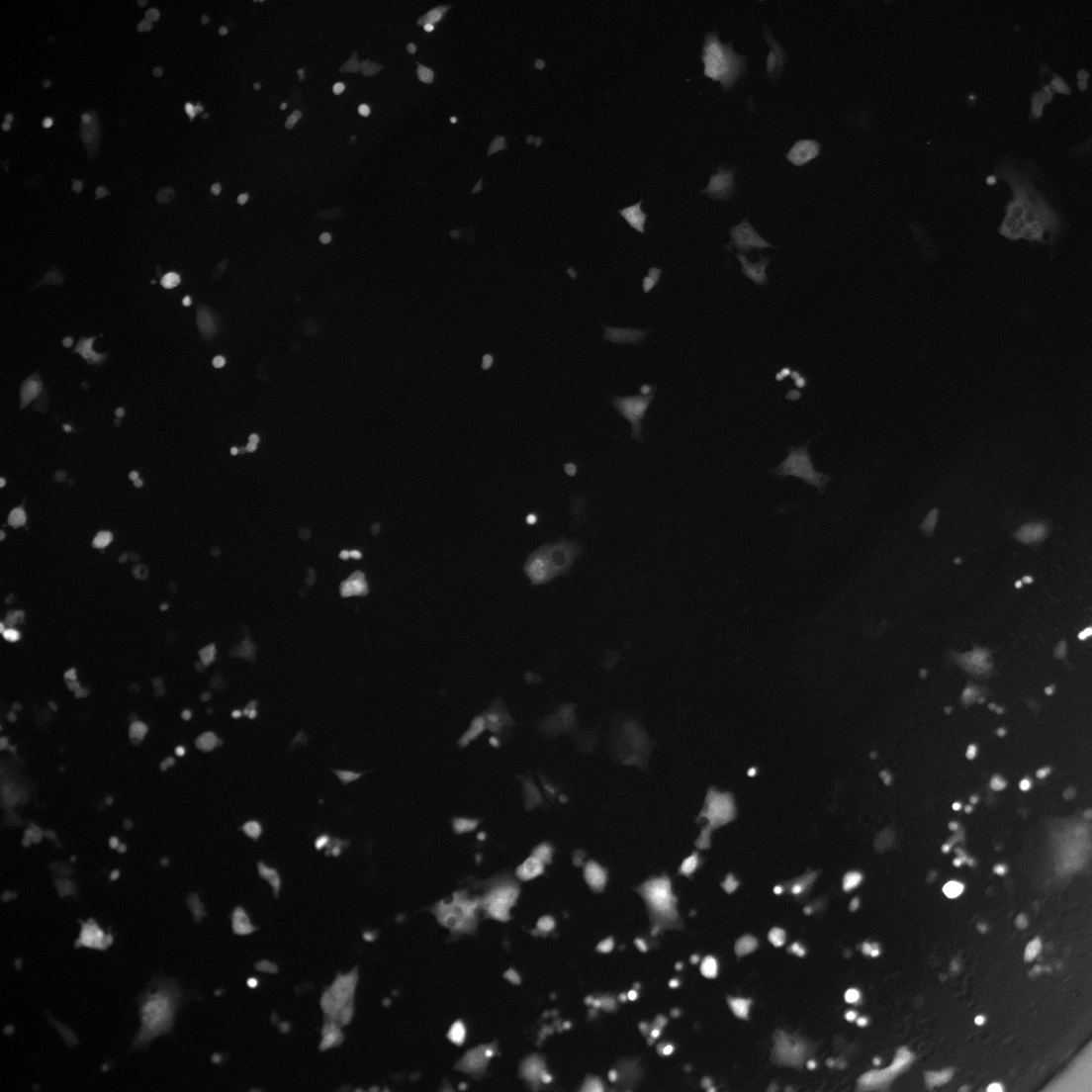

Supplement: Supplementary file 8 — Source Data for Figure 6 [file EMBJ-41-e111653-s003.zip › panel C/SME/GFP.tif]

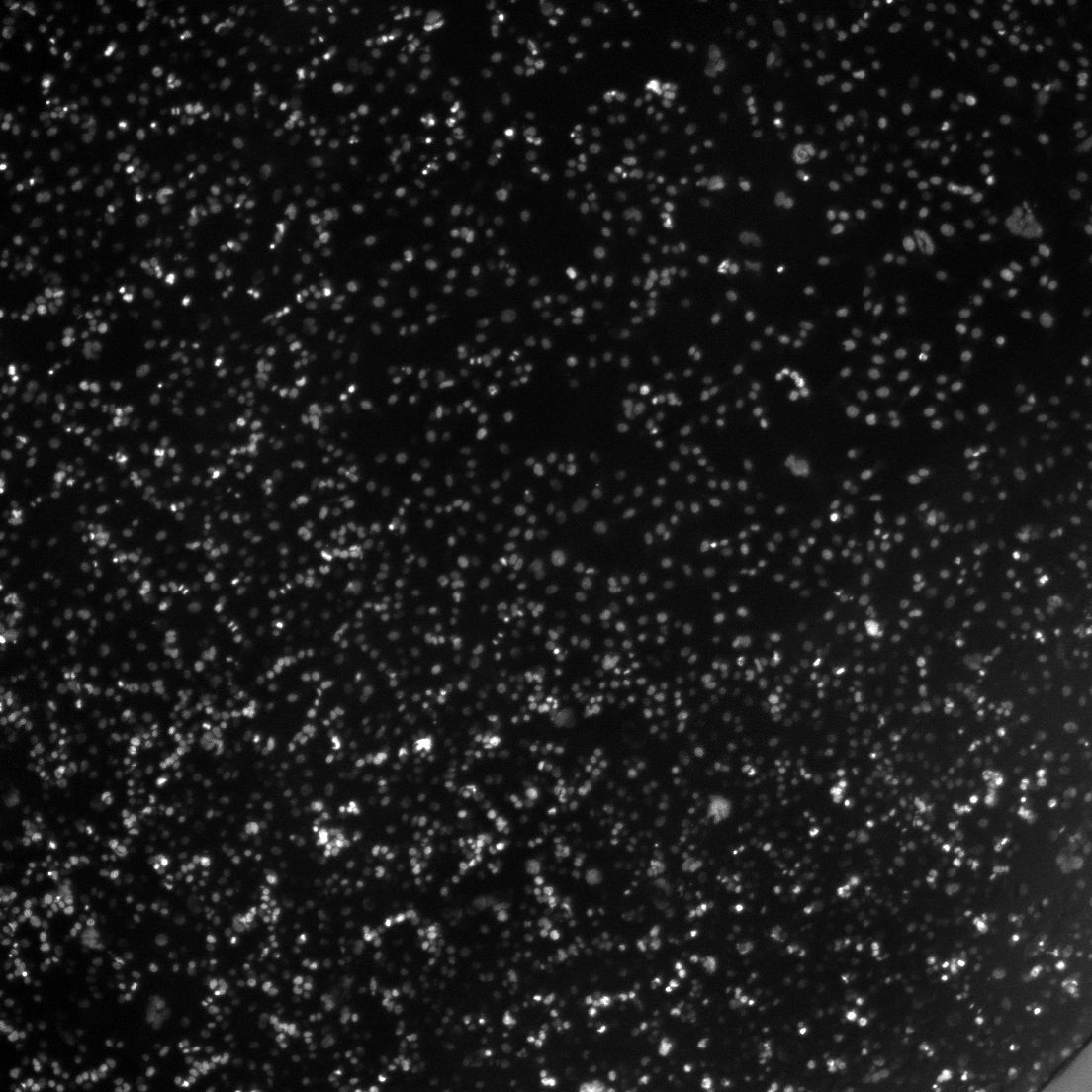

Supplement: Supplementary file 8 — Source Data for Figure 6 [file EMBJ-41-e111653-s003.zip › panel C/SME/nuclei.tif]

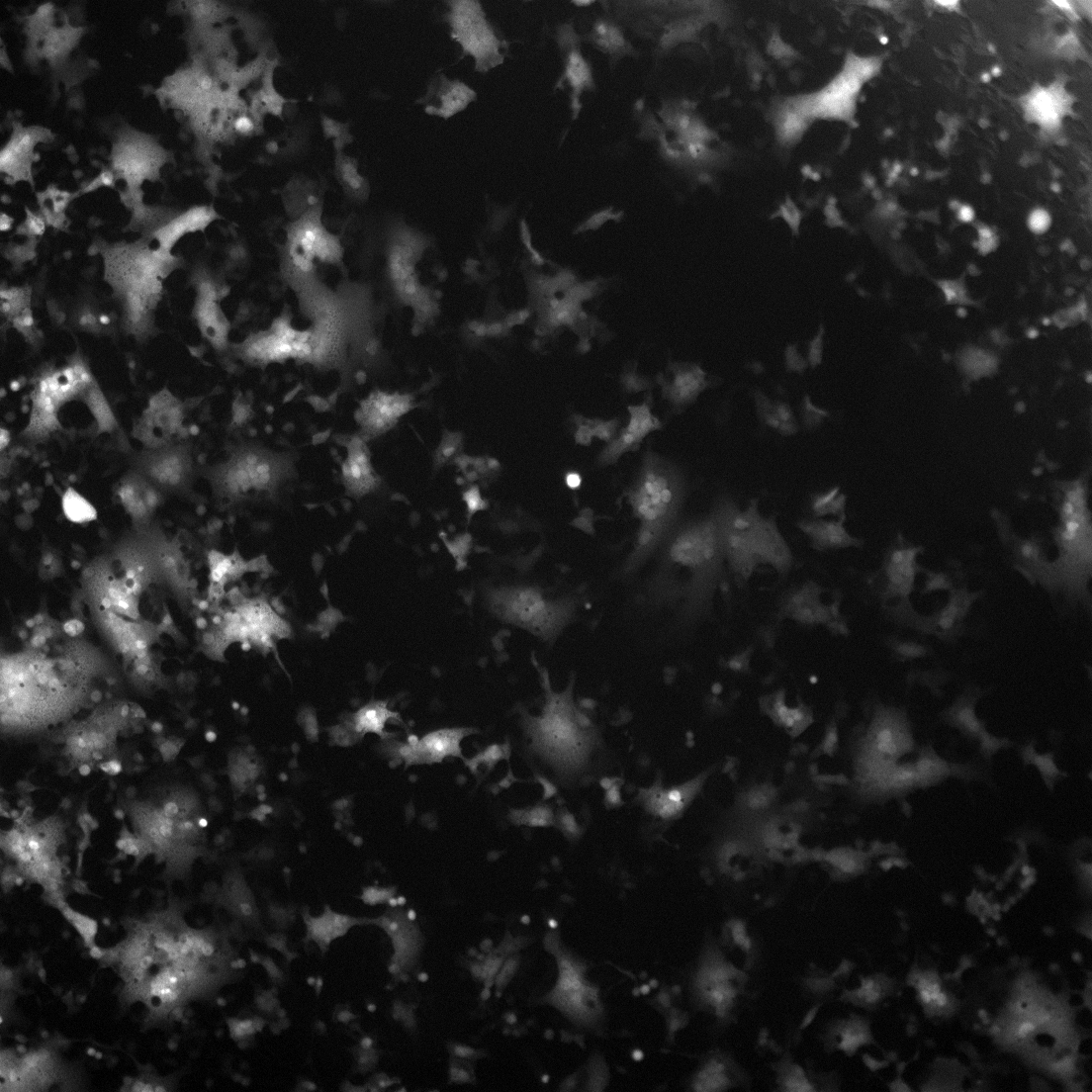

Supplement: Supplementary file 8 — Source Data for Figure 6 [file EMBJ-41-e111653-s003.zip › panel C/SM/GFP.TIFF]

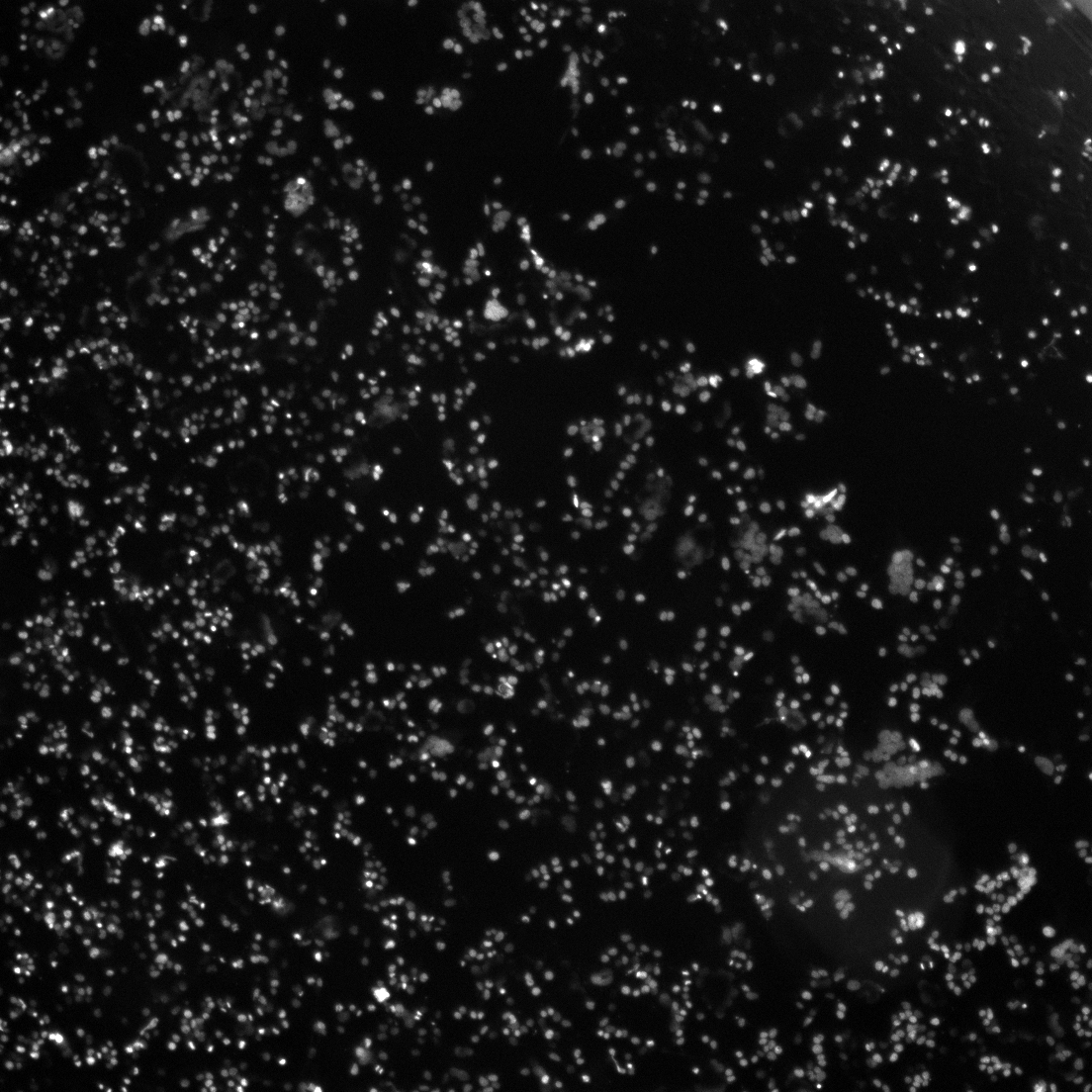

Supplement: Supplementary file 8 — Source Data for Figure 6 [file EMBJ-41-e111653-s003.zip › panel C/SM/nuclei.TIFF]

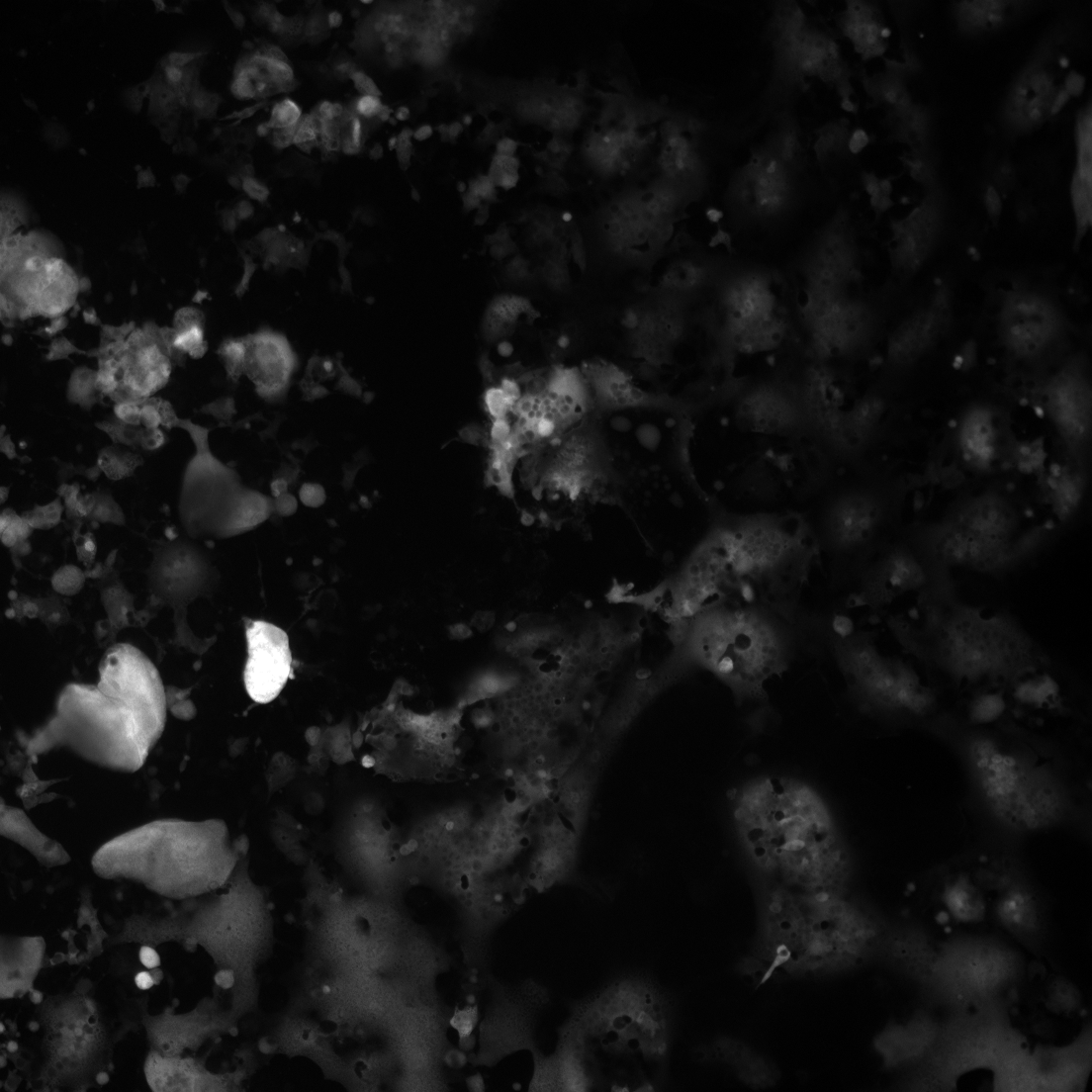

Supplement: Supplementary file 8 — Source Data for Figure 6 [file EMBJ-41-e111653-s003.zip › panel C/S/GFP.TIFF]

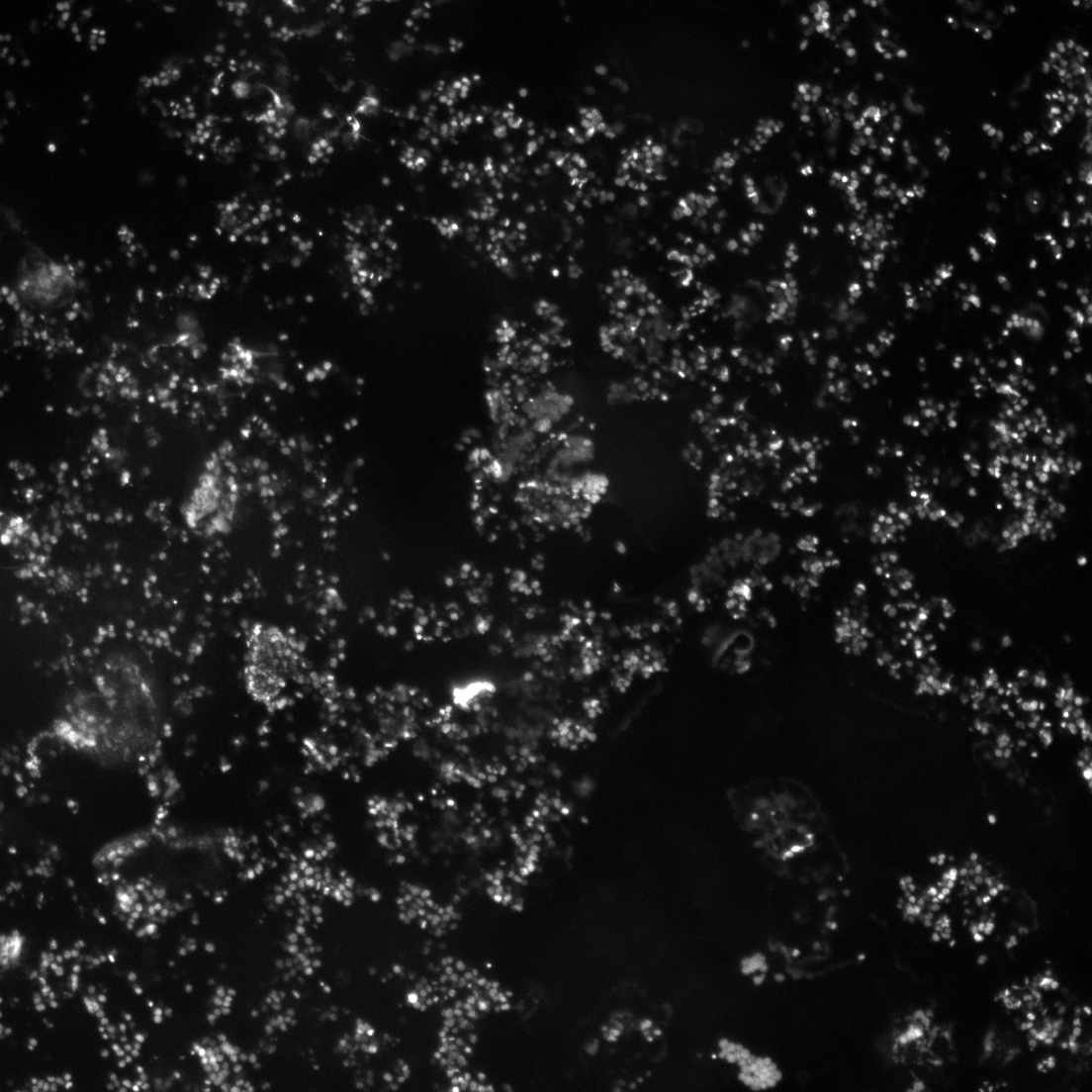

Supplement: Supplementary file 8 — Source Data for Figure 6 [file EMBJ-41-e111653-s003.zip › panel C/S/nuclei.TIFF]

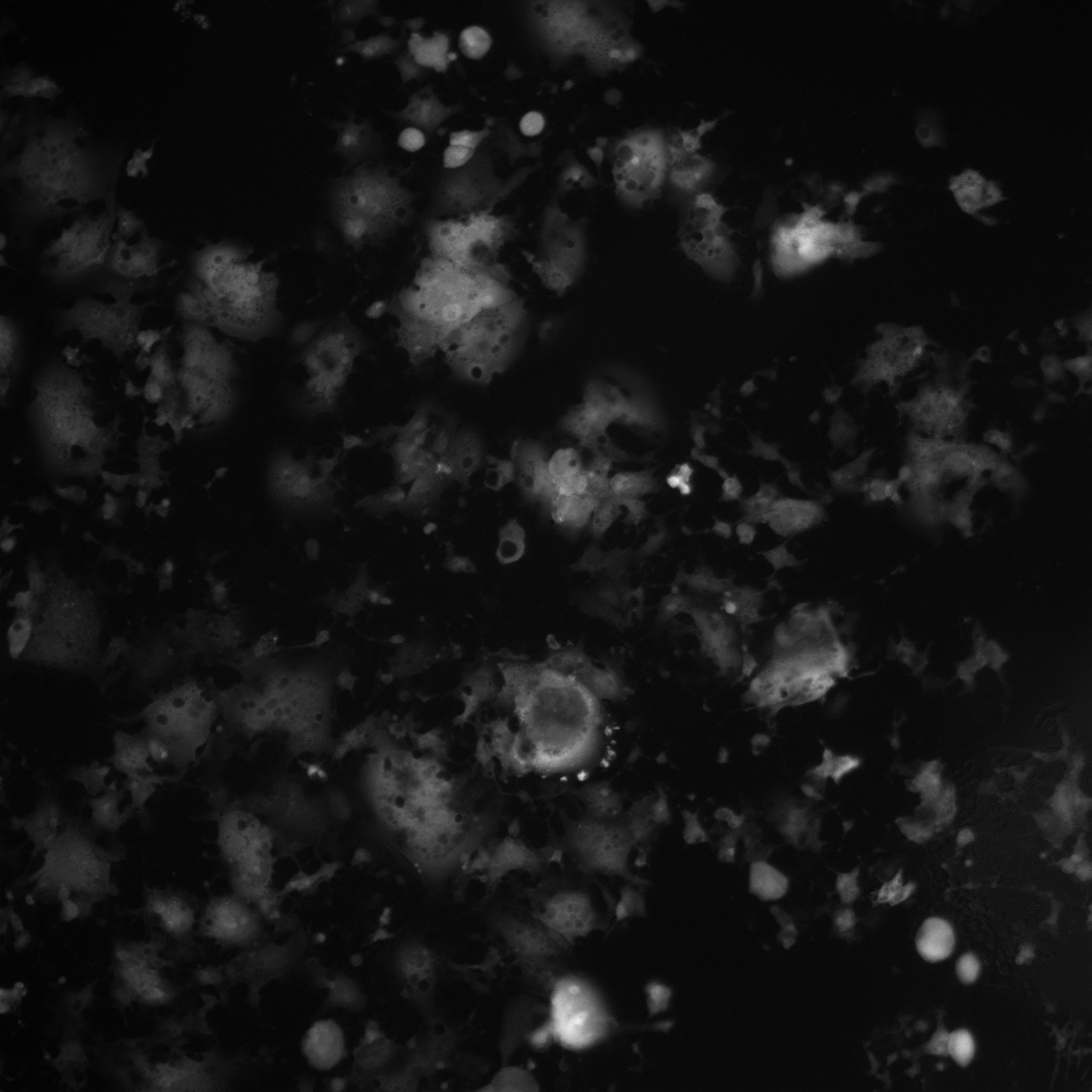

Supplement: Supplementary file 8 — Source Data for Figure 6 [file EMBJ-41-e111653-s003.zip › panel D/S D3/GFP.tif]

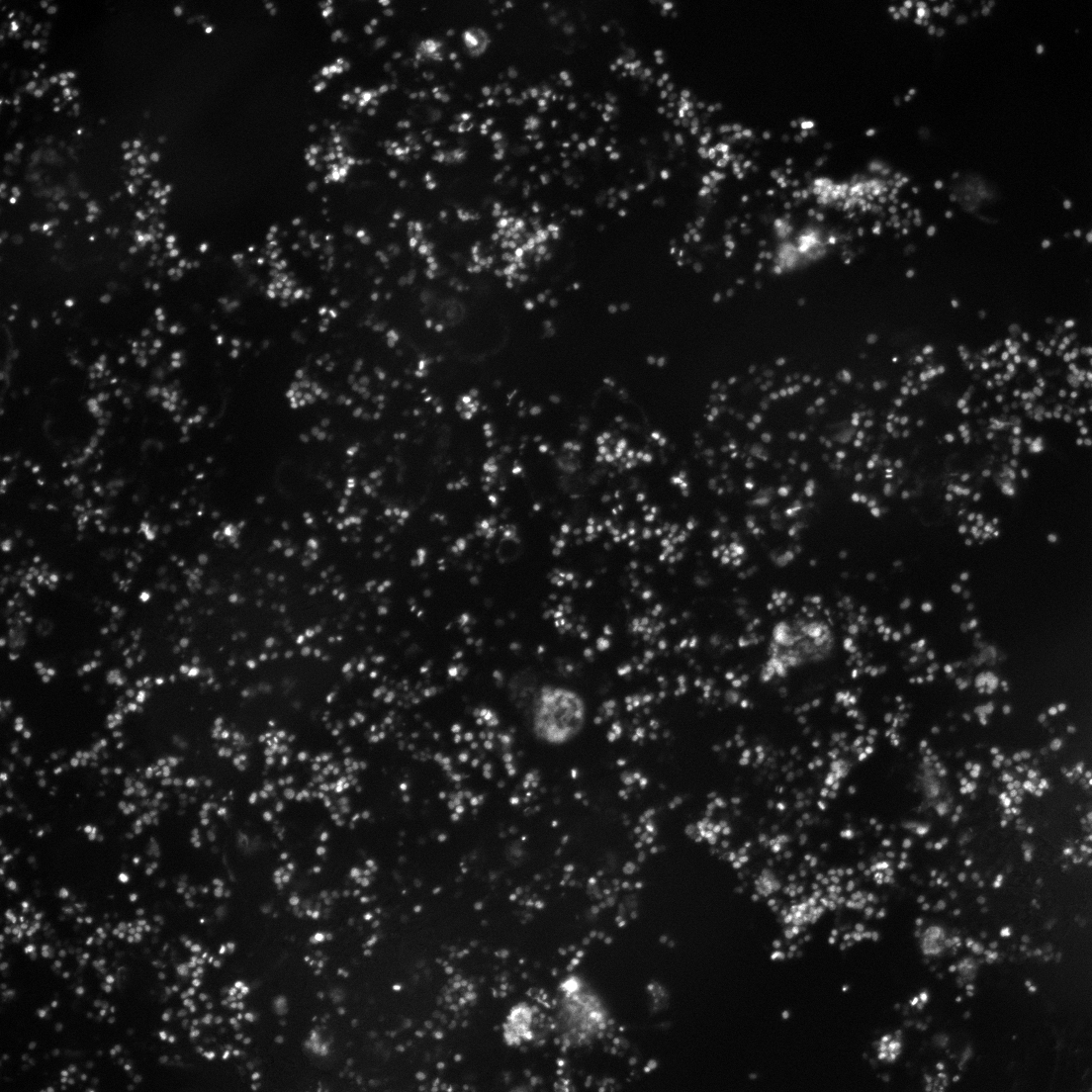

Supplement: Supplementary file 8 — Source Data for Figure 6 [file EMBJ-41-e111653-s003.zip › panel D/S D3/nuclei.tif]

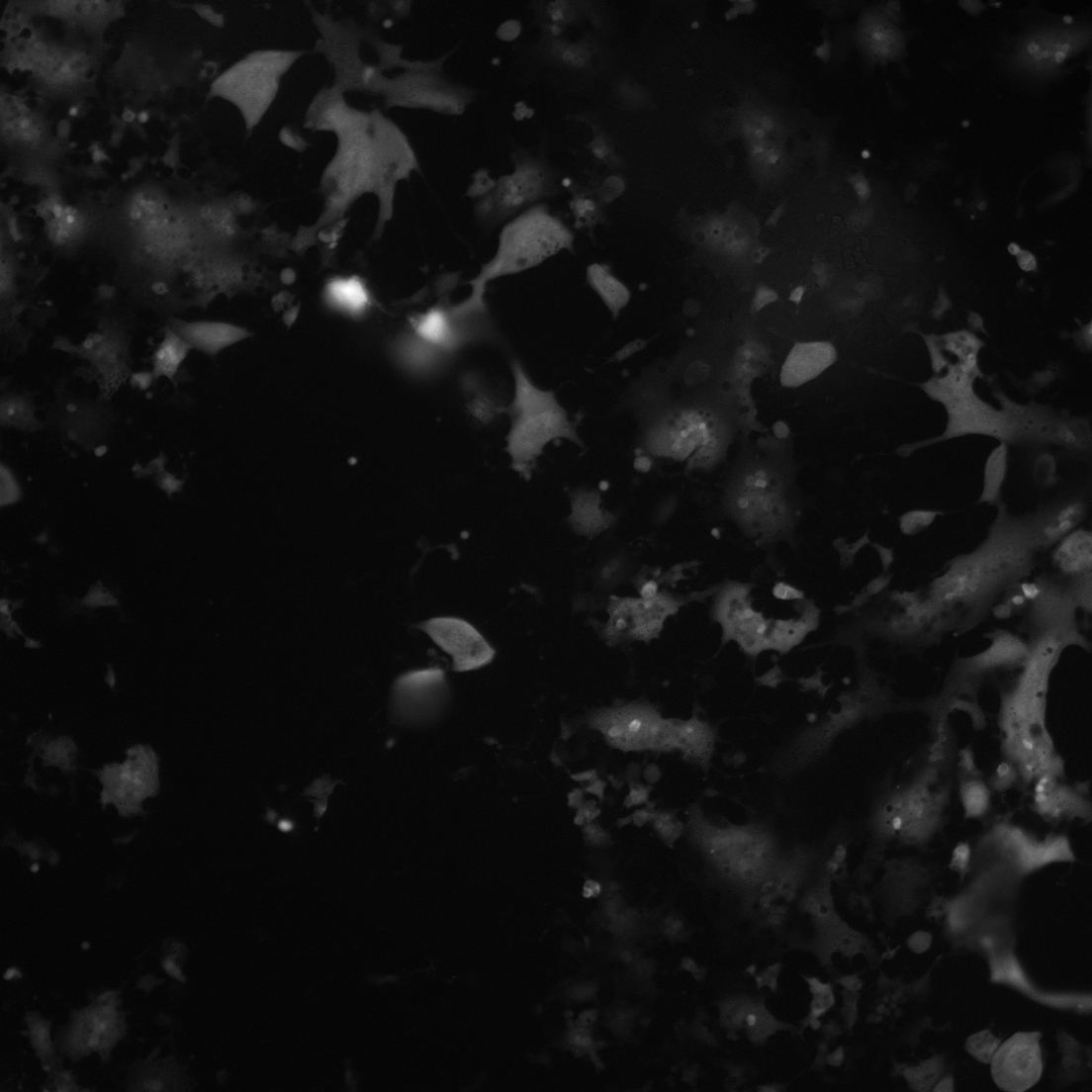

Supplement: Supplementary file 8 — Source Data for Figure 6 [file EMBJ-41-e111653-s003.zip › panel D/SMED3/GFp.tif]

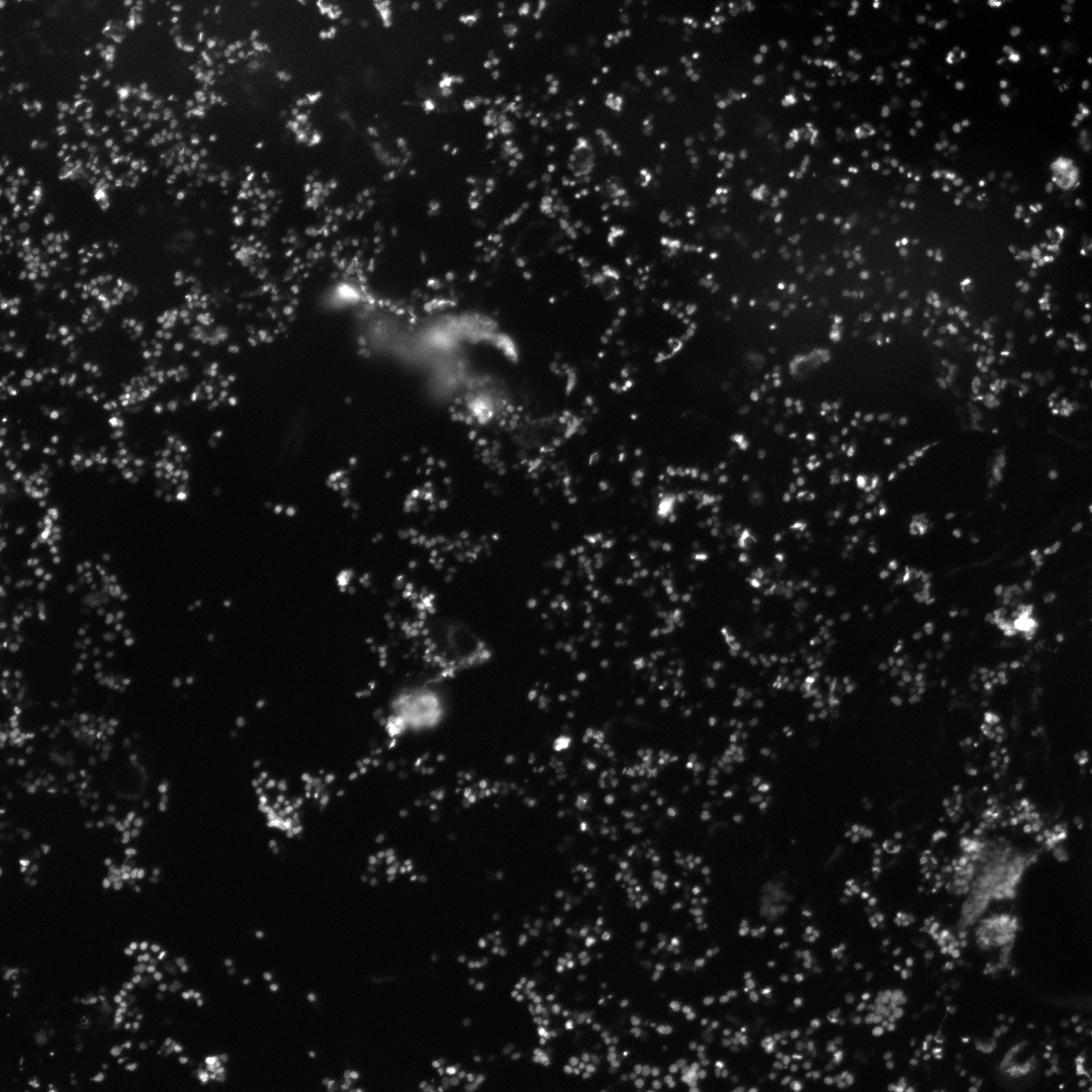

Supplement: Supplementary file 8 — Source Data for Figure 6 [file EMBJ-41-e111653-s003.zip › panel D/SMED3/nuclei.tif]

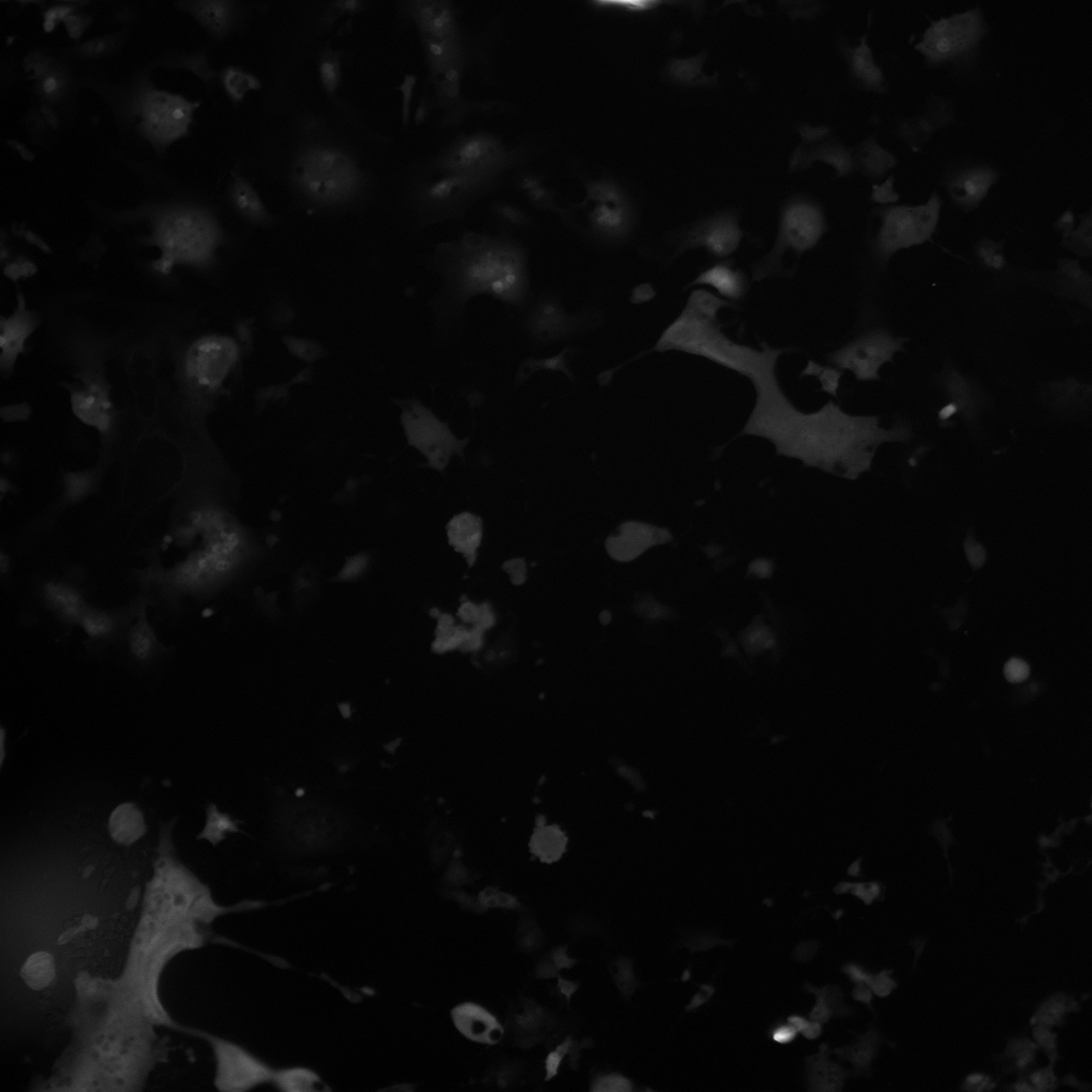

Supplement: Supplementary file 8 — Source Data for Figure 6 [file EMBJ-41-e111653-s003.zip › panel D/SME/GFP.tif]

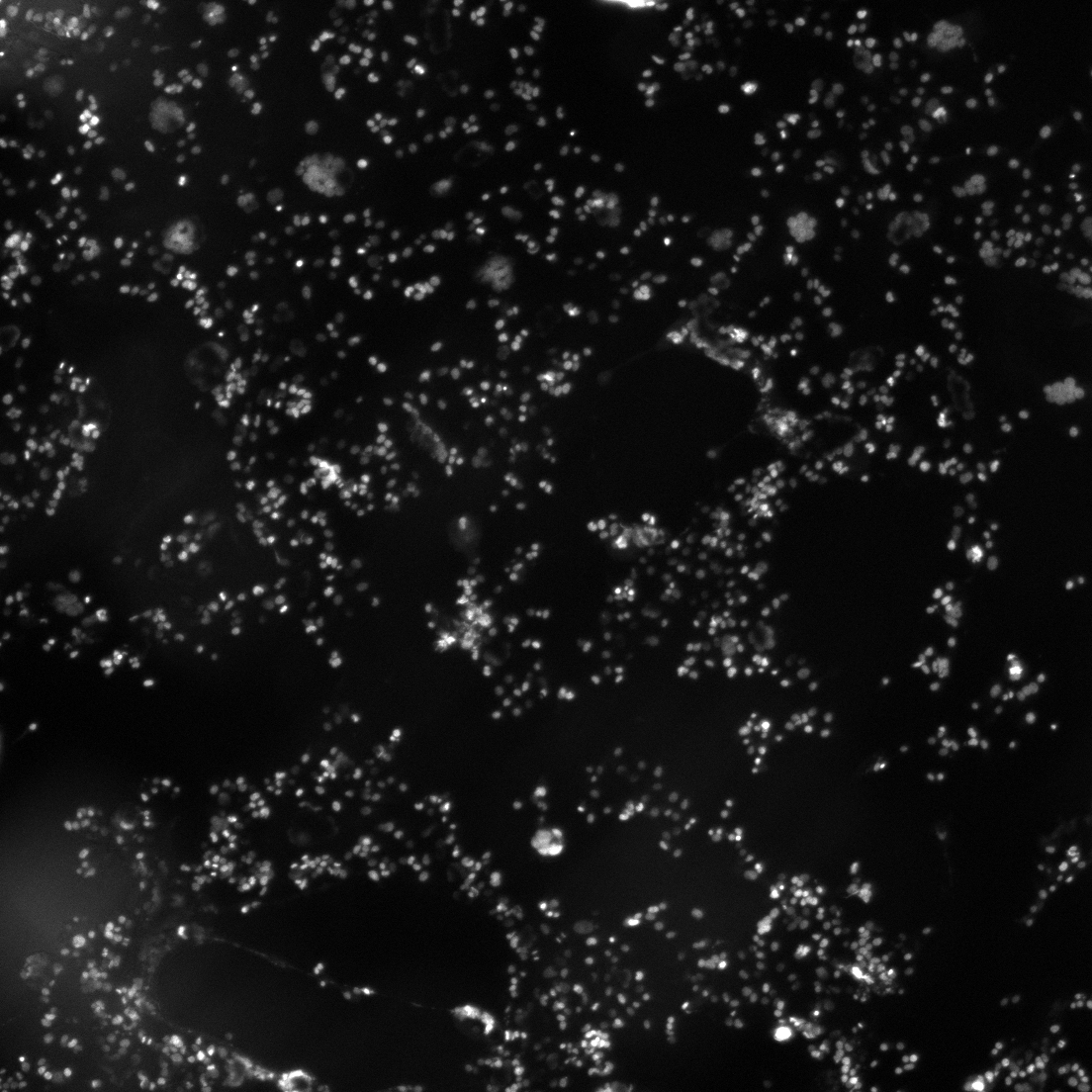

Supplement: Supplementary file 8 — Source Data for Figure 6 [file EMBJ-41-e111653-s003.zip › panel D/SME/nuclei.tif]

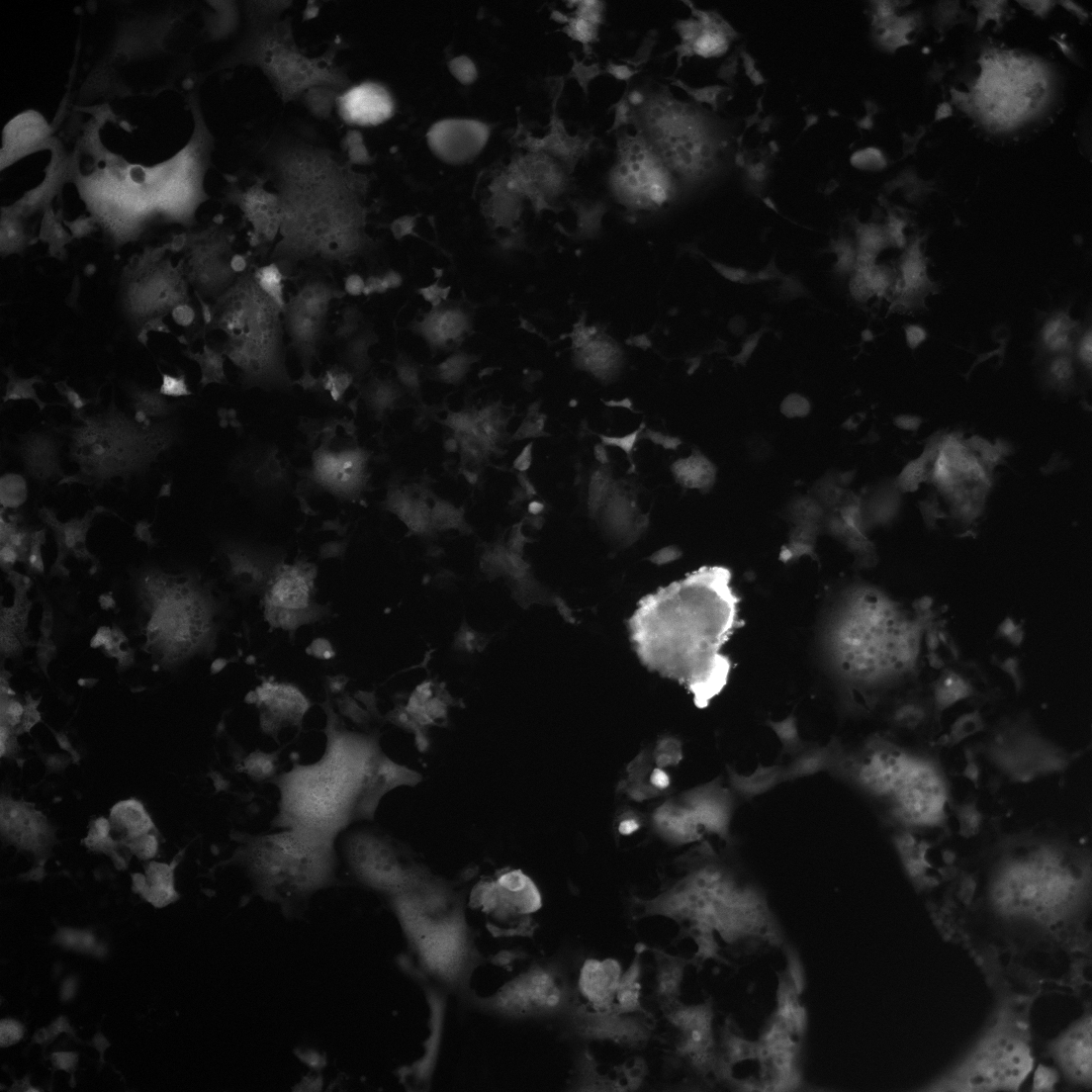

Supplement: Supplementary file 8 — Source Data for Figure 6 [file EMBJ-41-e111653-s003.zip › panel D/S/GFP.tif]

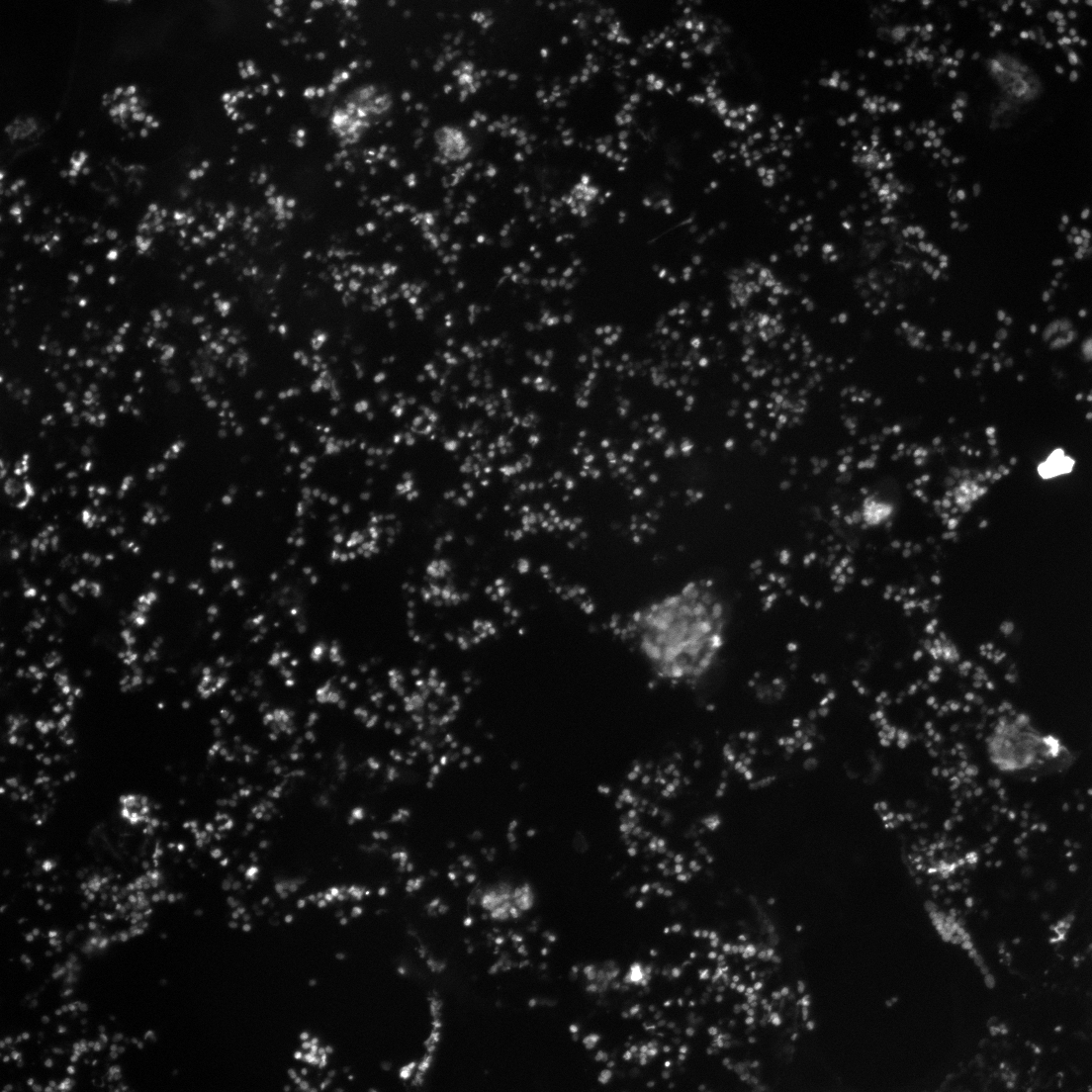

Supplement: Supplementary file 8 — Source Data for Figure 6 [file EMBJ-41-e111653-s003.zip › panel D/S/nuclei.tif]
