# Supplementary material for: Cyclin D3 restricts SARS‐CoV‐2 envelope incorporation into virions and interferes with viral spread
Source: EMBO J. 2022 Oct 10;41(22):e111653. doi: 10.15252/embj.2022111653 (PMC9539236; doi:10.15252/embj.2022111653)
Supplement: Supplementary file 9 — PDF+ [file EMBJ-41-e111653-s007.pdf]

Article

SOURCE DATA | TRANSPARENT PROCESS | OPEN ACCESS

THE EMBO JOURNAL

# Cyclin D3 restricts SARS-CoV-2 envelope incorporation into virions and interferes with viral spread

Ravi K Gupta[1,2,3] & Petra Mlcochova[1,2,*]

## Abstract

**The COVID-19 pandemic caused by severe acute respiratory syndrome coronavirus 2 (SARS-CoV-2) presents a great threat to human health. The interplay between the virus and host plays a crucial role in successful virus replication and transmission. Understanding host–virus interactions are essential for the development of new COVID-19 treatment strategies. Here, we show that SARS-CoV-2 infection triggers redistribution of cyclin D1 and cyclin D3 from the nucleus to the cytoplasm, followed by proteasomal degradation. No changes to other cyclins or cyclin-dependent kinases were observed. Further, cyclin D depletion was independent of SARS-CoV-2-mediated cell cycle arrest in the early S phase or S/G2/M phase. Cyclin D3 knockdown by small-interfering RNA specifically enhanced progeny virus titres in supernatants. Finally, cyclin D3 co-immunoprecipitated with SARS-CoV-2 envelope (E) and membrane (M) proteins. We propose that cyclin D3 impairs the efficient incorporation of envelope protein into virions during assembly and is depleted during SARS-CoV-2 infection to restore efficient assembly and release of newly produced virions.**

**Keywords** assembly; cell cycle; cyclin D3; Fucci; SARS-CoV-2
**Subject Category** Microbiology, Virology & Host Pathogen Interaction
**DOI** 10.15252/embj.2022111653 | Received 12 May 2022 | Revised 13 September 2022 | Accepted 15 September 2022 | Published online 10 October 2022
**The EMBO Journal (2022) 41: e111653**

## Introduction

The severe acute respiratory syndrome coronavirus 2 (SARS-CoV-2) is the causative agent for the global Covid-19 pandemic. To date, SARS-CoV-2 has infected over 265 million of people with a death toll of more than 5 million people (WHO Coronavirus (COVID-19) Dashboard; https://covid19.who.int/). While strategies of counteracting SARS-CoV-2 infection through vaccination have been partially successful, there is still a need for effective antiviral drugs given the emergence of vaccine escape variants such as Omicron. Coronaviruses, including SARS-CoV-2, like other viruses, are intracellular pathogens exploiting the host cell machinery to their own advantage. The identification of cellular mechanisms and host cell targets required for the SARS-CoV-2 life cycle will provide us with new knowledge that could be used to interfere with viral replication and therefore presents an alternative approach to block viral infection.

Cyclins and cyclin-dependent kinases (CDKs) are the major regulators of cell cycle progression. Many viruses, including coronaviruses, adopt a strategy of manipulating cell cycle progression through cyclin-CDKs complexes (Chen *et al*, 2004; Chen & Makino, 2004; Harrison *et al*, 2007; Sun *et al*, 2018) to facilitate viral replication. Several SARS-CoV-1 proteins have been shown to reduce cyclin D and cyclin E and A expression that is connected to cell cycle arrest (Surjit *et al*, 2006; Yuan *et al*, 2006, 2007). For example, SARS-CoV-1 N protein directly interacts with cyclin D to prolong the S phase (Surjit *et al*, 2006) that ensure enough supply of nucleotides for viral replication. Nsp13 protein both in SARS-CoV-1 and infectious bronchitis virus (IBV) interacts with DNA polymerase subunit to induce DNA damage and cell cycle arrest (Xu *et al*, 2011). It is believed that the virus infection-associated cell cycle arrest increases essential DNA repair processes and replication proteins that are required by virus replication.

A recent study showed that SARS-CoV-2 infection is correlated with cell arrest at S/G2 transition based on a comparison of phosphoproteomic profiles of SARS-CoV-2-infected VERO E6 cells and phosphorylation profiles collected at specific cell cycle phases. Further, by measuring DNA content, an increase in the fraction of cells in S and G2/M phases with a decreased proportion of cells in G0/G1 phase has been observed. Additionally, kinase activity profiling uncovered that CDK1/2 activities are reduced by SARS-CoV-2, possibly adding to S/G2 phase arrest (Bouhaddou *et al*, 2020).

Here, we present additional comprehensive data on SARS-CoV-2 cell cycle changes and identified cyclin D3 as a novel interactor with SARS-CoV-2 viral proteins. We propose that SARS-CoV-2 effectively reduces cyclin D3 levels in infected cells to achieve efficient viral assembly.

1 Cambridge Institute of Therapeutic Immunology & Infectious Disease (CITIID), Cambridge, UK
2 Department of Medicine, University of Cambridge, Cambridge, UK
3 Africa Health Research Institute, Durban, South Africa
*Corresponding author. Tel: +44 1223331497; E-mail: pm685@cam.ac.uk

© 2022 The Authors. Published under the terms of the CC BY 4.0 license.

## Results

### SARS-CoV-2 infection depletes cyclin D1 and D3

Several coronaviruses are known to regulate cell cycle and cell-cycle-associated proteins, including cyclins (Chen *et al*, 2004; Chen & Makino, 2004; Yuan *et al*, 2006, 2007; Harrison *et al*, 2007; Li *et al*, 2007). To determine whether the reduction in cyclins can occur during productive SARS-CoV-2 infection, the abundance of cyclins and cell-cycle-associated proteins (Fig 1) from SARS-CoV-2-infected cells was compared to uninfected VERO AT2 cells (Fig 1B–D) and human epithelial cell line A549 AT2 (Fig 1E and F). Western blots (Fig 1B and E) and densitometric analysis (Fig EV1A and B) indicate that cyclin D1 and D3 levels are significantly reduced compared to uninfected cells or cells infected with heat-inactivated virus.

Several other cyclins tested did not show any changes in expression nor did cell-cycle-associated kinases that form a functional complex with cyclins and regulate together cell cycle (Fig 1A and B).

We examined the cellular distribution of cyclins D1, D3 and A2 in infected VERO AT2 cells by immunofluorescence analysis (Figs 1C and EV1C). The ratio between the fluorescence intensity of cyclins in the nucleus and cytoplasm (N/C ratio) indicates that while uninfected cells localise cyclin D1/D3 predominantly in the nucleus (higher ratios), these cyclins are relocalised to the cytoplasm in infected cells (lower ratios) (Fig 1C, D, and F). Importantly, cyclin A2 was not degraded in infected cells (Fig 1B), and did not differ in cellular localisation between infected and uninfected cells. Relocation of cyclin D3 was also confirmed in HeLa cells expressing ACE2 (Appendix Fig S1). Importantly, cyclin D3 degradation was confirmed in the Calu3 lung cell line endogenously expressing ACE2/TMPRSS2 (Fig EV1D).

These data indicate that a productive SARS-CoV-2 infection leads to both reduced levels of cyclin D1 and D3 and their cellular relocalisation.

### Proteasome inhibition abolishes the effect of SARS-CoV-2 infection on D-cyclins depletion

It is known that D-cyclins are degraded mainly through the ubiquitin-dependent 26S proteasomal degradation pathway (Diehl *et al*, 1997; Casanovas *et al*, 2004). To further investigate the mechanism of D-cyclins depletion during SARS-CoV-2 infection, we investigated the involvement of the proteasome degradation pathway. Cells were infected in the absence or presence of protease inhibitors MG-132 and Bortezomib and D-cyclin levels were subsequently measured (Fig 2; Appendix Fig S2). Firstly, the addition of both inhibitors before or early after infection blocked SARS-CoV-2 infection (Appendix Fig S2A). This is in concordance with the published inhibitory effect of MG-132 on SARS-CoV-2 Mpro (Wang *et al*, 2021). In view of these results, A459 AT2 cells were infected with Delta SARS-CoV-2 variant for 24 h, before the addition of the proteasome inhibitor Bortezomib for an additional 24 h. Uninfected cells treated with the same drug showed an increase in D-cyclin levels, suggestive of cyclin stabilisation in cells. This increase was more pronounced in the case of cyclin D1 than in D3 which did not reach statistically significant levels (Fig 2A and B). Importantly, in the absence of proteasome inhibition, D-cyclins relocalised from the nucleus to cytoplasm and were degraded in infected cells. But proteasome inhibition significantly stabilised D-cyclins levels in infected cells where D-cyclins expression levels were equal to those in uninfected cells (Fig 2A and B; Appendix Fig S2B and C). Further, proteasome inhibition also prevented relocalisation of cyclin D3 to the cytoplasm in SARS-CoV-2-infected VERO AT2 cell (Appendix Fig S2B and C). Even though the proteasome inhibition did not completely prevent the translocation of cyclin D3 in infected A549 AT2 cells, it revealed a significant presence of cyclin D3 in the nucleus (Fig 2C and D). These data indicate that the D-cyclin depletion during SARS-CoV-2 infection is mediated by a proteasome-dependent pathway.

### Cyclin D3 negatively regulates SARS-CoV-2 infection

To understand the functional role of D-cyclins in SARS-CoV-2 pathogenesis, the effect of cyclin knockdown on viral replication was investigated. siRNA effectively reduced levels of D and A2 cyclins by > 80% at 48 h post-transfection, compared to non-targeting control (NT) in A549 AT2 cells (Fig 3A; Appendix Fig S3A and B) or VERO AT2 (Fig 3C). Cells depleted for individual cyclins were infected with Delta, Alpha and WT variant to allow multiple rounds of infection (Fig 3; Appendix Fig S3). Interestingly, only viral titres from cyclin D3-depleted cells were significantly higher than those from control (NT). This was evident for all SARS-CoV-2 variants tested in both A549 AT2 and VERO AT2 cells (Fig. 3B and D) and confirmed in Calu3 lung cells (Fig 3E) endogenously expressing ACE2/TMPRSS2. These data support the notion that cyclin D3 negatively modulates SARS-CoV-2 infection and could potentially have an impact on the viral spread.

To exclude the possibility that cyclin D3 depletion affects genomic replication, we depleted cyclin D3 in VERO AT2 cells and infected these cells with SARS-CoV-2 for 8 h, allowing one round of infection, and for 24 h, allowing multiple (~ 3) rounds of infection. RNA was isolated from cells and qPCR detecting nucleocapsid transcripts was performed. If genome replication was affected, we would expect a change in nucleocapsid transcripts in the cells, even after one round of infection. We did not detect any differences between cells expressing and cells depleted for cyclin D3 at either 8 or 24 h (Appendix Fig S3C).

### Productive SARS-CoV-2 infection induces cell cycle arrest

Cyclin D1, D2 and D3 are important regulators of G1 to S phase progression. Given the observed depletion of cyclin D in SARS-CoV-2-infected cells, we speculated that this depletion might affect cell cycle progression. Indeed, SARS-CoV-2-mediated S/G2 cell cycle arrest has been reported recently in non-human VERO E6 cells (Bouhaddou *et al*, 2020) but its role in human cells is unknown. To understand the role between infection and D-cyclin depletion in cell cycle regulation, we firstly aimed to examine previously reported cell cycle arrest phenotype. We used the fluorescence ubiquitination cell cycle indicator (Fucci) cell cycle sensor (Fig EV2; Sakaue-Sawano *et al*, 2008) in VERO AT2 and A549 AT2 cells. VSV-G pseudotyped viral particles containing Fucci sensor were used to transduce cells. Cells were then infected with a replication-competent strain of SARS-CoV-2 for 24 h, fixed and stained for nucleocapsid.


© 2022 The Authors

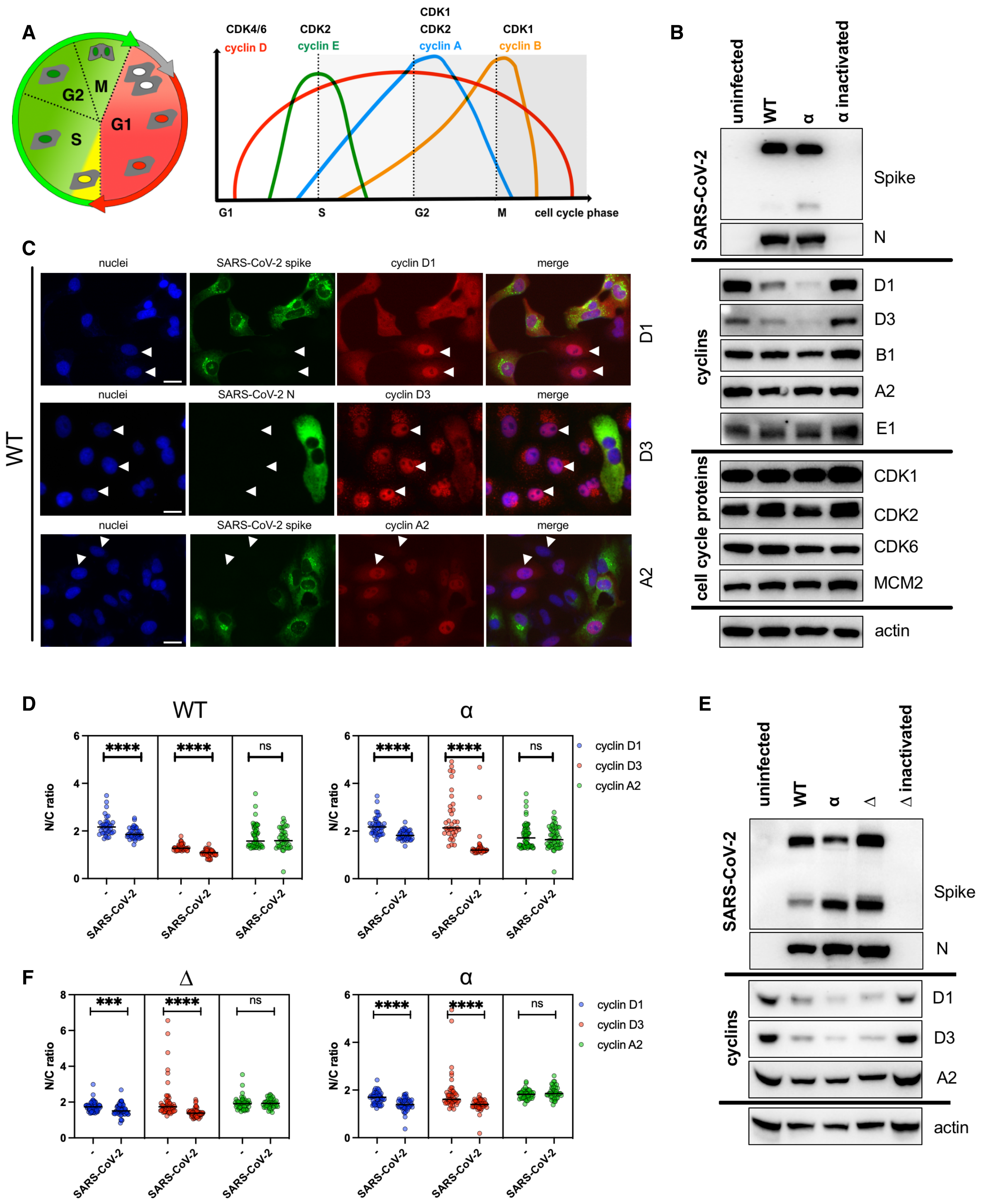
A
G2
M
G1
S
CDK4/6
cyclin D
CDK2
cyclin E
CDK1
CDK2
cyclin A
CDK1
cyclin B
G1
S
G2
M
cell cycle phase
B
uninfected
WT
α
α inactivated
SARS-CoV-2
Spike
N
cyclins
D1
D3
B1
A2
E1
cell cycle proteins
CDK1
CDK2
CDK6
MCM2
actin
C
WT
nuclei
SARS-CoV-2 spike
cyclin D1
merge
D1
SARS-CoV-2 N
cyclin D3
D3
cyclin A2
A2
D
WT
α
N/C ratio
****
ns
SARS-CoV-2
cyclin D1
cyclin D3
cyclin A2
E
Δ
Δ inactivated
F
***

Figure 1.

◀

**Figure 1. SARS-CoV-2 infection depletes cyclin D1 and D3.**

A Diagram of cell cycle and cyclin expression during the cell cycle. Cyclins drive cell cycle changes by interacting with cyclin-dependent kinases (CDKs).

B, C VERO AT2 cells were infected with WT and Alpha (α) SARS-CoV-2-live virus variants, and heat-inactivated α variant at MOI 0.1. (B) Cells were lysed 48 h post-infection and viral protein, as well as cell-cycle-associated protein expression, was analysed by western blot. N, nucleocapsid. (C) VERO AT2 cells were fixed 24 h post-infection and stained for viral proteins and cyclins. Arrowheads highlight uninfected cells and cyclin D/A nuclear localisation. Arrowheads: Nuclear cyclin staining in uninfected cells. Scale bars: 20 μm.

D VERO AT2 cells. Quantification of cyclin A2, D1 and D3 relocalisation from nucleus after infection. Uninfected (−) and SARS-CoV-2-infected cells were identified by negative/positive nucleocapsid or spike staining. The ratio between nuclear and cytoplasm staining intensity of cyclins was measured using ImageJ and Harmony (PerkinElmer). At least 50 cells were counted. Bars indicate the mean with SD. Statistical analysis was performed using two-sided unpaired Student's *t*-tests; ns, non-significant; ****$P < 0.0001$.

E A549 AT2 cells were infected with WT, Alpha (α) and Delta (Δ) SARS-CoV-2 variants. Cells were lysed 48 h post-infection and viral and cyclin proteins expression was analysed by western blot. N, nucleocapsid.

F A549 AT2 cells. Quantification of cyclin A2, D1 and D3 relocalisation from nucleus after infection. Uninfected (−) and SARS-CoV-2-infected cells were identified by negative/positive nucleocapsid or spike staining. The ratio between nuclear and cytoplasm (N/C ratio) staining intensity of cyclins was measured using ImageJ and Harmony (PerkinElmer). At least 50 cells were counted. Bars indicate the mean with SD. Statistical analysis was performed using two-sided unpaired Student's *t*-tests; ns, non-significant; ***$P < 0.001$; ****$P < 0.0001$.

Source data are available online for this figure.

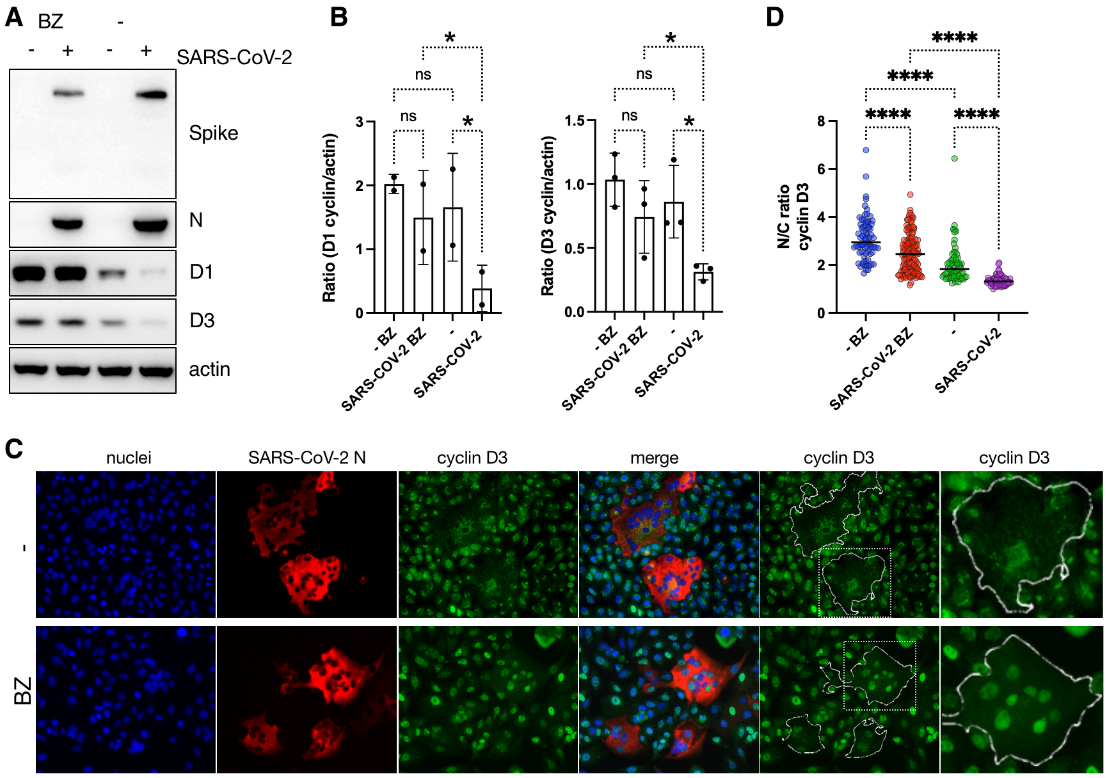


**Figure 2. Proteasome inhibition abolishes the effect of SARS-CoV-2 infection on D-cyclins depletion.**

A A549 AT2 cells were infected with Delta SARS-CoV-2 variant. Proteasome inhibitor Bortezomib (BZ, 1 μM) was added to cells 18 h post-infection. Cells were lysed 24 h post-addition of inhibitor and cyclins and viral proteins were detected by western blot. (N) nucleocapsid.

B Densitometry analysis of western blots for D-cyclins (normalised to actin) in A549 AT2 cells. Plots are an average of two (cyclin D1) and three (cyclin D3) biological replicates. Bars indicate the mean with SD. Statistical analysis was performed using ordinary two-way ANOVA; ns, non-significant; *$P < 0.1$.

C A459 AT2 cells were infected with Delta SARS-CoV-2 variant. Proteasome inhibitor Bortezomib (BZ, 1 μM) was added to cells 8 h post-infection. Cells were fixed and stained 24 h post-addition of inhibitor. (N) Nucleocapsid. Scale bars: 40 μm. Contour lines represent outlines of infected cells.

D Quantification of D3 cyclin relocalisation from nucleus after infection. Uninfected (−) and SARS-CoV-2-infected cells were identified by negative/positive nucleocapsid staining. The ratio between nuclear and cytoplasm (N/C ratio) staining intensity of cyclins was measured using ImageJ and Harmony (PerkinElmer). At least 50 cells have been counted. Bars indicate the mean with SD. Statistical analysis was performed using ordinary two-way ANOVA; ***$P < 0.001$.

Source data are available online for this figure.

 © 2022 The Authors

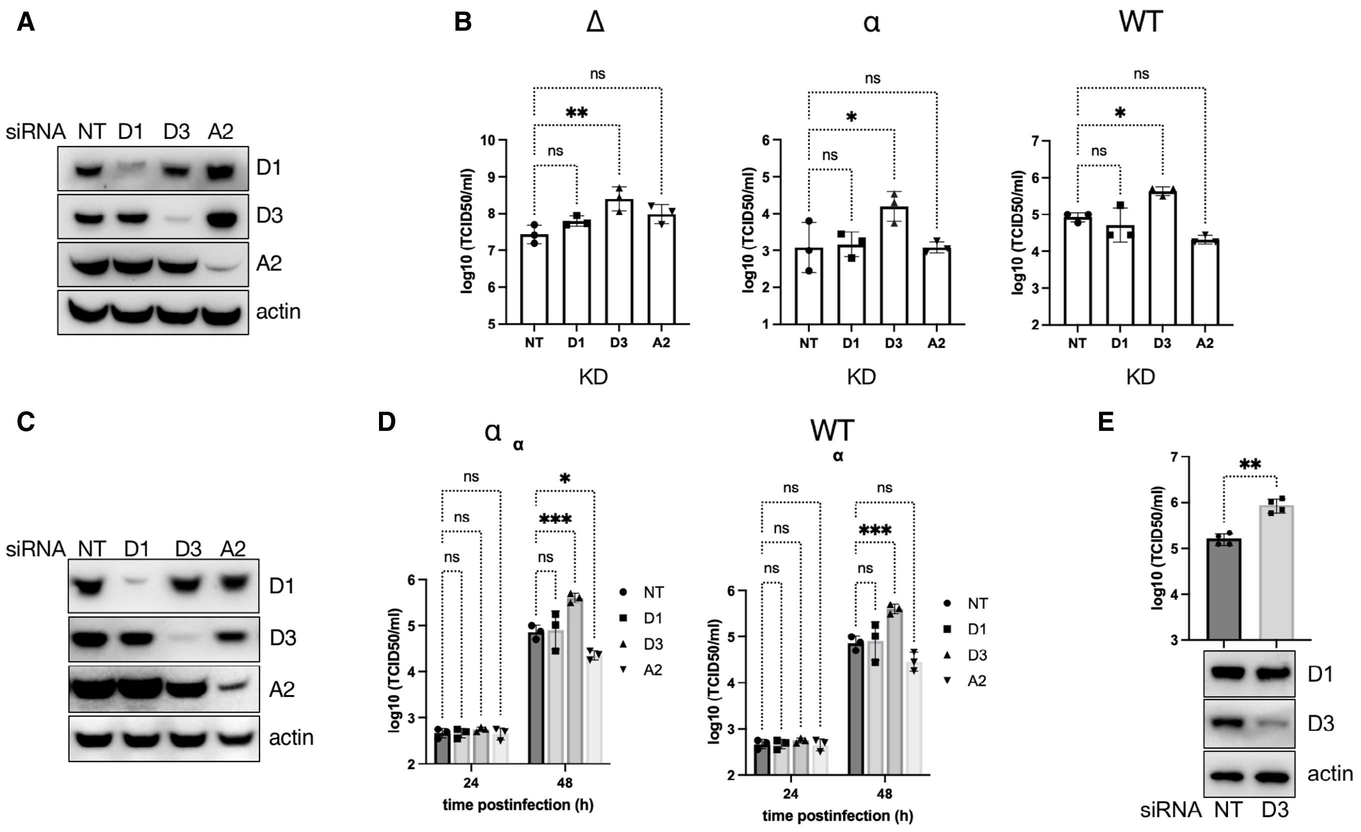


**Figure 3. Cyclin D3 impairs SARS-CoV-2 spreading infection.**

A–D (A, B) D and A-cyclins were depleted using siRNA in A549 AT2 cells. Cells were infected 18 h later with Delta (Δ), Alpha (α) or wild-type (WT) SARS-CoV-2 variants at MOI 0.001, 0.1 and 0.1 respectively. Cells were washed 4 h post-infection and new media were added. Supernatants and cells were collected 48 h later for western blots (A) and TCID50 (B). (C, D) D and A-cyclins were depleted using siRNA in VERO AT2 cells. Cells were infected 18 h later with Alpha (α) or wild-type (WT) SARS-CoV-2 variants at MOI 0.1. Cells were washed 4 h post-infection and new media were added. Supernatants and cells were collected at 24 h and 48 h later. (A, C) Representative example of western blot from uninfected cell lysates collected at 72 h post-knockdown. (B, D) Virus titres in cell culture supernatants were determined as TCID50 in VERO AT2 cells.

E Cyclin D3 was depleted using siRNA in Calu3 cells. Cells were infected 48 h later with Delta SARS-CoV-2 at MOI 0.01. Cells were washed 4 h post-infection and new media were added. Supernatants and cells were collected 48 h later for western blot and TCID50. Uninfected cell lysates were used for the western blot.

Data information: Graphs represent the average of $n = 3$ (B, D); $n = 4$ (E) biological replicates. Statistical analysis was performed using one-way ANOVA with Dunnett's multiple comparisons test. NT, non-targeting control. ns, non-significant; *$P < 0.1$; **$P < 0.01$; ***$P < 0.001$. Bars indicate the mean with SD.
Source data are available online for this figure.

Flow cytometry analysis was used to visualise G1, early S and S/G2/M phases. The Fucci system cannot demonstrate the G0 phase as it is defined as a cell population void of any fluorescent protein but cannot be differentiated from an untransduced cell population. Cells were gated on infected (expressing SARS-CoV-2 nucleocapsid) or uninfected cells with Cdt1/geminin expression determined in both gated populations and compared (Fig 4A–D). Indeed, SARS-CoV-2 infection mediated cycle arrest in S/G2/M phase in VERO AT2 cells (Fig 4C), confirming previously published data (Bouhaddou *et al*, 2020). Interestingly, SARS-CoV-2-mediated cell cycle arrest in A549 AT2 cells was identified specifically in the early S phase (Fig 4D). This cell cycle arrest was caused by productive SARS-CoV-2 infection as use of heat-inactivated virus or Remdesivir (RDV) treatment abrogated cell cycle arrest (Fig EV2D–F) and population of arrested cells increased with increasing MOI (Fig EV2G and H). All SARS-CoV-2 variants tested (WT, Alpha and Delta) caused cell cycle arrest in S/G2/M phase in VERO AT2 cells and early S phase in A549 AT2 and (Fig 4E and F). Furthermore, cell cycle kinetics in virus-exposed but uninfected cells were similar to unexposed uninfected cells (Fig EV2I and J).

These observations support the hypothesis that a productive SARS-CoV-2 infection is responsible for cell cycle arrest and that the arrest is not the result of by-stander effects.

### Cell cycle arrest induced by SARS-CoV-2 is not dependent on cyclin D3 degradation

To dissect whether the increase in viral titre is a direct consequence of cylin D depletion or the aftermath of cell cycle arrest caused by the absence of this cyclin, we investigated cell cycle arrest during SARS-CoV-2 infection and linked it to cyclin D expression/cellular localisation. It has been previously reported that cyclin D degradation is sufficient to cause cell cycle arrest in G1 phase (Agami & Bernards, 2000; Masamha & Benbrook, 2009). We confirmed cell

© 2022 The Authors

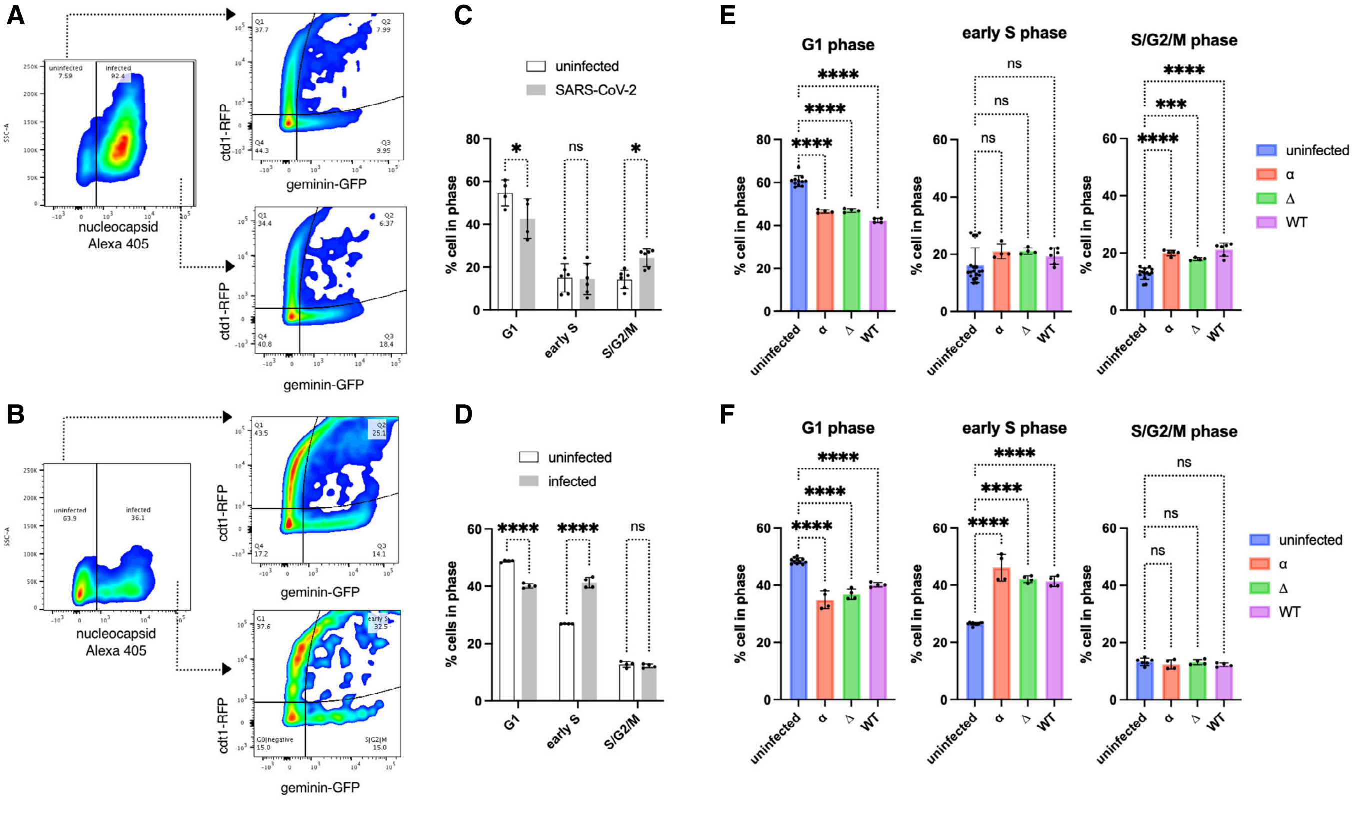


**Figure 4. SARS-CoV-2 infection arrests the cell cycle.**

Cells were transduced with Fucci-containing lentiviral particles for 18 h and infected with SARS-CoV-2 variants for an additional 24 h.

A, B (A) VERO AT2 or (B) A549 AT2 cells. Example of gating strategy for cell cycle analysis. The population of cells exposed to SARS-CoV-2 was stained for nucleocapsid (N) protein and gated on N+ve and N-ve populations. Cells were further analysed for expression of Cdt1 and Geminin. See also Appendix Fig S5.

C, D (C) VERO AT2 or (D) A549 AT2 cells infected with WT SARS-CoV-2. Quantification of Cdt1 + ve cells (G1), Cdt1/geminin +ve cells (early S phase) and geminin +ve cells (S, G2, M phase) cells. $n = 4$ biological replicates; one-way ANOVA with Dunnett's multiple comparisons test: ns, non-significant; *$P < 0.1$; ***$P < 0.001$; ****$P < 0.0001$. Bars indicate the mean with SD.

E, F (E) VERO AT2 or (F) A549 AT2 cells. Quantification of cell cycle arrest after exposure of cells to SARS-CoV-2 variants. α, Alpha (MOI 0.5); Δ, Delta (MOI 0.1); WT, Wuhan (MOI 0.5). $n = 4$ biological replicates; one-way ANOVA with Dunnett's multiple comparisons test: ns, non-significant; ****$P < 0.0001$; ***$P < 0.001$. Bars indicate the mean with SD.

cycle arrest in G1 phase in A549 AT2 after D-cyclin depletion (Appendix Fig S4A and B). Furthermore, VERO AT2 cells showed similar G1 arrest after individual D1 and D3 cyclin depletion but as well when both D1 and D3 cyclins were depleted together (Appendix Fig S4C). Importantly, no increase in the early S or S/G2/M phase has been observed after cyclin D depletion in uninfected cells. On the contrary, a decrease in these phases has been identified in concordance with more cells arresting in G1 phase and not progressing through the cell cycle (Appendix Fig S4). As we have already shown that SARS-CoV-2 infection increases the percentage of cells in early S (in A549 AT2 cells) and S/G2/M (in VERO AT2)-specific cell cycle phases (Fig 4), we investigated cell cycle progression in infected cells were D-cyclins and cyclin A had been depleted (Figs 5 and EV3). A549 AT2 cells have been depleted for D and A cyclins (Fig 5A) and infected with the Delta variant. The percentage of infected cells was determined 24 h later and a small increase (threefold) compared to NT control was detected in cells depleted for cyclin D3 (Fig 5B and C). The A549 AT2 cell population in early S increased without any changes in S/G2/M phase following infection in both non-targeted (NT) control cells and cells depleted for D and A cyclins (Fig 5D). These data were confirmed using an Alpha SARS-CoV-2 variant (Fig EV3A and B). Furthermore, cell cycle in virus-exposed but uninfected cells showed similar cell cycle kinetics as unexposed and uninfected cells excluding any by-stander effect of infection on cell cycle changes (Fig EV3C and D). Moreover, VERO AT2 cells showed an increase in the proportion of cells in S/G2/M phase after SARS-CoV-2 infection even when D and A cyclins were depleted, confirming our results in A549 AT2 cells (Fig EV3E and F).

Importantly, SARS-CoV-2 infection still caused cell cycle arrest in the early S phase in A549 AT2 even in the presence of high levels of the proteasome-resistant cyclin D3 mutant (Fig 5E). Mutation at T283 prevents phosphorylation, nuclear export and proteasomal degradation (Casanovas *et al*, 2004; Cato *et al*, 2011). Cells were transduced with lentivirus to deliver wild-type (WT) cyclin D3 or mutant T283A cyclin D3, before infection with live SARS-CoV-2. Cyclin D3 could be detected in non-transduced/control cells and was completely degraded during SARS-CoV-2 infection. Cells

 © 2022 The Authors

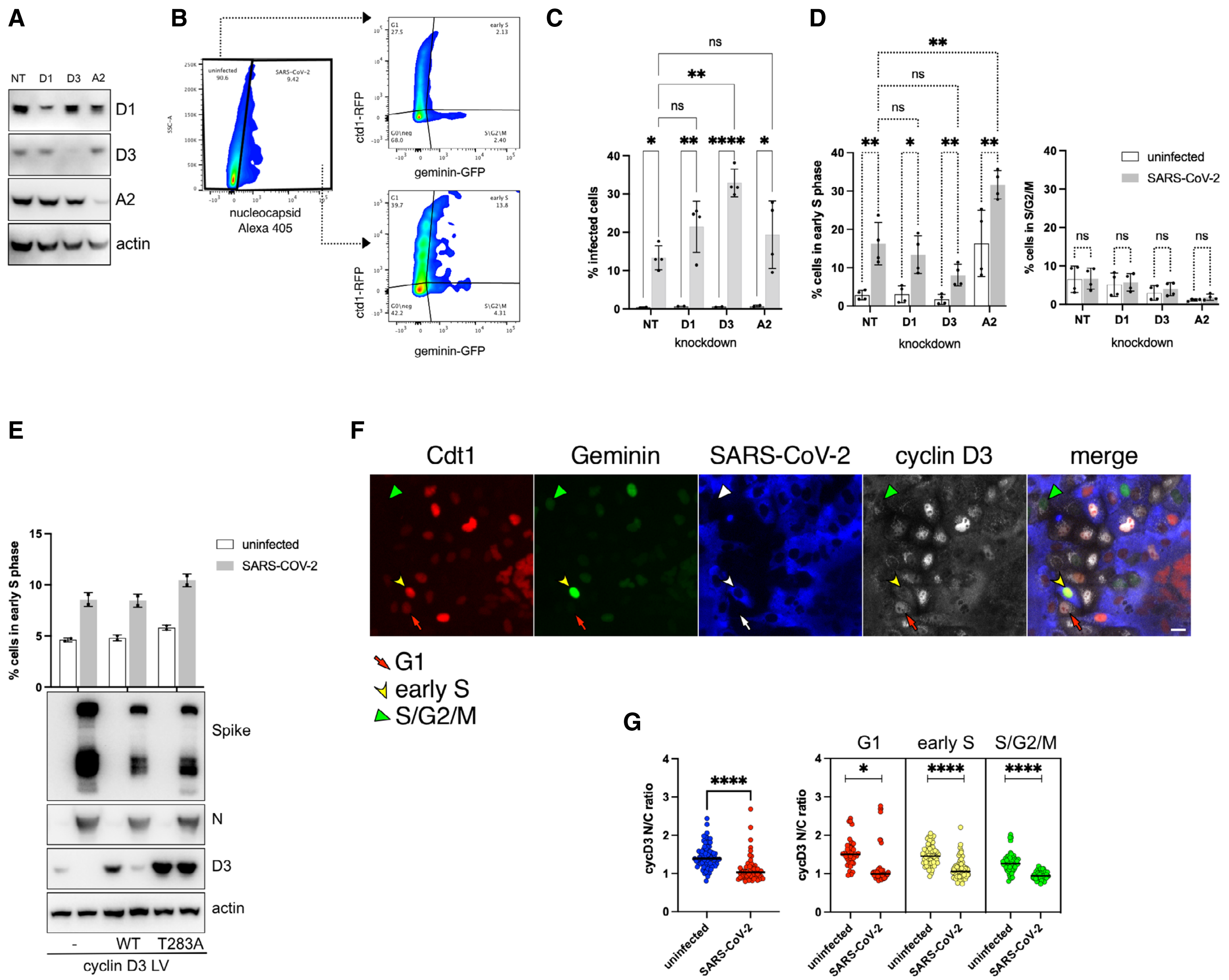


**Figure 5. SARS-CoV-2-mediated depletion of D-cyclins is cell cycle arrest independent.**

A–D A549 AT2 cells were depleted for D and A2 cyclins and 18 h later infected with Delta variant SARS-CoV-2 for 24 h. Cells were fixed, stained for SARS-CoV-2 nucleocapsid and analysed for infection and Fucci cell cycle sensor. (A) A representative western blot from lysates of uninfected knockdown cells. (B) Example of gating strategy for flow cytometry analysis. (C) Percentage of infected cells in cells depleted for cyclins. $n = 3$ biological replicates; ordinary two-way ANOVA with Sidak's multiple comparisons test: ns, non-significant; ****$P < 0.0001$; **$P < 0.01$; *$P < 0.1$. Bars indicate the mean with SD. (D) Flow cytometry analysis of early S and S/G2/M cell cycle phases comparing cyclin D1, D3 and A2 knockdown to NT (non-target siRNA). $n = 3$–4 biological replicates. Statistical analysis was performed using two-sided unpaired Student's $t$-tests; ns, non-significant; **$P < 0.01$; *$P < 0.1$. Bars indicate the mean with SD.

E A549 AT2 cells were transduced with VSV-G pseudotyped Fucci-containing lentiviral particles and VSV-G pseudotyped lentiviral particles containing WT cyclin D3 or T283A mutant cyclin D3 (mutant not degraded by proteasome). Cells were infected 24 h later and collected 48 h post-infection for flow cytometry analysis of early S and western blot. $n = 2$ biological replicates; Ordinary two-way ANOVA with Sidak's multiple comparisons test: ns, non-significant; **$P < 0.01$. Bars indicate the mean with SD.

F, G VERO AT2 cells were transduced with VSV-G pseudotyped Fucci-containing lentiviral particles and 18 h later infected with Delta variant SARS-CoV-2. Cells were fixed and stained for D-cyclins 24 h later. (F) Example of acquisition using the automated microscopic platform. Cells are identified for infection, expression of cyclin D3 and cell cycle (Red/arrow = G1phase; Green/arrowhead = S/G2/M; Red+Green/arrowhead = early S). Scale bar: 20 µm. (G) Quantification of D-cyclins relocalisation from the nucleus to cytoplasm and correlation with cell cycle phases using ImageJ and Harmony (PerkinElmer). At least 50–200 cells were analysed in each condition. Bars indicate the mean with SD. Statistical analysis was performed using two-sided unpaired Student's $t$-tests; *$P < 0.1$.

Source data are available online for this figure.

complemented with cyclin D3 WT and T283A showed higher cyclin D3 expression than controls as expected; however, after infection, only WT cyclin D3 was degraded while T283A cyclin D3 protein persisted. Moreover, even in the presence of high levels of T283A cyclin D3, cell cycle arrest in the early S phase was clearly evident (Fig 5E). Additionally, single-cell analysis showed that D-cyclins were relocated and degraded in SARS-CoV-2-infected cells independently of cell cycle phase. VERO AT2 cells were transduced with

© 2022 The Authors 

Fucci-containing lentiviral particles and 18 h later infected with Delta (Figs 5F and G, and EV3I) or Alpha (Fig EV3G and H) SARS-CoV-2 variant. Quantification of nuclear/cytoplasm ratio of D-cyclin staining in different cells cycle phases (identified by expression of Fucci sensor) clearly showed that cyclin D3 (Fig 5G) and cyclin D1 (Fig EV3I) in SARS-CoV-2-infected cells are relocalised from nucleus to cytoplasm probably for degradation, independent of the cell cycle phase they are in. These data suggest that cell cycle arrest induced by SARS-CoV-2 is not dependent on cyclin D3 degradation.

### Cyclin D3 associates with E and M SARS-CoV-2 proteins

Cyclin D3 has been previously implicated in the restriction of influenza A virus through impairment of virus assembly (Fan *et al*, 2017). Cyclin D3 has been shown to interact with IAV protein M2, an ion channel that promotes viral replication (Pinto & Lamb, 2006; Fan *et al*, 2017). Interestingly, SARS-CoV-2 envelope (E) protein has been suggested to be an ion channel (Singh Tomar & Arkin, 2020; Xia *et al*, 2021). In light of our data showing that cyclin D3 depletion increased SARS-CoV-2 viral titre, we investigated the potential implication of cyclin D3 in SARS-CoV-2 assembly. Firstly, the interaction between cyclin D3 and E protein was investigated using immunoprecipitation (Fig 6A). HA-tagged cyclin D3 was co-expressed together with Strep-tagged E and nsp9 protein in 293T cells. Nsp9 was chosen as a control on the basis of its diverse cellular localisation both in the nucleus and cytoplasm (Zhang *et al*, 2020). Of note, 293T cells showed undetectable endogenous expression of cyclin D3 (Fig EV4A). We showed that SARS-CoV-2 envelope (E) co-immunoprecipitated with HA-tagged cyclin D3 using anti-HA antibody while SARS-CoV-2 nsp9 protein did not (Fig 6A), which is suggestive of specific binding to E protein.

SARS-CoV-2 E protein is a known interactor of membrane (M) and nucleocapsid (N) proteins. Further, it has been shown for SARS-CoV-1 and SARS-CoV-2 that M, E and (N) structural proteins are required for efficient assembly (Siu *et al*, 2008; Yurkovetskiy *et al*, 2020; Kumar *et al*, 2021; Plescia *et al*, 2021). We expressed HA-tagged cyclin D3 with spike (S), or Strep-tagged N, M, E and nsp9 (Fig EV4). Co-immunoprecipitation with HA-tagged cyclin D3 using anti-HA antibody or anti-cyclin D3 mouse monoclonal antibody revealed an interaction with M and E proteins (Fig EV4C and D). Further, co-immunoprecipitation using beads binding Strep-tagged protein showed pull down of HA-tagged cyclin D3 with M and E proteins (Fig EV4E). There was no evidence for binding between cyclin D3 and N or S proteins (Fig EV4). Furthermore, cyclin D3 interaction with M and E was confirmed by expressing cyclin D3 with E or M or both proteins together (E/M) and immunoprecipitated using anti-HA antibody (Fig 6B).

To further confirm the interaction between cyclin D3 and E and M, we assessed cyclin D3 effect on spike trafficking where E and M play a significant role. It has been previously reported that SARS-CoV-2 E and M proteins regulate intracellular trafficking and processing of spike, leading to S retention in the ER and Golgi and preventing syncytia formation (Boson *et al*, 2021). It is possible that this interaction between structural proteins and spike retention allows S to target the virion assembly sites. We hypothesised that if cyclin D3 is interacting with E and M, it might impact their function in spike processing/trafficking, and we can use it as a read-out by assessing the syncytia formation.

Firstly, we confirmed that cyclin D3 degradation is independent of syncytia formation. VERO AT2 were infected with Omicron lineages BA.1 which has limited ability to form syncytia and BA.2 which is more efficient in syncytia formation (Appendix Fig S5A) (Yamasoba *et al*, 2022). Both Omicron lineages depleted cyclin D3 in cells (Appendix Fig S5B) verifying that cyclin D3 degradation is independent of syncytia formation, and also supporting the notion that cyclin D3 degradation is a conserved function among SARS-CoV-2 variants.

In the next step, a split GFP system (Cabantous *et al*, 2005) was used to confirm that E and M or a combination of both (E/M) impact spike-mediated syncytia formation as published previously (Boson *et al*, 2021). 293T GFP11 cells were transfected with full-length S (WT), and/or with E and M. Twenty-four hours post-transfection, cells were seeded at a 1:1 ratio with Vero-GFP10, and cell-to-cell fusion was measured 18 h later to determine a proportion of green area to total phase area (Fig 6C–G). Indeed, both structural proteins when co-expressed with S significantly decreased GFP+ve area (cell–cell fusion) (Fig 6C and E). Interestingly, when 293T GFP11 cells were transfected with full-length S (WT), and/or with E, M and cyclin D3, cyclin D3 had no effect on the reduction of syncytia when expressed together with S, S + E or S + M (Fig 6F). However, it increased syncytia formation in combination with S + M + E, suggestive of compromising E and M impact on spike processing/trafficking towards the cell surface (Fig 6D and G).

### Cyclin D3 impairs envelope incorporation into virions

To further understand the mechanism of cyclin D3 restriction during SARS-CoV-2 assembly, we used a SARS-CoV-2 viral-like particle (VLP) assay. It has been shown that four SARS-CoV-2 structural proteins, spike (S), nucleocapsid (N), envelope (E) and membrane (M) are essential for SARS-CoV-2 VLP formation. Formed VLPs have molecular and morphological properties of native virions (Xu *et al*, 2020; Yurkovetskiy *et al*, 2020; Syed *et al*, 2021). We transfected 293T cells with plasmids encoding S, N, M and E in the presence or absence of cyclin D3 expressed from transfected plasmids. VLPs were collected in media supernatants, purified through 20% sucrose and detected in western blot (Fig 6H–J). Even though all proteins were expressed at similar levels in cells, significantly less envelope protein was incorporated into VLPs in the presence of cyclin D3 (Fig 6I and J). Furthermore, we noted that cyclin D3 was incorporated into VLPs as well (Fig 6I). There was no change in the incorporation of spike, M or N into virions. Suggestive of the specific effect of cyclin D3 on SARS-CoV-2 envelope protein.

Of note, cyclin D3 has no direct effect on spike incorporation into virions or trafficking (in the absence of E and M) as confirmed by the lack of negative effect on syncytia formation (Fig 6D, F, and G), and spike incorporation into SARS-CoV-2 VLPs (Fig 6I and J) or spike pseudotyped HIV-1-based VLPs (Appendix Fig S5C).

Lastly, we investigated whether SARS-CoV-2 structural proteins are capable of inducing cyclin D3 degradation. We transfected 293T cells with a panel of selected SARS-CoV-2 genes. The choice of genes was based on SARS-CoV-2–human protein–protein interactions (Gordon *et al*, 2020), and based on cellular localisation (e.g. nsp9, like cyclin D3, localises to the nucleus and cytoplasm), known interaction with proteins that interact with cyclin D3 (e.g. M interacts with AKAP8L, known interactor of cyclin D3) and function that


© 2022 The Authors

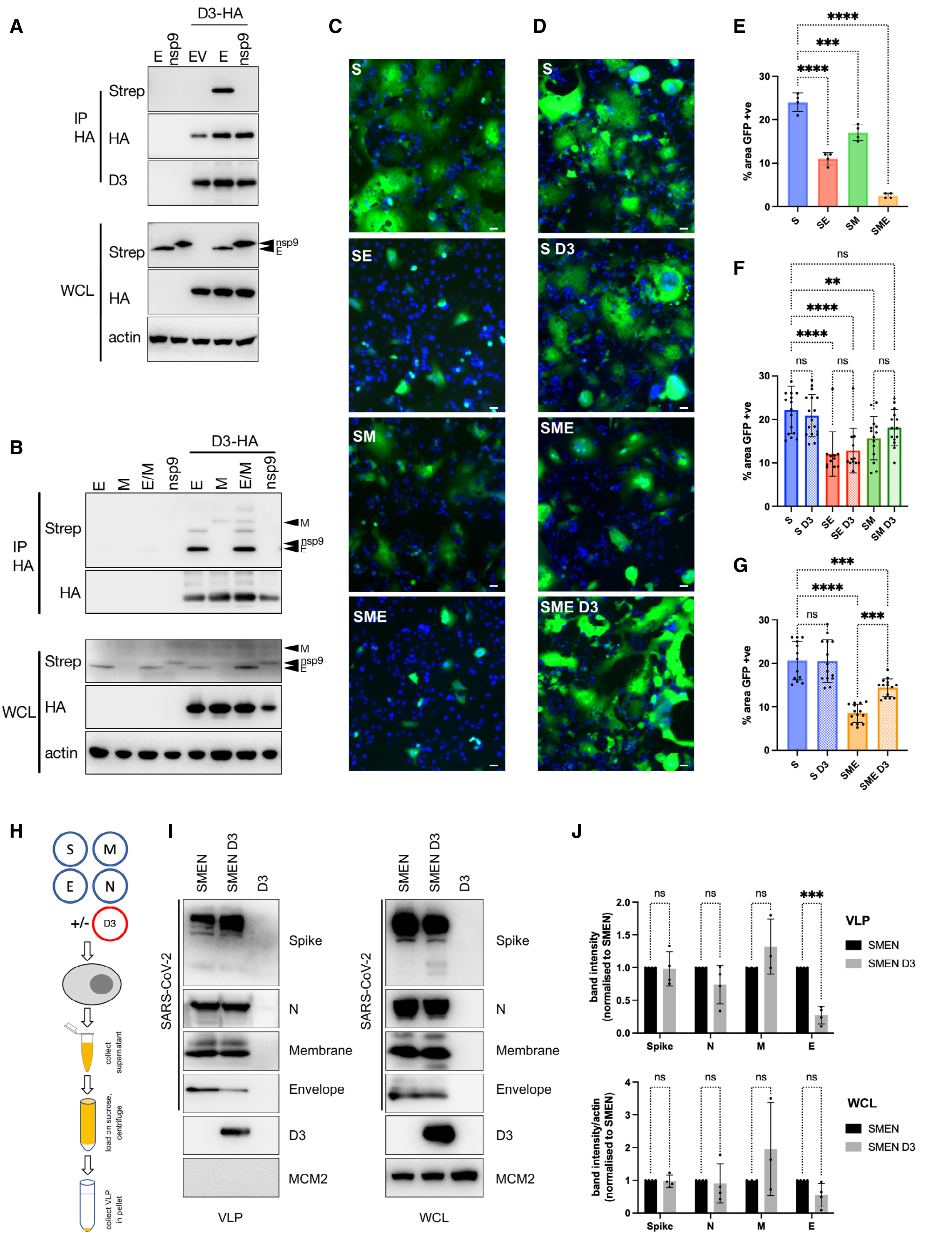
A
D3-HA
E nsp9 EV E nsp9
IP HA
Strep
HA
D3
WCL
Strep
HA
actin
nsp9
E
B
D3-HA
E M E/M nsp9 E M E/M nsp9
C
S
SE
SM
SME
D
S
S D3
SME
SME D3
E
% area GFP +ve
S SE SM SME
F
ns
S S D3 SE SE D3 SM SM D3
G
S S D3 SME SME D3
H
+/-
collect supernatant
load on sucrose, centrifuge
collect VLP in pellet
I
SMEN SMEN D3 D3
SARS-CoV-2
Spike
N
Membrane
Envelope
MCM2
VLP
WCL
J
band intensity (normalised to SMEN)
band intensity/actin (normalised to SMEN)
Spike N M E
SMEN
SMEN D3

Figure 6.

**Figure 6. Cyclin D3 associates with SARS-CoV-2 proteins E and M.**

A 293T cells were contransfected with HA-cyclin D3 and Strep-tag-SARS-CoV-2 E or nsp9, and control plasmid (EV). Immunoprecipitation was performed using anti-HA antibody. The immunoprecipitates were blotted with anti-Strep, anti-HA and cyclin D3 antibodies.

B 293T cells were contransfected with HA-cyclin D3 and Strep-tag-SARS-CoV-2 E, M, both E and M or nsp9. Immunoprecipitation was performed using anti-HA antibody. The immunoprecipitates were blotted with anti-Strep, anti-HA antibodies. WCL, whole-cell lysate.

C–G 293T GFP11 cells were transfected with spike, and/or with envelope, membrane and cyclin D3. 24 h post-transfection, cells were seeded at a 1:1 ratio with Vero-GFP10 cells, and the percentage of GFP + ve area (syncytia) was determined 18 h later. S, spike; E, envelope; M, membrane; D3, cyclin D3. (C, D) Representative images of GFP+ syncytia. Scale bars: 40 μm. (E–G) Quantification of cell-to-cell fusion showing the percentage of the GFP + ve area to the acquired total cell area. (E) $n = 4$ biological replicates. (F, G) $n = 4$ biological replicates; technical triplicates shown; one-way ANOVA with Dunnett's multiple comparisons test: ns, non-significant; ****$P < 0.0001$; ***$P < 0.001$; **$P < 0.01$. Bars indicate mean with SD.

H Diagram of SARS-CoV-2 VLPs construction in the presence or absence of cyclin D3 in 293T cells.

I Representative example of western blot from purified SARS-CoV-2 virus-like particles (VLP) and whole-cell lysates (WCL). MCM2 was used as a loading control.

J Quantification of protein incorporation (into VLPs) or expression (in WCL). $n = 4$ biological replicates; one-way ANOVA with Dunnett's multiple comparisons test: ns, non-significant; ***$P < 0.001$. Bars indicate the mean with SD. N, nucleocapsid; M, membrane; E, envelope; S, spike.

Source data are available online for this figure.

might link to proteasomal degradation in general (e.g. Orf8, binds to FBXL12, substrate-recognition component of the SCF). Of 15 studied proteins, orf3b and orf10 were not expressed. Structural proteins S, N, M and E did not mediate cyclin D3 degradation (Fig EV5). Two SARS-CoV-2 proteins capable of affecting cyclin D3 expression/degradation were identified, namely nsp1 and orf8 (Fig EV5B).

These data together demonstrate that cyclin D3 associates with M and E proteins important for SARS-CoV-2 assembly, impairing their optimal function in spike trafficking and disrupting the efficient incorporation of SARS-CoV-2 envelope into virions.

## Discussion

Here, we show that SARS-CoV-2 infection depletes levels of cyclin D and suggest that this depletion is independent of changes to cell cycle arrest in infected cells. Furthermore, cyclin D3 seems to interfere with envelope and membrane SARS-CoV-2 protein function in spike trafficking and E incorporation into virions.

The molecular mechanism of coronavirus-mediated regulation of cell cycle and cell-cycle-associated proteins has not been comprehensively investigated, especially in SARS-CoV-2 infection.

Many viruses can manipulate the cell cycle of infected cells. Coronaviruses are not an exception (Chen *et al*, 2004; Chen & Makino, 2004; Surjit *et al*, 2006; Yuan *et al*, 2006, 2007; Harrison *et al*, 2007; Sun *et al*, 2018). SARS-CoV-2 has been reported to arrest the cell cycle in the S/G2/M phase in VERO E6 cells (Bouhaddou *et al*, 2020). Our own data confirmed this observation using the Fucci system (Koh *et al*, 2017), and comparing two different cell lines VERO AT2 (monkey, *Cercopithecus aethiops*, epithelial/kidney) and A549 AT2 (human, epithelial/lung) both expressing ACE2/TMPRSS2, we uncovered that cell cycle arrest in SARS-CoV-2-infected cells occurs at different stages of cell cycle phases. While cell cycle arrest in VERO AT2 is at the late S and G2/M phases as previously reported (Bouhaddou *et al*, 2020), A549 AT2 cells are arrested specifically at the early S phase. This difference would not be possible to uncover if classical cell cycle techniques, like propidium iodine or DAPI staining, would be used as they cannot separate G0 and G1 phases or the early S phase. Based on these data, we can conclude that SARS-CoV-2 infection of human cells arrests the cell cycle in S phase, possibly creating a favourable environment for viral replication and spread. However, the exact mechanism is unclear at the present and warrants further investigation.

Cyclin-dependent kinases (CDKs) and corresponding cyclins are an essential part of cell cycle progression. Several coronaviruses have been shown to regulate these proteins (Chen *et al*, 2004; Chen & Makino, 2004; Surjit *et al*, 2006; Yuan *et al*, 2006, 2007; Harrison *et al*, 2007; Sun *et al*, 2018). Specifically, down-regulation of cyclin D1 has been shown in IBV (Harrison *et al*, 2007) and SARS-CoV-1 (Surjit *et al*, 2006) and cyclin D3 reduction in SARS-CoV-1 infection (Yuan *et al*, 2006). However, a decrease in cyclin Ds has been always connected to cell cycle arrest. Here, we show that SARS-CoV-2 mediates the translocation of cyclin D1 and D3 from the nucleus into cytoplasm for proteasome degradation in both VERO AT2 and A549 AT2, and Calu3 cells. Analysis of other cyclins in VERO AT2 cell did not reveal any changes to cyclin A (promoting S phase entry and mitosis), B (accumulates in G2 phase) or E (limiting factor for G1-phase progression and S-phase entry), supporting the notion that degradation of cyclin D proteins is specific.

Further, we present data supporting the notion that the down-regulation of cyclin D during SARS-CoV-2 infection is independent of cell cycle arrest. Firstly, a detailed analysis of cell cycle arrest in cells depleted for cyclin D1 and D3 revealed arrest in G1 phase as published previously (Masamha & Benbrook, 2009) not in S or G2/M phase. Nevertheless, when cells in the absence of cyclin Ds were infected, cells were still arrested in the early S phase (in A549 AT2) or S/G2/M (in VERO AT2), suggestive of cell cycle arrest during infection being caused by a factor other than depletion of cyclin D proteins. Secondly, expression of proteasome-resistant cyclin D3 mutant T283A had no effect on cell cycle arrest caused by SARS-CoV-2 infection. Lastly, using single-cell microscopy and Fucci cell cycle sensor allowed us to measure cyclin Ds relocalisation from the nucleus into cytoplasm for degradation in specific cell cycle phases. All cell cycle phases detected showed that in the presence of infection, cyclin Ds are always relocalised from the nucleus to cytoplasm for degradation. It has been published recently that CDK1/2 activities are reduced during SARS-CoV-2 infection and might be leading to S/G2 phase arrest (Bouhaddou *et al*, 2020). Although no changes at the expression level were evident for CDKs in our study, the phosphorylation state of these kinases was not investigated, and it is thus possible that such changes may occur and affect cell cycle progression.

 © 2022 The Authors

If cyclin D is not used as a means for the virus to arrest cell cycle, it is possible that it might represent a host restriction factor preventing optimal viral replication and spread. Importantly, depletion of cyclin D3 by siRNA increased viral titres after SARS-CoV-2 infection in viral supernatants. This suggests that cyclin D3 might play role in the viral spread. This effect is reminiscent of the role of cyclin D3 in influenza infection, where cyclin D3 depletion resulted in increased viral production. This study also showed that cyclin D3 binds M2 protein and interferes with the M1–M2 interaction leading to defective viral assembly (Fan *et al*, 2017).

Interestingly, M2 protein was identified as the first viroporin (Duff & Ashley, 1992; Pinto *et al*, 1992). In coronaviruses, several viroporins have been discovered, including SARS-CoV-1 E (Wilson *et al*, 2004; Verdia-Baguena *et al*, 2012). As E proteins are highly conserved in the SARS family, we investigated the possibility that SARS-CoV-2 envelope and cyclin D3 are potential binding partners. Our work indeed revealed cyclin D3 as a new interactor with SARS-CoV-2 E. Recently, a comparative viral–human protein–protein interaction analysis for SARS-CoV-2 has been published (Gordon *et al*, 2020). In their study, they did not uncover cyclin D3 as an interactor with any viral protein, however, the study was conducted in HEK293T cells, the cell line that we showed does not express cyclin D3. It has been shown that SARS-CoV-1 and SARS-CoV-2 proteins M, E and N are required for virion assembly that takes place in ER-Golgi-intermediate compartment cisternae (Krijnse-Locker *et al*, 1994; Cortese *et al*, 2020; V'Kovski *et al*, 2021). M and E proteins seem to present an assembly core interacting with both spike and nucleocapsid proteins (Godeke *et al*, 2000; Escors *et al*, 2001). Importantly, our data show that cyclin D3 associates with M as well, supporting our hypothesis that cyclin D3 could impair SARS-CoV-2 assembly and/or spread. Further, E and M proteins have been implicated in spike processing and trafficking (Boson *et al*, 2021). We have shown that while S incorporation into particles is not affected directly by cyclin D3, trafficking of S might be altered through binding to E and/or M proteins. It has been shown that spike is retained inside cells when expressed together with E and M probably to target S to proximity of intracellular virus assembly sites. Our data show that S is retained in the cells in the presence of M and E but its trafficking towards the membrane and ability to form syncytia is partially rescued when cyclin D3 is present. This supports the concept of cyclin D3 interacting with E and M and changing their optimal function in spike trafficking. Further, details from SARS-CoV-2 assembly assay that produces VLPs with molecular and morphological properties of native virions (Xu *et al*, 2020; Yurkovetskiy *et al*, 2020; Syed *et al*, 2021) revealed that in the presence of cyclin D3, SARS-CoV-2 E is inefficiently incorporated into virions. This is an important finding as it has been shown before that SARS-CoV-1 (SARS-CoV-1 E 95% identical to SARS-CoV-2 E) virus missing E protein replicates to 100- to 1,000-fold lower titres than the wild-type, and lower viral load was accompanied by less inflammation in the lungs in a hamster model. It has been suggested that the total number or virion morphology is similar, but the number of mature virions was higher in the SARS-CoV-1-infected cells than in SARS-CoV-1-ΔE-infected cells (DeDiego *et al*, 2007, 2011). Furthermore, E proteins are known viroporins, able to form ion-conductive pores in host membranes and disrupt the physiological function of cells. Viroporins are known to increase the release of infectious virus from cells but also facilitate the entry of the virus into cells. It has been shown that SARS-CoV-1 viruses, in which pore activity is inhibited, are less infectious and pathogenic (Nieto-Torres *et al*, 2015). It is possible that less incorporation of E in the presence of cyclin D3 into virions will have a similar effect on the virus as E depletion and cause changes in virus maturation and pathogenicity of SARS-CoV-2.

Surprisingly, none of the SARS-CoV-2 structural proteins could degrade cyclin D3. Changes in cyclin D3 expression levels were detected in the presence of nsp1 and Orf8. nsp1 binds the ribosomal mRNA channel to inhibit the translation of both viral and native 5′UTR-containing reporter mRNA (Wang *et al*, 2000; Schubert *et al*, 2020). It is possible that the actual translation of cyclin D is inhibited, but would not explain why other cyclins, e.g. A2, E1 or B1 (all cyclins in this study have similar short half-lives of 30–180 min; Penelova *et al*, 2005), are unaffected. Orf8 disrupts interferon signalling (Li *et al*, 2020), and down-regulates MHC-I in cells (Zhang *et al*, 2021), as well as regulating protein folding and transport machinery in ER (Liu *et al*, 2022). The potential role of orf8 in cyclin D3 degradation is currently not understood and is under investigation.

Our work provides important insight into the mechanism through which cyclin D3 limits SARS-CoV-2 infection. In the light of immune evasion from vaccination, it is important that this phenomenon was observed across different SARS-CoV-2 variants suggesting that this mechanism provides a universal target for the development of antivirals. Our data suggest that cyclin D3 associates with SARS-CoV-2 E and M proteins, decreasing E incorporation into virions and thereby interfering with efficient viral spread. SARS-CoV-2 has therefore evolved strategies to degrade cyclin D3 that require further investigation, with the hope that it can be translated to therapeutics.

## Materials and Methods

### Reagents

#### *Cell lines*

All cells were maintained in Dulbecco's modified Eagle medium (DMEM) supplemented with 10% foetal calf serum (FCS), 100 U $ml^{-1}$ penicillin and 100 mg $ml^{-1}$ streptomycin, and regularly tested and found to be mycoplasma free. Following cells were gifts: A549 ACE2/TMPRSS2 (Rihn *et al*, 2021) Massimo Palmerini; Vero ACE2/TMPRSS2 from Emma Thomson;HeLa-ACE2 from James Voss; 293T (a human embryonic kidney cell line, ATCC CRL-3216); 293T GFP11 cells and Vero-GFP10 cells for split GFP assay were a gift from Leo James (Papa *et al*, 2021); and Calu3 cells a gift from Paul Lehner, which were maintained in Eagle's minimum essential medium containing 10% foetal calf serum, 1% non-essential amino acid solution and 1% L-glutamine solution.

#### *Viruses*

WT (lineage B, SARS-CoV-2/human/Liverpool/REMRQ0001/2020), a kind gift from Ian Goodfellow, previously isolated by Lance Turtle (University of Liverpool), David Matthews and Andrew Davidson (University of Bristol). Alpha variant (B.1.1.7; SARS-CoV-2 England/ATACCC 174/2020) was a gift from G. Towers (preprint: Reuschl *et al*, 2021), lineages B.1.1.617.2 (Delta, GISAID: EPI_ISL_1731019) and B 0.1.1.529 (Omicron UK isolate, G.

© 2022 The Authors 

Screaton) (Dejnirattisai *et al*, 2022; Nutalai *et al*, 2022) were received as part of the work conducted by G2P-UK National Virology Consortium. Viral stocks were prepared by passaging once in VERO AT2 cells. Cells were infected at low MOI and incubated for 72 h. Virus-containing culture supernatants were clarified by centrifugation (500 *g*, 5 min) and aliquots frozen at −80°C. Standard TCID50 assay in VERO AT2 was used to determine MOI of viral stocks.

### Plasmids

pBOB-EF1-FastFUCCI-Puro was a gift from Kevin Brindle & Duncan Jodrell (Addgene plasmid # 86849; http://n2t.net/addgene:86849; RRID: Addgene_86849) (Koh *et al*, 2017). pCMV5 cyclin D3 HA was obtained from MRC-PPU Reagents and Services. Rc/CMV cyclin D1 HA was a gift from Philip Hinds (Addgene plasmid # 8948; http://n2t.net/addgene:8948; RRID: Addgene_8948) (Baker *et al*, 2005). pLVX-EF1alpha-SARS-CoV-2-E-2xStrep-IRES-Puro (Addgene plasmid # 141385; http://n2t.net/addgene:141385; RRID:Addgene_141385); pLVX-EF1alpha-SARS-CoV-2-M-2xStrep-IRES-Puro (Addgene plasmid # 141386; http://n2t.net/addgene:141386; RRID: Addgene_141386); pLVX-EF1alpha-SARS-CoV-2-nsp9-2xStrep-IRES-Puro (Addgene plasmid # 141375; http://n2t.net/addgene:141375; RRID:Addgene_141375); pLVX-EF1alpha-SARS-CoV-2-N-2xStrep-IRES-Puro (Addgene plasmid # 141391; http://n2t.net/addgene:141391; RRID:Addgene_141391); pLVX-EF1alpha-SARS-CoV-2-orf3a-2xStrep-IRES-Puro (Addgene plasmid # 141383; http://n2t.net/addgene:141383; RRID:Addgene_141383); pLXV-EF1alpha-2xStrep-SARS-CoV-2-orf3b-IRES-Puro (Addgene plasmid # 141384; http://n2t.net/addgene:141384; RRID:Addgene_141384); pLVX-EF1alpha-SARS-CoV-2-orf7a-2xStrep-IRES-Puro (Addgene plasmid # 141388; http://n2t.net/addgene:141388; RRID:Addgene_141388); pLVX-EF1alpha-SARS-CoV-2-orf8-2xStrep-IRES-Puro (Addgene plasmid # 141390; http://n2t.net/addgene:141390; RRID: Addgene_141390); pLVX-EF1alpha-SARS-CoV-2-orf9b-2xStrep-IRES-Puro (Addgene plasmid # 141392; http://n2t.net/addgene:141392; RRID:Addgene_141392); pLXV-EF1alpha-2xStrep-SARS-CoV-2-orf9c-IRES-Puro (Addgene plasmid # 141393; http://n2t.net/addgene:141393; RRID:Addgene_141393); pLVX-EF1alpha-SARS-CoV-2-orf10-2xStrep-IRES-Puro (Addgene plasmid # 141394; http://n2t.net/addgene:141394; RRID:Addgene_141394); pLVX-EF1alpha-SARS-CoV-2-nsp1-2xStrep-IRES-Puro (Addgene plasmid # 141367; http://n2t.net/addgene:141367; RRID:Addgene_141367); pLVX-EF1alpha-SARS-CoV-2-nsp12-2xStrep-IRES-Puro (Addgene plasmid # 141378; http://n2t.net/addgene:141378; RRID:Addgene_141378); pLVX-EF1alpha-SARS-CoV-2-nsp13-2xStrep-IRES-Puro (Addgene plasmid # 141379; http://n2t.net/addgene:141379; RRID: Addgene_141379); and pLVX-EF1alpha-eGFP-2xStrep-IRES-Puro (Addgene plasmid # 141395; http://n2t.net/addgene:141395; RRID: Addgene_141395) were a gift from Nevan Krogan (Gordon *et al*, 2020). pEXN-MNCX, MLV vector-encoding N-terminal double HA tag (Zhang *et al*, 2006). pCAGGS_SARS-CoV-2_Spike was obtained from NIBS. Plasmids used for SARS-CoV-2 assembly assay: pCov2-CoOpNucleocapsid-I-GFP, pCov2-CoOpSpike-I-GFP, pCov2-CoOpEnvelope-I-GFP and pCov2-CoOpMembrane-I-GFP were a gift from Nicholas Matheson, prepared by S. Marelli.

### Antibodies

Following antibodies were used. Anti-rabbit IgG, HRP-linked antibody (7074) and cyclin D3 mouse mAb (DCS22, 2936) were from Cell Signaling. Mouse IgG HRP-linked whole Ab (NXA931V) from Sigma. Goat anti-mouse IgG (H+L) cross-adsorbed secondary antibody: Alexa 488 (A-11001), Alexa 594 (A-11032) and Alexa 647 (A-21236); goat anti-rabbit IgG (H+L) cross-adsorbed secondary antibody: Alexa 488 (A-11034) and Alexa 405 (A-48254); rabbit polyclonal SARS-CoV-2 spike (PA1-41165) and rabbit monoclonal SARS-CoV-2 nucleocapsid (MA5-29982) from Thermo Fisher Scientific. Mouse monoclonal cyclin D3 (D-7, sc-6283) from Santa Cruz. Rabbit polyclonal cyclin A2 antibody (GTX103042); rabbit polyclonal cyclin D1 antibody (N1C3, GTX108824); rabbit polyclonal cyclin E1 antibody (GTX103045); rabbit polyclonal cyclin B1 antibody (GTX100911) and monoclonal SARS-CoV-2 spike (GTX632604) from GeneTex. Mouse monoclonal Strep II Tag antibody (NBP2-43735) from Novus Biologicals. Mouse monoclonal actin (ab6276) from Abcam. Strep-Tactin-HRP; MagStrep "type3" XT beads (2-4090-002) from IBA Lifesciences. Anti-HA magnetic beads (88836) from Thermo Fisher Scientific. Rabbit polyclonal SARS-CoV-2 membrane glycoprotein polyclonal antibody (SARS-COV2-M-101AP), rabbit polyclonal SARS-CoV-2 envelope protein polyclonal antibody (SARS-COV2-E-101AP) and rabbit polyclonal MCM2 (PA5-79645) from Thermo Fisher Scientific. Mouse monoclonal HIV-1 p24/p55 (ARP365 and ARP366) from NIBSC.

## Methods

### SDS–PAGE and immunoblots

Cells were lysed in reducing Laemmli SDS sample buffer containing PhosSTOP (Phosphatase Inhibitor Cocktail Tablets, Roche, Switzerland) at 96°C for 10 min and the proteins separated on NuPAGE® Novex® 4–12% Bis–Tris Gels. Subsequently, the proteins were transferred onto PVDF membranes (Millipore, Billerica, MA, USA), the membranes were quenched and proteins were detected using specific antibodies. Labelled protein bands were detected using Amersham ECL Prime Western Blotting Detection Reagent (GE Healthcare, USA) and ChemiDoc MP Imaging System (Bio-Rad) CCD camera. Protein band intensities were quantified using ChemiDoc MP Imaging System and Image Lab software (Bio-Rad, Hercules, CA, USA).

### Immunofluorescence

Cells were fixed in 4% PFA, quenched with 50 mM $NH_4Cl$ and permeabilised with 0.1% Triton X-100 in PBS. After blocking in PBS/1% FCS, cells were labelled for 1 h with primary antibodies diluted in PBS/1% FCS, washed and labelled again with Alexa Fluor secondary antibodies for 1 h. Cells were washed in PBS/1% FCS and stained with DAPI in PBS for 5 min. Labelled cells were detected using ArrayScan high-content system (Thermo Fisher, Waltham, MA, USA) and analysed using Harmony (PerkinElmer, Waltham, MA, USA) and ImageJ software. Infected cells have been identified by SARS-CoV-2 nucleocapsid or spike staining.

To measure the location of cyclin D staining in cells, DAPI staining was used to demarcate the nuclear and cytoplasmic regions of interest (ROI). Harmony (PerkinElmer, Waltham, MA, USA) and ImageJ software were used to measure MFI for each protein in each region. Values are presented as a ratio of signal (nucleus/cytoplasm). Usually, 50–200 cells have been quantified.

 © 2022 The Authors

#### Cell cycle analysis using fluorescence ubiquitination cell cycle indicator (Fucci)

Fucci cassette was cloned from pBOB-EF1-FastFucci-Puro vector to pEXN-MNCX using BamHI/NotI restriction sites. Fucci-containing lentiviral particles were produced as follows: 293Tv cells were transfected with pEXN-MNCX-Fucci, CMVi and pMD2.G. Cell supernatants containing viruses (Fucci VLP) were collected 48 h post-transfection and frozen at −80°C. Cells were transduced using Fucci VLP for 18 h. Cells were infected with SARS-CoV-2 variants and fixed in 4% PFA 24 h post-infection. SARS-CoV-2-positive cells were identified by nucleocapsid staining and flow cytometry. Cell populations positive or negative for SARS-CoV-2 nucleocapsid staining were gated and Cdt1-RFP-positive (G1 phase), geminin-GFP-positive (S/G2/M phase) and Cdt1-RFP/geminin-GFP-positive (early S phase) populations were identified using flow cytometry using LSRFortessa X-20 (BD Biosciences, UK) and FlowJo software (Tree Star, OR, USA).

For immunofluorescence and high-throughput microscopy, cells were transduced using Fucci VLP for 18 h. Cells were infected with SARS-CoV-2 variants and fixed in 4% PFA 24 h post-infection. SARS-CoV-2-positive cells were identified by nucleocapsid or spike staining. Cells in G1 phase were identified by Cdt1-RFP; early S, Cdt1-RFP/geminin-GFP and S/G2/M, geminin-GFP signal.

#### Knockdown

Cells were transfected with 20 pmol of siRNA Ambion Silencer Negative Control #1, predesigned invitrogen silencer siRNAs for cyclin D3 (siRNA ID s2523, Chr.6: 41934933–42048894), cyclin D1 (siRNA ID s229, Chr.11: 69641105–69654474) and cyclin A2 (siRNA ID s2514, Chr.4: 121816444–121823933) using lipofectamine RNAiMAX transfection reagent (Invitrogen). The medium was replaced 18 h post-transfection and cells infected with Delta (MOI 0.001), Alpha and WT (MOI 0.1) variants for 4 h. Cells were washed twice in PBS and incubated in a new medium for 48 h. Cell supernatant was collected and used to determine virus titres by standard TCID50, and cells were lysed and used for western blotting to detect viral and cyclins protein expression. Knockdown in Calu3 cell line: cells were transfected with 40 pmol of siRNA Ambion Silencer Negative Control #1, predesigned Invitrogen Silencer siRNAs for cyclin D3 using lipofectamine RNAiMAX transfection reagent. Twenty-four hours later cells were transfected again with 40 pmol of siRNA-negative control or cyclin D3. The medium was replaced 48 h post-transfection and cells infected with Delta (MOI 0.01) variant for 4 h. Cells were washed twice in PBS and incubated in a new medium for 48 h. Cell supernatant was collected and used to determine virus titres by standard TCID50.

#### Co-immunoprecipitation

Cells were transfected with SARS-CoV-2 genes encoding full-length spike (no tag), Strep-tagged nucleocapsid, envelope, membrane and nsp9 proteins in the absence/presence of HA-cyclin D3 for 24 h. Cells were lysed in Pierce IP lysis buffer (Thermo Fisher, Waltham, MA, USA) supplemented with protease and phosphatase inhibitor cocktail (Pierce, Rockford, IL, USA) and 1% digitonin. Cell lysates were precleared by centrifugation. A sample of whole-cell lysate was stored at this point. Precleared cell lysates were incubated with a-HA magnetic beads, MagStrep beads (IBA-Lifescience, Gottingen, Germany) or anti-cyclin D3 monoclonal antibody (sc-xx) bound Protein G Dynabeads for 1 h at 4°C. Beads were washed 3x in IP lysis buffer and 1× in PBS. BTX elution buffer (IBA-Lifescience) was used to elute proteins from MagStrep beads. Laemmli reducing buffer was added to a-HA beads, Dynabeads, and MagStrep beads and 10 min at 90°C was used to eluate/denature attached proteins. Samples were stored till further use.

#### Cell-to-cell fusion assay

293T GFP11 cells were transfected with WT full-length spike, and/or with WT envelope, membrane, cyclin D3 and empty vector (pCDNA, to ensure an equal amount of transfected DNA). Twenty-four hours post-transfection, cells were seeded at a 1:1 ratio with Vero-GFP10 cells, final cell number 6 × 10e4 cells/well. Cell-to-cell fusion was measured 18 h later and determined as a proportion of green area to total phase area using ArrayScan high-content system (Thermo-Fisher, Waltham, MA, USA) and analysed using ImageJ software.

#### Spike pseudotyped lentivirus

Viral vectors were prepared by transfection of 293T cells by using Fugene HD transfection reagent (Promega) as follows. Confluent 293T cells were transfected with a mixture of 13.5ul of Fugene HD, 1 μg of pCAGGS_SARS-CoV-2_Spike, 1ug of p8.91 HIV-1 gag-pol expression vector and 1.5 μg of pCSFLW (expressing the firefly luciferase reporter gene with the HIV-1 packaging signal)(Mlcochova *et al*, 2020), and 1 μg pCMV5 cyclin D3 HA or 1 μg of pcDNA3.1 (as a control). Viral supernatant was collected at 48 h after transfection and filtered through 0.45 um filter. The 50% tissue culture infectious dose ($TCID_{50}$) of SARS-CoV-2 pseudovirus was determined using Steady-Glo luciferase assay system (Promega). Viral supernatants were centrifuged onto 25% sucrose to purify virus particles (23,000 *g*, 2 h, 4°C) and the pellet was resuspended in Laemmli reducing buffer and resolved in western blot.

#### SARS-CoV-2 virus-like particles assembly assay

293T cells were transfected with 3ug of each plasmid-encoding spike, nucleocapsid, membrane, envelope and cyclin D3 or empty plasmid (pcDNA3.1) using Fugene HD (Promega). Media and cells were collected 48 h later. Media containing VLPs were filtered through 0.45 μm filter and purified through 20% sucrose, at 100,000 *g*, 2 h, 4°C. Pellets were washed in PBS, and centrifuged at 100,000 *g*, 2 h, 4°C. Pellets were dried overnight at 4°C and resuspended in 2% SDS in PBS; after the addition of Laemmli reducing buffer, samples were vortexed and heated at 50°C for 20 min. Western blots were performed with fresh samples immediately.

#### Lentiviral delivery of cyclin D3

Cyclin D3 WT and T283A mutant were cloned into pEXN-MNCX using BamHI/NotI restriction sites. Cyclin D3-containing lentiviral particles were produced as follows: 293T cells were transfected with pEXN-MNCX-cyclin D3 (TW or T283A) CMVi and pMD2.G using Fugen HD (Promega). Cell supernatants containing viruses were collected 48 h post-transfection and frozen at −80°C.

#### Quantitative PCR

Total RNA was isolated from the infected cells using Total RNA Purification Kit from Norgen Biotek (Thorold, Canada). cDNA was synthesised using Superscript III Reverse Transcriptase (Thermo Fisher Scientific) using 500 ng of template RNA. qPCR was performed

© 2022 The Authors

on QuantStudio7 (Thermo Fisher Scientific) using Fast SYRB green master mix (Thermo Fisher Scientific). Expression levels of target genes were normalised to glyceraldehyde-3-phosphate dehydrogenase (GAPDH). Forward primer: 5′GCCTCTTCTCGTTCCTCAT CAC3′; Reverse primer: 5′AGCAGCATCACCGCCATTG3′.

## Data availability

This study includes no data deposited in external repositories.

**Expanded View** for this article is available online.

### Acknowledgements
We thank Voss for HeLa ACE2; S. Rihn for the A549-ACE2/TMPRSS2 cell; N. Matheson and S. Marelli for full-length spike, membrane and envelope plasmids. This research was supported by the Cambridge NIHR BRC Cell Phenotyping Hub. In particular, we wish to thank V. Romashova for their advice and support in flow imaging. R.K.G. is supported by a Wellcome Trust Senior Fellowship in Clinical Science (WT108082AIA). We acknowledge additional support from Lister and Rosetrees Institutes.

### Author contributions
**Petra Mlcochova:** Conceptualization; data curation; formal analysis; validation; investigation; visualization; methodology; writing – original draft; writing – review and editing. **Ravindra K Gupta:** Data curation; funding acquisition; writing – review and editing.

### Disclosure and competing interests statement
The authors declare that they have no conflict of interest.

## References

Agami R, Bernards R (2000) Distinct initiation and maintenance mechanisms cooperate to induce G1 cell cycle arrest in response to DNA damage. *Cell* 102: 55–66

Baker GL, Landis MW, Hinds PW (2005) Multiple functions of D-type cyclins can antagonize pRb-mediated suppression of proliferation. *Cell Cycle* 4: 330–338

Boson B, Legros V, Zhou B, Siret E, Mathieu C, Cosset FL, Lavillette D, Denolly S (2021) The SARS-CoV-2 envelope and membrane proteins modulate maturation and retention of the spike protein, allowing assembly of virus-like particles. *J Biol Chem* 296: 100111

Bouhaddou M, Memon D, Meyer B, White KM, Rezelj VV, Marrero MC, Polacco BJ, Melnyk JE, Ulferts S, Kaake RM *et al* (2020) The global phosphorylation landscape of SARS-CoV-2 infection. *Cell* 182: 685-+

Cabantous S, Terwilliger TC, Waldo GS (2005) Protein tagging and detection with engineered self-assembling fragments of green fluorescent protein. *Nat Biotechnol* 23: 102–107

Casanovas O, Jaumot M, Paules AB, Agell N, Bachs O (2004) P38SAPK2 phosphorylates cyclin D3 at Thr-283 and targets it for proteasomal degradation. *Oncogene* 23: 7537–7544

Cato MH, Chintalapati SK, Yau IW, Omori SA, Rickert RC (2011) Cyclin D3 is selectively required for proliferative expansion of germinal center B cells. *Mol Cell Biol* 31: 127–137

Chen CJ, Makino S (2004) Murine coronavirus replication induces cell cycle arrest in G0/G1 phase. *J Virol* 78: 5658–5669

Chen CJ, Sugiyama K, Kubo H, Huang C, Makino S (2004) Murine coronavirus nonstructural protein p28 arrests cell cycle in G0/G1 phase. *J Virol* 78: 10410–10419

Cortese M, Lee JY, Cerikan B, Neufeldt CJ, Oorschot VMJ, Kohrer S, Hennies J, Schieber NL, Ronchi P, Mizzon G *et al* (2020) Integrative imaging reveals SARS-CoV-2-induced reshaping of subcellular morphologies. *Cell Host Microbe* 28: 853–866.e5

DeDiego ML, Alvarez E, Almazan F, Rejas MT, Lamirande E, Roberts A, Shieh WJ, Zaki SR, Subbarao K, Enjuanes L (2007) A severe acute respiratory syndrome coronavirus that lacks the E gene is attenuated *in vitro* and *in vivo*. *J Virol* 81: 1701–1713

DeDiego ML, Nieto-Torres JL, Jimenez-Guardeno JM, Regla-Nava JA, Alvarez E, Oliveros JC, Zhao J, Fett C, Perlman S, Enjuanes L (2011) Severe acute respiratory syndrome coronavirus envelope protein regulates cell stress response and apoptosis. *PLoS Pathog* 7: e1002315

Dejnirattisai W, Huo J, Zhou D, Zahradnik J, Supasa P, Liu C, Duyvesteyn HME, Ginn HM, Mentzer AJ, Tuekprakhon A *et al* (2022) SARS-CoV-2 omicron-B.1.1.529 leads to widespread escape from neutralizing antibody responses. *Cell* 185: 467–484.e15

Diehl JA, Zindy F, Sherr CJ (1997) Inhibition of cyclin D1 phosphorylation on threonine-286 prevents its rapid degradation via the ubiquitin-proteasome pathway. *Genes Dev* 11: 957–972

Duff KC, Ashley RH (1992) The transmembrane domain of influenza A M2 protein forms amantadine-sensitive proton channels in planar lipid bilayers. *Virology* 190: 485–489

Escors D, Ortego J, Laude H, Enjuanes L (2001) The membrane M protein carboxy terminus binds to transmissible gastroenteritis coronavirus core and contributes to core stability. *J Virol* 75: 1312–1324

Fan Y, Mok CK, Chan MC, Zhang Y, Nal B, Kien F, Bruzzone R, Sanyal S (2017) Cell cycle-independent role of cyclin D3 in host restriction of influenza virus infection. *J Biol Chem* 292: 5070–5088

Godeke GJ, de Haan CA, Rossen JW, Vennema H, Rottier PJ (2000) Assembly of spikes into coronavirus particles is mediated by the carboxy-terminal domain of the spike protein. *J Virol* 74: 1566–1571

Gordon DE, Hiatt J, Bouhaddou M, Rezelj VV, Ulferts S, Braberg H, Jureka AS, Obernier K, Guo JZ, Batra J *et al* (2020) Comparative host-coronavirus protein interaction networks reveal pan-viral disease mechanisms. *Science* 370: eabe9403

Harrison SM, Dove BK, Rothwell L, Kaiser P, Tarpey I, Brooks G, Hiscox JA (2007) Characterisation of cyclin D1 down-regulation in coronavirus infected cells. *FEBS Lett* 581: 1275–1286

Koh SB, Mascalchi P, Rodriguez E, Lin Y, Jodrell DI, Richards FM, Lyons SK (2017) A quantitative FastFUCCI assay defines cell cycle dynamics at a single-cell level. *J Cell Sci* 130: 512–520

Krijnse-Locker J, Ericsson M, Rottier PJ, Griffiths G (1994) Characterization of the budding compartment of mouse hepatitis virus: evidence that transport from the RER to the Golgi complex requires only one vesicular transport step. *J Cell Biol* 124: 55–70

Kumar B, Hawkins GM, Kicmal T, Qing E, Timm E, Gallagher T (2021) Assembly and entry of severe acute respiratory syndrome coronavirus 2 (SARS-CoV2): evaluation using virus-like particles. *Cell* 10: 853

Li FQ, Tam JP, Liu DX (2007) Cell cycle arrest and apoptosis induced by the coronavirus infectious bronchitis virus in the absence of p53. *Virology* 365: 435–445

Li JY, Liao CH, Wang Q, Tan YJ, Luo R, Qiu Y, Ge XY (2020) The ORF6, ORF8 and nucleocapsid proteins of SARS-CoV-2 inhibit type I interferon signaling pathway. *Virus Res* 286: 198074

Liu P, Wang X, Sun Y, Zhao H, Cheng F, Wang J, Yang F, Hu J, Zhang H, Wang CC *et al* (2022) SARS-CoV-2 ORF8 reshapes the ER through forming mixed disulfides with ER oxidoreductases. *Redox Biol* 54: 102388

 © 2022 The Authors

Masamha CP, Benbrook DM (2009) Cyclin D1 degradation is sufficient to induce G1 cell cycle arrest despite constitutive expression of cyclin E2 in ovarian cancer cells. *Cancer Res* 69: 6565 – 6572

Mlcochova P, Collier D, Ritchie A, Assennato SM, Hosmillo M, Goel N, Meng B, Chatterjee K, Mendoza V, Temperton N *et al* (2020) Combined point-of-care nucleic acid and antibody testing for SARS-CoV-2 following emergence of D614G spike variant. *Cell Rep Med* 1: 100099

Nieto-Torres JL, Verdia-Baguena C, Jimenez-Guardeno JM, Regla-Nava JA, Castano-Rodriguez C, Fernandez-Delgado R, Torres J, Aguilella VM, Enjuanes L (2015) Severe acute respiratory syndrome coronavirus E protein transports calcium ions and activates the NLRP3 inflammasome. *Virology* 485: 330 – 339

Nutalai R, Zhou D, Tuekprakhon A, Ginn HM, Supasa P, Liu C, Huo J, Mentzer AJ, Duyvesteyn HME, Dijokaite-Guraliuc A *et al* (2022) Potent cross-reactive antibodies following omicron breakthrough in vaccinees. *Cell* 185: 2116 – 2131.e18

Papa G, Mallery DL, Albecka A, Welch LG, Cattin-Ortola J, Luptak J, Paul D, McMahon HT, Goodfellow IG, Carter A *et al* (2021) Furin cleavage of SARS-CoV-2 spike promotes but is not essential for infection and cell-cell fusion. *PLoS Pathog* 17: e1009246

Penelova A, Richman L, Neupert B, Simanis V, Kuhn LC (2005) Analysis of the contribution of changes in mRNA stability to the changes in steady-state levels of cyclin mRNA in the mammalian cell cycle. *FEBS J* 272: 5217 – 5229

Pinto LH, Lamb RA (2006) The M2 proton channels of influenza a and B viruses. *J Biol Chem* 281: 8997 – 9000

Pinto LH, Holsinger LJ, Lamb RA (1992) Influenza virus M2 protein has ion channel activity. *Cell* 69: 517 – 528

Plescia CB, David EA, Patra D, Sengupta R, Amiar S, Su Y, Stahelin RV (2021) SARS-CoV-2 viral budding and entry can be modeled using BSL-2 level virus-like particles. *J Biol Chem* 296: 100103

Reuschl AK, Thorne LG, Zuliani-Alvarez L, Bouhaddou M, Obernier K, Hiatt J, Soucheray M, Turner J, Fabius JM, Nguyen GT *et al* (2021) Host-directed therapies against early-lineage SARS-CoV-2 retain efficacy against B.1.1.7 variant. *bioRxiv* https://doi.org/10.1101/2021.01.24.427991 [PREPRINT]

Rihn SJ, Merits A, Bakshi S, Turnbull ML, Wickenhagen A, Alexander AJT, Baillie C, Brennan B, Brown F, Brunker K *et al* (2021) A plasmid DNA-launched SARS-CoV-2 reverse genetics system and coronavirus toolkit for COVID-19 research. *PLoS Biol* 19: e3001091

Sakaue-Sawano A, Kurokawa H, Morimura T, Hanyu A, Hama H, Osawa H, Kashiwagi S, Fukami K, Miyata T, Miyoshi H *et al* (2008) Visualizing spatiotemporal dynamics of multicellular cell-cycle progression. *Cell* 132: 487 – 498

Schubert K, Karousis ED, Jomaa A, Scaiola A, Echeverria B, Gurzeler LA, Leibundgut M, Thiel V, Muhlemann O, Ban N (2020) Author correction: SARS-CoV-2 Nsp1 binds the ribosomal mRNA channel to inhibit translation. *Nat Struct Mol Biol* 27: 1094

Singh Tomar PP, Arkin IT (2020) SARS-CoV-2 E protein is a potential ion channel that can be inhibited by gliclazide and memantine. *Biochem Biophys Res Commun* 530: 10 – 14

Siu YL, Teoh KT, Lo J, Chan CM, Kien F, Escriou N, Tsao SW, Nicholls JM, Altmeyer R, Peiris JS *et al* (2008) The M, E, and N structural proteins of the severe acute respiratory syndrome coronavirus are required for efficient assembly, trafficking, and release of virus-like particles. *J Virol* 82: 11318 – 11330

Sun P, Wu H, Huang J, Xu Y, Yang F, Zhang Q, Xu X (2018) Porcine epidemic diarrhea virus through p53-dependent pathway causes cell cycle arrest in the G0/G1 phase. *Virus Res* 253: 1 – 11

Surjit M, Liu B, Chow VT, Lal SK (2006) The nucleocapsid protein of severe acute respiratory syndrome-coronavirus inhibits the activity of cyclin-cyclin-dependent kinase complex and blocks S phase progression in mammalian cells. *J Biol Chem* 281: 10669 – 10681

Syed AM, Taha TY, Tabata T, Chen IP, Ciling A, Khalid MM, Sreekumar B, Chen PY, Hayashi JM, Soczek KM *et al* (2021) Rapid assessment of SARS-CoV-2 evolved variants using virus-like particles. *Science* 374: 1626 – 1632

Verdia-Baguena C, Nieto-Torres JL, Alcaraz A, DeDiego ML, Torres J, Aguilella VM, Enjuanes L (2012) Coronavirus E protein forms ion channels with functionally and structurally-involved membrane lipids. *Virology* 432: 485 – 494

V'Kovski P, Kratzel A, Steiner S, Stalder H, Thiel V (2021) Coronavirus biology and replication: implications for SARS-CoV-2. *Nat Rev Microbiol* 19: 155 – 170

Wang W, Caldwell MC, Lin S, Furneaux H, Gorospe M (2000) HuR regulates cyclin a and cyclin B1 mRNA stability during cell proliferation. *EMBO J* 19: 2340 – 2350

Wang Z, Zhao Y, Wang Q, Xing Y, Feng L, Kong J, Peng C, Zhang L, Yang H, Lu M (2021) Identification of proteasome and caspase inhibitors targeting SARS-CoV-2 M(pro). *Signal Transduct Target Ther* 6: 214

Wilson L, McKinlay C, Gage P, Ewart G (2004) SARS coronavirus E protein forms cation-selective ion channels. *Virology* 330: 322 – 331

Xia B, Shen X, He Y, Pan X, Liu FL, Wang Y, Yang F, Fang S, Wu Y, Duan Z *et al* (2021) SARS-CoV-2 envelope protein causes acute respiratory distress syndrome (ARDS)-like pathological damages and constitutes an antiviral target. *Cell Res* 31: 847 – 860

Xu LH, Huang M, Fang SG, Liu DX (2011) Coronavirus infection induces DNA replication stress partly through interaction of its nonstructural protein 13 with the p125 subunit of DNA polymerase delta. *J Biol Chem* 286: 39546 – 39559

Xu R, Shi M, Li J, Song P, Li N (2020) Construction of SARS-CoV-2 virus-like particles by mammalian expression system. *Front Bioeng Biotechnol* 8: 862

Yamasoba D, Kimura I, Nasser H, Morioka Y, Nao N, Ito J, Uriu K, Tsuda M, Zahradnik J, Shirakawa K *et al* (2022) Virological characteristics of the SARS-CoV-2 omicron BA.2 spike. *Cell* 185: 2103 – 2115.e9

Yuan X, Wu J, Shan Y, Yao Z, Dong B, Chen B, Zhao Z, Wang S, Chen J, Cong Y (2006) SARS coronavirus 7a protein blocks cell cycle progression at G0/G1 phase via the cyclin D3/pRb pathway. *Virology* 346: 74 – 85

Yuan X, Yao Z, Wu J, Zhou Y, Shan Y, Dong B, Zhao Z, Hua P, Chen J, Cong Y (2007) G1 phase cell cycle arrest induced by SARS-CoV 3a protein via the cyclin D3/pRb pathway. *Am J Respir Cell Mol Biol* 37: 9 – 19

Yurkovetskiy L, Wang X, Pascal KE, Tomkins-Tinch C, Nyalile TP, Wang Y, Baum A, Diehl WE, Dauphin A, Carbone C *et al* (2020) Structural and functional analysis of the D614G SARS-CoV-2 spike protein variant. *Cell* 183: 739 – 751.e8

Zhang F, Hatziioannou T, Perez-Caballero D, Derse D, Bieniasz PD (2006) Antiretroviral potential of human tripartite motif-5 and related proteins. *Virology* 353: 396 – 409

Zhang J, Cruz-Cosme R, Zhuang MW, Liu D, Liu Y, Teng S, Wang PH, Tang Q (2020) A systemic and molecular study of subcellular localization of SARS-CoV-2 proteins. *Signal Transduct Target Ther* 5: 269

Zhang Y, Chen Y, Li Y, Huang F, Luo B, Yuan Y, Xia B, Ma X, Yang T, Yu F *et al* (2021) The ORF8 protein of SARS-CoV-2 mediates immune evasion through down-regulating MHC-iota. *Proc Natl Acad Sci U S A* 118: e2024202118

**License:** This is an open access article under the terms of the Creative Commons Attribution License, which permits use, distribution and reproduction in any medium, provided the original work is properly cited.

# Expanded View Figures

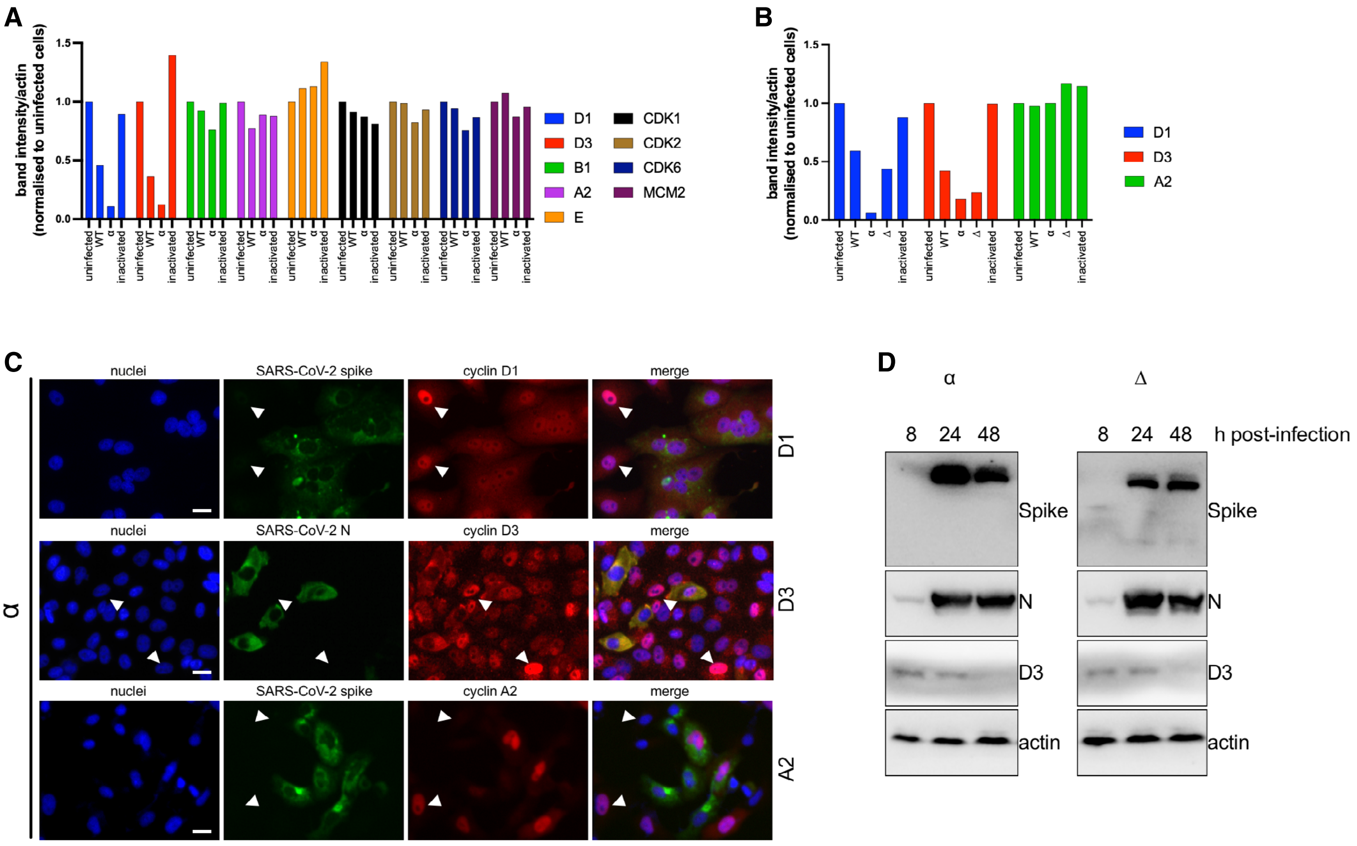


**Figure EV1. Densitometry data from cell lysates from SARS-CoV-2-infected cells.**

A, B ImageJ was used to record densitometry of bands from Fig 1A and D. All band densities were normalised to actin and further normalised to uninfected cells (=1). (A) VERO AT2 cell line. (B) A549 AT2 cell line.

C VERO AT2 cells were infected with Alpha (α) SARS-CoV-2 variant. Cells were fixed 24 h post-infection and stained for viral proteins and cyclins. Arrowheads highlight uninfected cells and cyclin D/A nuclear localisation. Arrowheads: Nuclear cyclin staining in uninfected cells. Scale bars: 20 μm.

D Calu3 cells were infected with Alpha (α) and Delta (Δ) SARS-CoV-2 variants at MOI 0.1. Cells were lysed 8, 24 and 48 h post-infection and viral proteins and cyclin D3 expression were analysed by western blot. N, nucleocapsid.

Source data are available online for this figure.


© 2022 The Authors

**Figure EV2. SARS-CoV-2 infection of VERO AT2 arrests cells in S and G2/M phases.**

A Fluorescence ubiquitination cell cycle indicator (Fucci) cell cycle sensor is a two-colour (red and green) indicator. Red: RFP-Cdt1 protein is expressed in G1 phase. GFP-geminin protein is expressed in S, G2 and M phases. Both proteins are expressed in the early S phase (both red and green colours).

B Cell cycle analysis can be performed using flow cytometry. G0/neg population of cells cannot be analysed as it comprises of cells that are in G0 phase and/or were not transduced by Fucci-containing lentiviral particles.

C Automated microscope platform and ImageJ and/or Harmony imaging software (PerkinElmer) analysis can be used to study cell cycle changes. Scale bar: 20 μm.

D–F (D) VERO AT2 cells were transduced with Fucci-containing lentiviral particles for 18 h and infected with SARS-CoV-2 WT in the absence (−) or presence of Chloroquine (CQ), Remdesivir (RVD) or infected with heat-inactivated virus for additional 24 h. The percentage of infected cells was determined by staining of SARS-CoV-2 nucleocapsid (NP) in infected cells using flow cytometry. (E) Western blot for SARS-CoV-2 spike protein as a measure of infection in cells. (F) Analysis of cell cycle phases. $n = 3$ biological replicates; Statistical analysis was performed using two-sided unpaired Student's $t$-tests; ns, non-significant; ***$P < 0.001$; **$P < 0.01$. Bars indicate mean with SD.

G, H VERO AT2 cells were transduced with Fucci-containing lentiviral particles for 18 h and infected with SARS-CoV-2 WT at different MOI for 24 h. (G) Cells were lysed and used for Western blot. (H) Cells and their cell cycle status were analysed using Flow cytometry. $n = 3$ biological replicates; one-way ANOVA with Dunnett's multiple comparisons test: ***$P < 0.001$; **$P < 0.01$. Bars indicate the mean with SD.

I, J A549 AT2 cells were transduced with Fucci-containing lentiviral particles for 18 h and infected with SARS-CoV-2 variants for an additional 24 h. Comparison of uninfected cell populations from truly uninfected cells (not exposed to virus, uninfected) and cells exposed to SARS-CoV-2 but uninfected (nucleocapsid negative, uninfected (INF)) or infected (nucleocapsid positive). (I) Example of gating strategy for cell cycle analysis. (J) Quantification of cell cycle arrest in early S phase after exposure of SARS-CoV-2 variants. α, Alpha; Δ, Delta; WT, Wuhan. $n = 3$ biological replicates; two-way ANOVA test: ns, non-significant; ****$P < 0.0001$; ***$P < 0.001$; **$P < 0.01$. Bars indicate the mean with SD.

Source data are available online for this figure.

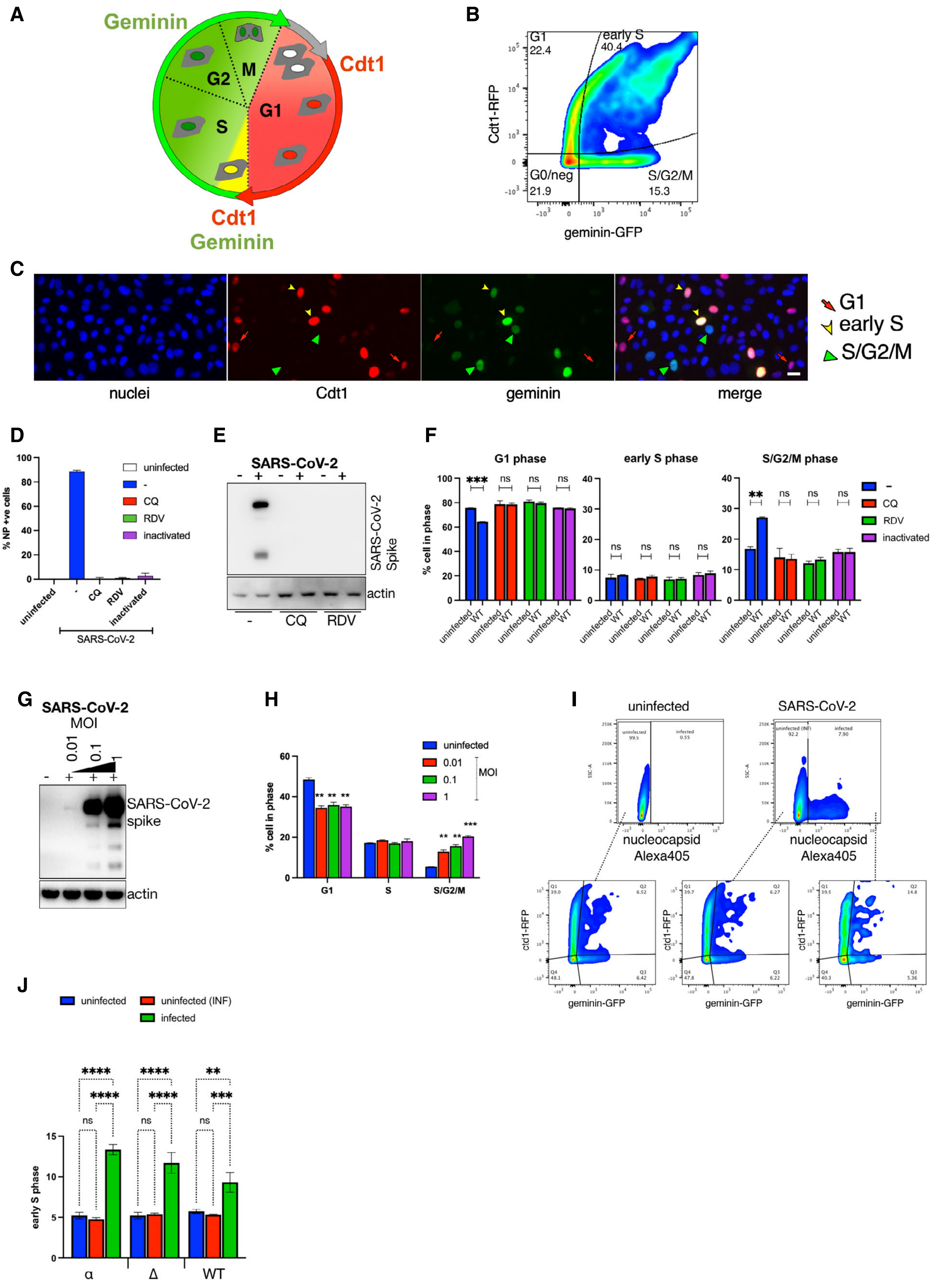


**Figure EV2.**

 © 2022 The Authors

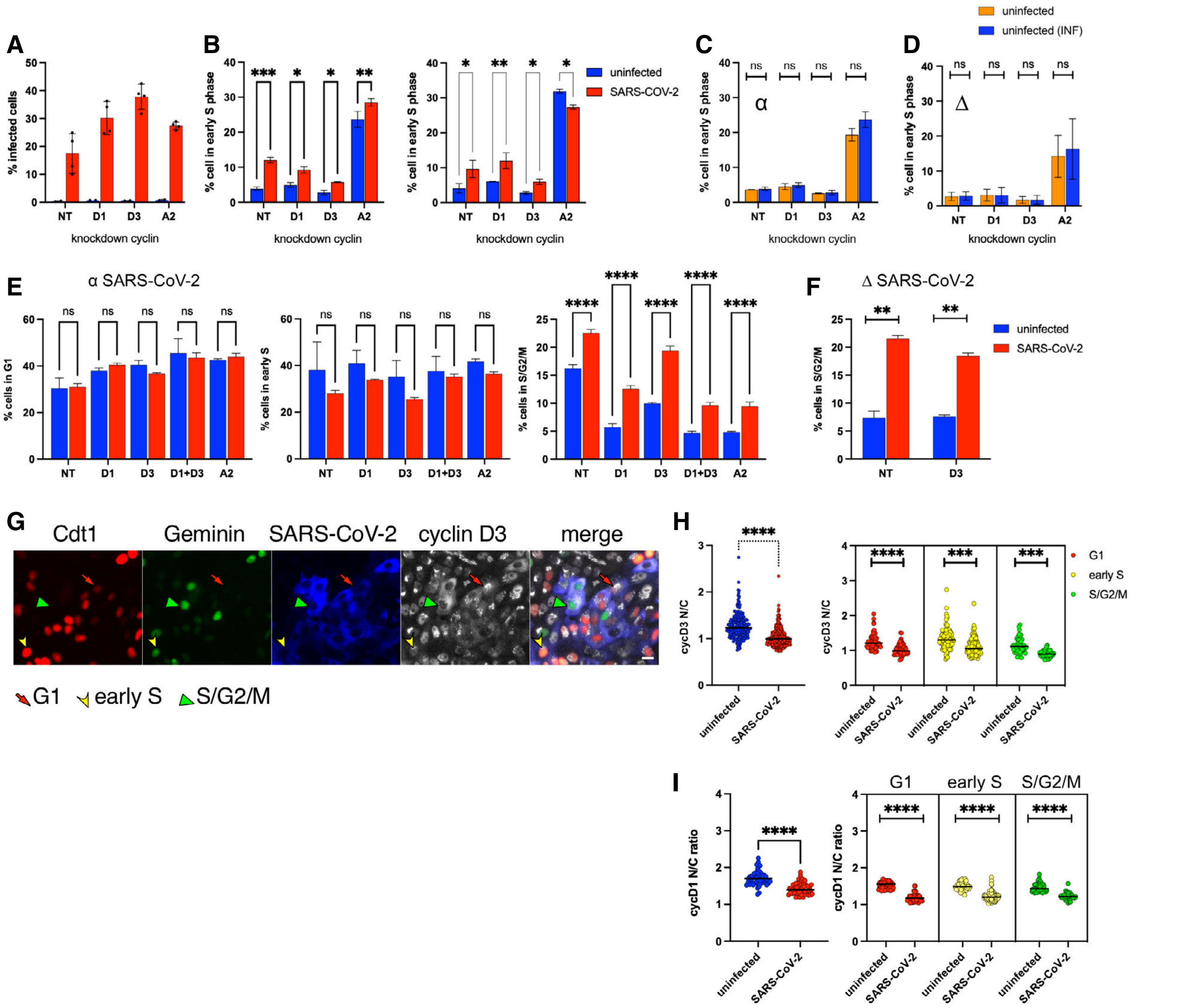


**Figure EV3. SARS-CoV-2-mediated depletion of D-cyclins is cell cycle arrest independent.**

A–D A549 AT2 cells were depleted for D and A2 cyclins and 18 h later infected with Alpha variant SARS-CoV-2 for 24 h. Cells were fixed, stained for SARS-CoV-2 nucleocapsid and analysed for infection and Fucci cell cycle sensor. (A) Percentage of infected cells in cells depleted for cyclins. $n = 2$ biological replicates. Bars indicate the mean with SD. (B) Flow cytometry analysis of early S cell cycle phase comparing cyclin D1, D3 and A2 knockdown to NT (non-target siRNA) in two independent experiments in duplicates. Statistical analysis was performed using two-sided unpaired Student's *t*-tests; ns, non-significant; ****P* < 0.001; ***P* < 0.01; **P* < 0.1. Bars indicate the mean with SD. (C, D) Comparison of uninfected cell populations from truly uninfected cells (not exposed to virus and uninfected) and cells exposed to SARS-CoV-2 but uninfected (nucleocapsid negative and uninfected (INF)). A549 AT2 cells were transduced with Fucci-containing lentiviral particles for 18 h and infected with (C) Alpha and (D) Delta SARS-CoV-2 variants for an additional 24 h. $n = 3$ biological replicates. Statistical analysis was performed using two-sided unpaired Student's *t*-test: ns, non-significant. Bars indicate the mean with SD.

E–I VERO AT2 cells were transduced with VSV-G pseudotyped Fucci-containing lentiviral particles and 18 h later infected with Alpha SARS-CoV-2. Cells were fixed and stained for SARS-CoV-2 nucleocapsid, D-cyclins and analysed for infection and Fucci cell cycle sensor 24 h later. Statistical analysis was performed using two-sided unpaired Student's *t*-tests; ns, non-significant. Bars indicate the mean with SD. (E) VERO AT2 cells infected with Alpha variant. Flow cytometry analysis of cell cycle comparing cyclin D1, D3 and combined D1 + D3 knockdown to NT (non-target siRNA) in uninfected and SARS-CoV-2-infected cells. The plot is an example of three biological replicates in technical duplicates. Statistical analysis was performed using two-sided unpaired Student's *t*-test: ns, non-significant; *****P* < 0.0001. Bars indicate the mean with SD. (F) VERO AT2 cells infected with Delta variant. Flow cytometry analysis of S/G2/M cell cycle phase comparing cyclin D3 knockdown to NT (non-target siRNA). $n = 3$ biological replicates. Statistical analysis was performed using two-sided unpaired Student's *t*-test: ns, non-significant; ***P* < 0.01. Bars indicate the mean with SD. (G) Example of acquisition using the automated microscopic platform. Cells are identified for infection, cell cycle (Red/arrow = G1phase; Green/arrowhead = S/G2/M; Red+Green/arrowhead = early S) and expression of cyclin D3. Scale bar: 20 µm. (H, I) Quantification of cyclin D3 relocalisation from the nucleus to cytoplasm and correlation with cell cycle phases using ImageJ and Harmony (PerkinElmer). (H) Cyclin D3. (I) Cyclin D1. At least 50–200 cells were analysed in each condition. Bars indicate the mean with SD. Statistical analysis was performed using two-sided unpaired Student's *t*-test: *****P* < 0.0001; ****P* < 0.001.

Source data are available online for this figure.

**Figure EV4. Cyclin D3 associates with E and M proteins.**

A Western blot of cell lysates from cell lines used in this study, detecting endogenous expression of cyclin D1 and D3.

B 293T cells were contransfected with HA-cyclin D3 and SARS-CoV-2 spike, or Strep-tagged E, M, N or nsp9. Whole-cell lysates show expression levels of protein input into immunoprecipitation.

C–E Immunoprecipitation was performed using (C) mouse anti-cyclin D3 antibody, (D) anti-HA antibody and (E) anti-Strep beads. The immunoprecipitates were blotted and stained with anti-Strep, anti-HA and anti-spike antibodies. *Non-specific band. N, nucleocapsid; E, envelope; M, membrane; S, spike.

Source data are available online for this figure.

 © 2022 The Authors

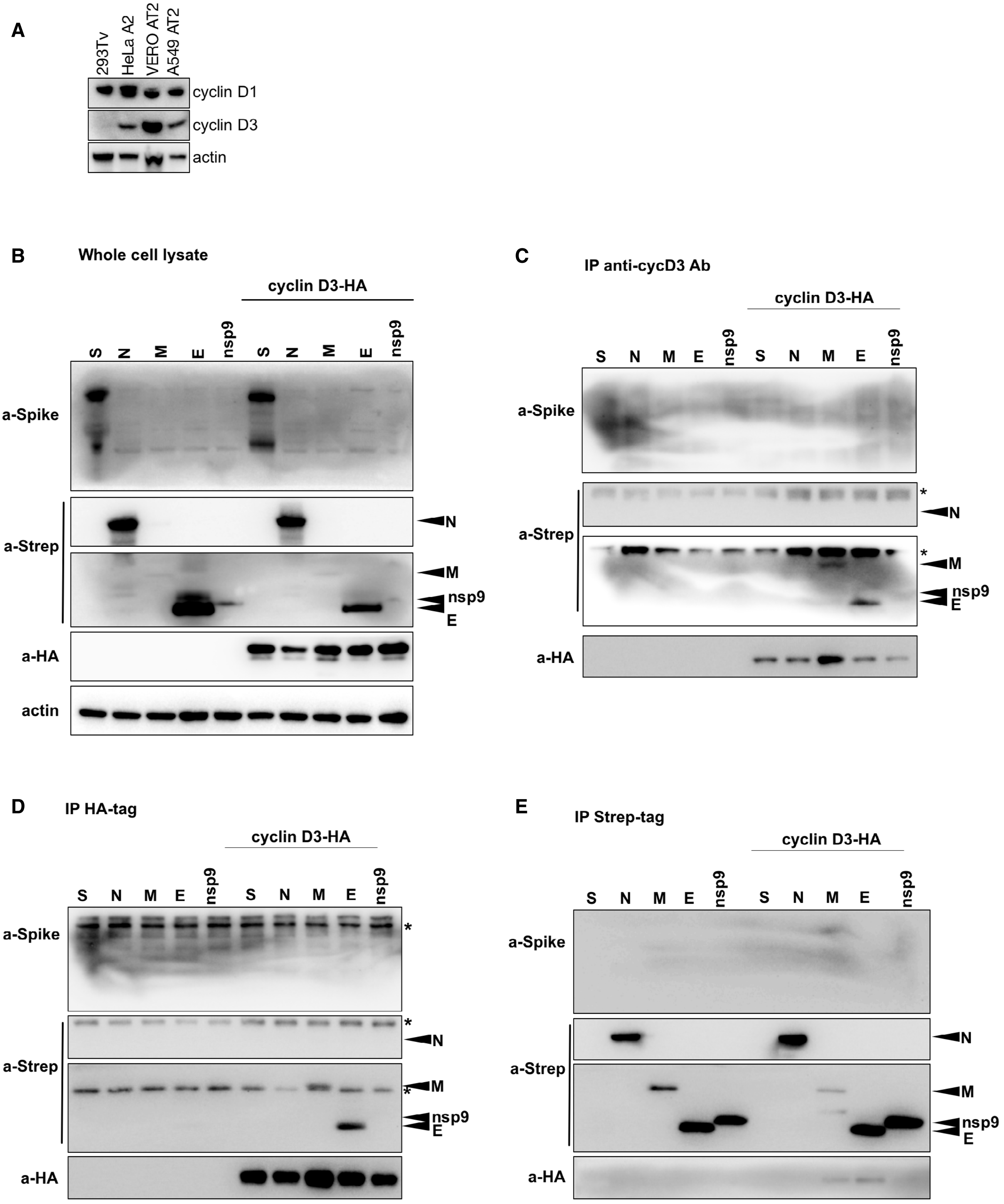
A
293Tv
HeLa A2
VERO AT2
A549 AT2
cyclin D1
cyclin D3
actin
B
Whole cell lysate
cyclin D3-HA
S N M E nsp9 S N M E nsp9
a-Spike
a-Strep
N
M
nsp9
E
a-HA
actin
C
IP anti-cycD3 Ab
cyclin D3-HA
S N M E nsp9 S N M E nsp9
a-Spike
a-Strep
*
N
M
nsp9
E
a-HA
D
IP HA-tag
cyclin D3-HA
S N M E nsp9 S N M E nsp9
a-Spike
*
a-Strep
N
M
nsp9
E
a-HA
E
IP Strep-tag
cyclin D3-HA
S N M E nsp9 S N M E nsp9
a-Spike
a-Strep
N
M
nsp9
E
a-HA

Figure EV4.

**Figure EV5. Envelope and membrane proteins are not responsible for cyclin D degradation.**

293T cells were cotransfected with SARS-CoV-2 genes (tagged with Strep-Tag) and cyclin D3.

A Cell lysates were collected 24 h post-transfection and subjected to western blotting.

B Cyclin D3 expression/degradation was quantified as cyclin D3 band intensity and normalised to actin. $n = 5$ biological replicates; ordinary one-way ANOVA with Dunnett's multiple comparisons test: ***$P < 0.001$; *$P < 0.1$. Bars indicate the mean with SD.

C, D Examples of two independent experiments demonstrating experimental variation. EV, empty vector; GFP, Strep-tagged GFP expressed.

Source data are available online for this figure.

 © 2022 The Authors

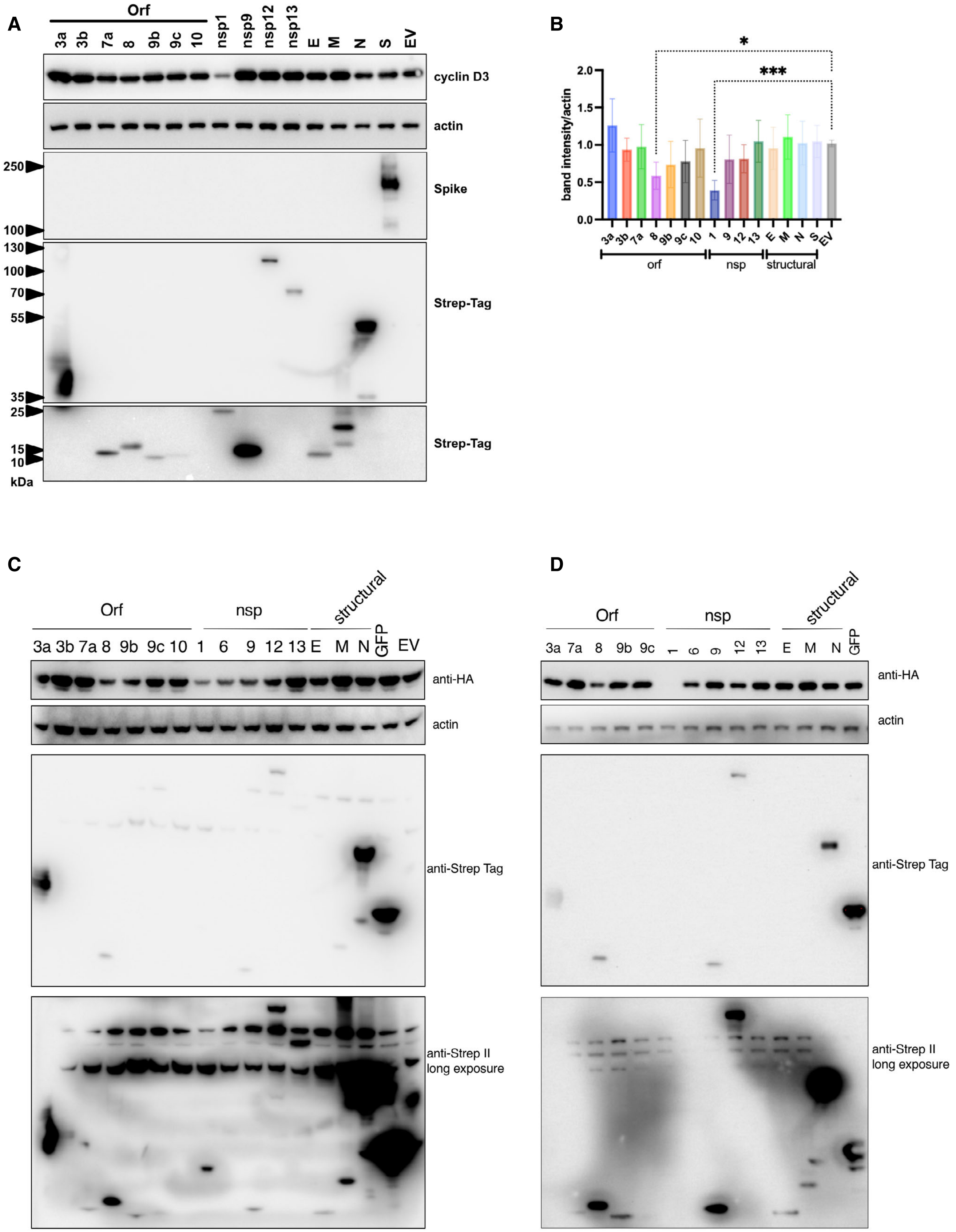
A
Orf
3a 3b 7a 8 9b 9c 10 nsp1 nsp9 nsp12 nsp13 E M N S EV
cyclin D3
actin
Spike
Strep-Tag
Strep-Tag
250
100
130
100
70
55
35
25
15
10
kDa
B
band intensity/actin
2.0
1.5
1.0
0.5
0.0
*
***
3a 3b 7a 8 9b 9c 10 1 9 12 13 E M N S EV
orf
nsp
structural
C
Orf
nsp
structural
3a 3b 7a 8 9b 9c 10 1 6 9 12 13 E M N GFP EV
anti-HA
actin
anti-Strep Tag
anti-Strep II
long exposure
D
Orf
nsp
structural
3a 7a 8 9b 9c 1 6 9 12 13 E M N GFP
anti-HA
actin
anti-Strep Tag
anti-Strep II
long exposure

**Figure EV5.**

# Cyclin D3 restricts SARS-CoV-2 Envelope incorporation into virions and interferes with viral spread

**Ravi K. Gupta**[1,2,3], **Petra Mlcochova**[1,2,*]
1 Cambridge Institute of Therapeutic Immunology & Infectious Disease (CITIID), Cambridge, UK.
2 Department of Medicine, University of Cambridge, Cambridge, UK.
3 Africa Health Research Institute, Durban, South Africa.
* Corresponding author

**APPENDIX MATERIAL**

## Table of Contents

*Appendix Figure S1.* ***SARS-CoV-2 infection relocalises cyclin D3 from the nucleus in HeLa ACE2 cells.*** ........ *2*
*Appendix Figure S2.* ***Effect of proteasome inhibition on SARS-CoV-2 infection***. ........ *3*
*Appendix Figure S3.* ***Depletion of cyclins by siRNA and genome replication***. ........ *4*
*Appendix Figure S4.* ***D-cyclin knock-down arrests cell cycle in uninfected cells***. ........ *5*
*Appendix Figure S5.* ***Cyclin D3 degradation is independent from syncytium formation***. ...... *6*

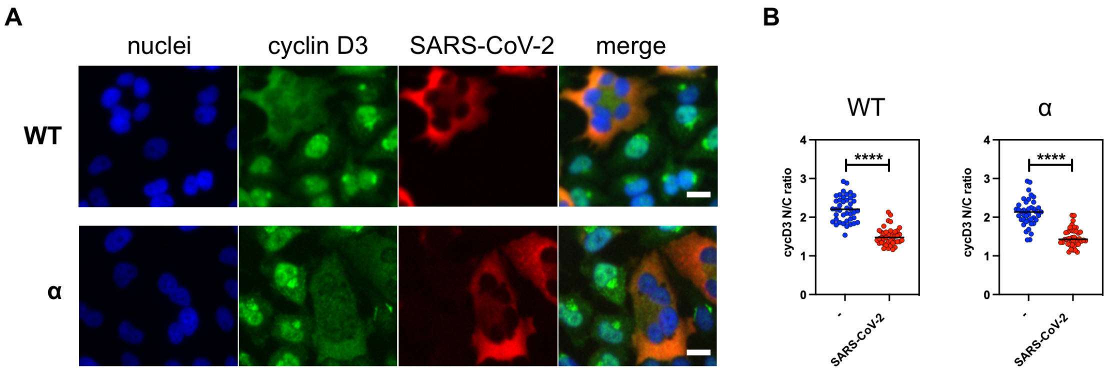


Appendix Figure S1. **SARS-CoV-2 infection relocalises cyclin D3 from the nucleus in HeLa ACE2 cells.**

(A) HeLa cells expressing ACE2 were infected with WT and α variants and 24h later fixed and stained for SARS-CoV-2 nucleocapsid and cyclin D3. Scale bar: 20μm.

(B) Quantification of ratio between nuclear and cytoplasm (N/C) staining of cyclin D3. Statistical analysis was performed using two-sided unpaired Student's t-tests; ***$p < 0.001$.

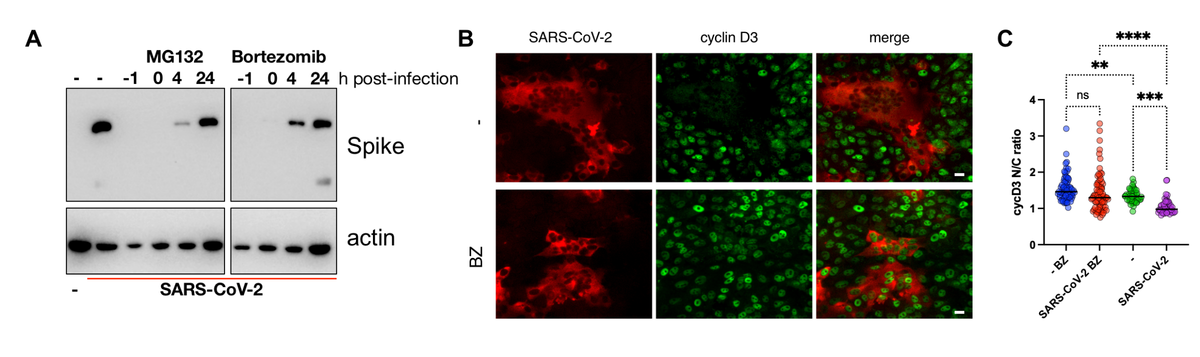


Appendix Figure S2. **Effect of proteasome inhibition on SARS-CoV-2 infection.**

(A) Proteasome inhibitors MG-132(1µM), Bortezomib (BZ, 1µM) were added to A549 AT2 cells 1h before infection (-1), at infection (0), 4h (4) and 24h (24) post-infection. Cells were infected with Delta SARS-CoV-2 variant and lysed 28h post-infection, viral protein Spike as measure of infection was detected by western blot.

(B) VERO AT2 cells were infected with Delta SARS-CoV-2 variant. Proteasome inhibitor Bortezomib (BZ, 1µM) was added to cells 8h later. Cells were fixed and stained 24h post-addition of inhibitor. Scale bar: 20µm.

(C) Quantification of D3 cyclin relocalization from nucleus after infection. Uninfected (-) and SARS-CoV-2 infected cells were identified by negative/positive nucleocapsid staining. Ratio between nuclear and cytoplasm (N/C ratio) staining intensity of cyclins was measured using ImageJ and Harmony (PerkinElmer). At least 50 cells have been counted. Ordinary two way ANOVA; ns, non-significant; ****$p < 0.0001$; ***$p < 0.001$; **$p < 0.01$.

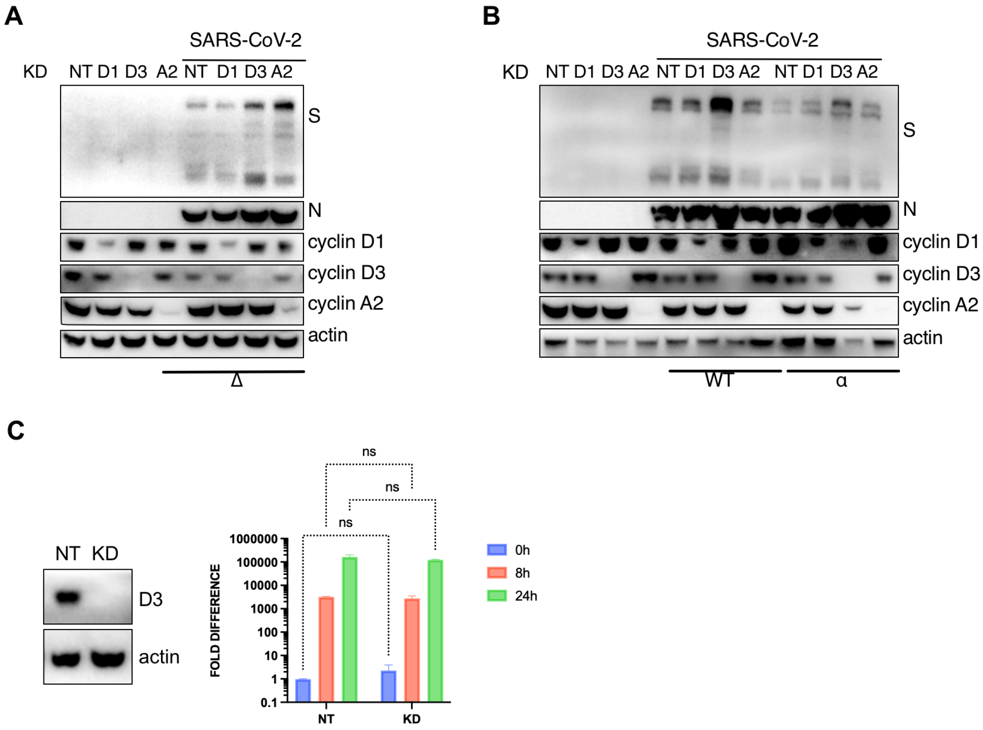


Appendix Figure S3. **Depletion of cyclins by siRNA and genome replication.**

(A,B) D and A-cyclins were depleted using siRNA. A549 AT2 Cells were infected 18h later with Delta (Δ), Alpha (α) or wild type (WT) SARS-CoV-2 variants at MOI 0.001, 0.1, 0.1 respectively. Cells were washed 4h post-infection and new media added. Cells were collected 48h later, viral proteins and cyclins detected using western blotting.

(C) **Cyclin D3 does not affect genome replication.** Cyclin D3 was depleted from VERO AT2 cells using siRNA, cells were infected 24h later and cells were collected at 8h (1 round of replication) and 24h (multiple rounds) post-infection. cDNA was prepared from isolated RNA and used in qPCR to detect nucleocapsid transcripts. n = 2 biological replicates; ordinary two way ANOVA with Dunnett's multiple comparisons test: ns, non-significant. Bars indicate mean with SD. NT, non-target siRNA. KD, knock-down.

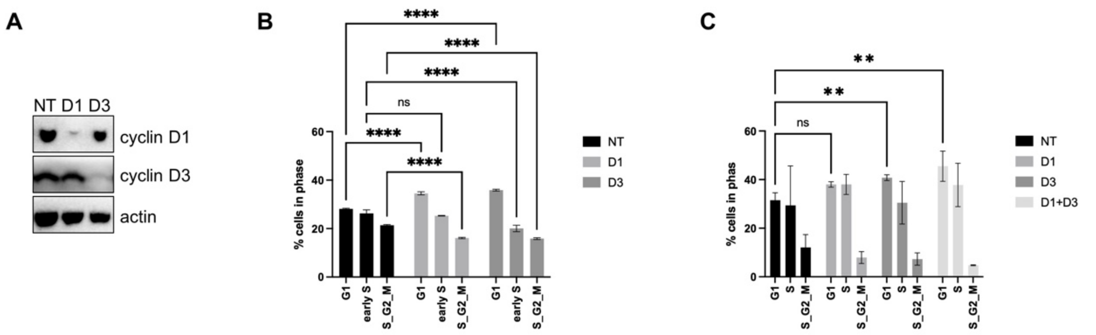


Appendix Figure S4. **D-cyclin knock-down arrests cell cycle in uninfected cells.**

(A-C) Cyclins D1, D3 were depleted by siRNA. Cells were transduced with Fucci VSV-G pseudotype virus 18h later and flow cytometry used to identify cell cycle phases 24h later.

(A) Western blot of cell lysates from A549 AT2 cells depleted for various cyclins.

(B) Flow cytometry analysis of cell cycle comparing cyclin D1, D3 knockdown to NT (non-target siRNA) in A549 AT2 cells. n = 3 biological replicates; ordinary two way ANOVA with Dunnett's multiple comparisons test: ns, non-significant; ****p < 0.0001; ***p < 0.001; *p < 0.1. Bars indicate mean with SD.

(C) Flow cytometry analysis of cell cycle comparing cyclin D1, D3, combined D1+D3 knockdown to NT (non-target siRNA) in VERO AT2 cells. n = 3 biological replicates; ordinary two way ANOVA with Dunnett's multiple comparisons test: ns, non-significant; **p < 0.01; *p < 0.1. Bars indicate mean with SD.

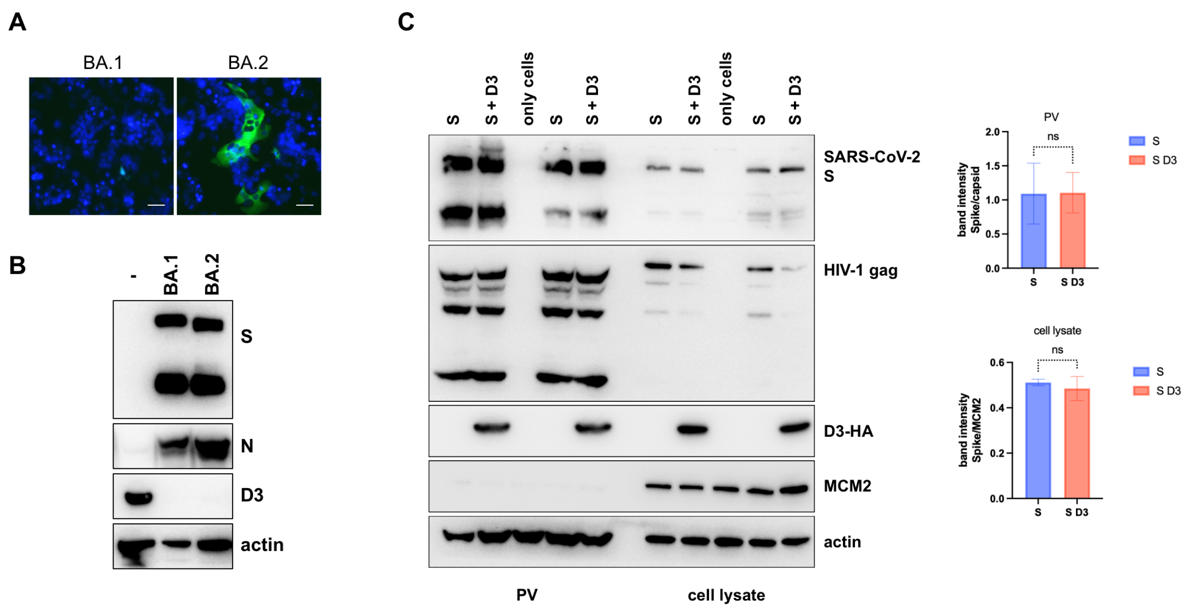


Appendix Figure S5. **Cyclin D3 degradation is independent from syncytium formation. Cyclin D3 does not change Spike incorporation into pseudotyped viral particles.**

(A,B) **Cyclin D3 degradation is independent from syncytium formation.**

(A) GFP split system was used to visualise syncytia formation after expression of Omicron Spike protein from lineages BA.1 and BA.2. Scale bar: 20µm.

(B) VERO AT2 cell were infected with BA.1 and BA.2 at MOI 0.1. Cells were collected 48h later and subjected to western blot. NP, nucleocapsid.

(C) **Cyclin D3 does not change Spike incorporation into pseudotyped viral particles.** SARS-CoV-2 Spike containing HIV-1 gag-pol based pseudotyped particles (PV) were generated in the presence or absence of cyclin D3. PV were purified and subjected to western blotting. Graphs represent quantification of immunoblot. n=2 biological replicates. Statistical analysis was performed using two-sided unpaired Student's t-tests; ns, non-significant. S, spike.
