# Supplementary material for: Cyclin D3 restricts SARS‐CoV‐2 envelope incorporation into virions and interferes with viral spread
Source: EMBO J. 2022 Oct 10;41(22):e111653. doi: 10.15252/embj.2022111653 (PMC9539236; doi:10.15252/embj.2022111653)
Supplement: Supplementary file 10 [file EMBJ-41-e111653-s008.pdf]

THE EMBO JOURNAL

Cyclin D3 restricts SARS-CoV-2 Envelope incorporation into virions and interferes with viral spread.

Petra Mlcochova and Ravindra Gupta
DOI: 10.15252/embj.2022111653

Corresponding author(s): Petra Mlcochova (pm685@cam.ac.uk)

Review Timeline:

Submission Date:	12th May 22
Editorial Decision:	9th Jun 22
Revision Received:	15th Aug 22
Editorial Decision:	12th Sep 22
Revision Received:	13th Sep 22
Accepted:	15th Sep 22

Editor: Karin Dumstrei

Transaction Report:

(Note: With the exception of the correction of typographical or spelling errors that could be a source of ambiguity, letters and reports are not edited. Depending on transfer agreements, referee reports obtained elsewhere may or may not be included in this compilation. Referee reports are anonymous unless the Referee chooses to sign their reports.)

Dear Petra,

Thank you for submitting your manuscript to The EMBO Journal. Your study has now been seen by two referees and their comments are provided below.

As you can see from the comments, the referees find the analysis interesting. However, they also both find that we would some further mechanistic insight into how cyclin D3 regulates SARS-CoV-2 replication. The referees also find that some of the findings need to be extended primary cells. Should you be able to extend the findings along the lines as suggested by the referees then we would be interested in considering a revised version.

I think it would be helpful to discuss the revisions further and I am available to do so via email or a video call.

When preparing your letter of response to the referees' comments, please bear in mind that this will form part of the Review Process File, and will therefore be available online to the community. For more details on our Transparent Editorial Process, please visit our website: https://www.embopress.org/page/journal/14602075/authorguide#transparentprocess

Thank you for the opportunity to consider your work for publication. I look forward to discussing the revisions further.

with best wishes

Karin

Karin Dumstrei, PhD
Senior Editor
The EMBO Journal

Instructions for preparing your revised manuscript:

I have attached a guide with helpful tips on how to prepare the revised version.

Guide For Authors: https://www.embopress.org/page/journal/14602075/authorguide

We realize that it is difficult to revise to a specific deadline. In the interest of protecting the conceptual advance provided by the work, we recommend a revision within 3 months (7th Sep 2022).

As a matter of policy, competing manuscripts published during this period will not negatively impact on our assessment of the conceptual advance presented by your study. However, we request that you contact the editor as soon as possible upon publication of any related work, to discuss how to proceed.

If you require more time to complete the revisions please contact me as we can grant an extension.

Use the link below to submit your revision:

https://emboj.msubmit.net/cgi-bin/main.plex

Referee #1:

The current MS from Mlcochova and Gupta provides evidence that SARS-CoV-2 infection destablizes D-type Cyclins, causing cell cycle arrest in S phase. Destablization was proteasome dependent. Knockdown of Cyclin D3 potentiated SARS-CoV-2 replication. Lastly D3 interacts with E and M in pulldowns and the authors propose this affects viral assembly/spike function.

This is a set of very nicely performed experiments that make a strong case that D cyclins impose a block to SARS-CoV-2 replication. What is less well developed is the "why" and the "how". While the authors have shown that D3 interacts with the E/M complex, they haven't identified the determinants, or shown what that interaction does to the subcellular distribution of E and M, or the processing of S. They only use a syncitium assay, the relevance of which to virus replication is not clear.
• Are E or M sufficient to induce D3 degradation? If not, which viral protein does?
• How does D3 affect virus assembly or the incorporation of S in virions? Is genome replication affected - as might be expected if

degradation was required to preserve NTPs?
• Is D3 degradation dependent on its destruction box, and does enforced expression of a D3delDBOX restrict virus replication.
• Is D3 degradtion dependent on syncytium formation - is it antiviral against a virus without an FCS?
I would encourage the authors to tackle some or all of these questions to gain better mechanistic insight as to what D3 is doing.

Referee #2:

Upon infection viral pathogens face cell intrinsic defences in human cells, often mediated by so-called restriction factors, that reduce viral replication. Thus, successful viruses like SARS-CoV-2 evolved strategies to counteract the activity of restriction factors to convert the host cell into an efficient virus replication facility.
In this manuscript, the authors propose that SARS-CoV-2 infection causes degradation and relocalisation of the cell cycle proteins cyclin D1 and D3. This relocalisation was suggested to promote proteasomal-dependent degradation. Infection by SARS-CoV-2 induce a cell cycle arrest, maybe in a cell type/organism dependent-manner. However, cyclin D targeting seemingly does not depend/impact the infection induced cell cycle arrest. Instead, especially cyclin D3 was shown by the authors to elicit an anti-viral effect, which may be counteracted by degradation. Mechanistically, the authors suggest that Cyclin D3 affect assembly of new virions due to its interaction with M and E protein of SARS-CoV-2.
Overall, the experiments are performed very well and generally support the conclusions of the manuscript. The manuscript itself is written in a brief but very concise style. The topic is relevant, interesting and timely: Cell cycle independent roles of cyclins in restriction of viruses are a novel aspect in SARS-CoV-2 biology and thus highly interesting. I suggest to consider a few points (as outlined below) to strengthen the study.
Major issues:
- The authors should show the impact of cyclin D3 depletion in primary human (lung, gut or heart) cells to establish relevance of the cell line experiments, which are not naturally infected by SARS-CoV-2 (TMPRSS2 and ACE2 overexpressing lines).

- The mechanistic part of the manuscript needs to be strengthened. The authors could either show the impact of cyclin D3 in more detail (e.g. is the SARS-CoV-2 particle infectivity/composition affected in the presence/absence of cyclin D3), or elucidate how the virus targets it for proteasomal degradation (e.g. which protein is responsible for relocalisation?).

- Abstract: "cyclin D depletion was independent from SARS-CoV-2 mediated cell cycle arrest". While the authors already gathered a lot of data, suggesting that cell cycle arrest induced by SARS-CoV-2 is not dependent on cyclin D3 degradation, this information needs to be strengthened by direct evidence or the conclusions rephrased. The data indicates (Fig. 3D) that cyclin D3 depletion affects the magnitude of the cell cycle arrest inducted by SARS-CoV-2. The authors need to ideally show experimentally cyclin D3 degradation by a recombinant SARS-CoV-2 that is not inducing a cell cycle arrest (see also above). Alternatively, the authors could show that in cells complemented with (degradation-resistant) cyclin D3, cell cycle arrest by SARS-CoV-2 is unaltered or show that a single protein by SARS-CoV-2 is sufficient to mediate cyclin D3 degradation without inducing cell cycle arrest.
Minor issues:
- line: 264-266: The authors should tone down the overreaching conclusion that all human vs monkey cells are differently affected by the SARS-CoV-2 cell cycle arrest, considering the limited cell line number used, and no primary human/monkey cells.
- line 110 and title: I would suggest to rephrase to "Cyclin D3 negatively regulates SARS-CoV-2" and "Cell cycle independent role of cyclin D3 in host restriction of SARS-CoV-2"
- Fig 1B, D, E. The choice of cell line model and strains, VOCs used should be reasoned in the manuscript. Ideally, the authors show the impact/degradation of cyclin D3 in one human cell line, ideally Calu-3 and/or primary cells (see first major issue) comparing the currently available VOCs.
- Fig. 1C: Was N and S stained? Why did the authors switch to N staining for the D3 sample?
- Fig. 2A: Would autophagy/lysosome inhibition (e.g. Chloroquine, Bafilomycin A1) not prevent cyclin D3 degradation?
- Fig. 2C legend: Please add the staining for SARS-CoV-2 ?
- Fig. 2C: Why were only syncytia looked at? Is the relocalisation of cyclin D3 also prevented by proteasome inhibition in non-syncytia?
- Fig. 3A: Were the lysates infected as stated in the legends?
- Fig. 3B,D: Please show the replicates as individual dots.
- Fig 5D: Are the differences between the grey bars significant?
- Fig. 6B: M is not visible in the input. The IP of Cyclin D3 for the nsp9 sample is less efficient, which may explain no binding.
- Please replace uM with µM throughout the manuscript
- Just as a comment: Please check that every person that contributed to the manuscript is listed as an author.

August 15th 2022

RE: Manuscript EMBOJ-2022-111653

“Cell cycle independent role of cyclin D3 in host restriction of SARS-CoV-2 infection”

Dear Dr. Dumstrei
Thank you for your letter and for the opportunity to respond to the reviewer’s comments. We are appreciative of the comments and suggestions made by both reviewers and believe that addressing their comments and concerns substantially improved our manuscript.
All the changes in the manuscripts have been highlighted. Please see below for point-by-point responses.

We are looking forward to hearing from you soon.

Referee #1:

The current MS from Mlcochova and Gupta provides evidence that SARS-CoV-2 infection destablizes D-type Cyclins, causing cell cycle arrest in S phase. Destablization was proteasome dependent. Knockdown of Cyclin D3 potentiated SARS-CoV-2 replication. Lastly D3 interacts with E and M in pulldowns and the authors propose this affects viral assembly/spike function.

This is a set of very nicely performed experiments that make a strong case that D cyclins impose a block to SARS-CoV-2 replication. What is less well developed is the "why" and the "how". While the authors have shown that D3 interacts with the E/M complex, they haven't identified the determinants, or shown what that interaction does to the subcellular distribution of E and M, or the processing of S. They only use a syncitium assay, the relevance of which to virus replication is not clear.
• Are E or M sufficient to induce D3 degradation? If not, which viral protein does?
• How does D3 affect virus assembly or the incorporation of S in virions? Is genome replication affected - as might be expected if degradation was required to preserve NTPs?
• Is D3 degradation dependent on its destruction box, and does enforced expression of a D3delDBOX restrict virus replication.
• Is D3 degradtion dependent on syncytium formation - is it antiviral against a virus without an FCS?
I would encourage the authors to tackle some or all of these questions to gain better mechanistic insight as to what D3 is doing.

• Are E or M sufficient to induce D3 degradation? If not, which viral protein does?

RESPONSE:
We have now included experiments showing that E and M are not sufficient for cyclin D3 degradation. These experiments were done by overexpression of selected SARS-CoV-2 genes/proteins and cyclin D3 in 293T cells. 293T cell do not express cyclin D3 and are thus a useful cell line to conduct these experiments in. We have identified 2 SARS-CoV-2 proteins able to impact expression levels of cyclin D3, specifically Orf8 and nsp1. We have included these results in the manuscript result and discussion section and in Expanded View Figure 5.

EV5: Envelope and Membrane proteins are not responsible for cyclin D degradation. 293T cells were co-transfected with SARS-CoV-2 genes (tagged with Strep-Tag) and cyclin D3. (A) Cell lysates were collected 24h post-transfection and subjected to western blotting. (B) Cyclin D3 expression/degradation was quantified as cyclin D3 band intensity, normalised to actin. n=5; one way ANOVA with Dunnett's multiple comparisons test: $^{***}p < 0.001$; $^{*}p < 0.1$. Bars indicate mean with SD.

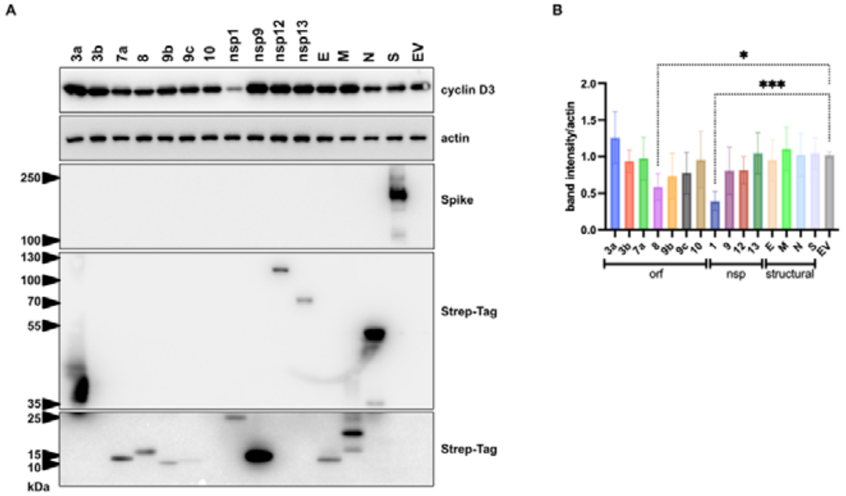

• How does D3 affect virus assembly or the incorporation of S in virions?

RESPONSE:

Virus assembly:

To understand how cyclin D3 affects virus assembly we employed a SARS-CoV-2 Virus Like Particles (VLPs) assay. This assay uses SARS-CoV-2 Spike, Envelope, Membrane and Nucleocapsid transfection into mammalian cells to produce VLPs and has been published several times (doi: 10.1038/s41598-020-78656-w; doi: 10.1126/science.abl6184.).

We used this assay to assemble particles in the presence or absence of cyclin D3 and discovered that cyclin D3 impairs Envelope incorporation into SARS-CoV-2 virions. This is a very interesting observation as Envelope is important virus budding, morphogenesis, trafficking, and pathogenesis. In addition, recombinant CoVs lacking E exhibit significantly reduced viral titres, crippled viral maturation (doi: 10.1128/JVI.01467-06; doi: 10.1016/j.virol.2007.05.032.). Further SARS-CoV-2 E is predicted to be a viroporin. Viroporins are known to positively contribute to the viral release and facilitate the penetration of the viruses into the cell (doi: 10.1038/nrmicro2820.).Spike, Membrane or Nucleocapsid incorporation or expression in cells was not affected by presence of cyclin D3.

These data are now included in revised manuscript and in Figure 6 I-J.

Figure 6 I-J:

(H) Diagram of SARS-CoV-2 VLP construction in the presence or absence of cyclin D3 in 293T cells.

(I) Purified SARS-CoV-2 VLPs were subjected to immunoblot, and proteins visualised in VLPs and whole cell lysate (WCL).

(J) Quantification of protein incorporation (into VLPs) or expression (in WCL). n = 4; one-way ANOVA with Dunnett's multiple comparisons test: ns, non-significant; ***p < 0.001. Bars indicate mean with SD.

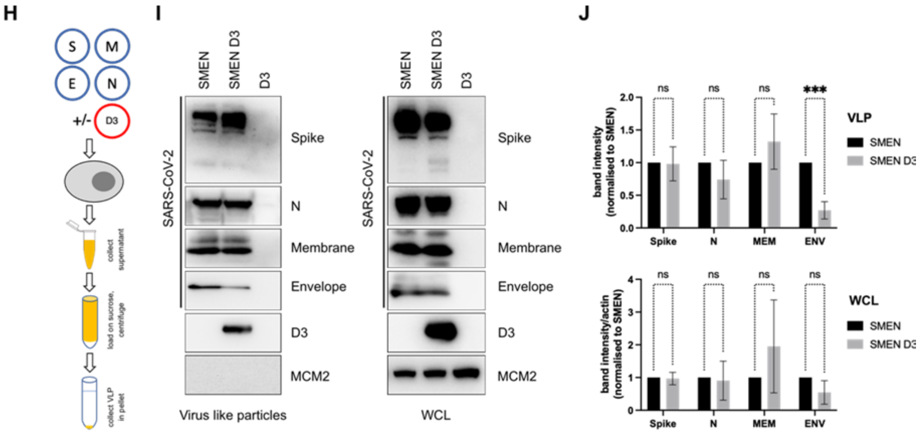

Incorporation of S in virions:

Our additional data, now included in revised manuscript show that Spike incorporation into virions is not affected by cyclin D3. Firstly, SARS-CoV-2 VLPs (see above) do not show any difference in Spike incorporation in the presence or absence of cyclin D3. Secondly, spike incorporation into Spike pseudotyped lentiviral particles, widely used to study Spike incorporation and processing of SARS-CoV-2 Spike (doi: 10.1038/s41586-022-04474-x; doi: 10.1371/journal.ppat.1009246), was not different in the presence or absence of cyclin D3. These data are now included in manuscript in Fig 6H-J and Appendix Fig S5C.

Appendix Fig S5C: **Cyclin D3 does not change Spike incorporation into pseudotyped lentiviral particles.** SARS-CoV-2 Spike containing HIV-1 gag-pol based pseudotyped particles were generated in the presence or absence of cyclin D3. PV were purified and subjected to immunoblotting. Graphs represent quantification of immunoblot. n=2. Statistical analysis was performed using two-sided unpaired Student's t-tests; ns, non-significant.

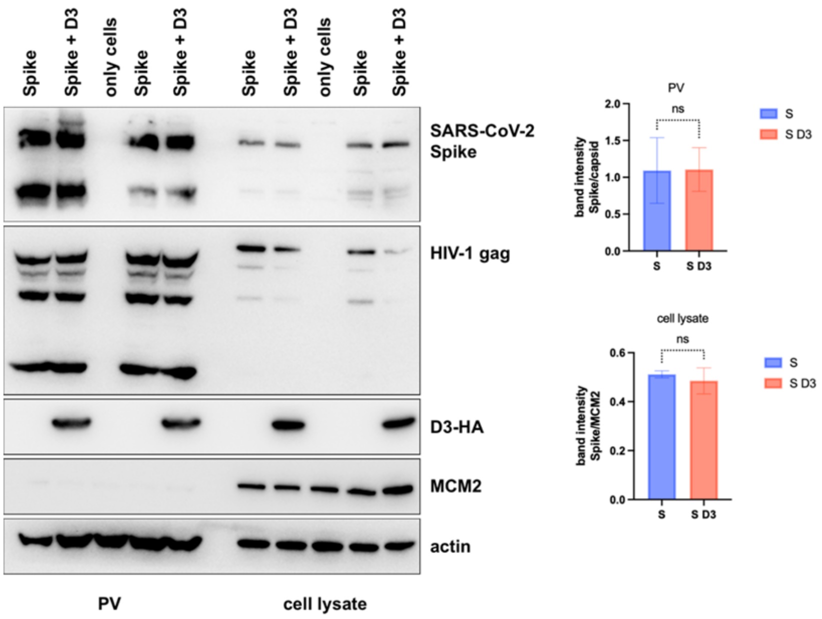

- **Is genome replication affected – as might be expected if degradation was required to preserve NTPs?**

RESPONSE:
Data presented in our manuscript suggest that the block to SARS-CoV-2 infection caused by cyclin D3 occurs later during viral assembly. We would not expect genome replication to be directly affected. To confirm this, we depleted cyclin D3 in VERO AT2 cells and infected cells with SARS-CoV-2 for 8h, allowing one round of infection and 24h, allowing multiple (~ 3) rounds of infection. RNA was isolated from cells and qPCR detecting Nucleocapsid transcripts was performed. If genome replication was affected, we would expect increase in nucleocapsid transcripts in the cells lacking cyclin D3, even after one round of infection. We did not detect any differences between cells expressing and cell depleted for cyclin D3. These data are now included in manuscript in Appendix Fig S3C.

Appendix S3C: Cyclin D3 does not affect genome replication. Cyclin D3 was depleted from VERO AT2 cells using siRNA, cells were infected 2h later and cells were collected at 6h (1 round of replication) and 24h (multiple rounds) post-infection. cDNA was prepared from isolated RNA and used in qPCR to detect nucleocapsid transcripts. N = 2; ordinary two way ANOVA with Dunnett's multiple comparisons test: ns, non-significant. Bars indicate mean with SD. NT, non-target siRNA. KD, knock-down.

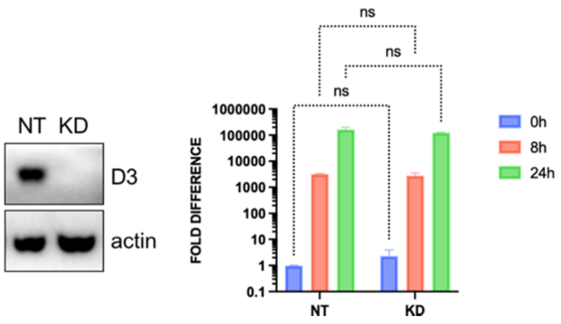

• Is D3 degradation dependent on its destruction box, and does enforced expression of a D3delDBOX restrict virus replication.

RESPONSE:
Destruction box, or D-box is the sequence required for degradation of specific cyclins. However, it is restricted to cyclins playing role in anaphase-promoting complex, cyclin A and B. Cyclin Ds do not have known D-box.

• Is D3 degradation dependent on syncytium formation - is it antiviral against a virus without an FCS?

RESPONSE:
To investigate this possibility, we infected VERO AT2 cells with a SARS-CoV-2 variant with very limited ability to form syncytia (Omicron BA.1) and with a variant Omicron BA.2 that is significantly more efficient in syncytia formation (doi: 10.1016/j.cell.2022.04.035). Our data, now included in the manuscript as Appendix Fig S5A,B, show that both variants are able to degrade cyclin D3.

Appendix Fig S5A,B: (A,B) Cyclin D3 degradation is independent of syncytium formation.

(A) GFP split system was used to visualise syncytia formation after expression of Omicron Spike protein BA.1 and BA.2.

(B) VERO AT2 cell were infected with BA.1 and BA.2 at MOI 0.1. Cells were collected 48h later and subjected to immunoblotting.

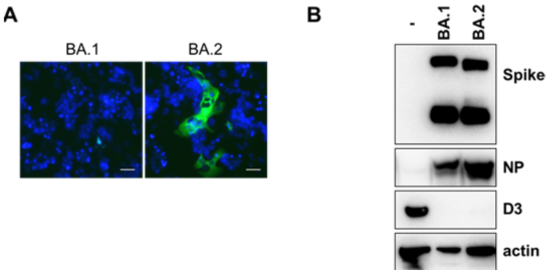

Referee #2:

Upon infection viral pathogens face cell intrinsic defences in human cells, often mediated by so-called restriction factors, that reduce viral replication. Thus, successful viruses like SARS-CoV-2 evolved strategies to counteract the activity of restriction factors to convert the host cell into an efficient virus replication facility.
In this manuscript, the authors propose that SARS-CoV-2 infection causes degradation and relocalisation of the cell cycle proteins cyclin D1 and D3. This relocalisation was suggested to promote proteasomal-dependent degradation. Infection by SARS-CoV-2 induce a cell cycle arrest, maybe in a cell type/organism dependent-manner. However, cyclin D targeting seemingly does not depend/impact the infection induced cell cycle arrest. Instead, especially cyclin D3 was shown by the authors to elicit an anti-viral effect, which may be counteracted by degradation. Mechanistically, the authors suggest that Cyclin D3 affect assembly of new virions due to its interaction with M and E protein of SARS-CoV-2.
Overall, the experiments are performed very well and generally support the conclusions of the manuscript. The manuscript itself is written in a brief but very concise style. The topic is relevant, interesting and timely: Cell cycle independent roles of cyclins in restriction of viruses are a novel aspect in SARS-CoV-2 biology and thus highly interesting. I suggest to consider a few points (as outlined below) to strengthen the study.
Major issues:
- The authors should show the impact of cyclin D3 depletion in primary human (lung, gut or heart) cells to establish relevance of the cell line experiments, which are not naturally infected by SARS-CoV-2 (TMPRSS2 and ACE2 overexpressing lines).

- The mechanistic part of the manuscript needs to be strengthened. The authors could either show the impact of cyclin D3 in more detail (e.g. is the SARS-CoV-2 particle infectivity/composition affected in the presence/absence of cyclin D3), or elucidate how the virus targets it for proteasomal degradation (e.g. which protein is responsible for relocalisation?).

- Abstract: "cyclin D depletion was independent from SARS-CoV-2 mediated cell cycle arrest". While the authors already gathered a lot of data, suggesting that cell cycle arrest induced by SARS-CoV-2 is not dependent on cyclin D3 degradation, this information needs to be strengthened by direct evidence or the conclusions rephrased. The data indicates (Fig. 3D) that cyclin D3 depletion affects the magnitude of the cell cycle arrest inducted by SARS-CoV-2. The authors need to ideally show experimentally cyclin D3 degradation by a recombinant SARS-CoV-2 that is not inducing a cell cycle arrest (see also above). Alternatively, the authors could show that in cells complemented with (degradation-resistant) cyclin D3, cell cycle arrest by SARS-CoV-2 is unaltered or show that a single protein by SARS-CoV-2 is sufficient to mediate cyclin D3 degradation without inducing cell cycle arrest.
Minor issues:
- line: 264-266: The authors should tone down the overreaching conclusion that all human vs monkey cells are differently affected by the SARS-CoV-2 cell cycle arrest, considering the limited cell line number used, and no primary human/monkey cells.
- line 110 and title: I would suggest to rephrase to "Cyclin D3 negatively regulates SARS-CoV-2" and "Cell cycle independent role of cyclin D3 in host restriction of SARS-CoV-2"
- Fig 1B, D, E. The choice of cell line model and strains, VOCs used should be reasoned in the manuscript. Ideally, the authors show the impact/degradation of cyclin D3 in one human cell line, ideally Calu-3 and/or primary cells (see first major issue) comparing the currently

available VOCs.
- Fig. 1C: Was N and S stained? Why did the authors switch to N staining for the D3 sample?
- Fig. 2A: Would autophagy/lysosome inhibition (e.g. Chloroquine, Bafilomycin A1) not prevent cyclin D3 degradation?
- Fig. 2C legend: Please add the staining for SARS-CoV-2 ?
- Fig. 2C: Why were only syncytia looked at? Is the relocalisation of cyclin D3 also prevented by proteasome inhibition in non-syncytia?
- Fig. 3A: Were the lysates infected as stated in the legends?
- Fig. 3B,D: Please show the replicates as individual dots.
- Fig 5D: Are the differences between the grey bars significant?
- Fig. 6B: M is not visible in the input. The IP of Cyclin D3 for the nsp9 sample is less efficient, which may explain no binding.
- Please replace uM with µM throughout the manuscript
- Just as a comment: Please check that every person that contributed to the manuscript is listed as an author.

Major issues:

- **The authors should show the impact of cyclin D3 depletion in primary human (lung, gut or heart) cells to establish relevance of the cell line experiments, which are not naturally infected by SARS-CoV-2 (TMPRSS2 and ACE2 overexpressing lines).**

RESPONSE:
To support the relevance of our observation we used Calu3 cell derived from human lung epithelia. These cells are known to naturally express high TMPRSS2 levels. Existing data, including our group, support that they are good model for natural SARS-CoV-2 infection and recapitulate data from primary organoids (doi: 10.1038/s41586-022-04474-x; doi: 10.1038/s41586-021-03944-y).
We have infected Calu3 cells depleted for cyclin D3 and collected cells (for western blot analysis) and viral supernatant (for TCID50 analysis). Our data show that cyclin D3 depletion increases viral titers (Figure 3E) and confirm thus our previous results from VERO AT2 and A549 AT2 cell lines. These data are now included in the manuscript in Fig 3E.

Figure 3E: Cyclin D3 was depleted using siRNA in Calu3 cells. Cells were infected 48h later with Delta SARS-CoV-2 at MOI 0.01. Cells were washed 4h post-infection and new media were added. Supernatants and cells were collected 48h later for western blot and TCID50. Graphs represent average of n=3. Statistical analysis was performed using one-way ANOVA with Dunnett's multiple comparisons test. NT, non-targeting control. ns, non-significant; *$p < 0.1$; **$p < 0.01$. Bars indicate mean with SD.

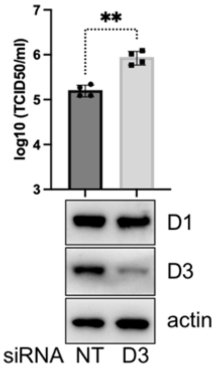

Further we have included data showing cyclin D3 degradation in Calu3 cells infected with α and Δ SARS-CoV-2 variant. Fig EV1D.

EV1D: Calu3 cells were infected with alpha (α), and delta (Δ) SARS-CoV-2 variants at MOI 0.1. Cells were lysed 8, 24 and 48h post-infection and viral, cyclin D3 expression was analysed by western blot. N, nucleocapsid.

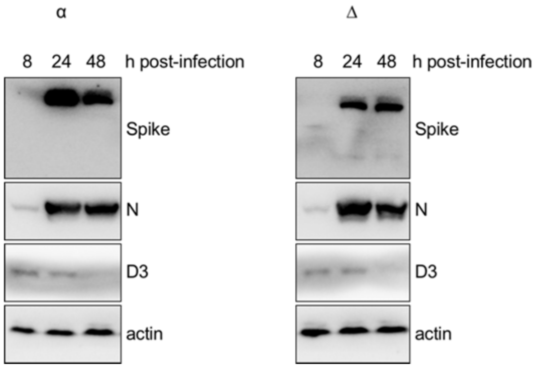

- **The mechanistic part of the manuscript needs to be strengthened. The authors could either show the impact of cyclin D3 in more detail (e.g. is the SARS-CoV-2 particle infectivity/composition affected in the presence/absence of cyclin D3), or elucidate how the virus targets it for proteasomal degradation (e.g. which protein is responsible for relocalisation?).**

RESPONSE:
SARS-CoV-2 particle infectivity/composition/ which protein is responsible for relocalisation (degradation)
We have shown in our manuscript (Figure 3) that absence of cyclin D3 increases titers of newly generated SARS-CoV-2 virions in VERO AT2, A549 AT2, and now Calu3 cells.
We have performed additional experiments to address composition of viral particles in the absence and presence of cyclin D3, now included in manuscript Figures 6, and Appendix Fig S5.

Figure 6 I-J: Cyclin D3 impairs SRS-CoV-2 Envelope incorporation into virions

(H) Diagram of SARS-CoV-2 VLPs construction in the presence or absence of cyclin D3 in 293T cells.

(I) Purified SARS-CoV-2 VLPs were subjected to immunoblot, and proteins visualised in VLPs and whole cell lysate (WCL).

(J) Quantification of protein incorporation (into VLPs) or expression (in WCL). n = 4; one-way ANOVA with Dunnett's multiple comparisons test: ns, non-significant; ***$p < 0.001$. Bars indicate mean with SD.

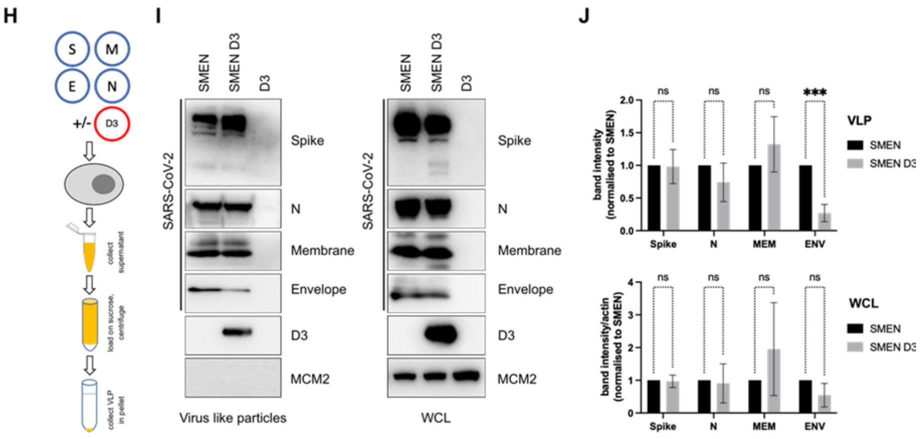

Appendix Fig S5C: Cyclin D3 does not change Spike incorporation into pseudotyped viral particles. SARS-CoV-2 Spike containing HIV-1 gag-pol based pseudotyped particles were generated in the presence or absence of cyclin D3. PV were purified and subjected to immunoblotting. Graphs represent quantification of immunoblot. n=2. Statistical analysis was performed using two-sided unpaired Student's t-tests; ns, non-significant.

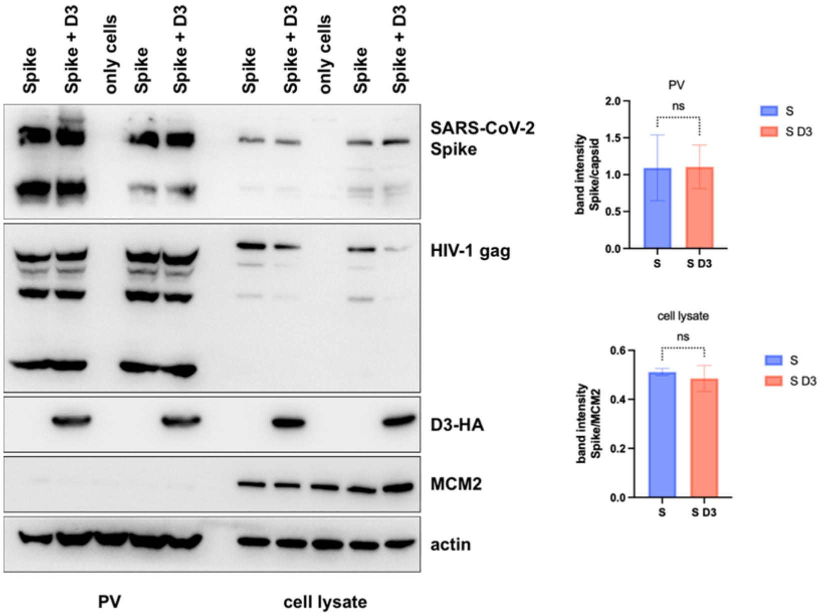

Appendix Fig S5A,B: (A,B) Cyclin D3 degradation is independent from syncytium formation.

(A) GFP split system was used to visualise syncytia formation after expression of Omicron Spike protein BA.1 and BA.2.

(B) VERO AT2 cell were infected with BA.1 and BA.2 at MOI 0.1. Cells were collected 48h later and subjected to immunoblotting.

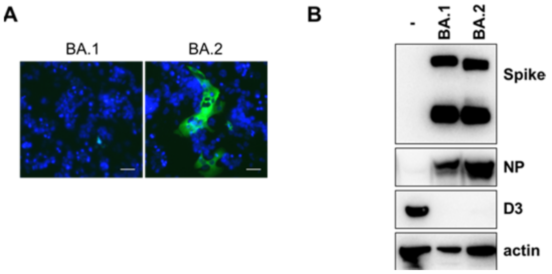

Appendix Fig S3C: Cyclin D3 does not affect genome replication. Cyclin D3 was depleted from VERO AT2 cells using siRNA, cells were infected 2h later and cells were collected at 6h (1 round of replication) and 24h (multiple rounds) post-infection. cDNA was prepared from isolated RNA and used in qPCR to detect nucleocapsid transcripts. n = 2; ordinary two way ANOVA with Dunnett's multiple comparisons test: ns, non-significant. Bars indicate mean with SD. NT, non-target siRNA. KD, knock-down.

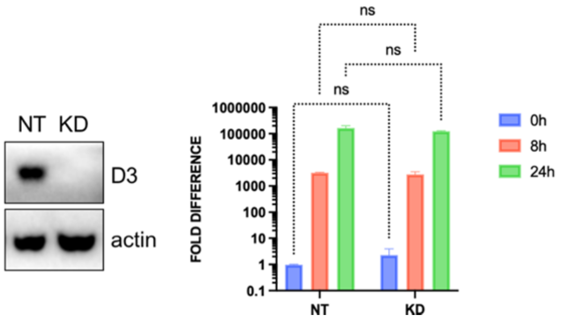

We have now included experiments showing that E and M are not sufficient for cyclin D3 degradation. These experiments were done as overexpression of selection of SARS-CoV-2 genes/proteins and cyclin D3 in 293T cells. 293T cell do not express cyclin D3 and present thus useful cell line to conduct these experiments in. We have identified 2 SARS-CoV-2 proteins able to change expression levels of cyclin D3, specifically Orf8 and nsp1. We have included these results in manuscript result and discussion section and in Expanded View Figure 5.

EV5: Envelope and Membrane proteins are not responsible for cyclin D degradation. 293T cells were co-transfected with SARS-CoV-2 genes (tagged with Strep-Tag) and cyclin D3. (A) Cell lysates were collected 24h later and subjected to immunoblotting. (B) Cyclin D3 expression/degradation was quantified as cyclin D3 band intensity, normalised to actin. n=5; ordinary one way ANOVA with Dunnett's multiple comparisons test: ***$p < 0.001$; *$p < 0.1$. Bars indicate mean with SD.

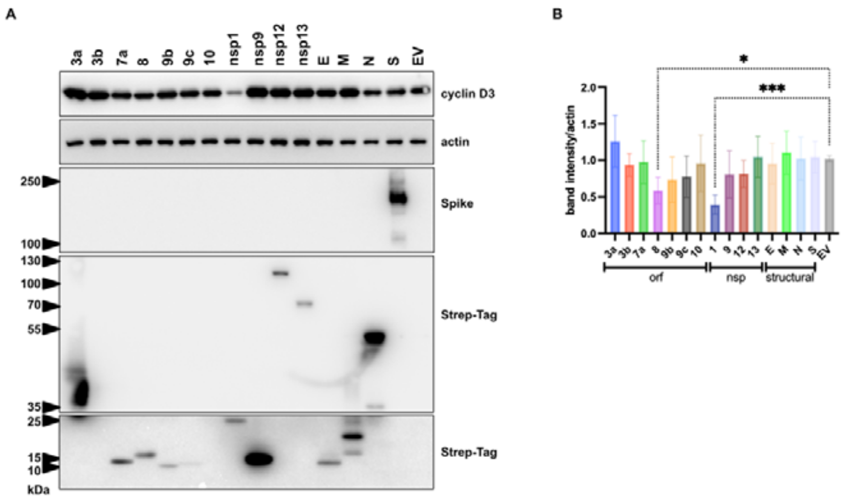

- Abstract: "cyclin D depletion was independent from SARS-CoV-2 mediated cell cycle arrest". While the authors already gathered a lot of data, suggesting that cell cycle arrest induced by SARS-CoV-2 is not dependent on cyclin D3 degradation, this information needs to be strengthened by direct evidence or the conclusions rephrased. The data indicates (Fig. 3D) that cyclin D3 depletion affects the magnitude of the cell cycle arrest inducted by SARS-CoV-2. The authors need to ideally show experimentally cyclin D3 degradation by a recombinant SARS-CoV-2 that is not inducing a cell cycle arrest (see also above). Alternatively, the authors could show that in cells complemented with (degradation-resistant) cyclin D3, cell cycle arrest by SARS-CoV-2 is unaltered or show that a single protein by SARS-CoV-2 is sufficient to mediate cyclin D3 degradation without inducing cell cycle arrest.

RESPONSE:
We have performed additional experiments as suggested by reviewer. We would like to thank reviewer for his suggestions as they made the manuscript comprehensive.
Cells have been transduced with lentivirus to deliver wild type (WT) cyclin D3 or mutant T283A cyclin D3. Mutation at T283 prevents phosphorylation, nuclear export and proteasomal degradation (Casanovas *et al.*, 2004; Cato *et al*, 2011). Cyclin D3 could be detected in non-transduced/control cells and has been completely degraded during infection. Cells complemented with cyclin D3 WT and T283A showed higher cyclin D3 expression than control but after infection only WT has been degraded while T283A cyclin D3 has been still highly expressed. Even in the presence of high levels of T283A cyclin D3 clear cell cycle arrest in early S phase has been detected in A549 AT cells (Fig 5E).

Figure 5E: A549 AT2 cells were transduced with VSV-G pseudotyped Fucci containing lentiviral particles and VSV-G pseudotyped lentiviral particles containing WT cyclin D3 or T283A mutant cyclin D3 (mutant not degraded by proteasome). Cells were infected 24h later and collected 48h post-infection for flow cytometry analysis of early S and western blot. . n=2; Ordinary two-way ANOVA with Sidak's multiple comparisons test: ns, non-significant; **p < 0.01. Bars indicate mean with SD.

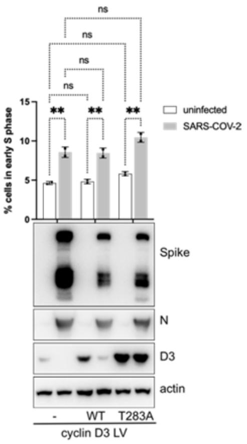

Minor issues:

- line: 264-266: The authors should tone down the overreaching conclusion that all human vs monkey cells are differently affected by the SARS-CoV-2 cell cycle arrest, considering the limited cell line number used, and no primary human/monkey cells.

We have corrected this in manuscript.

- line 110 and title: I would suggest to rephrase to "Cyclin D3 negatively regulates SARS-CoV-2" and "Cell cycle independent role of cyclin D3 in host restriction of SARS-CoV-2"

We have changed the title to "**The role of cyclin D3 in host restriction of SARS-CoV-2 infection**"

- Fig 1B, D, E. The choice of cell line model and strains, VOCs used should be reasoned in the manuscript. Ideally, the authors show the impact/degradation of cyclin D3 in one human cell line, ideally Calu-3 and/or primary cells (see first major issue) comparing the currently available VOCs.

At the time of manuscript preparation α and Δ VOC have been circulating worldwide and thus used for this study.
We have now included new data in:
EV1D: Calu3 cells have been infected with Alpha and Delta SARS-CoV-2 variant and cyclin D3 degradation was detected.
Appendix Fig S5A,B: VERO AT2 cell have been infected with Omicron VOC BA.1 and BA.2. All VOC studied do degrade cyclin D3.

All studied variants have the ability to deplete cyclin D3 in infected cells, supporting the notion that cyclin D3 degradation is universal mechanism.

- Fig. 1C: Was N and S stained? Why did the authors switch to N staining for the D3 sample?

Nucleocapsid and Spike staining are both efficient and overlay in detecting infected cells. Anti-cyclin D3 is mouse antibody, while Spike antibody is also mouse and can not be used together. For this reason, we used anti-N, which is rabbit antibody.

- Fig. 2A: Would autophagy/lysosome inhibition (e.g. Chloroquine, Bafilomycin A1) not prevent cyclin D3 degradation?

We have attempted to used above mentioned drugs and monitored cyclin D3 degradation in the past.

Chloroquine does prevent cyclin D3 degradation but it does prevent infection by SARS-CoV-2 at the same time, making interpretation of these results inconclusive.

Bafilomycin A1 degrades cyclin D3 even in uninfected cells, similarly to previously reported for cyclin D1 (doi: 10.1016/j.bbrc.2009.03.051).

We believe that adding these observations would not bring any more clarity into the manuscript.

- Fig. 2C legend: Please add the staining for SARS-CoV-2 ?

Added into Figure and legend.

- Fig. 2C: Why were only syncytia looked at? Is the relocalisation of cyclin D3 also prevented by proteasome inhibition in non-syncytia?

Relocalization of cyclin D3 is also prevented by proteasome inhibition in single cell (non-syncytia). See example bellow.
SARS-CoV-2 Delta variant is well known to cause substantial syncytia and most of infection will be visible in syncytia formation. Random infected cells (not only syncytia) were selected to calculate N/C ration of cyclin D3.

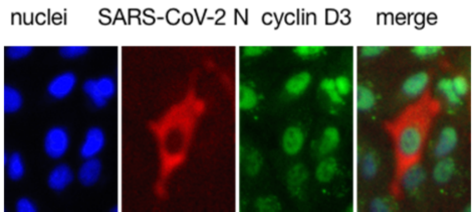

- Fig. 3A: Were the lysates infected as stated in the legends?

The cell lysates used for immunoblot were from un-infected cells and we have corrected this in the Figure legend.

- Fig. 3B,D: Please show the replicates as individual dots.

We have amended this in Figure 3. And also added individual dots/values in all Figures in the main manuscript.

- Fig 5D: Are the differences between the grey bars significant?

Only statistical significance between grey bars have been detected between NT and cylin A2 KD. See amended graphs bellow.

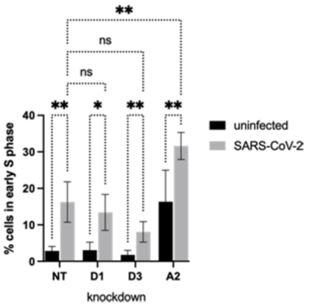

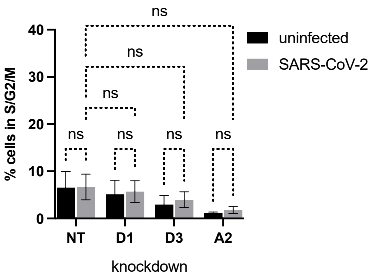

- Fig. 6B: M is not visible in the input.

Detection of M protein in cell lysates proved to be challenging. M is embedded into cellular membranes and harsh lysing condition must be used to detect it later in western blot. This harsh condition has not been used in this experiment because of fear to prevent favourable condition for co-immunoprecipitation. Nevertheless, as M has been co-immunoprecipitated from cell lysates together with cyclin D3, it is present in cell lysate in the first place.

The IP of Cyclin D3 for the nsp9 sample is less efficient, which may explain no binding.

There are some variations between individual experiments. But Figure 6A shows efficient cyclin D3 immunoprecipitation and still no binding to nsp9.

- Please replace uM with µM throughout the manuscript

We have amended this.

- Just as a comment: Please check that every person that contributed to the manuscript is listed as an author.

Every contributing persons are listed as an authors.

1st Revision - Editorial Decision **12th Sep 2022**

Dear Petra and Ravi,

Thank you for submitting your revised manuscript to The EMBO Journal. Your study has now been seen by the two referees and their comments are provided below.

As you can see the referees appreciate the introduced changes and support publication here. I am therefore very pleased to let you know that we will accept your manuscript for publication here.

Before sending you the formal acceptance letter there are just a few referees' and editorial points to address:

- You are missing 3-5 keywords

- For the data availability section: If no data is generated that needs to be deposited in a database then please state: This study includes no data deposited in external repositories.

- COI needs to be re-labelled to Disclosure and competing interests statement

- CRediT has replaced the traditional author contributions section because it offers a systematic machine-readable author contributions format that allows for more effective research assessment. Please remove the Authors Contributions from the manuscript and use the free text boxes beneath each contributing author's name in our system to add specific details on the author's contribution. More information is available in our guide to authors.

- The ToC in the appendix needs page numbers

- We include a synopsis of the paper (see http://emboj.embopress.org/). Please provide me with a general summary statement and 3-5 bullet points that capture the key findings of the paper.

- We also need a summary figure for the synopsis. The size should be 550 wide by [200-400] high (pixels). You can also use something from the figures if that is easier.

- Our publisher has also done their pre-publication check on your manuscript. When you log into the manuscript submission system you will see the file "Data Edited Manuscript file". Take a look at the word file and the comments regarding the figure legends and respond to the issues.

- We don't encourage to show statistic when N=2 (Figure 2B (left panel), Figure 5E, Figure EV3A). Please just show the data points.

- "Material and Correspondence" paragraph should be removed from the ms.

- Acknowledgements and competing interests should be moved to above the Reference list.

That should be all - Congratulations on a nice study!

Let me know if you have any further questions

Best Karin

Karin Dumstrei, PhD
Senior Editor
The EMBO Journal

Use the link below to submit your revision:

https://emboj.msubmit.net/cgi-bin/main.plex

Referee #1:

The authors have addressed my concerns

a couple of minor points
1: That Nsp1 affects D3 expression suggests that it's not degradation per se but inhibition of D3 translation as part of the viral shut-off that is the key.
2: Use E and not Env when referring to CoV envelope proteins
3:I'm not sure the authorship represents who did the experimental work well. Mlcochova should really be first, corresponding and snr author

Referee #2:

The authors have sufficiently and comprehensively addressed all of my initial concerns. Great work! The manuscript is significantly stronger now, very interesting to read and a relevant addition to our knowledge of host-virus interactions during SARS-CoV-2 infections.

Just one very minor issue remains, it would be amazing if you could use the protein abbreviations for SARS-CoV-2, i.e. M, S and E in Appendix Fig S5C and Appendix Fig S3C.

September 13th 2022

RE: Manuscript EMBOJ-2022-111653

"The role of cyclin D3 in host restriction of SARS-CoV-2 infection"

Dear Dr. Dumstrei
Thank you for your letter and for the opportunity to respond to the reviewer's comments. Please see below for point-by-point responses.

Referee #1:

The authors have addressed my concerns

a couple of minor points
1: That Nsp1 affects D3 expression suggests that it's not degradation per se but inhibition of D3 translation as part of the viral shut-off that is the key.
2: Use E and not Env when referring to CoV envelope proteins
3:I'm not sure the authorship represents who did the experimental work well. Mlcochova should really be first, corresponding and snr author

We would like to thank the reviewer.

1: That Nsp1 affects D3 expression suggests that it's not degradation per se but inhibition of D3 translation as part of the viral shut-off that is the key.

We agree with reviewer that inhibition of cyclin D3 translation by SARS-CoV-2 nsp1might be one possible explanation of cyclin D3 depletion. And we suggested so in the manuscript Discussion:

398-400"It is possible that actual translation of cyclin D is inhibited, but would not explain why other cyclins e.g. A2, E1 or B1 (all cyclins in this study have similarly short half-lives of 30-180min) (Penelova *et al*, 2005) are unaffected."

Nevertheless, our experiments where proteasome degradation was blocked and cyclin D levels were rescued (Figure 2) suggest direct degradation can not be excluded at present.

2: Use E and not Env when referring to CoV envelope proteins

We are using E or Envelope in the text now.

3:I'm not sure the authorship represents who did the experimental work well. Mlcochova should really be first, corresponding and snr author

We understand concern about the order of authors. I hope that reviewer and editor would appreciate the fact that for senior postdoctoral fellow career progression it is imperative to prove independence and being senior author is one of the means to achieve this. The issue has

been discussed in the research group with senior PI and all parties agreed on present author order.

Referee #2:

The authors have sufficiently and comprehensively addressed all of my initial concerns. Great work! The manuscript is significantly stronger now, very interesting to read and a relevant addition to our knowledge of host-virus interactions during SARS-CoV-2 infections.

We would like to thank the reviewer.

Just one very minor issue remains, it would be amazing if you could use the protein abbreviations for SARS-CoV-2, i.e. M, S and E in Appendix Fig S5C and Appendix Fig S3C.

We have corrected Fig S5 and S3.

2nd Revision - Editorial Decision 15th Sep 2022

Dear Petra and Ravi,

Thank you for submitting your revised manuscript to The EMBO Journal. I have now looked at everything and all looks good.

I am therefore very pleased to accept the MS for publication here.

Congratulations on a nice study!

With best wishes

Karin

Karin Dumstrei, PhD
Senior Editor
The EMBO Journal

Please note that it is EMBO Journal policy for the transcript of the editorial process (containing referee reports and your response letter) to be published as an online supplement to each paper. If you do NOT want this, you will need to inform the Editorial Office via email immediately. More information is available here: https://www.embopress.org/page/journal/14602075/authorguide#transparentprocess

Your manuscript will be processed for publication in the journal by EMBO Press. Manuscripts in the PDF and electronic editions of The EMBO Journal will be copy edited, and you will be provided with page proofs prior to publication. Please note that supplementary information is not included in the proofs.

You will be contacted by Wiley Author Services to complete licensing and payment information. The required 'Page Charges Authorization Form' is available here: https://www.embopress.org/pb-assets/embo-site/tej_apc.pdf - please download and complete the form and return to embopressproduction@wiley.com

EMBO Press participates in many Publish and Read agreements that allow authors to publish Open Access with reduced/no publication charges. Check your eligibility: https://authorservices.wiley.com/author-resources/Journal-Authors/open-access/affiliation-policies-payments/index.html

Should you be planning a Press Release on your article, please get in contact with embojournal@wiley.com as early as possible, in order to coordinate publication and release dates.

If you have any questions, please do not hesitate to call or email the Editorial Office. Thank you for your contribution to The EMBO Journal.

** Click here to be directed to your login page: https://emboj.msubmit.net

EMBO Press Author Checklist

Corresponding Author Name: Petra MLCOCHOVA	
Journal Submitted to: The EMBO Journal	
Manuscript Number: EMBOJ-2022-111653	

USEFUL LINKS FOR COMPLETING THIS FORM
The EMBO Journal - Author Guidelines
EMBO Reports - Author Guidelines
Molecular Systems Biology - Author Guidelines
EMBO Molecular Medicine - Author Guidelines

Reporting Checklist for Life Science Articles (updated January
This checklist is adapted from Materials Design Analysis Reporting (MDAR) Checklist for Authors. MDAR establishes a minimum set of requirements in transparent reporting in the life sciences (see Statement of Task: 10.31222/osf.io/9sm4x). Please follow the journal's guidelines in preparing your
Please note that a copy of this checklist will be published alongside your article.

Abridged guidelines for figures
1. Data
The data shown in figures should satisfy the following conditions:
- the data were obtained and processed according to the field's best practice and are presented to reflect the results of the experiments in an accurate and unbiased manner.
- ideally, figure panels should include only measurements that are directly comparable to each other and obtained with the same assay.
- plots include clearly labeled error bars for independent experiments and sample sizes. Unless justified, error bars should not be shown for technical
- if n<5, the individual data points from each experiment should be plotted. Any statistical test employed should be justified.
- Source Data should be included to report the data underlying figures according to the guidelines set out in the authorship guidelines on Data

2. Captions
Each figure caption should contain the following information, for each panel where they are relevant:
- a specification of the experimental system investigated (eg cell line, species name).
- the assay(s) and method(s) used to carry out the reported observations and measurements.
- an explicit mention of the biological and chemical entity(ies) that are being measured.
- an explicit mention of the biological and chemical entity(ies) that are altered/varied/perturbed in a controlled manner.
- the exact sample size (n) for each experimental group/condition, given as a number, not a range;
- a description of the sample collection allowing the reader to understand whether the samples represent technical or biological replicates (including how many animals, litters, cultures, etc.).
- a statement of how many times the experiment shown was independently replicated in the laboratory.
- definitions of statistical methods and measures:
  - common tests, such as t-test (please specify whether paired vs. unpaired), simple χ2 tests, Wilcoxon and Mann-Whitney tests, can be unambiguously identified by name only, but more complex techniques should be described in the methods section;
  - are tests one-sided or two-sided?
  - are there adjustments for multiple comparisons?
  - exact statistical test results, e.g., P values = x but not P values < x;
  - definition of 'center values' as median or average;
  - definition of error bars as s.d. or s.e.m.

Please complete ALL of the questions below.
Select "Not Applicable" only when the requested information is not relevant for your study.

Materials

Newly Created Materials	Information included in the manuscript?	In which section is the information available? (Reagents and Tools Table, Materials and Methods, Figures, Data Availability Section)
New materials and reagents need to be available; do any restrictions apply?	Not Applicable	

Antibodies	Information included in the manuscript?	In which section is the information available? (Reagents and Tools Table, Materials and Methods, Figures, Data Availability Section)
For **antibodies** provide the following information:<br>- Commercial antibodies: RRID (if possible) or supplier name, catalogue number and or/clone number<br>- Non-commercial: RRID or citation	Yes	Materials and Methods

DNA and RNA sequences	Information included in the manuscript?	In which section is the information available? (Reagents and Tools Table, Materials and Methods, Figures, Data Availability Section)
**Short novel DNA or RNA including primers, probes**: provide the sequences.	Yes	Materials and Methods

Cell materials	Information included in the manuscript?	In which section is the information available? (Reagents and Tools Table, Materials and Methods, Figures, Data Availability Section)
**Cell lines:** Provide species information, strain. Provide accession number in repository **OR** supplier name, catalog number, clone number, and/**OR** RRID.	Yes	Materials and Methods
**Primary cultures:** Provide species, strain, sex of origin, genetic modification status.	Not Applicable	
Report if the cell lines were recently **authenticated** (e.g., by STR profiling) and tested for mycoplasma contamination.	Not Applicable	

Experimental animals	Information included in the manuscript?	In which section is the information available? (Reagents and Tools Table, Materials and Methods, Figures, Data Availability Section)
**Laboratory animals or Model organisms:** Provide species, strain, sex, age, genetic modification status. Provide accession number in repository **OR** supplier name, catalog number, clone number, **OR** RRID.	Not Applicable	
**Animal observed in or captured from the field:** Provide species, sex, and age where possible**.**	Not Applicable	
Please detail **housing and husbandry conditions**.	Not Applicable	

Plants and microbes	Information included in the manuscript?	In which section is the information available? (Reagents and Tools Table, Materials and Methods, Figures, Data Availability Section)
**Plants:** provide species and strain, ecotype and cultivar where relevant, unique accession number if available, and source (including location for collected wild specimens).	Not Applicable	
**Microbes:** provide species and strain, unique accession number if available, and source.	Not Applicable	

Human research participants	Information included in the manuscript?	In which section is the information available? (Reagents and Tools Table, Materials and Methods, Figures, Data Availability Section)
If collected and within the bounds of privacy constraints report on age, sex and gender or ethnicity for all study participants.	Not Applicable	

Core facilities	Information included in the manuscript?	In which section is the information available? (Reagents and Tools Table, Materials and Methods, Figures, Data Availability Section)
If your work benefited from core facilities, was their service mentioned in the acknowledgments section?	Yes	Acknowledgments

Design

Study protocol	Information included in the manuscript?	In which section is the information available? (Reagents and Tools Table, Materials and Methods, Figures, Data Availability Section)
If study protocol has been **pre-registered, provide DOI in the manuscript**. For clinical trials, provide the trial registration number **OR** cite DOI.	Not Applicable	
Report the **clinical trial registration number** (at ClinicalTrials.gov or equivalent), where applicable.	Not Applicable	

Laboratory protocol	Information included in the manuscript?	In which section is the information available? (Reagents and Tools Table, Materials and Methods, Figures, Data Availability Section)
Provide DOI OR other citation details if **external detailed step-by-step protocols** are available.	Not Applicable	

Experimental study design and statistics	Information included in the manuscript?	In which section is the information available? (Reagents and Tools Table, Materials and Methods, Figures, Data Availability Section)
Include a statement about **sample size** estimate even if no statistical methods were used.	Yes	Figure legends
Were any steps taken to minimize the effects of subjective bias when allocating animals/samples to treatment (e.g. **randomization procedure**)? If yes, have they been described?	Not Applicable	
Include a statement about **blinding** even if no blinding was done.	Not Applicable	
Describe **inclusion/exclusion criteria** if samples or animals were excluded from the analysis. Were the criteria pre-established? If sample or data points were omitted from analysis, report if this was due to attrition or intentional exclusion and provide justification.	Not Applicable	
For every figure, are **statistical tests** justified as appropriate? Do the data meet the assumptions of the tests (e.g., normal distribution)? Describe any methods used to assess it. Is there an estimate of variation within each group of data? Is the variance similar between the groups that are being statistically compared?	Yes	Figure legends

Sample definition and in-laboratory replication	Information included in the manuscript?	In which section is the information available? (Reagents and Tools Table, Materials and Methods, Figures, Data Availability Section)
In the figure legends: state number of times the experiment was **replicated** in laboratory.	Yes	Figure legends
In the figure legends: define whether data describe **technical or biological replicates**.	Yes	Figure legends

Ethics

Ethics	Information included in the manuscript?	In which section is the information available? (Reagents and Tools Table, Materials and Methods, Figures, Data Availability Section)
Studies involving **human participants**: State details of **authority granting ethics approval** (IRB or equivalent committee(s), provide reference number for approval.	Not Applicable	
Studies involving **human participants**: Include a statement confirming that **informed consent** was obtained from all subjects and that the experiments conformed to the principles set out in the WMA Declaration of Helsinki and the Department of Health and Human Services Belmont Report.	Not Applicable	
Studies involving **human participants:** For publication of **patient photos**, include a statement confirming that consent to publish was obtained.	Not Applicable	
Studies involving experimental **animals**: State details of **authority granting ethics approva**l (IRB or equivalent committee(s), provide reference number for approval. Include a statement of compliance with ethical regulations.	Not Applicable	
Studies involving **specimen and field samples:** State if relevant **permits** obtained, provide details of authority approving study; if none were required, explain why.	Not Applicable	

Dual Use Research of Concern (DURC)	Information included in the manuscript?	In which section is the information available? (Reagents and Tools Table, Materials and Methods, Figures, Data Availability Section)
Could your study fall under dual use research restrictions? Please check biosecurity documents and list of **select agents and toxins** (CDC): https://www.selectagents.gov/sat/list.htm	Not Applicable	
If you used a select agent, is the security level of the lab appropriate and reported in the manuscript?	Not Applicable	
If a study is subject to dual use research of concern regulations, is the name of the **authority granting approval and reference number** for the regulatory approval provided in the manuscript?	Not Applicable	

Reporting

The MDAR framework recommends adoption of discipline-specific guidelines, established and endorsed through community initiatives. Journals have their own policy about requiring specific guidelines and recommendations to complement MDAR.

Adherence to community standards	Information included in the manuscript?	In which section is the information available? (Reagents and Tools Table, Materials and Methods, Figures, Data Availability Section)
State if relevant guidelines or checklists (e.g., **ICMJE, MIBBI, ARRIVE, PRISMA**) have been followed or provided.	Not Applicable	
For **tumor marker prognostic studies,** we recommend that you follow the **REMARK** reporting guidelines (see link list at top right). See author guidelines, under 'Reporting Guidelines'. Please confirm you have followed these guidelines.	Not Applicable	
For **phase II and III randomized controlled trials**, please refer to the **CONSORT** flow diagram (see link list at top right) and submit the CONSORT checklist (see link list at top right) with your submission. See author guidelines, under 'Reporting Guidelines'. Please confirm you have submitted this list.	Not Applicable	

Data Availability

Data availability	Information included in the manuscript?	In which section is the information available? (Reagents and Tools Table, Materials and Methods, Figures, Data Availability Section)
Have **primary datasets** been deposited according to the journal's guidelines (see 'Data Deposition' section) and the respective accession numbers provided in the Data Availability Section?	Not Applicable	
Were **human clinical and genomic datasets** deposited in a public access-controlled repository in accordance to ethical obligations to the patients and to the applicable consent agreement?	Not Applicable	
Are **computational models** that are central and integral to a study available without restrictions in a machine-readable form? Were the relevant accession numbers or links provided?	Not Applicable	
If publicly available data were reused, provide the respective **data citations in the reference list**.	Not Applicable	
